# The global burden of vascular intestinal diseases: results from the 2021 Global Burden of Disease Study and projections using Bayesian age-period-cohort analysis

**DOI:** 10.1265/ehpm.24-00206

**Published:** 2024-12-11

**Authors:** Xiqiang Zhang, Longchao Wu, Yu Li, Ze Tao, Na Li, Haoyang Zhang, Ming Ren, Kexin Wang

**Affiliations:** 1 The First Clinical College, Shandong University, Jinan, China; 2 Department of General Surgery, Qilu Hospital of Shandong University, Jinan, China

**Keywords:** Global burden of disease, Vascular intestinal disease, Estimated annual percentage change, Average annual percentage change, Socio-demographic index

## Abstract

**Background:**

Vascular intestinal disease is a major health concern that often requires emergency surgery in patients with intestinal obstruction, perforation, or bowel necrosis. We aimed to provide data on the incidence, prevalence, mortality and disability-adjusted life years (DALYs) of vascular intestinal diseases from 1990 to 2021, thereby contributing to the development of health policies.

**Methods:**

Using standardized methods from the 2021 Global Burden of Disease study, we analyzed the incidence, prevalence, mortality, and DALYs of vascular intestinal disease from the perspectives of the sociodemographic index (SDI), regional, and country, along with the corresponding estimated annual percentage changes. Additionally, we used join-point regression to identify the key time points for disease burden changes.

**Results:**

In 2021, a total of 169,432 cases [95% uncertainty interval (UI): 155,127–185,189] of vascular intestinal disease were identified worldwide. The age-standardized incidence rate decreased from 18.81 (95% UI: 16.07–21.73) in 1990 to 15.98 (95% UI: 13.99–19.10) in 2021. In 2021, the age-standardized mortality rate was 1.12/100,000 people (95% UI: 1.00–1.21). Over the 32-year period, the global DALYs rate declined by 1.44 (95% Confidence Interval: −1.55 to −1.34). Within the five SDI regions, the high-middle SDI areas recorded the peak standardized mortality rates in 2021. Regionally, the greatest increase in incidence occurred in North Africa and the Middle East. Canada recorded the greatest national incidence rate [58.35 (95% UI: 50.05–67.37)] in 2021 among 204 countries, while Russia exhibited the highest related mortality [5.64/100,000 people (95% UI: 5.19–6.11)] and DALYs rate [101.48/100,000 people (95% UI: 93.83–109.66)].

**Conclusions:**

Despite a global decline in the burden of vascular intestinal disease from 1990 to 2021, significant regional and national disparities persist and the disease burden among the elderly has increased.

**Supplementary information:**

The online version contains supplementary material available at https://doi.org/10.1265/ehpm.24-00206.

## 1. Introduction

Vascular intestinal disease (VID) has a relatively low overall incidence among gastrointestinal diseases. However, its severity and high mortality rate during acute episodes make it clinically significant [1, 2]. This group of diseases is characterized by an insufficient blood supply, leading to intestinal damage, including acute and chronic mesenteric ischemia, colonic ischemia, and intestinal vascular malformations [3]. VID is commonly observed in elderly patients with mesenteric vascular atherosclerosis, particularly acute mesenteric ischemia [4].

The risk factors include congestive heart failure, cocaine abuse, diabetes, and hypertension [5]. Mesenteric ischemia occurs when blood supply is reduced by >50% [6]. However, as ischemia progresses, vasoconstriction becomes irreversible and can quickly lead to intestinal infarction. Early symptoms of VID are nonspecific and are often confused with other common gastrointestinal diseases (gastritis, peptic ulcer, or enteritis), leading to delayed diagnosis without high clinical suspicion [7]. A meta-analysis indicated that the fatality rate of acute mesenteric venous thrombosis was 44.2%, and the mortality rate of acute non-occlusive mesenteric ischemia reached 68.5% [8]. Disease progression to bowel necrosis severely impacts the quality of life of patients, causing long-term abdominal discomfort, weight loss, and food aversion, which increases psychological stress with high examination costs, adding to the economic burden on patients and society [9, 10].

According to previous studies, the Age-standardized incidence rate (ASIR) for ischemic colitis is approximately 6.1/100,000 person-years, representing a four-fold increase between 2005 and 2009 [11]. A meta-analysis by Tamme et al. evaluated the incidence of mesenteric ischemia, revealing an average of 6.2–8.7 new cases per 100,000 residents annually and a higher prevalence in females, accounting for 58% of the total population [8]. The VID is a part of the Global Burden of Disease (GBD) assessment owing to its recognition by the World Health Organization as a significant global health concern. Previous studies have reported a declining trend in the prevalence, mortality, and disability-adjusted life years (DALYs) of VID globally over the past three decades [3]. Higher sociodemographic index (SDI) level regions exhibit a greater disease burden, with significantly higher prevalence rates than lower-level regions, whereas mortality rates have consistently declined. In 2019, Eastern Europe had a peak Age-standardized mortality rate (ASMR) of 4.32 (3.87–4.78) and was the region with the largest increase, highlighting the uneven global distribution of VID [3]. During the COVID-19 pandemic, the infection-induced hypercoagulable state significantly increased the incidence of VID [12]. Most countries are undergoing demographic changes, with an aging population becoming a trend [13]. Innovations in diagnostic technology have made diagnoses more accurate and rapid. Stem cell therapies for promoting intestinal ischemia-reperfusion injury repair [14] and interventional procedures such as angioplasty and stent implantation effectively prevent severe complications and reduce the rates of bowel resection and mortality [15, 16]. These advancements emphasize the need to keep abreast of the disease burden.

The GBD study is an effective resource for acquiring knowledge regarding the epidemiological features of VID [17]. The 2021 GBD repository has updated the latest epidemiological data, improving statistical models and calculation methods (including Bayesian hierarchical and spatiotemporal models) with particular attention to the ramifications of the COVID-19 pandemic. It incorporates additional health indicators and data sources and supports thorough evaluations of health hazards involving 369 diseases, injuries, and conditions and 88 risk indicators in 204 countries and territories [18]. The COVID-19 pandemic has dramatically disrupted global health systems, exacerbating existing inequalities and leading to significant declines in health outcomes across numerous countries [19]. This study comprehensively describes the burden of VID in 2021 using GBD statistical models, exploring the latest trends in incidence and prevalence at global, regional, and national levels while emphasizing age and gender differences and trends across different SDI levels. This analysis helps track disease progression, identify health disparities and inequities, and assist epidemiologists and health policymakers in developing targeted public health interventions to improve global and regional health.

## 2. Methods

### 2.1 Disease overview and data download

This cross-sectional analysis utilized the Global Health Data Exchange query tool to collect updated information on VID, including standardized disease definitions. Because the data used for this study were obtained from a secondary analysis and were anonymized, the Ethics Review Committee of Qilu Hospital of Shandong University exempted the requirement for patient consent. The GBD 2021 study systematically evaluated global health status and disease burden by enhancing data collection and processing methods. The VID data analyzed in this study were derived from the GBD 2021 study, and the relevant data can be accessed using the Global Health Data Exchange tool (https://ghdx.healthdata.org/gbd-2021/sources). The methodological framework for data collection, modeling, and analysis in the GBD 2021 study has been extensively documented in previous publications [20, 21]. A detailed description of this is provided in Appendix 1. In the GBD 2021 study, causes are classified into four levels, ranging from level 1, which includes non-communicable diseases, infectious diseases, maternal and neonatal conditions, nutritional deficiencies, and injuries, to level 4, which includes alcoholic cardiomyopathy. VID is classified as a level 3 cause [22]. GBD 2021 provides a standardized definition for VID, encompassing diseases involving intestinal vascular abnormalities, such as mesenteric ischemia, ischemic colitis, and intestinal vascular malformations. These conditions cause reduced or interrupted intestinal blood flow, resulting in various symptoms and complications. VID cases were identified using the International Classification of Diseases, 10th Revision (ICD-10) codes, including K55.0–K55.3, K55.8, and K55.9 (Appendix 2). The data inputs, exclusion criteria, and modeling strategies for VID are detailed in Appendix 3. The incidence, prevalence, mortality, and DALY measurements for vascular intestinal disorders at global, regional, and national levels from 1990 to 2021 were retrieved using the GBD Results Tool (https://vizhub.healthdata.org/gbd-results/), including 95% uncertainty interval (UI).

### 2.2 Study dimensions

We utilized the SDI to explore the relationship between VID and the socio-economic levels of various countries and regions. The SDI incorporates crucial factors such as the average years of education for those aged ≥15, total fertility rate for women aged <25 years, and lag-distributed income, categorizing countries and regions into five SDI levels (high, high-middle, middle, low-middle, low-middle, and low) that range from zero to one [23]. As the value approaches one, the socioeconomic development level increases. We also assessed the disease burden at regional, national, and global levels, explored the differences between different age groups and sexes, and described their temporal trends.

### 2.3 Join-point regression analysis

The segmented regression model proposed by Kim et al. was applied to establish long-term trends in disease distribution through join-point regression. Each join-point divides the time frame into different intervals, and the annual percent change (APC) for each stage is calculated [https://surveillance.cancer.gov/help/joinpoint/setting-parameters/method-and-parameters-tab/apc-aapc-tau-confidence-intervals/estimate-average-percent-change-apc-and-confidence-interval] to fit and optimize the trends [24]. We used the ‘segment’ package (version 2.1-3) [https://cran.r-project.org/web/packages/segmented/index.html] to perform the segmented regression and determine the optimal data partition points through model fitting. The ‘EPi’ package (version 2.56) [https://cran.r-project.org/web/packages/Epi/index.html] was used to calculate the APC and its 95% CI. The following is the specific formula: APC = (e^β^ − 1) × 100, where β is the slope estimated from the log-linear regression model log(y) ∼ year. The 95% CI is calculated as (e^(β±1.96×SE(β)^ − 1) × 100. If the lower bound of the APC and its 95% CI are greater than zero, the age-standardized rate (ASR) shows an increasing trend; otherwise, it indicates a decreasing trend.

### 2.4 Statistical analysis

According to the GBD algorithms, the incidence, prevalence, DALYs, and mortality rates are reported as ASR per 100,000 people with 95% UI to reflect data reliability and model robustness [25]. To measure age-standardized dynamic changes over specific periods, the linear modeling method proposed by Hankey et al. was applied, assuming the natural logarithm of ASR fits the linear regression model y = α + βx + ε, where y equals ln (ASR) and x represents the calendar year. The Estimated annual percentage changes (EAPC) was 100 × (exp(β) − 1) [26]. If both the EAPC and its 95% CI are above 0, this indicates an increase in the corresponding ASR and vice versa for a decrease. Smooth spline models were used to highlight the relationship between the disease burden of VID and SDI in 21 areas and 204 countries, fitted with the weighted least squares method to automatically determine the degree and position of nodes (knots) based on data and span parameters for visualization. Spearman correlation analysis was applied to evaluate the relationship between the SDI and ASR and R indices and p-values [18]. The Pearson method was used to measure the correlation between the EAPCs and ASR/SDI. In addition, the Bayesian Age-Period-Cohort (BAPC) model was used to predict changes in the burden of VID from 2022 to 2035 [27]. The BAPC model was applied using the ‘BAPC’ R package (version 0.0.36) in conjunction with the ‘INLA’ package (version 22.05.07) (https://folk.ntnu.no/andrerie/software.html), which allowed for efficient Bayesian inference using integrated nested Laplace approximation (INLA) [28]. R version 4.4.2 was used for all the analyses. The ‘BAPC()’ and ‘INLA()’ functions were employed to fit the age-period-cohort model and estimate posterior distributions of model parameters, respectively. The data were structured into 5-year age intervals (specifically, age groups were divided into under 5 years, 5–9 years, 10–14 years, 15–19 years, and so forth up to 95 years and above, covering a total of 20 age groups), with time periods spanning 1990–2021 in 1-year increments. Population data and disease rates for each age group and period were prepared in a matrix format, where rows represented age groups and columns represented time periods. These data were fed into the ‘BAPC()’ function, specifying the age and time dimensions accordingly. Standard population data were sourced from the GBD Study 2021, available at [IHME GBD 2021 Demographics] (https://ghdx.healthdata.org/record/ihme-data/global-burden-disease-study-2021-gbd-2021-demographics-1950-2021). This dataset provides age-specific population weights from 1950 to 2021, which were used for standardization in our analysis. The 95% UIs for the predicted rates were calculated using the posterior distributions derived from the BAPC model. These were obtained through the ‘INLA’ package, which provides posterior marginal distributions for each parameter. The UIs were computed as the 2.5th and 97.5th percentiles of posterior distributions.

All p-values are bilateral, with p < 0.05 deemed statistically significant. All statistical analyses were conducted using the R software (version 4.4.2).

## 3. Results

### 3.1 Global trends

**Fig. 1:**
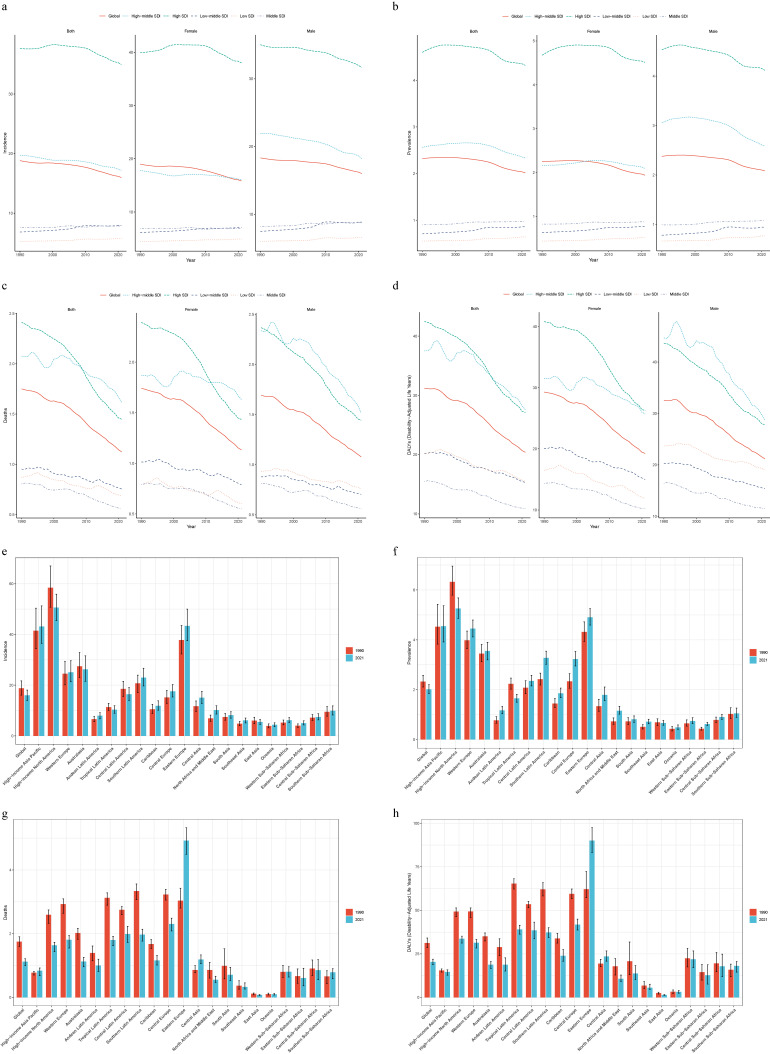
Changes in the burden of VID and a comparison between 1990 and 2021. (a, e) Age-standardized incidence rate; (b, f) Age-standardized prevalence rate; (c, g) Age-standardized mortality rate; (d, h) Age-standardized DALYs rate.

In 2021, 169,432 cases (95% UI: 155,127–185,189) of VID were identified, with 81,840 [48.30%] and 87,592 [51.70%] cases in males and females, respectively (Table 1). The age-standardized prevalence rate (ASPR) was 2.02 cases per 100,000 people (95% UI: 1.85–2.20). From 1990 to 2021, this rate decreased by 0.50 cases per 100,000 people (95% CI: −0.58 to −0.41), with a greater reduction in females (0.51 per 100,000 people) than males (0.47 per 100,000 people) (Table 1). However, the number of global VID cases increased from 757,507 (95% UI: 647,682–879,672) in 1990 to 1,347,021 (95% UI: 1,178,809–1,532,645) in 2021. The corresponding ASIR declined from 18.81 (95% UI: 16.07–21.73) to 15.98 (95% UI: 13.99–19.10), with an EAPC of −0.48 (95% CI: −0.54 to −0.41) (Table 1, Table S1, and Fig. 1e). The number of deaths remained stable at 91,515 cases (95% UI: 81,930–98,538), and the ASMR for males and females were similar [1.08 (95% UI: 0.99–1.15) for males vs. 1.13 (95% UI: 0.98–1.25) for females]. A reduction of 1.09 cases per 100,000 people (95% CI: −1.22 to −0.97) in ASMR over the 32 years was observed (Table 1). The age-standardized disability-adjusted life years rate (ASDR) dropped significantly from 31.21 cases per 100,000 people (95% UI: 28.71–34.12) in 1990 to 20.39 cases per 100,000 people (95% UI: 18.78–21.94) in 2021, with a reduction of 34.85% and 34.28% in males and females, respectively (Table 1, Table S1, and Fig. 1h). Overall, from 1990 to 2021, global mortality, DALYs, and the incidence and prevalence of VID exhibited downward trends, with the latter two declining more significantly (Table 1 and Fig. 1).

**Table 1:** The number of disease burden cases of VID in 2021, along with ASR and EAPC.

**Characteristics**	**Incidence (95% uncertainty interval)**			**Prevalence (95% uncertainty interval)**			**Deaths (95% uncertainty interval)**			**DALYs (95% uncertainty interval)**		
			
	**Cases, 2021**	**ASIR, 2021**	**EAPC1990–2021**	**Cases, 2021**	**ASPR, 2021**	**EAPC1990–2021**	**Cases, 2021**	**ASMR, 2021**	**EAPC1990–2021**	**Cases, 2021**	**ASDR, 2021**	**EAPC1990–2021**
Global	1347020.54 (1178808.68– 1532644.51)	15.98 (13.99–18.10)	−0.48 (−0.54–−0.41)	169431.96 (155127.21– 185188.93)	2.02 (1.85–2.20)	−0.50 (−0.58–−0.41)	91514.92 (81930.47– 98537.59)	1.12 (1.00–1.21)	−1.09 (−1.22–−0.97)	1708446.84 (1580467.77– 1836379.45)	20.39 (18.78–21.94)	−1.44 (−1.55–−1.34)
Sex												
Male	630636.26 (551814.92– 723125.83)	16.02 (14.07–18.21)	−0.38 (−0.43–−0.34)	81839.93 (74695.87– 90116.73)	2.08 (1.91–2.28)	−0.47 (−0.55–−0.39)	38363.68 (35749.53– 41131.97)	1.08 (0.99–1.15)	−1.21 (−1.30–−1.11)	821073.93 (768304.88– 884232.05)	21.19 (19.77–22.78)	−1.47 (−1.55–−1.38)
Female	716384.28 (627210.28– 812563.63)	15.83 (13.84–17.93)	−0.54 (−0.62–−0.46)	87592.04 (80647.04– 95323.17)	1.938 (1.78–2.12)	−0.51 (−0.60–−0.42)	53151.24 (46072.69– 58762.37)	1.13 (0.98–1.25)	−1.01 (−1.14–−0.87)	887372.91 (789796.99– 972878.82)	19.25 (17.14–21.10)	−1.40 (−1.52–−1.29)
SDI												
High-middle SDI	320004.27 (276175.11– 367271.08)	17.15 (14.92–19.72)	−0.39 (−0.43–−0.35)	43546.75 (40602.82– 46812.75)	2.33 (2.16–2.53)	−0.31 (−0.42–−0.20)	31154.04 (28182.93– 33214.36)	1.62 (1.46–1.73)	−0.35 (−0.48–−0.23)	536408.87 (496659.18– 568791.48)	27.60 (25.54–29.26)	−1.01 (−1.16–−0.86)
Low SDI	37187.52 (30675.84– 45006.71)	5.75 (4.91–6.62)	0.31 (0.28–0.34)	4618.22 (3885.32– 5651.05)	0.64 (0.56–0.73)	0.51 (0.46–0.56)	3132.01 (2457.06– 3822.04)	0.69 (0.53–0.86)	−0.55 (−0.64–−0.45)	97903.41 (76468.49– 118875.20)	15.49 (12.17–18.89)	−0.95 (−1.03–−0.87)
High SDI	662807.322 (583941.680– 750524.881)	34.959 (30.875–39.713)	−0.211 (−0.280–−0.141)	82162.032 (76519.528– 88296.845)	4.32 (3.95–4.73)	−0.28 (−0.36–−0.20)	34780.98 (29676.33– 37341.45)	1.45 (1.26–1.54)	−1.33 (−1.48–−1.18)	575245.05 (517026.26– 606380.65)	27.12 (24.81–28.40)	−1.54 (−1.67–−1.42)
Low-middle SDI	124277.74 (104954.67– 145255.35)	7.94 (6.81–9.13)	0.58 (0.52–0.64)	13866.27 (11832.48– 16394.21)	0.86 (0.75–0.99)	0.72 (0.65–0.79)	9281.34 (7593.12– 11675.20)	0.76 (0.62–0.950)	−0.53 (−0.61–−0.44)	218767.75 (180476.03– 270671.15)	15.29 (12.60–19.05)	−0.97 (−1.02–−0.91)
Middle SDI	201756.30 (171981.28– 232676.95)	7.84 (6.69–9.00)	0.13 (0.10–0.16)	25064.29 (21991.52– 28701.87)	0.97 (0.86–1.11)	0.27 (0.24–0.30)	13041.57 (11832.08– 14399.49)	0.56 (0.50–0.62)	−0.93 (−1.04–−0.83)	277912.32 (253302.36– 304511.17)	10.89 (9.92–11.97)	−1.22 (−1.29–−1.15)
Region												
East Asia	112448.59 (91472.83– 134328.14)	5.48 (4.56–6.47)	−0.52 (−0.70–−0.33)	13397.96 (11472.56– 15508.66)	0.66 (0.57–0.77)	−0.17 (−0.29–−0.05)	1348.80 (1133.32– 1546.51)	0.08 (0.06–0.09)	−0.77 (−1.27–−0.28)	26698.42 (23126.43– 30393.61)	1.39 (1.20–1.58)	−2.30 (−2.87–−1.73)
Oceania	428.99 (347.38– 520.67)	4.38 (3.65–5.14)	0.29 (0.23–0.34)	50.94 (41.258– 64.70)	0.48 (0.41–0.59)	0.33 (0.27–0.40)	8.04 (6.17– 10.05)	0.10 (0.08–0.12)	0.00 (−0.08–0.08)	335.63 (262.85– 421.54)	3.15 (2.44–3.92)	0.05 (−0.04–0.15)
Southeast Asia	38402.60 (31678.88– 45096.63)	6.12 (5.10–7.03)	0.79 (0.76–0.82)	4474.45 (3874.52– 5225.62)	0.72 (0.63–0.82)	1.15 (1.12–1.19)	1597.22 (1302.28– 2207.63)	0.33 (0.26–0.46)	−0.21 (−0.31–−0.11)	31594.82 (26727.20– 42710.38)	5.56 (4.66–7.59)	−0.78 (−0.87–−0.70)
Central Asia	12883.22 (10811.20– 15176.91)	15.07 (12.75–17.54)	1.06 (0.94–1.18)	1528.12 (1307.64– 1838.71)	1.79 (1.57–2.11)	1.22 (1.09–1.35)	806.94 (717.30– 913.22)	1.19 (1.06–1.33)	0.90 (0.820–0.98)	18294.63 (16079.09– 20964.87)	23.53 (20.78–26.69)	0.33 (0.19–0.47)
Eastern Europe	143811.76 (123087.40– 165938.22)	43.35 (37.54–50.00)	0.69 (0.59–0.79)	16046.64 (15110.40– 16982.44)	4.90 (4.59–5.25)	0.570 (0.46–0.68)	17714.27 (16162.81– 19168.93)	4.93 (4.50–5.33)	1.80 (1.69–1.91)	315911.29 (291631.73– 341050.30)	90.08 (83.27–97.43)	1.20 (0.99–1.41)
Central Europe	31219.00 (27334.37– 35732.65)	17.59 (15.37–20.25)	0.24 (0.13–0.35)	6231.18 (5787.24– 6650.62)	3.23 (2.95–3.53)	0.86 (0.70–1.02)	5485.79 (4960.75– 5921.11)	2.31 (2.09–2.49)	−0.62 (−0.83–−0.41)	94270.41 (86686.15– 101548.51)	41.80 (38.68–45.00)	−1.26 (−1.46–−1.05)
Western Europe	216856.36 (183736.23– 254407.46)	25.11 (21.34–29.64)	0.20 (0.11–0.29)	40134.62 (37572.63– 42628.79)	4.45 (4.12–4.80)	0.21 (0.08–0.34)	20706.60 (17397.43– 22386.21)	1.81 (1.55–1.94)	−1.15 (−1.30—1.00)	311839.86 (275404.89– 333499.30)	31.42 (28.39–33.29)	−1.45 (−1.58–−1.32)
Australasia	13620.59 (11046.03– 16539.37)	26.23 (21.55–31.55)	−0.03 (−0.08–0.01)	1858.01 (1698.44– 2024.71)	3.55 (3.20–3.91)	0.02 (−0.04–0.07)	694.85 (586.93– 774.8)	1.13 (0.96–1.26)	−1.57 (−1.71–−1.42)	10584.84 (9290.77– 11639.87)	18.76 (16.65–20.55)	−2.03 (−2.20–−1.87)
High-income Asia Pacific	149362.13 (127764.37– 173223.42)	43.15 (36.53–51.28)	0.24 (0.14–0.35)	15334.28 (13666.99– 17450.48)	4.55 (3.91–5.37)	0.095 (−0.01–0.20)	5401.36 (4237.02– 6068.14)	0.84 (0.68–0.93)	0.09 (−0.12–0.29)	74823.44 (62256.00– 82025.60)	14.66 (12.80–15.84)	0.06 (−0.17–0.29)
Central Latin America	40501.80 (34546.70– 47158.28)	16.44 (13.97–19.10)	−0.52 (−0.60–−0.44)	5807.09 (5292.42– 6375.30)	2.35 (2.15–2.58)	0.33 (0.27–0.38)	4703.50 (4085.46– 5280.09)	1.99 (1.73–2.23)	−0.96 (−1.03–−0.88)	94901.72 (82818.91– 106452.78)	38.51 (33.64–43.21)	−0.98 (−1.10–−0.87)
Southern Latin America	19604.59 (16771.93– 22904.76)	23.01 (19.71–26.69)	0.32 (0.24–0.41)	2798.06 (2590.90– 3010.07)	3.27 (3.02–3.55)	1.01 (0.94–1.08)	1790.77 (1598.19– 1943.07)	1.97 (1.77–2.14)	−1.39 (−1.48–−1.30)	32677.97 (30012.48– 35028.37)	37.31 (34.42–39.97)	−1.51 (−1.61–−1.40)
Andean Latin America	4792.44 (4064.81– 5531.37)	7.94 (6.75–9.16)	0.67 (0.55–0.78)	708.60 (622.62– 813.55)	1.17 (1.04–1.33)	1.53 (1.42–1.64)	564.243 (457.23– 677.75)	1.00 (0.81–1.20)	−1.04 (−1.13–−0.95)	11008.08 (8865.69– 13362.10)	18.75 (15.16–22.75)	−1.4 (−1.58–−1.26)
High-income North America	311223.60 (279075.22– 345849.39)	50.62 (45.46–55.89)	−0.51 (−0.60–−0.43)	31643.51 (29619.50– 33808.33)	5.26 (4.86–5.68)	−0.68 (−0.73–−0.63)	11337.976 (9857.03– 12060.74)	1.64 (1.44–1.73)	−1.09 (−1.33–−0.85)	213716.77 (196002.37– 223858.94)	33.70 (31.22–35.19)	−1.51 (−1.69–−1.33)
Caribbean	6180.78 (5289.37– 7209.44)	11.83 (10.15–13.84)	0.45 (0.41–0.48)	970.24 (881.47– 1078.13)	1.85 (1.68–2.07)	0.80 (0.75–0.84)	631.72 (554.09– 718.45)	1.16 (1.02–1.322)	−1.01 (−1.12–−0.91)	12802.43 (11171.69– 14704.57)	23.87 (20.81–27.43)	−1.20 (−1.28–−1.12)
South Asia	131060.72 (109699.71– 157105.64)	8.17 (7.00–9.54)	0.36 (0.26–0.46)	13389.39 (11258.63– 16200.15)	0.81 (0.69–0.95)	0.42 (0.30–0.53)	8807.27 (6560.26– 11762.78)	0.71 (0.53–0.95)	−0.79 (−0.92–−0.66)	195644.66 (146485.20– 260531.81)	13.71 (10.23–18.26)	−1.48 (−1.57–−1.39)
North Africaand Middle East	49513.23 (41076.91– 58815.76)	10.19 (8.60–11.87)	1.16 (1.08–1.23)	5652.95 (4826.92– 6720.51)	1.15 (1.02–1.33)	1.515 (1.469–1.56)	2043.23 (1741.87– 2458.18)	0.55 (0.47–0.67)	−1.43 (−1.51–−1.35)	46574.97 (40474.44– 57276.77)	10.66 (9.20–12.86)	−1.83 (−1.95–−1.71)
Southern Sub-Saharan Africa	7004.37 (5705.40– 8640.75)	9.92 (8.27–11.81)	0.21 (0.11–0.30)	762.60 (631.842– 949.71)	1.05 (0.89–1.26)	0.14 (0.02–0.26)	401.75 (313.03– 457.42)	0.79 (0.58–0.90)	0.55 (0.35–0.74)	11251.46 (9337.46– 12845.46)	18.08 (14.55–20.60)	0.62 (0.26–0.97)
Eastern Sub-Saharan Africa	8852.72 (7508.96– 10440.06)	5.05 (4.30–5.96)	0.82 (0.76–0.88)	1212.57 (1049.45– 1416.51)	0.62 (0.56–0.69)	1.24 (1.17–1.30)	855.42 (508.71– 1263.35)	0.61 (0.36–0.92)	−0.16 (−0.24–−0.08)	24055.53 (14009.95– 35019.53)	12.74 (7.65–18.71)	−0.52 (−0.60–−0.44)
Tropical Latin America	25844.96 (22279.50– 29754.62)	10.32 (8.93–11.90)	0.41 (0.06–0.76)	4159.20 (3768.20– 4545.68)	1.65 (1.50–1.82)	−0.61 (−0.78–−0.44)	4482.39 (4055.64– 4775.56)	1.80 (1.62–1.92)	−1.64 (−1.73–−1.55)	99904.43 (93037.64– 105924.57)	39.08 (36.31–41.47)	−1.83 (−1.92–−1.75)
Central Sub-Saharan Africa	4957.72 (4114.87– 5907.89)	7.45 (6.30–8.75)	0.11 (−0.09–0.30)	667.06 (569.19– 810.49)	0.91 (0.81–1.01)	0.41 (0.14–0.68)	366.10 (242.32– 510.06)	0.85 (0.56–1.18)	−0.26 (−0.30–−0.233)	10833.59 (7268.09– 15305.94)	17.86 (11.94–24.88)	−0.28 (−0.33–−0.22)
Western Sub-Saharan Africa	18450.37 (14993.52– 22720.57)	6.19 (5.21–7.25)	0.57 (0.53–0.62)	2604.50 (2135.45– 3296.94)	0.74 (0.64–0.87)	0.44 (0.41–0.48)	1766.70 (1371.02– 2147.82)	0.80 (0.64–0.96)	0.25 (0.16–0.34)	70721.90 (51803.17– 89106.61)	21.92 (17.12–26.70)	−0.06 (−0.15–0.03)

VID, Vascular intestinal diseases; ASR, Age-standardized rates; ASIR, Age-standardized incidence rate; ASPR, Age-standardized prevalence rate; ASMR, Age-standardized mortality rate; ASDR, Age-standardized disability-adjusted life years rate; EAPC, Estimated annual percentage changes; DALYs, Disability-adjusted life years; SDI, Socio-demographic Index.

### 3.2 SDI region levels

In 2021, high SDI regions had the highest number of newly diagnosed VID cases, with an ASIR of 34.96/100,000 people (95% UI: 30.88–39.71) (Table 1). In contrast, the low SDI regions had the lowest ASIR [5.75 (95% UI: 4.91–6.62)], indicating a significant disparity (Table 1). From 1990 to 2021, changes in ASIR varied by development level: high and high-middle SDI areas exhibited a downward trajectory, with EAPCs of −0.21 (95% CI: −0.28 to −0.14) and −0.39 (95% CI: −0.43 to −0.35), respectively. Conversely, low, middle, and low-middle SDI areas experienced a rising trend, with the largest growth in the low-middle SDI region at 0.58 (95% CI: 0.52–0.64) and the smallest increase in the middle SDI areas at 0.13 (95% CI: 0.10–0.16) (Table 1 and Fig. 1a).

Similar to incidence trends, the prevalence rate in high SDI areas was significantly higher than in other areas, while the high-middle SDI areas had a prevalence closer to the global average, with an ASPR of 2.33/100,000 people (95% UI: 2.16–2.53) (Table 1). During the same period (1990–2021), the prevalence rates in high and high-middle SDI regions declined, with a greater reduction in the high-middle SDI region, EAPC of −0.31 (95% CI: −0.42 to −0.20). In contrast, prevalence rates in low, low-middle, and middle SDI regions rose, with the smallest increase in the middle SDI region at 0.27 (95% CI: 0.24–0.30) (Table 1 and Fig. 1b).

Overall, mortality rates in all regions showed a declining pattern. The high SDI region exhibited a significant decrease in mortality rates, reducing by 1.33 per 100,000 people (95% CI: 1.18–1.49) (Table 1 and Fig. 1c). By 2021, the high-middle SDI region had the highest ASMR, surpassing the high SDI region at 1.62/100,000 people (95% UI: 1.46–1.73) with 31,154 deaths (95% UI: 28,183–33,214) (Table 1 and Fig. 1c). The middle SDI region maintained the lowest ASMR over 32 years, reaching 0.56 per 100,000 people (95% UI: 0.50–0.62) in 2021 (Table 1, Fig. 1c).

Similarly, no direct correlation between SDI and ASDR existed; however, all regions showed a downward trend (Fig. 1d). For more information on the DALYs, see Table 1 and Fig. 1d.

### 3.3 Changes in geographic regions

Analysis of 21 geographic regions revealed that the burden of VID relative to other digestive system diseases was not particularly high (Figs. S1a and Sb). Geographically, in 2021, high-income North America had the highest number of new cases (311,224 cases [95% UI: 279,075–345,849]), whereas Oceania had the fewest (429 cases [95% UI: 347–521]) (Table 1 and Fig. 1e). The highest ASIR was in high-income North America [50.6 (95% UI: 45.46–55.89)], followed by Eastern Europe [43.35 (95% UI: 37.54–50.00)], while Oceania had the lowest incidence rate [4.38 (95% UI: 3.65–5.14)] (Table 1). Between 1990 and 2021, East Asia, Central Latin America, high-income North America, and Australasia exhibited declining trends, whereas the incidence rate rose in the other 17 regions, with the largest increase in North Africa and the Middle East [EAPC: 1.16 (95% CI: 1.08–1.23)] (Table 1).

Eight regions (including high-income Asia Pacific and Eastern Europe) had prevalence rates higher than the global average, whereas 13 regions (e.g., the Caribbean, South Asia, and Southeast Asia) had rates below the global average (Table 1 and Fig. 1f). Compared to 1990, the ASPR in high-income North America reduced from 6.33 (95% UI: 5.79–6.96) to 5.26 (95% UI: 4.86–5.68) in 2021, remaining the zone with the highest prevalence rate, and exhibiting the largest reduction [EAPC: −0.68 (95% CI: −0.73 to −0.63)] (Table 1, Table S1 and Fig. 1f).

Since 1998, the mortality rates in Eastern Europe have shown a sharp upward trend (Fig. S1c). By 2021, Eastern Europe had the highest mortality rate, with an ASMR of 4.93/100,000 people (95% UI: 4.50–5.33) and 17,714 deaths (95% UI: 16,163–19,169), increasing from 7,959 deaths (95% UI: 7,387–9,028) in 1990 (Table 1, Table S1, and Fig. S1c). It ranked first among the six regions with increasing mortality trends, with an EAPC of 1.80 (95% CI: 1.69–1.91). Oceania remained stable [EAPC: 0.002 (95% CI: −0.08–0.08)] (Table 1 and Fig. 1g). Among other regions, Tropical Latin America demonstrated the largest decline in ASMR [EAPC: −1.64 (95% CI: −1.73 to −1.55)]. Eastern Asia and Oceania had the lowest mortality rates at 0.08/100,000 people (95% UI: 0.06–0.09) and 0.10/100,000 people (95% UI: 0.08–0.12), respectively (Table 1 and Fig. S1c). Additionally, several regions, including Southeast Asia, North Africa, the Middle East, Eastern Sub-Saharan Africa, and South Asia, have maintained low mortality rates over the years (Fig. S1c).

Globally, the ASDR for VID exhibited a declining trend (Fig. 1d). The most significant reductions were observed in East Asia [EAPC: −2.30 (95% CI: −2.87 to −1.73)] (Table 1 and Fig. S1d). Conversely, Eastern Europe had the highest ASDR [90.08 (95% UI: 83.27–97.43)], followed by Central Europe [41.80 (95% UI: 38.68–45.00)] and Tropical Latin America [39.08 (95% UI: 36.31–41.47)] (Table 1 and Fig. 1h). Eastern Europe also had the highest number of DALYs, reaching 315,911 cases (95% UI: 291,632–341,050), and was one of the few regions with an increasing trend [EAPC 1.20 (95% CI: 0.99–1.41)]. Ten regions, including Central Latin America, had DALY rates exceeding the global average, whereas 11 regions, including Andean Latin America, had DALY rates below the global average (Table 1 and Fig. 1h).

### 3.4 National level

**Fig. 2:**
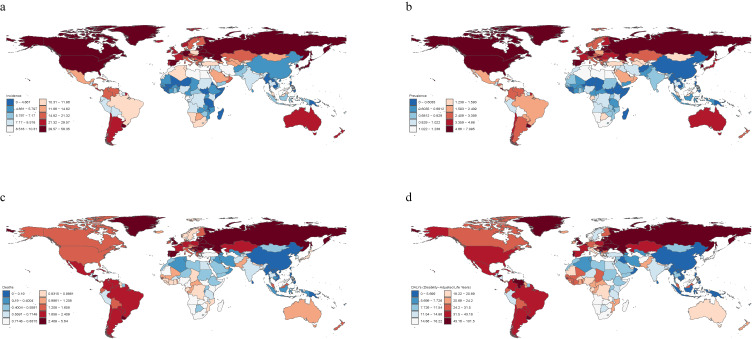
Global distribution of vascular intestinal diseases burden in 2021. (a) Age-standardized incidence rate; (b) Age-standardized prevalence rate; (c) Age-standardized mortality rate; (d) Age-standardized DALYs rate.

In 2021, the United States had the highest number of new cases (271,867 cases [95% UI: 244,550–300,816]), with an ASIR of 49.67/100,000 people (95% UI: 44.90–54.70), ranking fourth globally. Canada had the highest incidence rate [58.35 (95% UI: 50.05–67.37)] (Table S2 and Fig. 2a). Overall, most countries showed an increasing trend in incidence rates, with Indonesia exhibiting the most significant increase [EAPC 2.89 (95% CI: 2.63–3.15)] (Table S2 and Fig. S2a). The ASPR of VID ranged from 1.85 to 2.20 per 100,000 people (Table 1). Montenegro [7.09/100,000 people (95% UI: 6.54–7.63)], Estonia [6.53/100,000 people (95% UI: 6.08–7.04)], Belgium [6.38/100,000 people (95% UI: 5.92–6.85)], Canada [6.18/100,000 people (95% UI: 5.62–6.80)], Spain [6.17/100,000 people (95% UI: 5.71–6.61)], and Uruguay [6.01/100,000 people (95% UI: 5.59–6.42)] had the highest ASPR (Table S2 and Fig. 2b). In contrast, Somalia [0.28/100,000 people (95% UI: 0.25–0.32)], Papua New Guinea [0.37/100,000 people (95% UI: 0.30–0.46)], Myanmar [0.38/100,000 people (95% UI: 0.31–0.46)], Timor-Leste [0.43/100,000 people (95% UI: 0.37–0.50)], Laos [0.44/100,000 people (95% UI: 0.38–0.52)], and the Philippines [0.45/100,000 people (95% UI: 0.37–0.54)] had the lowest ASPR (Table S2 and Fig. 2B). Between 1990 and 2021, the ASPR varied significantly among the countries (Fig. S2b). Specifically, Equatorial Guinea [EAPC: 3.75 (95% CI: 3.46–4.04)], Taiwan [EAPC: 3.26 (95% CI: 2.70–3.82)], and Malaysia [EAPC: 2.99 (95% CI: 2.82–3.16)] exhibited the most significant increases. In contrast, the largest decrease was seen in Sweden [EAPC: −2.22 (95% CI: −2.37 to −2.08)] (Table S2 and Fig. S2b).

Russia (13,639 cases [95% UI: 12,561–14,766]), the United States (10,069 cases [95% UI: 8,817–10,710]), and India (6,703 cases [95% UI: 4,827–9,413]) reported the highest number of deaths (Table S2). The ASMR ranged from 1.00 to 1.21 cases per 100,000 people, with Russia reporting the highest rate at 5.64 per 100,000 people (95% UI: 5.19–6.11), followed by Armenia, Latvia, Montenegro, and Lithuania (Table S2 and Fig. 2c). Notably, between 1990 and 2021, the ASMR of Georgia increased dramatically, with a rise of 14.53 (95% CI: 12.63–16.47), followed by Taiwan at 7.62 (95% CI: 5.96–9.31). Puerto Rico showed a substantial reduction in mortality [EAPC: −3.51 (95% CI: −3.75 to −3.26)] (Table S2 and Fig. S2c).

In 2021, the ASDR ranged from 18.78 to 21.94 cases per 100,000 people (Table 1). The top five countries with the highest rates were Russia [101.48 per 100,000 people (95% UI: 93.83–109.66)], Latvia [93.02 per 100,000 people (95% UI: 80.86–105.49)], Armenia [89.75 per 100,000 people (95% UI: 74.82–103.41)], Uruguay [85.79 per 100,000 people (95% UI: 78.25–92.93)], and Lithuania [84.70 per 100,000 people (95% UI: 73.86–94.69)] (Table S2 and Fig. 2d). Surprisingly, China had a high number of DALYs cases at 17,820 (95% UI: 14,669–21,150), but its ASDR was only 0.96/100,000 people (95% UI: 0.79–1.14), ranking last and continuing to decline (Table S2 and Fig. 2d). Table S2 and Fig. S2d provide additional information on the changes in DALY rates.

### 3.5 Age and gender patterns and overall time trends

**Fig. 3:**
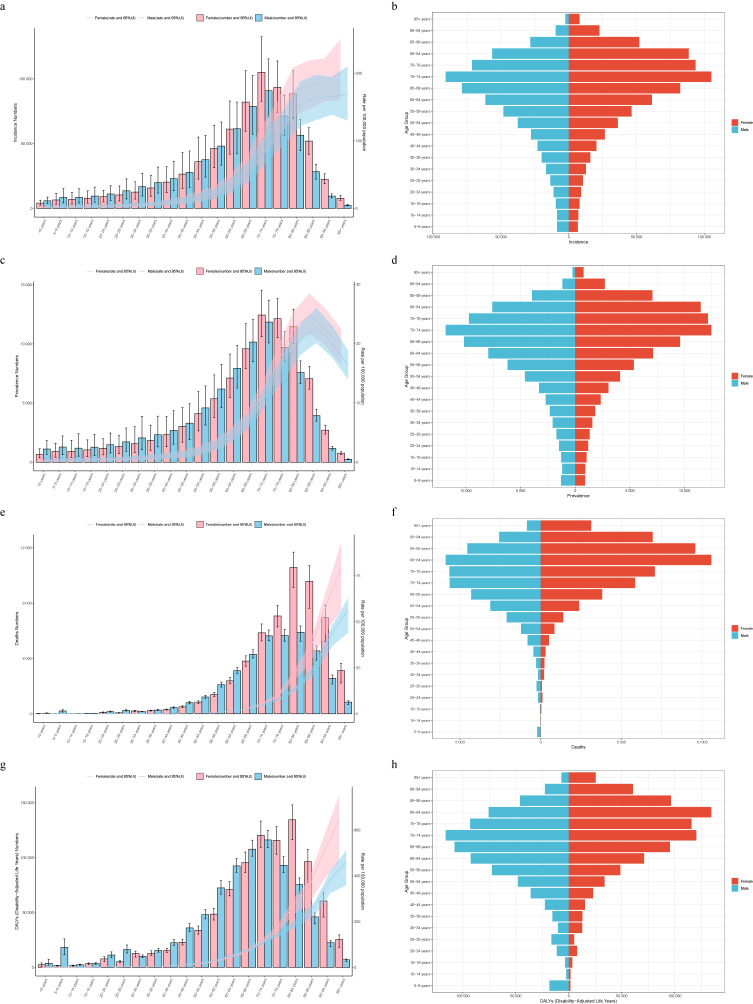
Distribution and trends (by Age and Sex) of the burden of VID in 2021. (a, b) Incidence; (c, d) Prevalence; (e, f) Mortality and (g, h) DALYs.

**Fig. 4:**
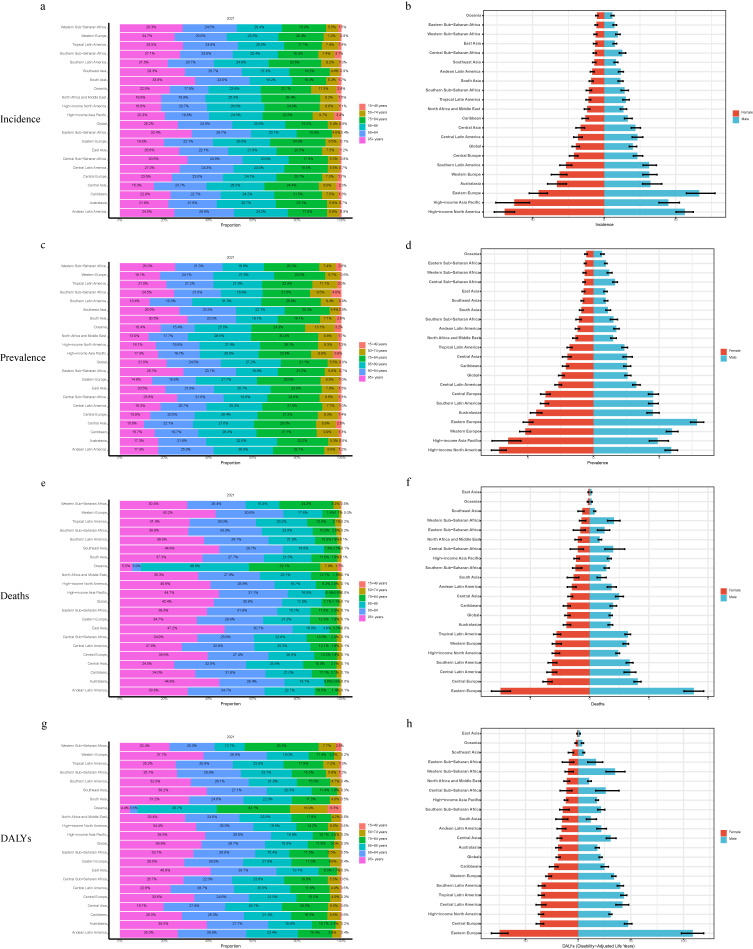
Proportion of disease burden of VID by age and gender globally and regions in 2021. (a, b) Age-standardized incidence rate; (c, d) Age-standardized prevalence rate; (e, f) Age-standardized mortality rate; (g, h) Age-standardized DALYs rate.

In 2021, most new cases worldwide were concentrated in individuals aged 60–85 years, with more males than females aged <65 years, and the opposite trend for those aged >65 years (Figs. 3a and 3b). The incidence rate increased with age, with individuals >85 years of age accounting for 74.6% of ASIR cases (Figs. 3a and 4a). A noticeable sex disparity existed in the elderly population, with females outnumbering males (Figs. 3a and 4a). In South Asia, individuals aged >95 accounted for one-third of the incidence rate (Fig. 4a). Detailed data for the other regions are presented in Figs. 4a and 4b. From 1990 to 2021, the >95 age group exhibited the most significant “hump-shaped” change, with a decline after 2010 (Fig. S3a). Trends varied across the SDI regions, see additional Fig. 3 for details. The global distribution of cases across the age groups was similar to that of the incidence rates (Figs. 3c, 3d, and 4c). However, the highest ASPR occurred in the 85–89 years age cohort (Fig. 4c). This pattern was also observed in Western Europe, Australasia, and East Asia, while in South Asia, individuals >95 were predominant (Figs. 4c and 4d). Global and SDI region-ASPR changes are presented in Figs. S3g–l.

The mortality and DALY rates increased with age, with more significant sex differences (higher in females) (Figs. 3e–h, 4f, and 4h). An exception was Oceania, where the 90–94 and >95 years age groups accounted for <10% of the combined data (Figs. 4e and 4g). Globally, apart from the 15–49 years age cohort, all other cohorts exhibited a declining trend in mortality and DALY rates, with more pronounced declines in the older age groups (Figs. S3m and S3s). High-SDI regions followed the global trend, whereas other regions exhibited significant fluctuations (Figs. S3r and S3x). For instance, low and low-middle SDI, as well as high-middle SDI areas, exhibited “mountain peak” changes in the four older age groups, with the >95 age group showing the most considerable fluctuation (Fig. S3). In contrast, the middle SDI regions exhibited a “sharp peak” pattern in the same age group, peaking in 2005, with significant gender differences (Figs. S3p and S3v).

### 3.6 Correlation of SDI with regional, national, and EAPC disease burden

**Fig. 5:**
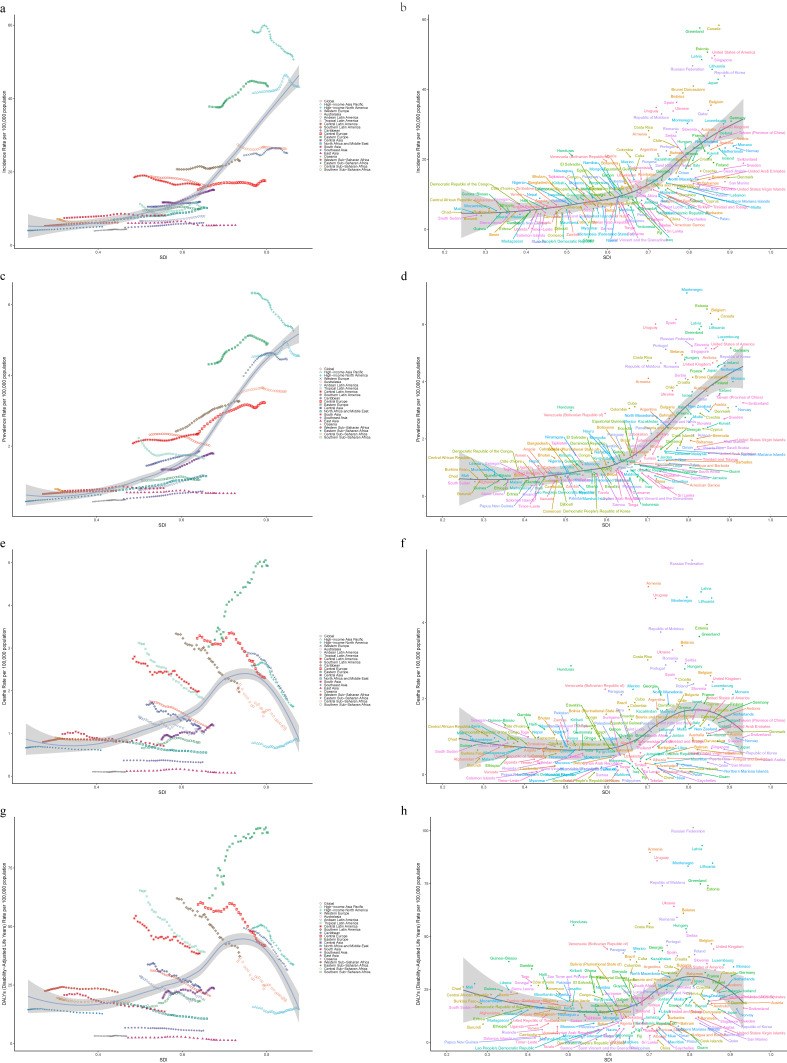
Correlation between ASR of VID and SDI at the national and regional levels in 2021. (a, b) Age-standardized incidence rate; (c, d) Age-standardized prevalence rate; (e, f) Age-standardized mortality rate; (g, h) Age-standardized DALYs rate.

SDI was positively associated with ASIR in 21 regions (R = 0.82, p < 0.0001) and 204 countries (R = 0.84, p < 0.0001) with a similar prevalence rate pattern (Table S3 and Figs. 5a–d). In contrast, the association among the SDI, mortality, and DALY rates was not as prominent. The correlation between SDI and mortality rates was R = 0.47 (p < 0.0001) for the regions and R = 0.365 (p < 0.0001) for the countries. For DALYs rates, it was R = 0.40 (p < 0.0001) for the regions and R = 0.23 (p < 0.0001) for the countries (Table S3 and Figs. 5e–f). Additionally, EAPC demonstrated a direct correlation with changes in ASMR (R = 0.20, p = 0.004) and DALYs rate (R = 0.16, p = 0.02) but no association with incidence and prevalence rates (Fig. S4). No significant correlation was observed between the SDI and changes in ASPR and ASMR (Fig. S4).

### 3.7 Join-point regression analysis

**Fig. 6:**
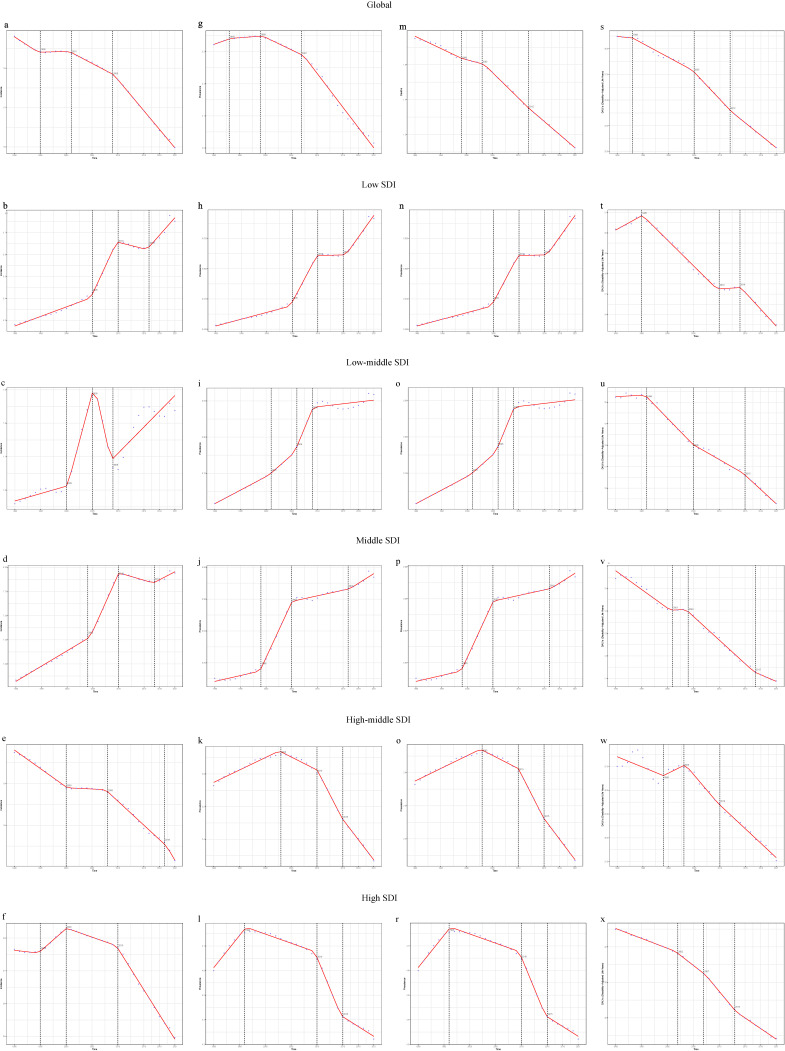
Join-point Regression Analysis of temporal trends in the burden of VID from 1990 to 2021. (a–f) Age-standardized incidence rate; (g–l) Age-standardized prevalence rate; (m–r) Age-standardized mortality rate; (s–x) Age-standardized DALYs rate.

From 1990 to 2021, the global burden of VID showed a decreasing trend. Notably, the ASMR showed the most significant decline between 2012 and 2021 (APC = −2.241; 95% CI: −2.939 to −1.538; p < 0.0001), and the DALYs rate declined most notably between 2005 and 2012 (APC = −2.029; 95% CI: −2.187 to −1.871; p < 0.0001) (Table S4 and Figs. 6m and 6s). Join-point regression analysis revealed varying trends in the SDI regions (Fig. 6). Specifically, the high-middle SDI region exhibited an increase in ASPR from 1990 to 2003 (APC = 0.354; 95% CI: 0.300–0.407; p < 0.0001), and the high SDI region exhibited an increase from 1990 to 1996 (APC = 0.561; 95% CI: 0.495–0.627; p < 0.0001). Regarding ASMR, the high SDI region exhibited a consistent decline, with the sharpest decrease occurring between 2010 and 2015 (APC = −2.947; 95% CI: −3.105 to −2.788; p < 0.0001) (Table S4, Table S5, and Figs. 5a–d). Detailed trends in the incidence and DALY rates are shown in Fig. 6.

The three regions with the highest prevalence rates were also analyzed. The ASPR in high-income North America exhibited a steady decline, while the high-income Asia Pacific revealed significant “spike-like” changes, with a sharp increase between 2005 and 2009 (APC = 1.505; 95% CI: 1.029–1.982; p < 0.0001), followed by a sharp decrease (APC = −0.846; 95% CI: −0.969 to −0.723; p < 0.0001) (Table S4, Figs. S5a and S5e). Conversely, Eastern Europe is on the rise (Fig. S5c). Although the ASMR in the high-income Asia-Pacific region demonstrated a general increasing trend over 32 years, it experienced significant fluctuations, gradually decreasing between 1990 and 2005 and achieving a substantial increase between 2005 and 2018 (APC = 1.825; 95% CI: 1.614–2.037; p < 0.0001) (Table S4 and Fig. S5f).

### 3.8 Predicted trends in disease burden

**Fig. 7:**
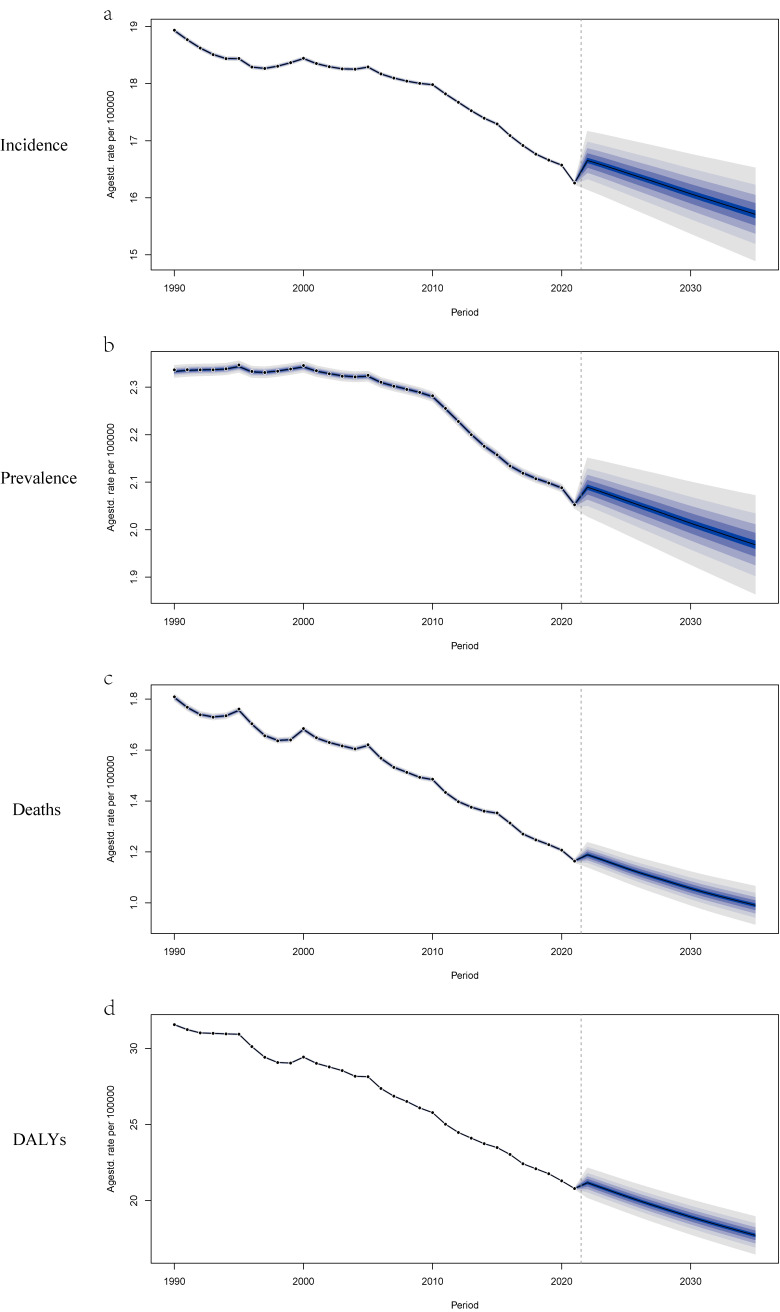
Trends in the burden of VID: observed rates (1990–2021) and predicted rates (2022–2035). (a) Age-standardized incidence rate; (b) Age-standardized prevalence rate; (c) Age-standardized mortality rate; (d) Age-standardized DALYs rate. The blue region in shows the upper and lower limits of the 95% UI.

The predicted trends and changes in the ASIR, ASPR, ASMR, and ASDR for VID are shown in Table S5 and Fig. 7. By 2035, the global trends are projected to be 15.71 (95% UI: 14.74–16.68), 1.97 (95% UI: 1.85–2.09), 0.99 (95% UI: 0.90–1.08), and 17.72 (95% UI: 16.25–19.18), respectively. The predictions of ASIR, ASPR, ASMR, and ASDR across different age groups are presented in Table S6 and Figs. S6–S9. Overall, the burden of VID is expected to continue declining from 2022 to 2035 (Fig. 7).

## 4. Discussion

Presently, no serological tests can identify VID early [29], and while CT angiography and magnetic resonance angiography have enhanced diagnostic capabilities, they are often costly [30]. Early intervention before irreversible damage occurs remains challenging and poses a significant global public health issue. Our study describes the burden of VID in 2021, including the prevalence and mortality across 204 countries, 21 regions, and five SDI levels, and explores disease variations among various age groups and sexes. Unlike previous studies [3], we analyzed the global burden of VID following the COVID-19 pandemic for the first time and identified the key years of change in various indicators. Additionally, this study predicts future trends in the burden of VID.

By 2021, the number of new cases reached 1,347,021. Owing to the uncertainty measurement method, new cases ranged from 1,178,809 to 1,532,645; these figures should be interpreted with caution. We observed that lower SDI regions had a smaller disease burden, which can be partly attributed to inadequate routine screening and lack of physician awareness [31], leading to underdiagnosis and misdiagnosis, thereby masking the true disease burden. However, incomplete data reporting and statistical biases may not accurately reflect the actual situation. Low-income regions often face challenges in data collection infrastructure and healthcare resource allocation, which may lead to an underestimation of the disease burden in low-SDI areas [32]. However, the burden associated with VID may be genuinely lower in these regions, which also exhibit a gradual increase in incidence and prevalence, potentially reflecting demographic changes, such as increased birth rates and urbanization, which require large-scale statistical surveys to reveal their impact. The significant decline in global prevalence and mortality rates in 2021 relative to 1990 indicates substantial global efforts to diagnose and treat VID [33]. Interventional techniques [34] include catheterization of the proximal mesenteric vessels with papaverine to relieve vasospasm and the use of drug-eluting stents to reduce restenosis rates. Endovascular treatment of acute mesenteric ischemia has a 100% success rate, with studies confirming lower mortality rates than open surgery (16.7% vs. 33.3%) [35]. Additionally, diagnostic advances, such as portable ultrasound for early emergency diagnosis and near-infrared fluorescence imaging for dynamic blood flow perfusion observation have been made [36]. However, challenges persist in countries and regions where screening and prevention are not feasible. Continuous monitoring of the disease epidemiology is required to better understand the current population and healthcare status.

We observed that although the disease burden in higher SDI regions continued to decline, the ASIR in 2021 was still more than six times higher than that in low SDI regions. Moreover, the ASMR in middle-high SDI regions reached 1.62 per 100,000 people, exhibiting the highest burden over the past 32 years. While high SDI regions have abundant resources, managing chronic or geriatric diseases remains a challenge, particularly with an increasing aging population [37]. The disease risk disease also increases throughout life, leading to persistently high mortality and DALY rates in some cases. In contrast, the younger population structure in low SDI regions may result in less significant VID-related mortality. Additionally, the primary health threats in high-SDI regions are often non-communicable diseases associated with aging, such as cardiovascular diseases or cancer [38], while acute illnesses, such as infectious diseases and malnutrition, continue to pose major health risks in low-SDI regions. Therefore, despite the advanced healthcare infrastructure and medical technologies available in high SDI areas, the high incidence of chronic diseases increases the DALYs burden, weakening the correlation between SDI and DALYs rates. Studies have suggested that residents of high-income regions consume diets rich in fats and refined sugars, which can lead to dyslipidemia and increase the risk of arteriosclerosis and thrombosis, thereby affecting the vascular health of the intestines [39]. Moreover, unhealthy lifestyle habits such as smoking [40] and physical inactivity [41] are more prevalent in these regions, further exacerbating the burden of vascular diseases. Currently, limited direct evidence of the correlation between VID and education or income levels exists. However, indirect factors may also be associated with the risk of developing such diseases. For instance, populations with lower income and educational attainment are often more vulnerable to risk factors associated with health inequalities such as poor dietary quality [42], which increases the risk of developing VID. Future research should delve deeper into these relationships to elucidate the specific mechanisms involved.

Countries such as the United States, Europe, and Australia have issued guidelines on VID and promoted related research [1, 43]. However, differences in the treatment, recommended drugs, and management exist across national guidelines, and innovative therapies and technologies have yet to be widely tested in large clinical trials. Uncertainty also exists regarding medical insurance reimbursements, economic burdens, and prognoses. Our study presents the latest developments in 2021 and data on changes over 32 years, thereby facilitating cross-national research. This provides crucial evidence for formulating effective public health policies and clinical management strategies, particularly in addressing the disease burden in high-SDI regions, which has become a key priority. Firstly, the high prevalence of chronic and geriatric diseases in high SDI regions [38], particularly among populations with unhealthy lifestyles [39], necessitates policy advocacy and public health programs that promote healthy diets, increase physical activity, and reduce harmful behaviors such as smoking and excessive alcohol consumption. Public health policies should be modified to enhance disease prevention. For instance, restricting the advertising and sale of high-fat, high-sugar foods, increasing taxes on these products to reduce consumption, or tightening regulations on unhealthy ingredients in food can not only lower the incidence of VID but also reduce the burden of other non-communicable diseases associated with aging. Secondly, improving chronic disease management and early intervention strategies is key to alleviating the burden of VID: (1) For high-risk populations, new predictive models combining clinical indicators and imaging techniques can be developed to identify potential risks, and non-invasive methods such as biomarker detection and serum testing (e.g., lactate levels) can be used to detect early symptoms of ischemic bowel disease; (2) Introducing higher-resolution imaging technologies, such as microvascular ultrasound, can detect subtle blood flow changes [44], while advances in endoscopy and capsule endoscopy provide a more direct observation of intestinal vasculature; (3) For ischemic bowel disease caused by different factors, personalized treatment plans should be developed. For instance, genetic testing and drug metabolism monitoring can help optimize the use of anticoagulants and antiplatelet drugs, thereby reducing the risk of vascular blockage and re-ischemia. Further advancement of minimally invasive vascular interventions (such as stenting and balloon angioplasty) [45] and research into novel treatment methods such as stem cell therapy [14] and tissue engineering could lower mortality rates; (4) Clinical guidelines should place greater emphasis on comprehensive management, including long-term anticoagulation therapy, vascular health monitoring, and the establishment of regular follow-up mechanisms. Finally, policymakers should address these health inequities. Despite the overall abundance of resources in high SDI regions, health disparities remain significant among the low-income and low-education populations. Strengthening the primary healthcare infrastructure in areas where these populations are concentrated, expanding health insurance coverage, and establishing special funds are promising approaches.

We found that the ASPR increased exponentially with age and that females were more commonly affected. This aligns with the previously reported trends described in a study on ischemic colitis, which showed an increase in prevalence with age [42]. This reflects the compelling concern of population aging and an increase in vascular-related diseases in most countries [45]. Elderly individuals often have atherosclerosis [4], atrial fibrillation, and congestive heart failure, all of which increase the risk of developing VID. For instance, 47% of the patients with mesenteric ischemia are diagnosed with atrial fibrillation [46]. Diabetes and chronic obstructive pulmonary disease also increase this risk [42, 47]. Complications increase with age, with bowel resection surgery often leading to short bowel syndrome, causing malabsorption, persistent diarrhea, weight loss, and requiring long-term parenteral nutrition in severe cases [48]. Another study confirmed that the prevalence of VID is higher in women than in men [1]. Estrogen promotes vasodilation, reduces inflammation, and inhibits atherosclerosis [49]. However, estrogen levels significantly decrease before and after menopause, increasing the risk of intestinal ischemia. In addition, elderly women are prone to diseases associated with atherosclerosis and often experience concerns such as osteoporosis and reduced mobility [50], further exacerbating intestinal ischemia. Insufficient dietary fiber intake, which is particularly common among older women, often worsens intestinal health problems [51]. Ischemia and inflammation can lead to intestinal nerve damage, resulting in chronic pain and digestive dysfunction, severely impacting quality of life, increasing DALY rates, and imposing a heavy family burden [52]. Despite a decline in standardized DALY rates over 32 years, the number of DALYs in 2021 remained high at 1,708,447. These findings suggest the need for increased awareness and education about VID, recognition of the current prognosis, and investigation of the reasons for the decline in DALY rates to better inform clinical decision-making.

Previous studies indicated that COVID-19 can induce a hypercoagulable state, thereby increasing the risk of intestinal vascular thrombosis [12, 53]. Additionally, in low-SDI regions, poor sanitation, overburdened healthcare systems due to the pandemic delaying patient visits and treatments [54], and unstable food supplies leading to unhealthy diets have contributed to the continued increase in incidence and prevalence rates. In contrast, high-SDI regions were better able to adapt to the COVID-19 pandemic, with enhanced public health monitoring indirectly improving basic healthcare services. As a result, the incidence and mortality rates in these regions continued to decline; however, the join-point regression analysis revealed no significant inflection point in the disease burden trend in 2019. This may be attributed to VID being a rare complication of COVID-19 [55]. A large database study revealed that only 2.1% of 18,185 patients with acute mesenteric ischemia had COVID-19 [56], supporting this finding. Moreover, as a chronic disease, VID was likely not significantly affected during the pandemic owing to the availability of telemedicine and chronic disease management programs, enabling patients to continue receiving essential care [57]. Public health emergency efforts during the pandemic may have affected the collection and reporting of disease burden data, particularly in low-SDI regions, where data collection capabilities are limited [58]. The pandemic may have exacerbated this issue, resulting in data gaps or delays, with reported incidence and mortality rates potentially much lower than the actual figures. As healthcare resources are reallocated and electronic health record systems become more widely adopted, future trends in VID burden may vary. The short timespan after the onset of the pandemic may not have fully captured these trends. Continued vigilance is necessary regarding the potential long-term impacts following the pandemic, especially as healthcare resources return to normal, which could result in data surges or hidden burdens caused by delayed chronic disease management.

The BAPC model used in this study is based on the following assumptions: 1) Future incidence or mortality rates will follow trends similar to historical data, and 2) Independence between different age groups exists. Potential uncertainties in the model arise from the variability in the underlying data, unknown long-term trend shifts, and possible unrecognized cohort effects. The BAPC model assumes that the changes in disease rates over time can be attributed to age, period, and cohort effects. This assumption is critical for the predictive power of the model. However, unforeseen changes in disease dynamics or interventions that are not captured in historical data could introduce bias into future projections. Additionally, the uncertainty intervals reflect variability in the posterior estimates but may not fully account for the impact of structural changes in population health or healthcare systems. Our study has some limitations: (1) The GBD database includes data from sources of varying quality, with limitations in data completeness and accuracy, particularly in rural and low-income regions where healthcare infrastructure is underdeveloped. These areas may experience underreporting, data gaps, or biases, leading to underestimation of the disease burden. Additionally, some countries with smaller populations lack available data, and estimates rely on predictive covariates and neighboring regions, which may further reduce data accuracy [59]. When assessing the burden of VID, insufficient screening, diagnostic challenges, and limited healthcare services may result in the misrepresentation of the prevalence and mortality rates. Although the GBD collaborators use complex statistical models to correct data inaccuracies, acknowledging these limitations is crucial for interpreting the results of our study. This recognition also highlights the importance of improving future data collection efforts to better reflect the actual burden of VID, particularly in low SDI regions. (2) Although the GBD database uses DALY rates to encompass multiple dimensions of health burden, GBD-related indicators do not fully incorporate cultural, social, and behavioral factors, potentially overlooking the impact of psychosocial health. (3) A classification system for VID has not yet been established, limiting further understanding of this disease.

## 5. Conclusion

A comprehensive overview of the disease burden of VID in 2021 is provided in the present study using the GBD database, which encompasses global, national, and regional levels. A general downward trend in the global disease burden from 1990 to 2021 is indicated, with the expectation of a continued decline. A significant disease burden was observed among the elderly and female populations, as well as in higher SDI regions, underscoring the necessity for healthcare providers to develop targeted strategies to mitigate these high-burden areas and affected groups. Further utilization of these findings can yield important insights for innovative concepts and therapies.

## Abbreviations

GBD: Global Burden of Disease
VID: Vascular intestinal diseases
ASR: Age-standardized rates
DALYs: Disability-adjusted life years
ASIR: Age-standardized incidence rate
ASPR: Age-standardized prevalence rate
ASMR: Age-standardized mortality rate
ASDR: Age-standardized disability-adjusted life years rate
EAPC: Estimated annual percentage changes
SDI: Socio-demographic Index
APC: Annual percentage change
UI: Uncertainty Interval
CI: Confidence Interval
BAPC: Bayesian Age-Period-Cohort
INLA: Integrated nested Laplace approximation

## Supplementary information

**Additional file 1:** **Appendix 1.** GBD data collection, modeling/analysis, and dissemination.

**Additional file 2:** **Appendix 2.** List of ICD-10 codes about vascular intestinal disorders-K55.

**Additional file 3:** **Appendix 3.**

**Additional file 4:** **Figure S1** Proportion of disease burden for various digestive diseases across different regions in 2021 and changes in the disease burden of vascular intestinal diseases in different regions from 1990 to 2021. (a, c) Age-standardized mortality rate; (b, d) Age-standardized DALYs rate.

**Additional file 5:** **Figure S2** The estimated annual percentage change of the age-standardized rate of incidence (a), prevalence (b), mortality (c), and DALYs (d) of vascular intestinal diseases from 1990 to 2021.

**Additional file 6:** **Figure S3** Temporal trends in the burden of vascular intestinal diseases by age globally and in SDI regions. (a–f) Age-standardized incidence rate; (g–l) Age-standardized prevalence rate; (m–r) Age-standardized mortality rate; (s–x) Age-standardized DALYs rate.

**Additional file 7:** **Figure S4** Correlation between Estimated Annual Percentage Change (EAPC) in 2021 and age-standardized rates of vascular intestinal diseases and SDI. (a) EAPC is not significantly correlated with ASIR/SDI; (b) EAPC is not significantly correlated with ASPR/SDI; (c) EAPC is positively correlated with ASMR but not significantly correlated with SDI; (d) EAPC is positively correlated with ASDR but not significantly correlated with SDI. In the left figures, circles represent countries, while in the right figures, circles represent countries for which Human Development Index data is available. The size of each circle is proportional to the number of (a) incident cases, (b) prevalent cases, (c) deaths, and (d) DALYs. The ρ values and p-values were obtained from Pearson correlation analysis.

**Additional file 8:** **Figure S5** Join-point Regression Analysis of temporal trends in the burden of vascular intestinal diseases in the Top 3 regions with the highest prevalence from 1990 to 2021. (a, c, e) Age-standardized prevalence rate; (b, d, f) Age-standardized mortality rate.

**Additional file 9:** **Figure S6** Trends of age-standardized incidence rate of vascular intestinal diseases across age groups: observed rates (1990–2021) and predicted rates (2022–2035). The blue region in shows the upper and lower limits of the 95% UI.

**Additional file 10:** **Figure S7** Trends of age-standardized prevalence rate of vascular intestinal diseases across age groups: observed rates (1990–2021) and predicted rates (2022–2035). The blue region in shows the upper and lower limits of the 95% UI.

**Additional file 11:** **Figure S8** Trends of age-standardized mortality rate of vascular intestinal diseases across age groups: observed rates (1990–2021) and predicted rates (2022–2035). The blue region in shows the upper and lower limits of the 95% UI.

**Additional file 12:** **Figure S9** Trends of age-standardized DALYs rate of vascular intestinal diseases across age groups: observed rates (1990–2021) and predicted rates (2022–2035). The blue region in shows the upper and lower limits of the 95% UI.

**Additional file 13:** **Table S1** The number of incident cases, prevalent cases, deaths, and DALYs of vascular intestinal diseases in 1990, along with corresponding ASR.

**Additional file 14:** **Table S2** The number of incident cases, prevalent cases, deaths, and DALYs of vascular intestinal diseases at the National Level in 2021, along with corresponding ASR and temporal trends.

**Additional file 15:** **Table S3** Correlation between SDI and disease burden across regions and countries, and EAPC.

**Additional file 16:** **Table S4** APC of VID from 1990 to 2021 for the World, SDI Regions, and the Top 3 Regions with the highest prevalence.

**Additional file 17:** **Table S5** Age-standardized rates (95% UI) of vascular intestinal diseases from 1990 to 2021 and predicted age-standardized rates (95% UI) from 2022 to 2035.

**Additional file 18:** **Table S6** Age-standardized rates (95% UI) of vascular intestinal diseases or different age groups from 1990 to 2021 and predicted age-standardized rates (95% UI) from 2022 to 2035.

## Declarations

### Data availability

This study utilized data from the Global Burden of Disease (GBD) 2021 database, maintained and provided by the Institute for Health Metrics and Evaluation (IHME). The GBD database is an open-access resource aimed at providing health statistics at global, regional, and national levels, covering the prevalence of various diseases, injuries, and risk factors, as well as their impact on public health.

Data Access: Access to and use of the GBD database are unrestricted. Researchers and the public can obtain the data through IHME’s official website (http://www.healthdata.org/).

Data Coverage: (1) Diseases and Injuries: Includes various health conditions such as infectious diseases, maternal and child health, accidental injuries, and violence; (2) Risk Factors: Includes behavioral, metabolic, and environmental risk factors such as smoking, unhealthy diet, and air pollution; (3) Demographic Characteristics: Data stratified by age, gender, and region.

Data Citation: We follow the IHME citation guidelines-Global Burden of Disease Collaborative Network. Global Burden of Disease Study 2021 (GBD 2021) Results. Seattle, United States: Institute for Health Metrics and Evaluation (IHME), 2020. Available from http://ghdx.healthdata.org/gbd-results-tool.

Statement Details: The GBD database is freely accessible, and any researcher can use the data in accordance with IHME’s data use policy. To ensure transparency and reproducibility, the GBD data used in this study can be obtained through the aforementioned methods.
