# Supplementary material for: The global burden of vascular intestinal diseases: results from the 2021 Global Burden of Disease Study and projections using Bayesian age-period-cohort analysis
Source: Environ Health Prev Med. 2024 Dec 11;29:71. doi: 10.1265/ehpm.24-00206 (PMC11653002; doi:10.1265/ehpm.24-00206)
Supplement: Supplementary file 1 — Additional file 1: Appendix 1. GBD data collection, modeling/analysis, and dissemination. [file ehpm-29-071-s001.docx]

**Appendix 1. GBD data collection, modeling/analysis, and dissemination**

The Global Burden of Disease (GBD) study is a comprehensive research initiative aimed at systematically assessing the health status and disease burden of populations worldwide. An international network comprising over 11,500 collaborators from 164 countries and territories contributed to the generation of GBD metrics through data provision, review, and analysis. GBD data collection involves diverse sources, including epidemiological surveys, hospital records, vital registration systems, disease surveillance systems, and additional sources such as academic papers and policy reports (https://ghdx.healthdata.org/gbd-2021/sources). The data is standardized using the International Classification of Diseases (ICD) codes to ensure accuracy and comparability (<https://ghdx.healthdata.org/record/ihme-data/gbd-2021-cause-icd-code-mappings>).

Sophisticated modeling tools, such as DisMod-MR and Spatiotemporal Gaussian Process Regression (ST-GPR), are employed to estimate prevalence, incidence, and mortality rates. Data processing includes corrections for heterogeneity and biases, as well as uncertainty analysis through Monte Carlo simulations. Key health metrics used are Disability-Adjusted Life Years (DALYs), Years of Life Lost (YLLs), and Years Lived with Disability (YLDs) (https://www.healthdata.org/gbd/methods-appendices-2021/cancers). Dissemination of GBD findings is achieved through scientific publications (https://www.healthdata.org/research-analysis/gbd-publications), and interactive tools like GBD Compare and Viz Hub (https://www.healthdata.org/research-analysis/gbd-data). These tools facilitate the exploration and comparison of health data across regions and time periods. The primary goal of GBD findings is to provide a comprehensive framework for understanding global and local health trends, thereby supporting evidence-based health decision-making and resource allocation.
