# Supplementary material for: The global burden of vascular intestinal diseases: results from the 2021 Global Burden of Disease Study and projections using Bayesian age-period-cohort analysis
Source: Environ Health Prev Med. 2024 Dec 11;29:71. doi: 10.1265/ehpm.24-00206 (PMC11653002; doi:10.1265/ehpm.24-00206)
Supplement: Supplementary file 2 — Additional file 2: Appendix 2. List of ICD-10 codes about vascular intestinal disorders-K55. [file ehpm-29-071-s002.docx]

**Appendix 2.List of ICD-10 codes about vascular intestinal disorders-K55.**

| K55.0 | Acute vascular disorders of intestine |  |
| --- | --- | --- |
|  | Acute | fulminant ischaemic colitis |
|  |  | intestinal infarction |
|  |  | small intestine ischaemia |
|  | Mesenteric (artery)(vein) | embolism |
|  |  | infarction |
|  |  | thrombosis |
|  |  | Subacute ischaemic colitis |
| K55.1 | Chronic vascular disorders of intestine |  |
|  | Chronic ischaemic | colitis |
|  |  | enteritis |
|  |  | enterocolitis |
|  | Ischaemic stricture of intestine |  |
|  | Mesenteric | atherosclerosis |
|  |  | vascular insufficiency |
| K55.2 | Angiodysplasia of colon |  |
| K55.3 | Angiodysplasia of small intestine |  |
| K55.8 | Other vascular disorders of intestine |  |
| K55.9 | Vascular disorder of intestine, unspecified |  |
