# Supplementary material for: The global burden of vascular intestinal diseases: results from the 2021 Global Burden of Disease Study and projections using Bayesian age-period-cohort analysis
Source: Environ Health Prev Med. 2024 Dec 11;29:71. doi: 10.1265/ehpm.24-00206 (PMC11653002; doi:10.1265/ehpm.24-00206)
Supplement: Supplementary file 3 — Additional file 3: Appendix 3. [file ehpm-29-071-s003.docx]

**Appendix 3.**

**Vascular intestinal disorders**


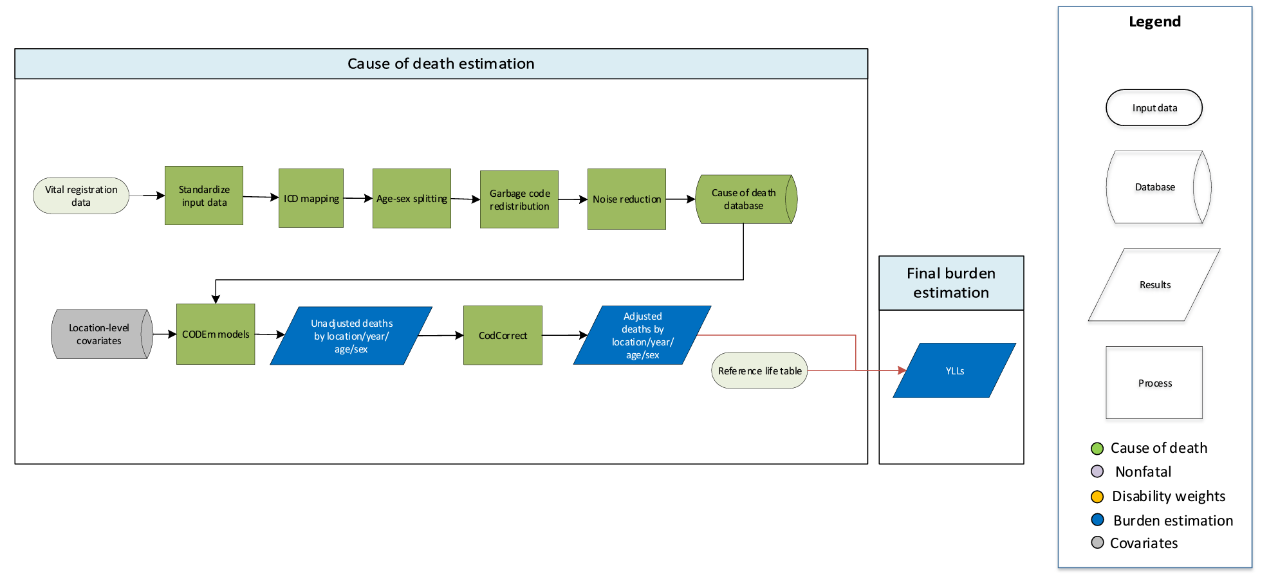
Flowchart

**Input data and methodological summary for vascular intestinal disorders**

Input data

Data used to estimate mortality of vascular intestinal disorders consisted of vital registration data from the cause of death (COD) database. Outliers were identified by systematic examination of datapoints for all location-years. Data were excluded if they violated well-established age or time trends, and data in instances where garbage code redistribution and noise reduction, in combination with small sample sizes, resulted in unreasonable cause fractions.

**Modelling strategy**

The estimation strategy used for fatal vascular intestinal disorders is largely similar to methods used in GBD 2019. A standard CODEm model with location-level covariates was used to model deaths due to vascular intestinal disorders (see appendix section on CODEm method for details). Separate models were conducted for male and female mortality, and age restrictions for death estimations included 2 years for lower bound (in GBD 2019, the lower bound was set at 1 year) and 95+ for upper bound. We hybridised separate global and data-rich models to acquire unadjusted results, which we adjusted using CoDCorrect and compared to the reference life table to calculate final YLLs due to vascular intestinal disorders.

In GBD 2021, we updated the linear floor value in our CODEm models to allow the model to be influenced by lower data, which resulted from changes to COD data processing. These data processing changes include changing the method of estimating the non-zero floor and population size cutoffs for when to noise-reduce at the country versus region level, and are described in greater detail in the appendix section “GBD 2021 Causes of Death database”. Apart from these, no other substantive changes were made in GBD 2021 from the modelling strategy used in GBD 2019.

The following table has the full list of covariates used for vascular intestinal disorders.


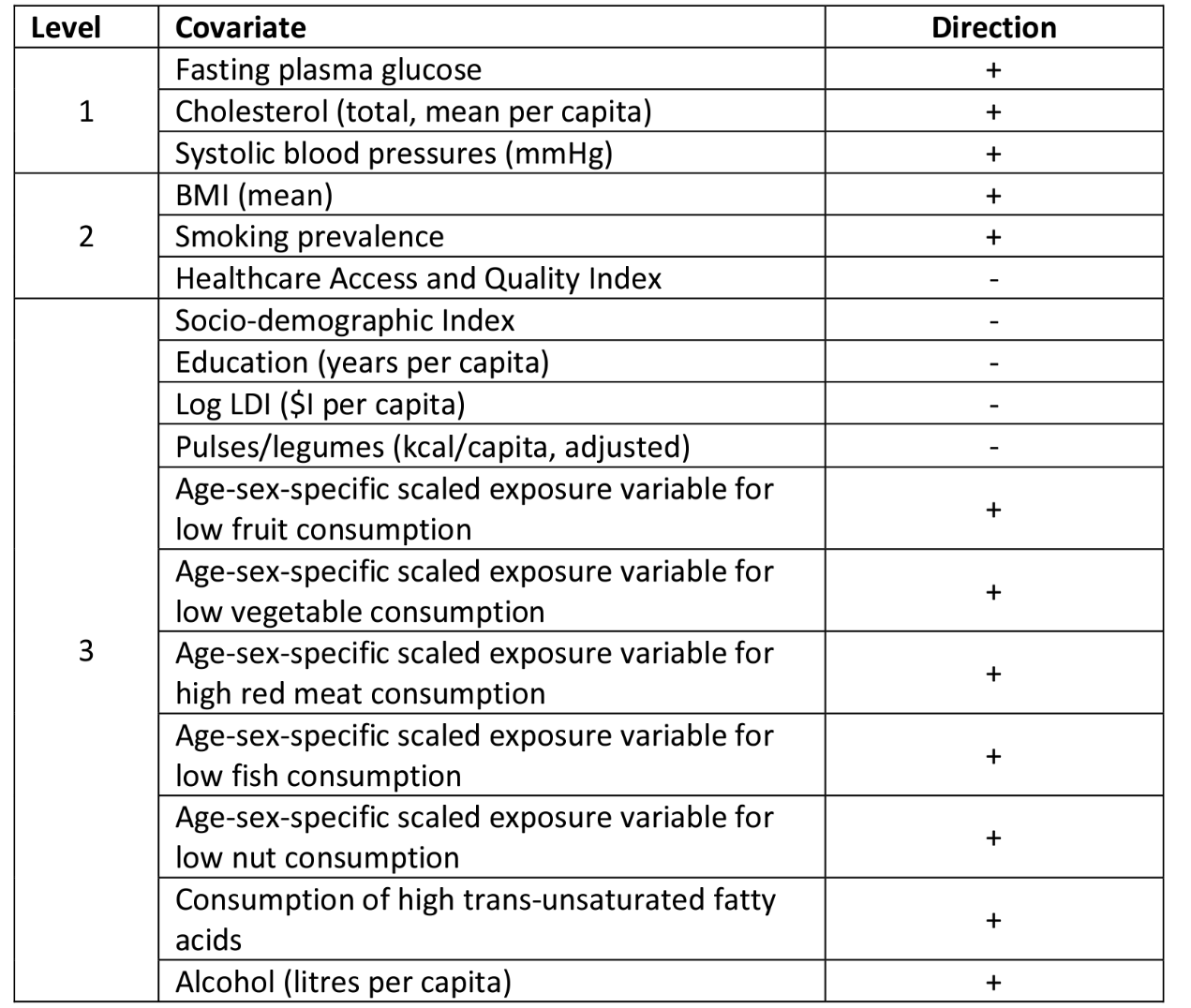


Adjustment in CoDCorrect included fitting unadjusted death estimates for all other specific and nonspecific digestive diseases to overall digestive disease deaths, which was then adjusted with all other causes to sum to all-cause counts of death.
