# Supplementary figures and images for: The global burden of vascular intestinal diseases: results from the 2021 Global Burden of Disease Study and projections using Bayesian age-period-cohort analysis
Source: Environ Health Prev Med. 2024 Dec 11;29:71. doi: 10.1265/ehpm.24-00206 (PMC11653002; doi:10.1265/ehpm.24-00206)

a

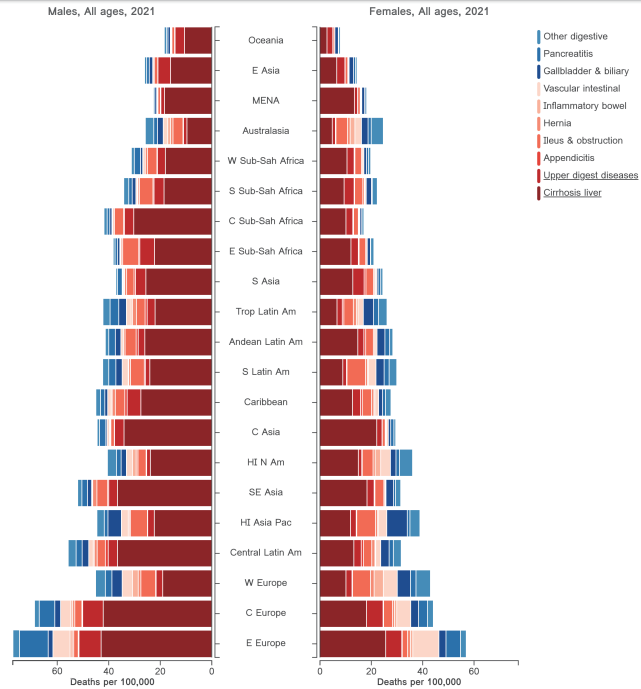

b

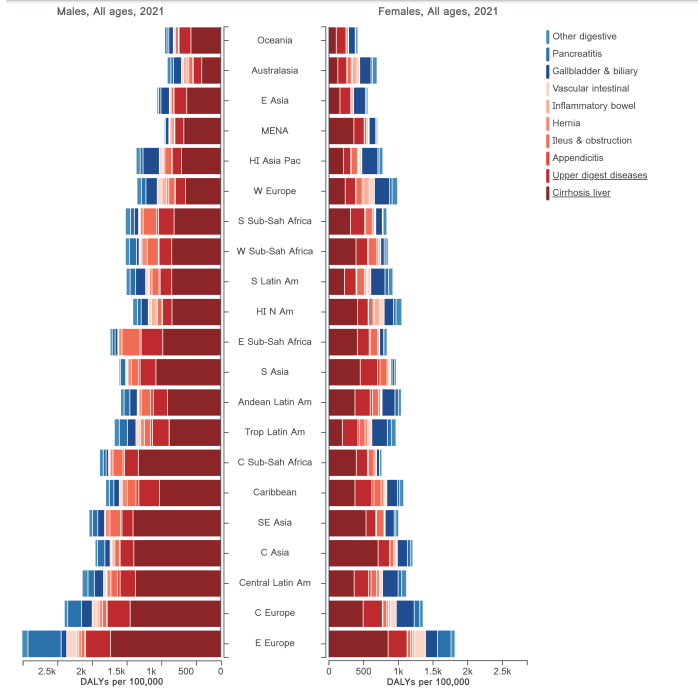

c

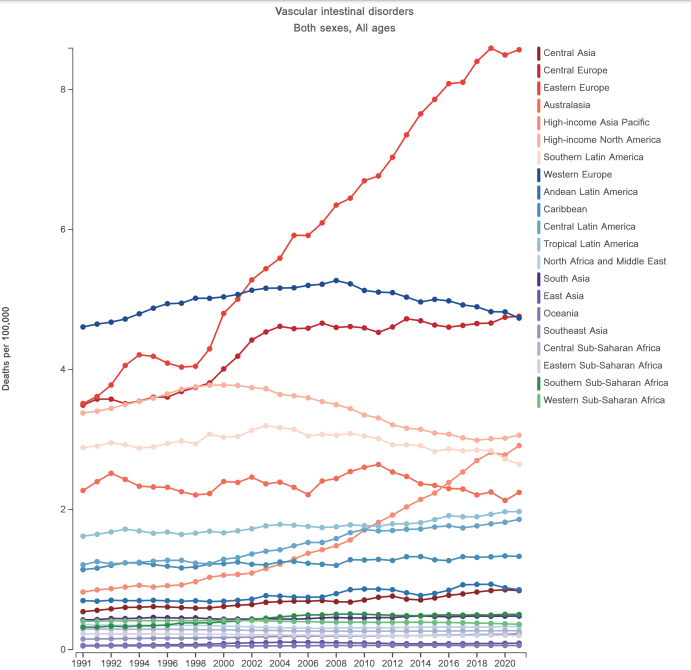

d

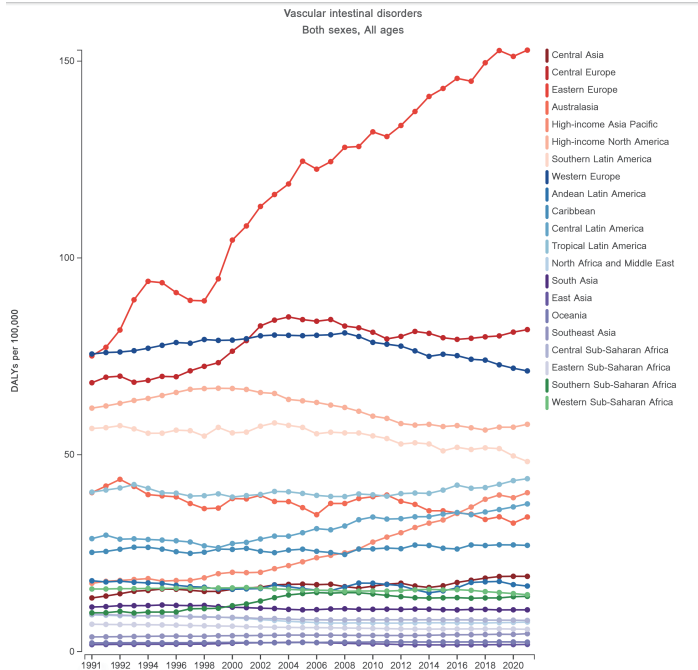

Supplement: Supplementary file 4 — Additional file 4: Figure S1 Proportion of disease burden for various digestive diseases across different regions in 2021 and changes in the disease burden of vascular intestinal diseases in different regions from 1990 to 2021. (a, c) Age-standardized mortality rate; (b, d) Age-standardized DALYs rate. [file ehpm-29-071-s004.pdf]

a

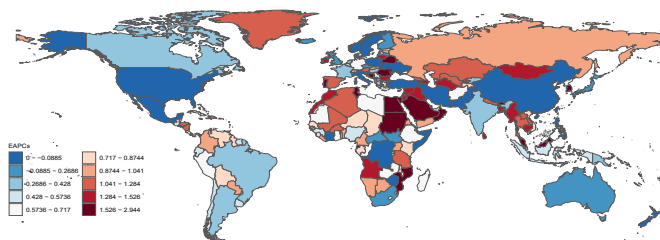

b

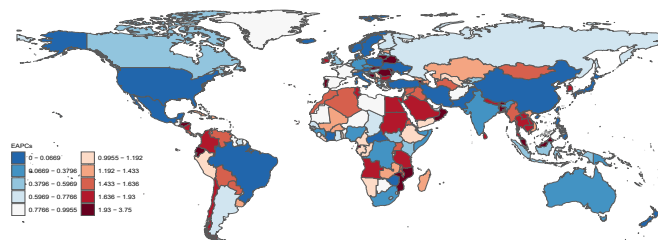

c

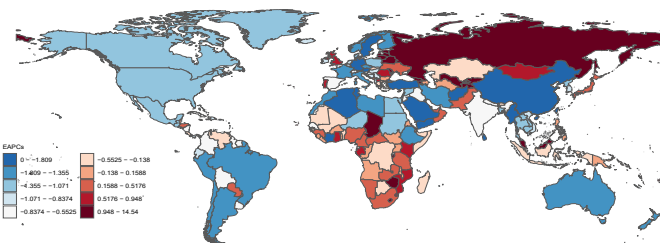

d

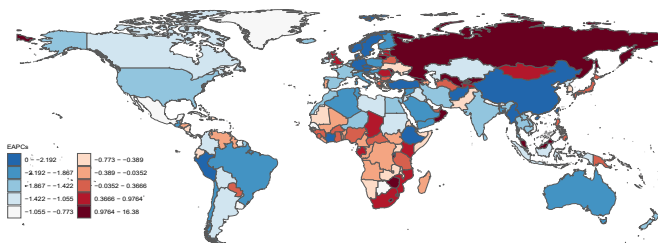

Supplement: Supplementary file 5 — Additional file 5: Figure S2 The estimated annual percentage change of the age-standardized rate of incidence (a), prevalence (b), mortality (c), and DALYs (d) of vascular intestinal diseases from 1990 to 2021. [file ehpm-29-071-s005.pdf]

# Global

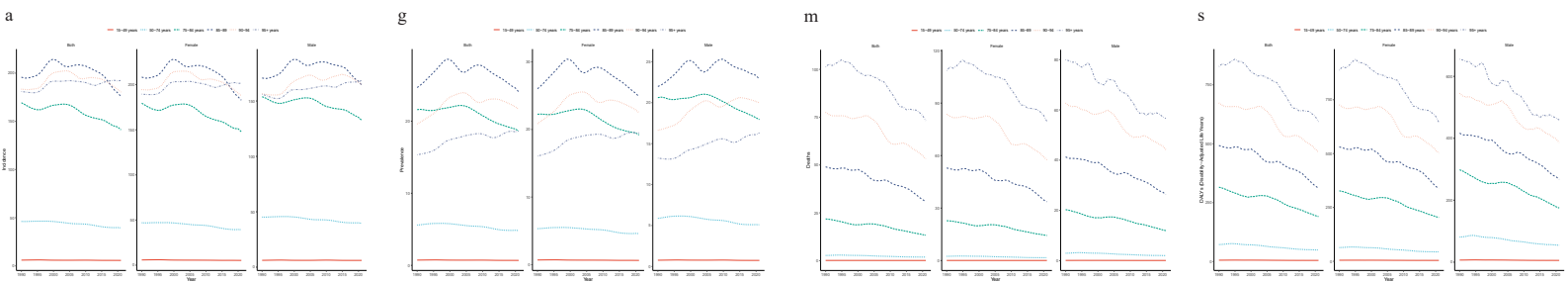

# Low SDI

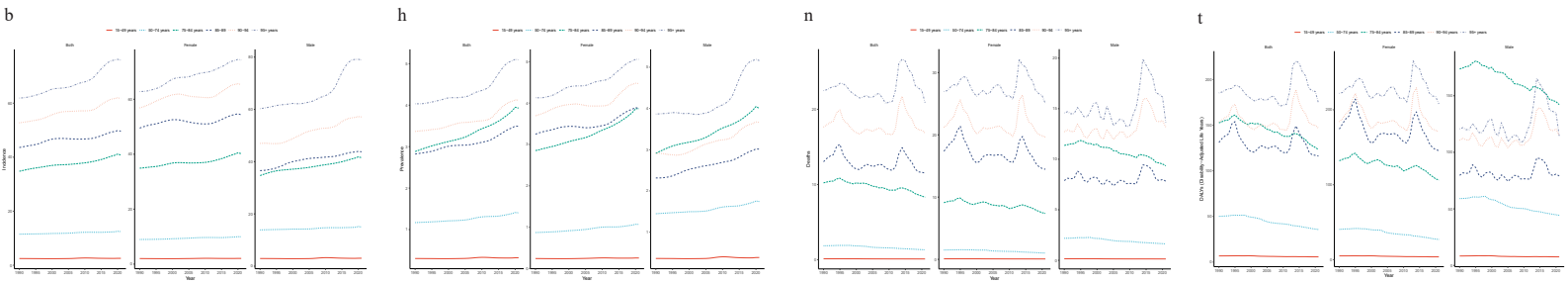

# Low-middle SDI

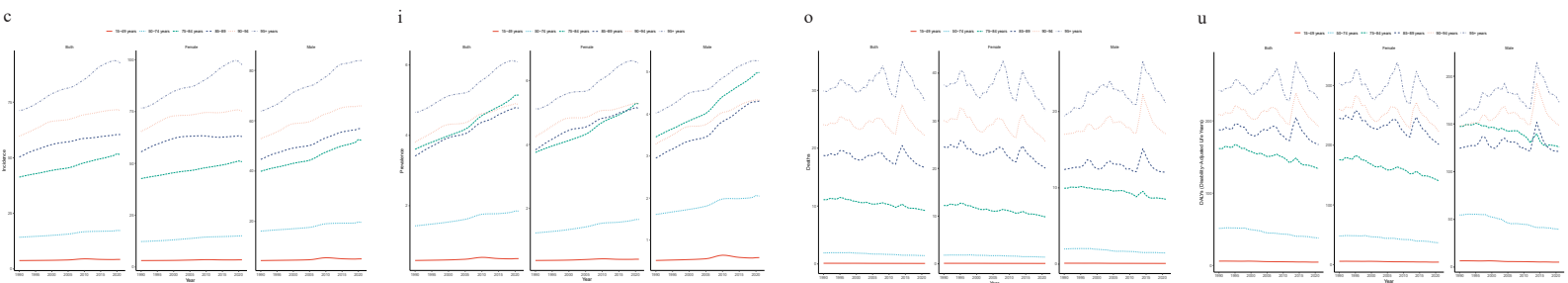

# Middle SDI

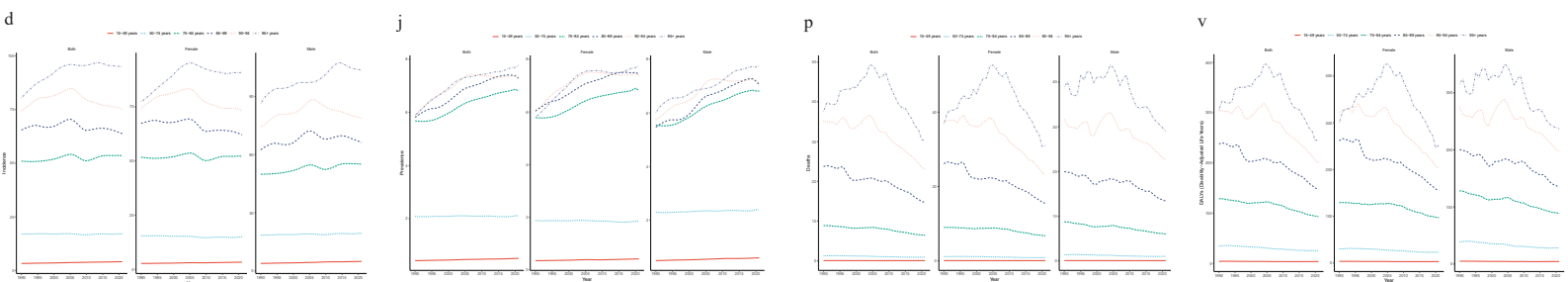

# High-middle SDI

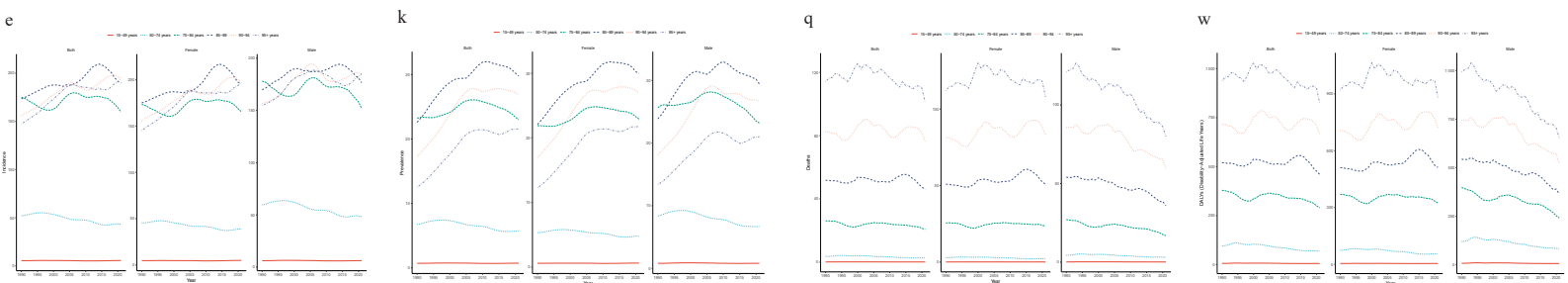

# High SDI

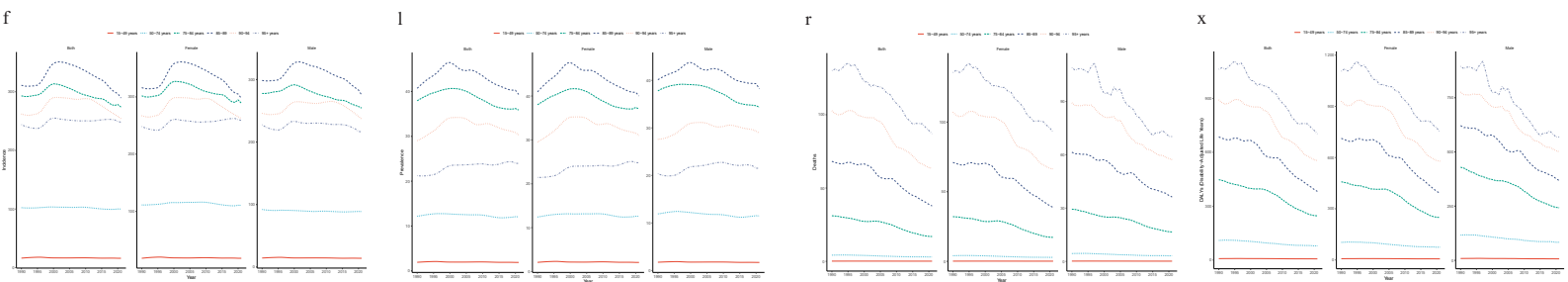

Supplement: Supplementary file 6 — Additional file 6: Figure S3 Temporal trends in the burden of vascular intestinal diseases by age globally and in SDI regions. (a–f) Age-standardized incidence rate; (g–l) Age-standardized prevalence rate; (m–r) Age-standardized mortality rate; (s–x) Age-standardized DALYs rate. [file ehpm-29-071-s006.pdf]

a

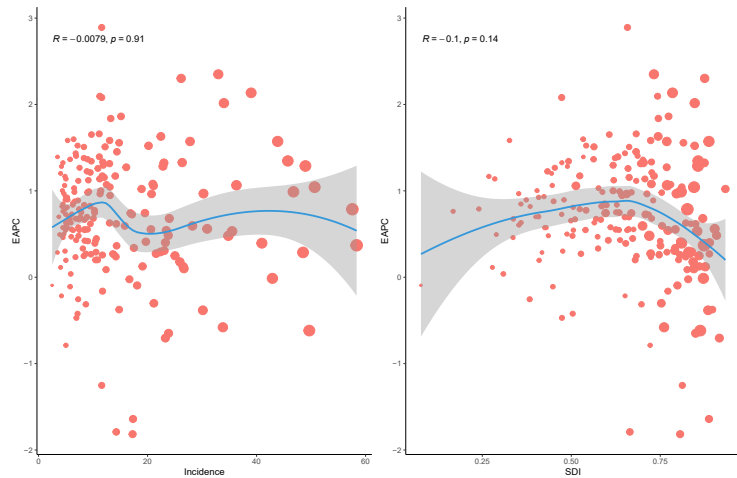

b

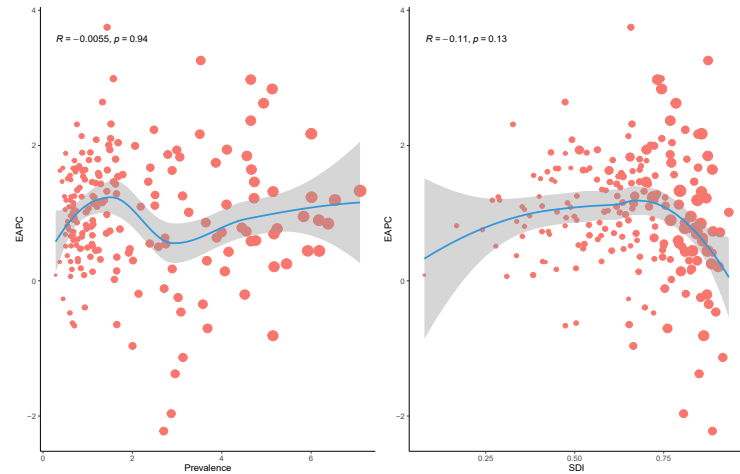

c

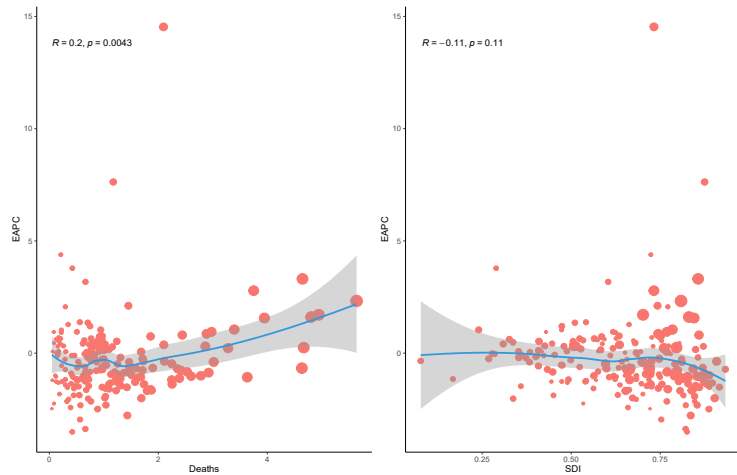

d

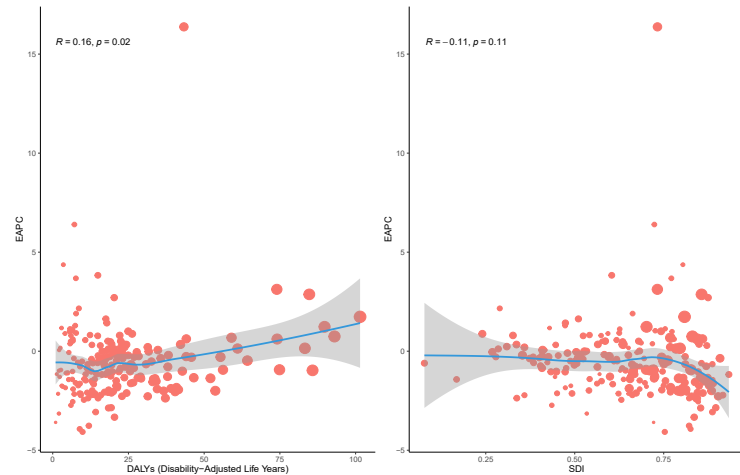

Supplement: Supplementary file 7 — Additional file 7: Figure S4 Correlation between Estimated Annual Percentage Change (EAPC) in 2021 and age-standardized rates of vascular intestinal diseases and SDI. (a) EAPC is not significantly correlated with ASIR/SDI; (b) EAPC is not significantly correlated with ASPR/SDI; (c) EAPC is positively correlated with ASMR but not significantly correlated with SDI; (d) EAPC is positively correlated with ASDR but not significantly correlated with SDI. In the left figures, circles represent countries, while in the right figures, circles represent countries for which Human Development Index data is available. The size of each circle is proportional to the number of (a) incident cases, (b) prevalent cases, (c) deaths, and (d) DALYs. The ρ values and p-values were obtained from Pearson correlation analysis. [file ehpm-29-071-s007.pdf]

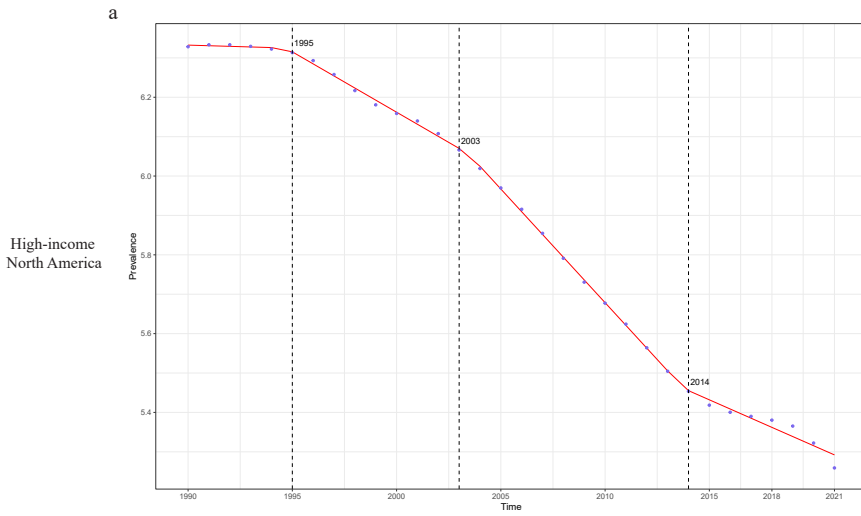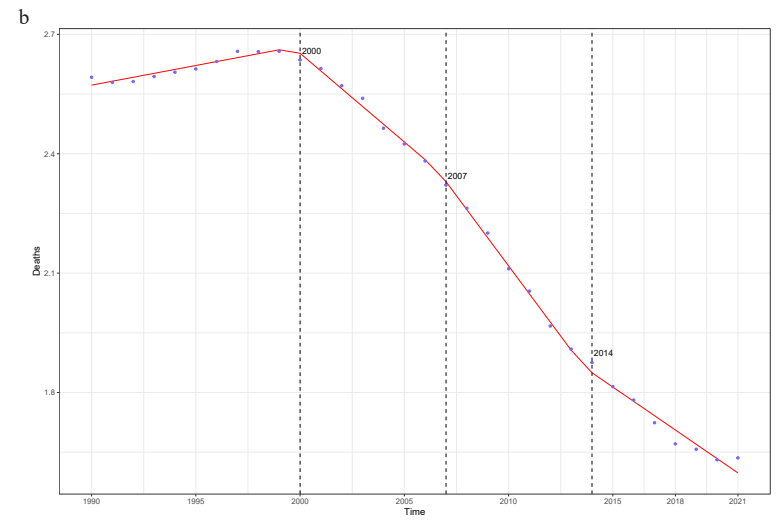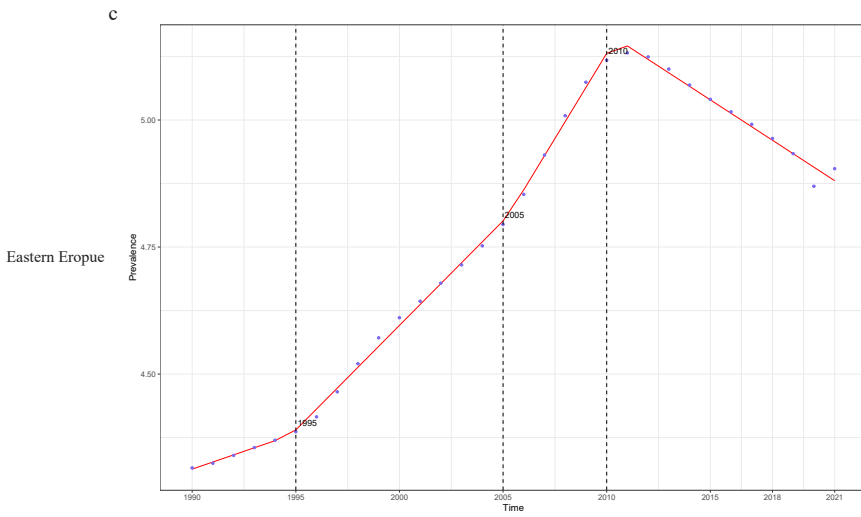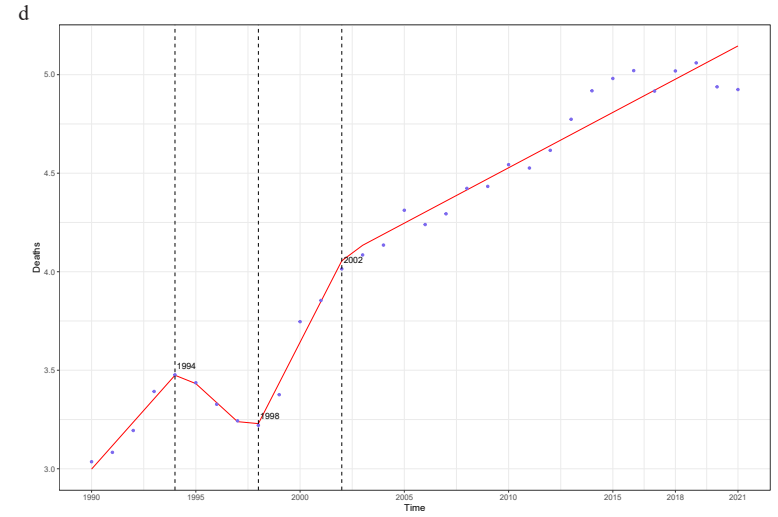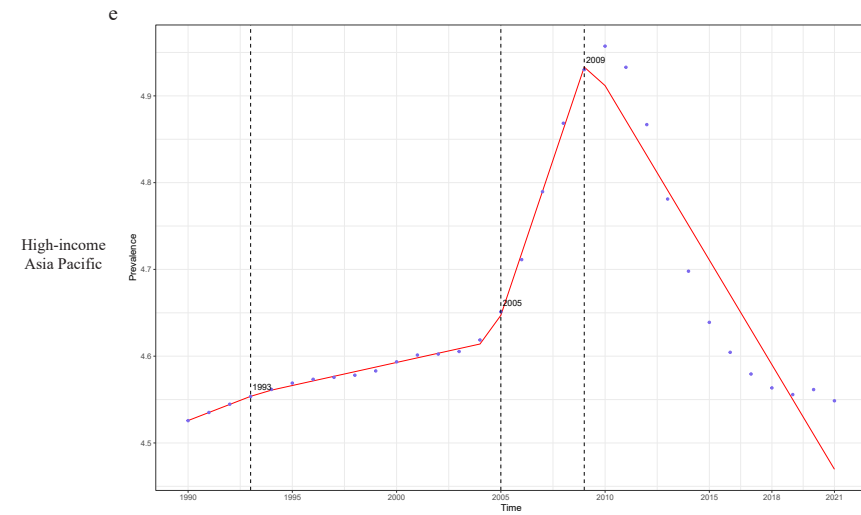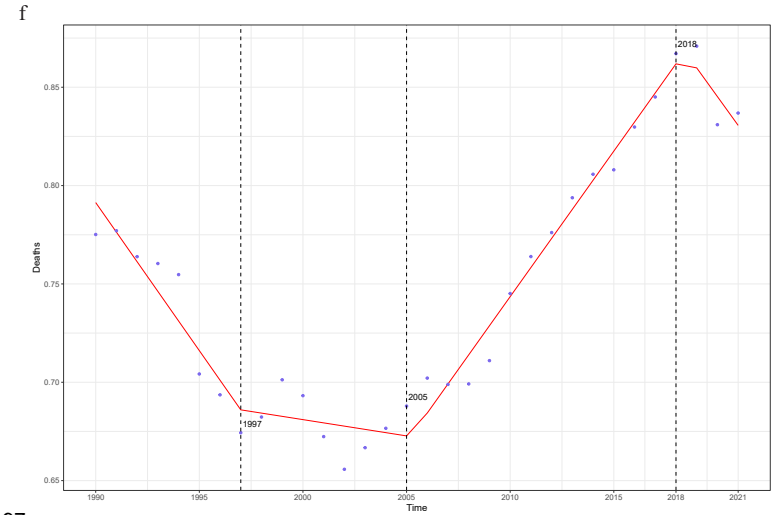

Supplement: Supplementary file 8 — Additional file 8: Figure S5 Join-point Regression Analysis of temporal trends in the burden of vascular intestinal diseases in the Top 3 regions with the highest prevalence from 1990 to 2021. (a, c, e) Age-standardized prevalence rate; (b, d, f) Age-standardized mortality rate. [file ehpm-29-071-s008.pdf]

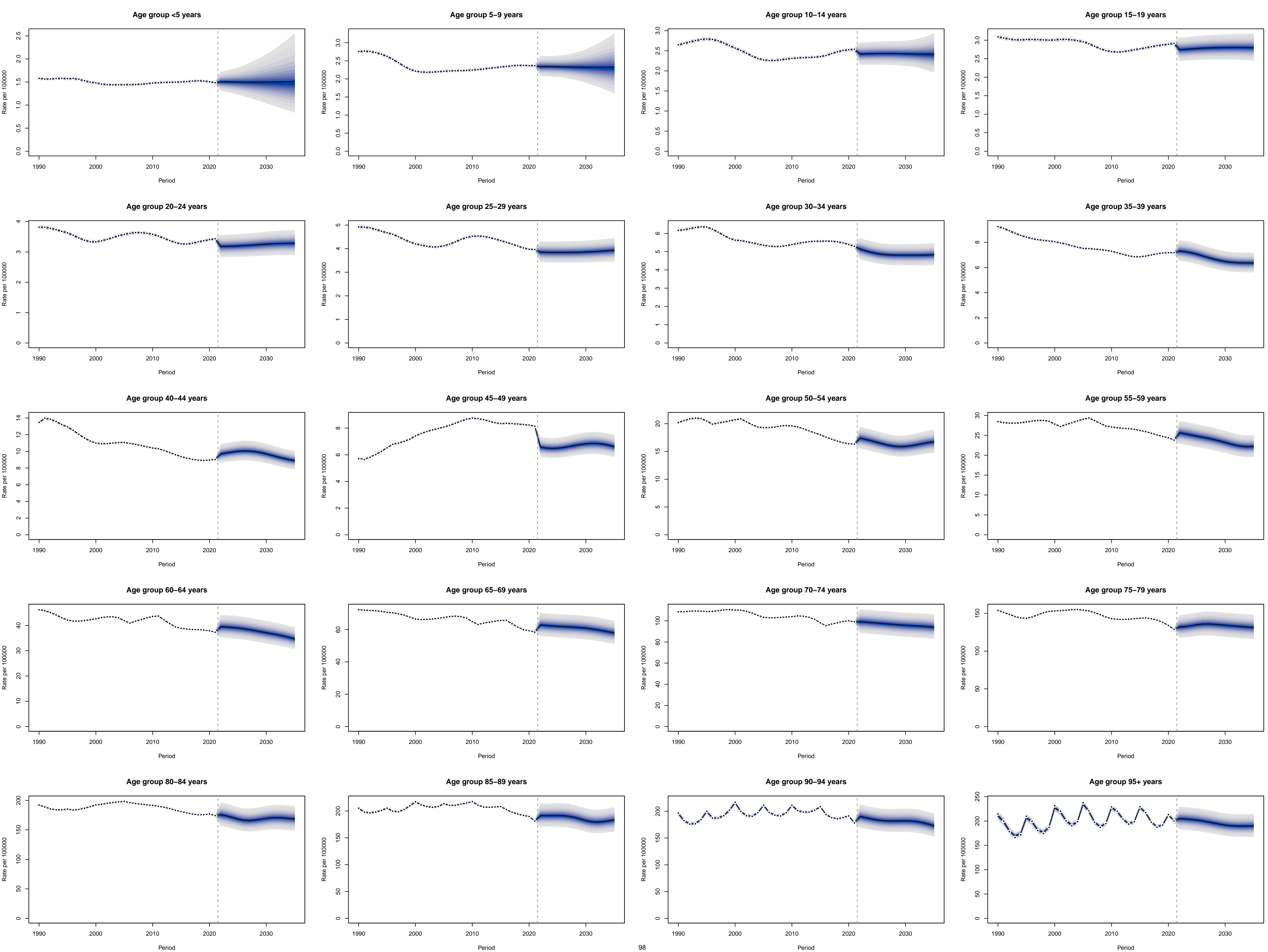

Supplement: Supplementary file 9 — Additional file 9: Figure S6 Trends of age-standardized incidence rate of vascular intestinal diseases across age groups: observed rates (1990–2021) and predicted rates (2022–2035). The blue region in shows the upper and lower limits of the 95% UI. [file ehpm-29-071-s009.pdf]

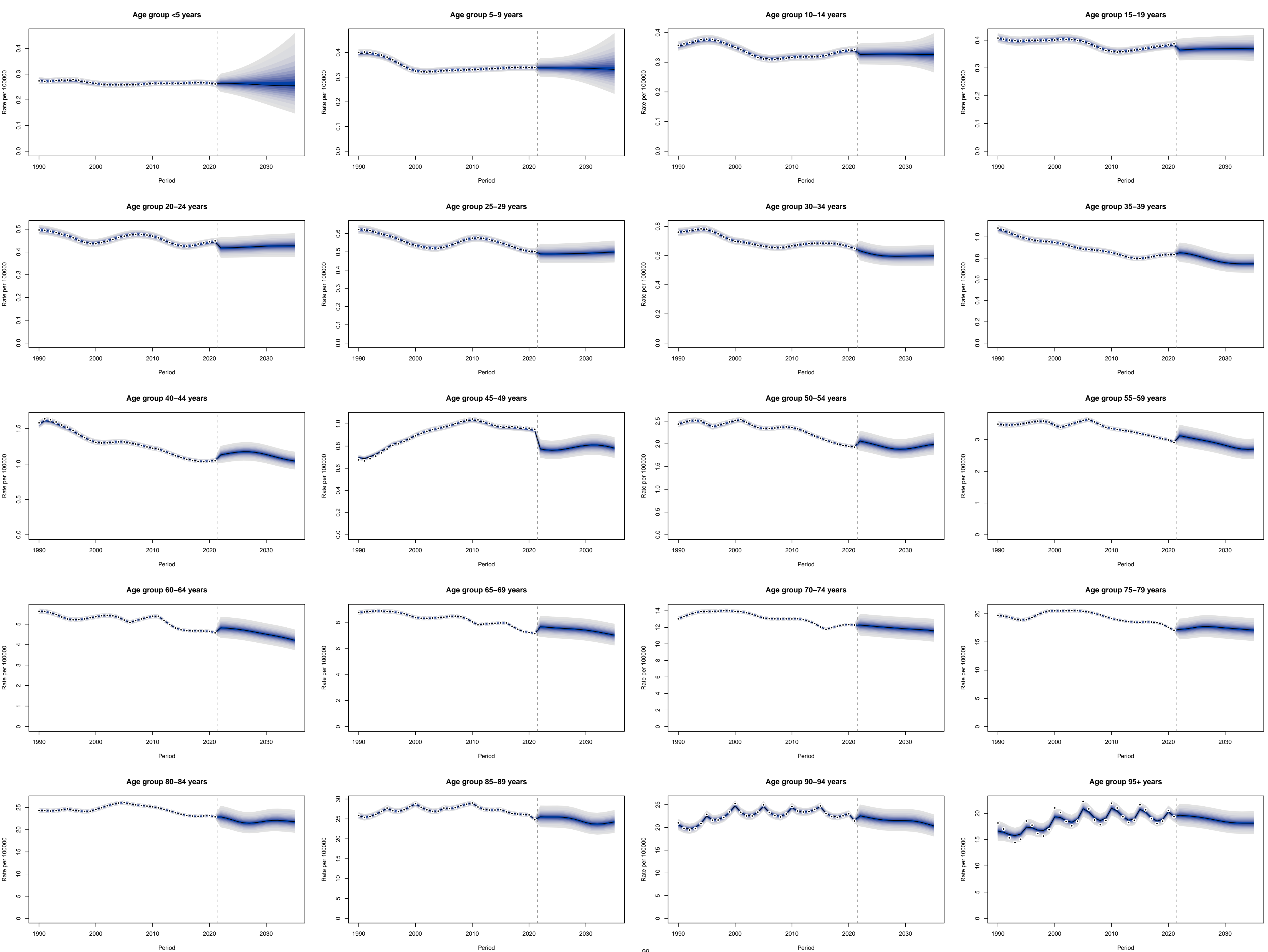

Supplement: Supplementary file 10 — Additional file 10: Figure S7 Trends of age-standardized prevalence rate of vascular intestinal diseases across age groups: observed rates (1990–2021) and predicted rates (2022–2035). The blue region in shows the upper and lower limits of the 95% UI. [file ehpm-29-071-s010.pdf]

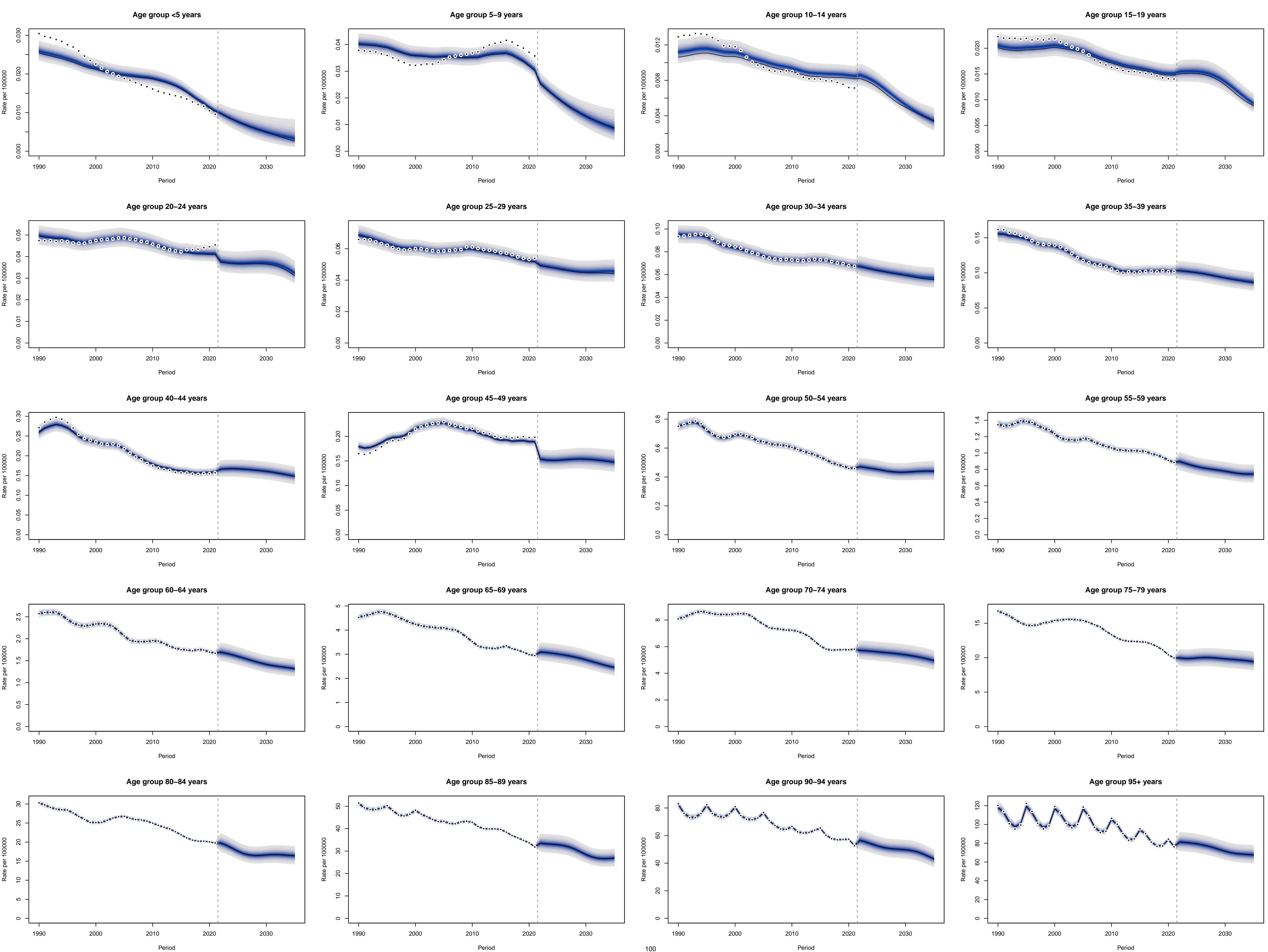

Supplement: Supplementary file 11 — Additional file 11: Figure S8 Trends of age-standardized mortality rate of vascular intestinal diseases across age groups: observed rates (1990–2021) and predicted rates (2022–2035). The blue region in shows the upper and lower limits of the 95% UI. [file ehpm-29-071-s011.pdf]

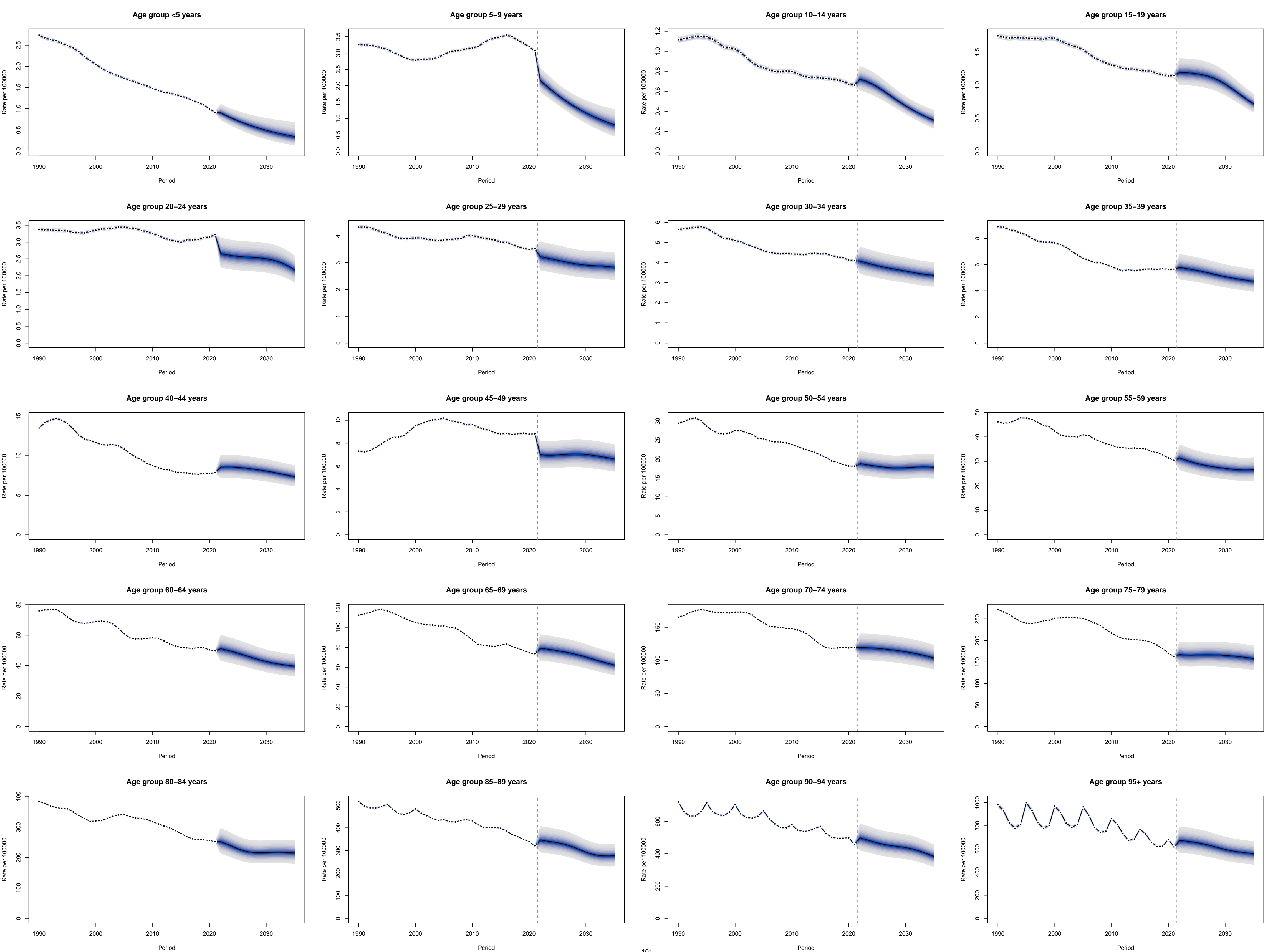

Supplement: Supplementary file 12 — Additional file 12: Figure S9 Trends of age-standardized DALYs rate of vascular intestinal diseases across age groups: observed rates (1990–2021) and predicted rates (2022–2035). The blue region in shows the upper and lower limits of the 95% UI. [file ehpm-29-071-s012.pdf]
