# Supplementary material for: The global burden of vascular intestinal diseases: results from the 2021 Global Burden of Disease Study and projections using Bayesian age-period-cohort analysis
Source: Environ Health Prev Med. 2024 Dec 11;29:71. doi: 10.1265/ehpm.24-00206 (PMC11653002; doi:10.1265/ehpm.24-00206)
Supplement: Supplementary file 13 — Additional file 13: Table S1 The number of incident cases, prevalent cases, deaths, and DALYs of vascular intestinal diseases in 1990, along with corresponding ASR. [file ehpm-29-071-s013.docx]

| **Table S1 The number of incident cases, prevalent cases, deaths, and DALYs of vascular intestinal diseases in 1990, along with corresponding ASR.** | | | | | | | | | | | | | | |  |
| --- | --- | --- | --- | --- | --- | --- | --- | --- | --- | --- | --- | --- | --- | --- | --- |
|  | **Incidence (95% uncertainty interval)** | | |  | **Prevalence (95% uncertainty interval)** | | |  | **Deaths (95% uncertainty interval)** | | |  | **DALYs (95% uncertainty interval)** | |  |
|  |  |  |  |  |  |  |  |  |  |  |  |  |  |  |  |
|  |  |  |  |  |  |  |  |  |  |  |  |  |  |  |  |
|  |  |  |  |  |  |  |  |  |  |  |  |  |  |  |  |
| **Characteristics** | **Cases,1990** | **ASIR,1990** |  |  | **Cases,1990** | **ASPR,1990** |  |  | **Cases,1990** | **ASMR,1990** |  |  | **Cases,1990** | **ASDR,1990** |  |
| Global | 757506.92 (647682.23-879672.23) | 18.81 (16.07-21.73) |  |  | 94057.38 (84225.47-105988.10) | 2.32 (2.108-2.58) |  |  | 57131.52 (52755.10-62009.66) | 1.75 (1.60-1.90) |  |  | 1168837.17 (1083807.43-1283676.05) | 31.21 (28.71-34.12) |  |
| Sex |  |  |  |  |  |  |  |  |  |  |  |  |  |  |  |
| Male | 345315.99 (295403.06-403167.67) | 18.33 (15.63-21.16) |  |  | 45070.01 (40089.85-50994.12) | 2.38 (2.16-2.63) |  |  | 24128.99 (22457.85-27257.53) | 1.69 (1.56-1.89) |  |  | 567636.34 (525516.18-646790.57) | 32.53 (30.22-36.77) |  |
| Female | 412190.92 (351128.98-478098.13) | 19.00 (16.22-21.97) |  |  | 48987.37 (44122.35-54906.22) | 2.25 (2.04-2.51) |  |  | 33002.53 (29807.71-35959.17) | 1.74 (1.56-1.90) |  |  | 601200.83 (539144.10-656120.96) | 29.28 (26.27-31.98) |  |
| SDI |  |  |  |  |  |  |  |  |  |  |  |  |  |  |  |
| High-middleSDI | 194578.86 (164310.73-226736.84) | 19.74 (16.70-22.84) |  |  | 25348.93 (22997.36-28024.23) | 2.57 (2.34-2.82) |  |  | 17347.90 (16262.05-18776.81) | 2.07 (1.918-2.24) |  |  | 350897.94 (330515.30-383620.38) | 37.50 (35.18-40.87) |  |
| LowSDI | 15249.77 (12595.13-18542.31) | 5.281 (4.49-6.19) |  |  | 1864.70 (1522.75-2352.36) | 0.55 (0.47-0.66) |  |  | 1765.97 (1304.86-2336.71) | 0.87 (0.62-1.16) |  |  | 57039.70 (43561.14-73423.07) | 20.18 (14.91-26.61) |  |
| HighSDI | 405359.36 (344942.44-469845.17) | 37.66 (32.22-43.70) |  |  | 49584.24 (44956.82-54904.32) | 4.60 (4.15-5.13) |  |  | 26815.35 (24260.37-28064.93) | 2.41 (2.173-2.53) |  |  | 469951.30 (439634.77-487348.256) | 42.46 (39.70-44.08) |  |
| Low-middleSDI | 52200.43 (42458.41-63669.44) | 6.83 (5.72-8.05) |  |  | 5902.33 (4810.68-7362.48) | 0.71 (0.60-0.85) |  |  | 4864.63 (3502.88-6821.21) | 0.95 (0.68-1.33) |  |  | 131991.73 (98428.85-183260.28) | 20.24 (14.80-28.27) |  |
| MiddleSDI | 89526.46 (72432.40-108981.19) | 7.56 (6.34-8.84) |  |  | 11266.73 (9344.72-13676.80) | 0.91 (0.78-1.06) |  |  | 6237.13 (5674.21-6882.54) | 0.81 (0.72-0.89) |  |  | 156983.21 (144996.28-173016.45) | 15.45 (14.13-17.01) |  |
| Region |  |  |  |  |  |  |  |  |  |  |  |  |  |  |  |
| EastAsia | 55778.74 (42972.84-69912.31) | 6.04 (4.79-7.37) |  |  | 6490.87 (5151.58-8119.37) | 0.69 (0.56-0.83) |  |  | 696.21 (520.01-822.01) | 0.12 (0.08-0.14) |  |  | 21292.33 (16426.91-25292.01) | 2.51 (1.90-2.96) |  |
| Oceania | 167.41 (131.99-208.36) | 3.92 (3.19-4.73) |  |  | 20.23 (15.70-26.59) | 0.43 (0.35-0.52) |  |  | 3.34 (2.34-4.44) | 0.10 (0.07-0.14) |  |  | 143.58 (102.27-194.76) | 3.26 (2.34-4.27) |  |
| SoutheastAsia | 14127.90 (11374.66-17231.27) | 4.83 (3.98-5.70) |  |  | 1585.81 (1281.76-1961.18) | 0.51 (0.43-0.60) |  |  | 694.25 (512.38-948.11) | 0.37 (0.26-0.52) |  |  | 17273.32 (13481.28-22501.40) | 6.76 (5.10-9.05) |  |
| CentralAsia | 6812.42 (5584.7-8334.76) | 11.68 (9.68-13.80) |  |  | 800.46 (660.74-1002.37) | 1.33 (1.13-1.61) |  |  | 374.91 (339.36-430.48) | 0.86 (0.78-1.00) |  |  | 9438.94 (8674.57-10486.07) | 19.39 (17.706-21.84) |  |
| EasternEurope | 101407.90 (86194.29-117175.261) | 37.81 (32.30-43.53) |  |  | 11590.01 (10618.43-12594.73) | 4.32 (3.93-4.72) |  |  | 7959.229 (7387.47-9028.23) | 3.04 (2.81-3.43) |  |  | 170118.14 (157214.44-198259.70) | 62.16 (57.27-72.25) |  |
| CentralEurope | 20738.86 (17561.17-24475.58) | 15.16 (12.81-17.94) |  |  | 3270.20 (2916.18-3660.43) | 2.34 (2.06-2.64) |  |  | 4366.68 (4154.40-4574.48) | 3.23 (3.06-3.39) |  |  | 85426.87 (81920.27-89413.77) | 59.43 (56.79-62.25) |  |
| WesternEurope | 137622.71 (111219.17-165281.39) | 24.50 (20.10-29.37) |  |  | 22888.25 (21189.31-24772.57) | 3.98 (3.65-4.35) |  |  | 17712.47 (16037.30-18681.15) | 2.93 (2.64-3.10) |  |  | 290612.00 (269854.05-303453.93) | 49.33 (45.94-51.43) |  |
| Australasia | 6479.06 (5400.54-7804.62) | 27.47 (23.03-32.88) |  |  | 813.23 (739.86-892.14) | 3.45 (3.12-3.82) |  |  | 460.45 (417.27-491.96) | 2.02 (1.83-2.17) |  |  | 8189.32 (7605.83-8653.10) | 35.03 (32.46-37.05) |  |
| High-incomeAsiaPacific | 81955.26 (67404.46-100369.78) | 41.47 (34.34-50.35) |  |  | 8893.71 (7509.90-10682.09) | 4.53 (3.83-5.42) |  |  | 1425.12 (1305.91-1499.18) | 0.78 (0.70-0.82) |  |  | 30073.70 (28300.62-31735.66) | 15.51 (14.54-16.35) |  |
| CentralLatinAmerica | 18496.90 (15165.84-21990.29) | 18.54 (15.59-21.55) |  |  | 2218.29 (1885.44-2701.77) | 2.08 (1.84-2.36) |  |  | 1988.75 (1899.69-2056.30) | 2.75 (2.60-2.85) |  |  | 47170.59 (45596.92-48585.06) | 53.48 (51.44-55.16) |  |
| SouthernLatinAmerica | 9615.76 (7893.28-11135.42) | 20.75 (17.10-23.960) |  |  | 1130.00 (1022.89-1250.02) | 2.42 (2.19-2.67) |  |  | 1428.48 (1322.40-1524.30) | 3.34 (3.07-3.56) |  |  | 28081.72 (26475.60-29808.43) | 62.02 (58.31-65.81) |  |
| AndeanLatinAmerica | 1667.90 (1388.88-1972.54) | 6.62 (5.66-7.68) |  |  | 227.00 (184.58-291.63) | 0.77 (0.65-0.92) |  |  | 265.56 (219.75-307.87) | 1.40 (1.15-1.61) |  |  | 6829.89 (5591.51-7979.16) | 28.90 (23.99-33.63) |  |
| High-incomeNorthAmerica | 203502.75 (175561.90-232983.28) | 58.45 (50.76-67.04) |  |  | 21858.93 (20095.76-23750.16) | 6.33 (5.79-6.96) |  |  | 9509.35 (8518.47-10086.07) | 2.59 (2.33-2.75) |  |  | 174022.69 (163332.77-181868.44) | 49.26 (46.33-51.37) |  |
| Caribbean | 2979.29 (2491.13-3575.56) | 10.50 (8.84-12.47) |  |  | 413.77 (362.32-483.09) | 1.44 (1.29-1.65) |  |  | 402.90 (370.40-437.27) | 1.67 (1.54-1.81) |  |  | 8890.67 (8172.91-9702.04) | 33.81 (31.22-36.81) |  |
| SouthAsia | 54279.86 (43992.90-67091.12) | 7.45 (6.21-8.83) |  |  | 5773.55 (4671.58-7320.84) | 0.73 (0.61-0.87) |  |  | 4620.59 (2839.44-7110.78) | 0.99 (0.58-1.53) |  |  | 123603.55 (79559.09-189881.95) | 20.75 (13.01-31.82) |  |
| NorthAfricaandMiddleEast | 14093.55 (11497.10-16999.78) | 6.93 (5.73-8.20) |  |  | 1588.54 (1287.01-1987.56) | 0.73 (0.61-0.87) |  |  | 1212.34 (865.51-1527.31) | 0.860 (0.61-1.10) |  |  | 31633.43 (22920.08-38954.52) | 17.88 (12.89-22.32) |  |
| SouthernSub-SaharanAfrica | 4086.49 (3255.40-5282.03) | 9.51 (7.80-11.57) |  |  | 469.91 (372.39-609.74) | 1.03 (0.84-1.28) |  |  | 167.42 (119.03-207.51) | 0.658 (0.44-0.84) |  |  | 5155.24 (4084.80-6123.34) | 15.838 (11.88-19.14) |  |
| EasternSub-SaharanAfrica | 3106.77 (2633.14-3707.60) | 4.03 (3.39-4.76) |  |  | 385.73 (322.04-464.04) | 0.44 (0.38-0.50) |  |  | 427.38 (291.00-557.77) | 0.67 (0.44-0.89) |  |  | 13248.54 (8859.89-17008.35) | 14.52 (9.93-18.91) |  |
| TropicalLatinAmerica | 11807.74 (10166.05-13830.734 | 11.32 (9.99-12.83) |  |  | 2359.34 (2092.45-2695.31) | 2.23 (2.02-2.46) |  |  | 2470.42 (2329.50-2581.43) | 3.12 (2.90-3.28) |  |  | 61781.75 (59351.04-64153.11) | 65.38 (62.24-68.11) |  |
| CentralSub-SaharanAfrica | 1862.13 (1559.95-2232.07) | 7.18 (6.11-8.44) |  |  | 239.47 (199.31-296.66) | 0.79 (0.69-0.90) |  |  | 161.29 (120.01-216.19) | 0.90 (0.69-1.18) |  |  | 5168.86 (3730.19-7394.38) | 19.52 (14.76-25.79) |  |
| WesternSub-SaharanAfrica | 6917.52 (5573.22-8618.86) | 5.26 (4.40-6.29) |  |  | 1040.09 (830.40-1343.76) | 0.65 (0.54-0.79) |  |  | 784.38 (614.24-978.54) | 0.80 (0.63-1.01) |  |  | 30682.04 (24083.91-37981.79) | 22.48 (17.54-28.11) |  |
|  |  |  |  | | | | | | | | | | | |  |

ASR, Age-standardized rates; ASIR,Age-standardized incidence rate; ASPR, Age-standardized prevalence rate; ASMR, Age-standardized mortality rate; ASDR, Age-standardized

disability-adjusted life years rate; DALYs, Disability-adjusted life years;SDI, Socio-demographic Index.
