# Supplementary material for: The global burden of vascular intestinal diseases: results from the 2021 Global Burden of Disease Study and projections using Bayesian age-period-cohort analysis
Source: Environ Health Prev Med. 2024 Dec 11;29:71. doi: 10.1265/ehpm.24-00206 (PMC11653002; doi:10.1265/ehpm.24-00206)
Supplement: Supplementary file 14 — Additional file 14: Table S2 The number of incident cases, prevalent cases, deaths, and DALYs of vascular intestinal diseases at the National Level in 2021, along with corresponding ASR and temporal trends. [file ehpm-29-071-s014.docx]

| **Table S2 The number of incident cases, prevalent cases, deaths, and DALYs of vascular intestinal diseases at the National Level in 2021, along with corresponding ASR and temporal trends.** | | | | | | | | | | | | | | | |
| --- | --- | --- | --- | --- | --- | --- | --- | --- | --- | --- | --- | --- | --- | --- | --- |
|  | **Incidence (95% uncertainty interval)** | | |  | **Prevalence (95% uncertainty interval)** | | |  | **Deaths (95% uncertainty interval)** | | |  | **DALYs (95% uncertainty interval)** | | |
|  |  |  |  |  |  |  |  |  |  |  |  |  |  |  |  |
|  |  |  |  |  |  |  |  |  |  |  |  |  |  |  |  |
|  |  |  |  |  |  |  |  |  |  |  |  |  |  |  |  |
| **Characteristics** | **Cases,2021** | **ASIR,2021** | **EAPC1990-2021** |  | **Cases,2021** | **ASPR,2021** | **EAPC1990-2021** |  | **Cases,2021** | **ASMR,2021** | **EAPC1990-2021** |  | **Cases,2021** | **ASDR,021** | **EAPC1990-2021** |
| Timor-Leste | 39.59  (32.49-47.01) | 4.05  (3.36-4.76) | 1.20  (1.05-1.36) |  | 4.42  (3.70-5.40) | 0.43  (0.37-0.50) | 1.47  (1.29-1.65) |  | 0.80  (0.30-1.51) | 0.12  (0.05-0.23) | -1.27  (-1.43--1.10) |  | 19.46  (8.73-34.71) | 2.31  (0.99-4.21) | -1.30  (-1.59--1.00) |
| Georgia | 689.13  (604.97-784.43) | 14.08  (12.27-16.40) | 1.17  (0.95-1.39) |  | 79.02  (71.50-87.94) | 1.64  (1.44-1.91) | 1.29  (1.08-1.50) |  | 129.64  (104.68-157.05) | 2.09  (1.69-2.54) | 14.53  (12.63-16.47) |  | 2503.08  (1983.01-3088.74) | 43.31  (34.39-53.71) | 16.37  (14.38-18.40) |
| Lao People's Democratic Republic | 220.59  (176.39-265.55) | 4.21  (3.46-4.94) | 1.28  (1.23-1.34) |  | 24.43  (19.96-30.05) | 0.44  (0.38-0.52) | 1.67  (1.60-1.74) |  | 5.35  (3.25-9.25) | 0.17  (0.10-0.29) | -1.31  (-1.41--1.22) |  | 132.93  (86.90-218.24) | 3.01  (1.89-5.03) | -1.75  (-1.84--1.65) |
| Maldives | 25.90  (20.99-31.54) | 6.81  (5.55-7.99) | 1.43  (1.31-1.56) |  | 3.23  (2.67-4.00) | 0.87  (0.75-1.00) | 2.14  (1.98-2.30) |  | 0.27  (0.18-0.37) | 0.11  (0.07-0.15) | -1.00  (-1.15--0.84) |  | 5.32  (3.84-6.78) | 1.78  (1.26-2.30) | -2.15  (-2.29--2.01) |
| Solomon Islands | 20.46  (16.49-24.90) | 4.47  (3.68-5.26) | 0.57  (0.51-0.64) |  | 2.46  (1.98-3.13) | 0.50  (0.42-0.60) | 0.69  (0.62-0.75) |  | 0.34  (0.20-0.51) | 0.08  (0.05-0.12) | 0.94  (0.86-1.02) |  | 15.65  (9.23-23.24) | 2.91  (1.75-4.28) | 1.17  (1.03-1.31) |
| Bosnia and Herzegovina | 613.46  (516.63-726.05) | 13.05 (10.93-15.52) | 1.58 (1.34-1.83) |  | 103.43 (92.62-114.58) | 2.06 (1.80-2.37) | 2.00 (1.66-2.34) |  | 82.55  (57.57-109.44) | 1.28 (0.89-1.69) | -0.95  (-1.11--0.78) |  | 1413.24 (1006.25-1885.62) | 22.39 (16.07-29.87) | -0.86 (-1.03--0.69) |
| Kyrgyzstan | 631.43  (507.38-777.07) | 10.49 (8.55-12.50) | 0.43 (0.33-0.53) |  | 72.74  (58.33-93.19) | 1.18 (0.98-1.47) | 0.45 (0.35-0.54) |  | 27.87  (21.61-34.23) | 0.66 (0.51-0.81) | 3.18  (2.66-3.69) |  | 745.31 (586.16-916.23) | 14.91 (11.69-18.30) | 3.83 (3.24-4.42) |
| Democratic People's Republic of Korea | 2159.14 (1732.80-2618.49) | 6.85 (5.57-8.15) | 0.68 (0.59-0.77) |  | 235.95  (197.50-280.67) | 0.76 (0.64-0.90) | 0.86 (0.76-0.97) |  | 15.52  (9.99-34.99) | 0.05 (0.04-0.12) | -1.20  (-1.27--1.14) |  | 406.75 (285.95-783.93) | 1.34 (0.94-2.61) | -1.16 (-1.23--1.09) |
| Vanuatu | 10.68  (8.81-13.00) | 5.00 (4.26-5.91) | 0.73 (0.69-0.76) |  | 1.23  (1.02-1.53) | 0.54 (0.46-0.63) | 0.81 (0.78-0.84) |  | 0.43  (0.27-0.71) | 0.22 (0.14-0.36) | 1.03 (0.94-1.13) |  | 18.05  (11.12-29.30) | 7.43 (4.65-12.03) | 1.29 (1.12-1.47) |
| Tajikistan | 827.72  (666.44-1032.32) | 10.27 (8.50-12.11) | 0.77 (0.63-0.92) |  | 94.02 (75.55-121.80) | 1.11 (0.92-1.36) | 0.67 (0.52-0.83) |  | 26.43 (16.30-38.38) | 0.56 (0.34-0.80) | 1.38 (1.12-1.64) |  | 779.67 (495.50-1153.32) | 12.32 (7.74-17.92) | 0.82 (0.49-1.15) |
| Croatia | 1398.27 (1216.12-1637.76) | 18.09 (15.73-21.09) | -0.10 (-0.38-0.19) |  | 346.89 (321.24-371.52) | 4.07 (3.73-4.43) | 0.14 (0.08-0.20) |  | 242.37 (214.42-268.03) | 2.42 (2.14-2.67) | -0.77  (-1.10--0.44) |  | 3806.71 (3386.76-4210.88) | 40.42 (36.05-44.73) | -2.05 (-2.24--1.86) |
| Kiribati | 7.30  (6.12-8.62) | 8.67 (7.36-10.05) | 0.27 (0.23-0.32) |  | 1.01  (0.90-1.16) | 1.09 (1.00-1.22) | 0.54 (0.49-0.60) |  | 0.82 (0.59-1.14) | 1.10 (0.78-1.56) | 0.19 (0.12-0.26) |  | 29.40  (21.00-41.70) | 31.68 (23.10-44.31) | -0.02 (-0.11-0.08) |
| Philippines | 3985.64 (3203.89-4916.98) | 4.21 (3.46-5.06) | -0.22 (-0.27--0.16) |  | 439.03 (353.09-549.64) | 0.45 (0.37-0.54) | -0.27 (-0.34--0.20) |  | 79.40 (59.69-94.16) | 0.12 (0.09-0.15) | 0.02  (-0.13-0.17) |  | 2048.43 (1704.86-2385.42) | 2.51 (2.01-2.94) | 0.17 (0.03-0.30) |
| Micronesia (Federated States of) | 5.29  (4.35-6.30) | 6.59 (5.49-7.67) | 0.99 (0.96-1.01) |  | 0.64  (0.54-0.75) | 0.77 (0.67-0.87) | 1.34 (1.30-1.37) |  | 0.15 (0.10-0.22) | 0.20 (0.13-0.28) | 0.42 (0.35-0.48) |  | 5.88  (3.82-8.71) | 6.69 (4.40-9.72) | 0.52 (0.42-0.62) |
| Uzbekistan | 3598.62 (2945.00-4409.36) | 12.05 (10.07-14.51) | 1.16 (1.08-1.23) |  | 405.53 (329.43-510.98) | 1.33 (1.11-1.64) | 1.15 (1.07-1.23) |  | 74.74 (61.11-89.77) | 0.34 (0.29-0.41) | 1.28 (0.85-1.70) |  | 2020.93 (1631.69-2443.70) | 7.71 (6.33-9.17) | 1.90 (1.31-2.49) |
| Indonesia | 13763.10 (11421.02-16040.18) | 6.34 (5.40-7.22) | 0.54 (0.48-0.61) |  | 1418.77 (1212.76-1691.42) | 0.63 (0.55-0.72) | 0.52 (0.44-0.60) |  | 380.39 (232.10-799.06) | 0.29 (0.17-0.62) | -0.28  (-0.39--0.16) |  | 7799.85 (5088.31-15114.69) | 4.43 (2.79-9.06) | -1.10 (-1.18--1.02) |
| Azerbaijan | 1316.01 (1086.08-1572.39) | 13.02 (10.75-15.62) | 1.31 (1.11-1.52) |  | 144.73 (120.66-176.10) | 1.43 (1.20-1.74) | 1.32 (1.13-1.52) |  | 26.08 (16.69-38.33) | 0.30 (0.19-0.45) | 0.06  (-0.06-0.18) |  | 694.84 (460.16-981.70) | 6.99 (4.73-9.91) | -0.37 (-0.55--0.19) |
| Thailand | 7277.36 (5865.01-8745.83) | 7.31 (5.97-8.65) | 1.12 (1.09-1.14) |  | 927.11 (818.69-1053.60) | 0.94 (0.83-1.09) | 1.64 (1.60-1.68) |  | 611.16 (443.38-811.00) | 0.58 (0.42-0.77) | -1.15  (-1.32--0.99) |  | 10908.37 (8142.16-14198.53) | 10.88 (8.20-14.09) | -1.48 (-1.68--1.27) |
| Kazakhstan | 3779.22 (3178.08-4476.30) | 20.97 (17.70-24.72) | 1.08 (0.95-1.21) |  | 454.02 (399.97-522.16) | 2.51 (2.22-2.86) | 1.27 (1.12-1.42) |  | 271.88 (217.74-344.00) | 1.72 (1.41-2.15) | -0.36  (-0.71--0.00) |  | 6770.63 (5369.53-8600.08) | 37.92 (30.27-47.85) | -1.28 (-1.77--0.80) |
| Samoa | 10.16  (8.42-12.09) | 6.33 (5.27-7.47) | 0.86 (0.81-0.90) |  | 1.23  (1.04-1.47) | 0.74 (0.64-0.87) | 1.04 (0.99-1.09) |  | 0.18 (0.12-0.26) | 0.12 (0.09-0.17) | 0.62 (0.55-0.69) |  | 6.82  (4.58-9.94) | 4.05 (2.72-5.86) | 0.89 (0.84-0.94) |
| Viet Nam | 5915.88 (4788.78-7108.28) | 6.29 (5.11-7.45) | 0.84 (0.79-0.89) |  | 758.82 (660.19-876.91) | 0.82 (0.73-0.93) | 1.38 (1.31-1.45) |  | 90.87 (57.70-186.63) | 0.12 (0.07-0.25) | -1.04  (-1.09--0.99) |  | 1797.55 (1236.66-3144.31) | 2.09 (1.42-3.84) | -0.98 (-1.08--0.89) |
| China | 100164.53 (80480.48-120558.59) | 5.03 (4.11-5.97) | -0.79 (-0.98--0.59) |  | 11803.18 (9965.15-13751.04) | 0.60 (0.51-0.70) | -0.48  (-0.59--0.37) |  | 817.54  (650.78-984.20) | 0.05  (0.04-0.06) | -2.48  (-2.87--2.08) |  | 17820.09 (14668.68-21149.76) | 0.96 (0.79-1.14) | -3.59 (-4.03--3.15) |
| Myanmar | 1801.53 (1449.15-2192.51) | 3.49 (2.86-4.20) | 1.39 (1.32-1.47) |  | 198.40 (160.84-244.77) | 0.38 (0.31-0.46) | 1.63 (1.54-1.71) |  | 35.20  (16.16-68.21) | 0.08 (0.04-0.17) | -2.24  (-2.48--2.00) |  | 1012.51 (500.73-1753.92) | 2.05 (1.02-3.63) | -3.15 (-3.39--2.91) |
| Albania | 367.00 (299.04-443.48) | 11.22 (9.14-13.85) | 1.14 (1.01-1.26) |  | 54.40 (46.51-63.65) | 1.62 (1.36-1.94) | 1.43 (1.22-1.65) |  | 16.45 (11.78-22.18) | 0.40 (0.29-0.53) | -0.89  (-1.01--0.78) |  | 273.10 (203.28-356.23) | 6.55 (4.89-8.49) | -1.02 (-1.20--0.85) |
| Fiji | 49.83  (41.40-59.52) | 6.05 (5.06-7.12) | 0.55 (0.52-0.59) |  | 5.73  (4.81-6.86) | 0.68 (0.58-0.80) | 0.60 (0.55-0.64) |  | 1.30 (0.95-1.68) | 0.17 (0.13-0.22) | 0.56 (0.40-0.72) |  | 46.17  (33.66-61.02) | 5.33 (3.92-6.94) | -0.07 (-0.23-0.10) |
| Taiwan (Province of China) | 10124.92 (9025.84-12142.55) | 26.16 (23.44-31.14) | 2.30 (1.66-2.95) |  | 1358.83  (1250.22-1490.84) | 3.53 (3.24-3.85) | 3.26 (2.70-3.82) |  | 515.74 (431.35-578.70) | 1.17 (0.98-1.31) | 7.62 (5.96-9.31) |  | 8471.58 (7360.58-9389.02) | 20.30 (17.75-22.41) | 2.70 (1.16-4.28) |
| Malaysia | 3141.86 (2575.69-3749.79) | 11.25 (9.31-13.18) | 2.10 (1.96-2.23) |  | 435.76 (388.92-488.92) | 1.57 (1.43-1.74) | 2.99 (2.82-3.16) |  | 325.68 (252.30-393.53) | 1.45 (1.10-1.78) | 2.11 (1.78-2.44) |  | 6460.45 (5133.45-7527.47) | 24.93 (19.67-29.39) | 1.01 (0.72-1.29) |
| Tonga | 6.69  (5.54-7.89) | 7.83 (6.47-9.23) | 0.75 (0.70-0.80) |  | 0.83  (0.73-0.97) | 0.96 (0.85-1.08) | 1.01 (0.96-1.06) |  | 0.17 (0.12-0.25) | 0.21 (0.15-0.30) | 0.65 (0.51-0.80) |  | 5.70  (4.01-8.03) | 6.51 (4.60-9.20) | 1.12 (0.91-1.32) |
| Mongolia | 347.98 (278.13-434.53) | 11.88 (9.68-14.50) | 1.30 (1.20-1.39) |  | 38.92 (31.28-49.24) | 1.29 (1.07-1.59) | 1.43 (1.32-1.55) |  | 9.36 (7.03-12.01) | 0.50 (0.37-0.65) | 0.95 (0.82-1.08) |  | 252.55 (197.22-319.49) | 10.75 (8.20-13.67) | 0.56 (0.38-0.75) |
| Turkmenistan | 657.64 (539.92-799.44) | 14.38 (11.96-17.22) | 1.45 (1.34-1.56) |  | 73.29 (61.13-90.89) | 1.57 (1.34-1.91) | 1.46 (1.37-1.56) |  | 27.48 (20.91-36.12) | 0.79 (0.60-1.02) | 0.02  (-0.08-0.13) |  | 721.30 (542.90-943.48) | 17.69 (13.52-23.16) | 0.01 (-0.15-0.17) |
| Bulgaria | 1751.31 (1490.16-2068.49) | 16.10 (13.70-18.99) | 1.27 (1.13-1.42) |  | 332.64 (306.44-359.84) | 2.79 (2.49-3.14) | 1.87 (1.69-2.05) |  | 252.63 (212.36-293.95) | 1.68 (1.42-1.96) | 0.06  (-0.22-0.34) |  | 4508.83 (3782.32-5292.13) | 31.38 (26.26-36.93) | 0.21 (-0.17-0.58) |
| Poland | 6666.63 (6061.69-7383.46) | 11.59 (10.44-12.85) | -1.25 (-1.71--0.79) |  | 1325.60 (1227.61-1415.90) | 2.14 (1.95-2.33) | -0.19 (-0.68-0.30) |  | 1856.05 (1630.80-2033.51) | 2.39 (2.10-2.62) | -1.12  (-1.45--0.80) |  | 30595.03 (27291.35-33612.75) | 41.52 (37.24-45.49) | -1.94 (-2.28--1.60) |
| Armenia | 1035.49 (887.31-1239.10) | 26.35 (22.68-30.93) | 1.33 (1.26-1.40) |  | 165.86 (154.09-178.63) | 4.12 (3.78-4.49) | 1.94 (1.84-2.04) |  | 213.45 (176.18-246.12) | 4.94 (4.09-5.70) | 1.71 (1.55-1.87) |  | 3806.31 (3167.31-4389.58) | 89.75 (74.82-103.41) | 1.23 (0.95-1.52) |
| Papua New Guinea | 264.24 (208.31-329.95) | 3.48 (2.87-4.19) | 0.29 (0.21-0.36) |  | 31.25 (24.60-40.78) | 0.37 (0.30-0.46) | 0.28 (0.20-0.36) |  | 3.73 (2.58-5.27) | 0.06 (0.04-0.08) | 0.07 (0.03-0.12) |  | 172.64 (122.25-241.28) | 2.11 (1.50-2.96) | 0.06 (-0.00-0.12) |
| Sri Lanka | 1495.84 (1191.84-1780.58) | 5.80 (4.67-6.80) | 1.36 (1.31-1.42) |  | 179.54 (151.90-209.33) | 0.70 (0.60-0.83) | 1.78 (1.71-1.84) |  | 41.20 (27.07-56.63) | 0.19 (0.12-0.25) | -1.82  (-1.93--1.72) |  | 800.60 (535.65-1070.30) | 3.30 (2.23-4.43) | -1.78 (-1.91--1.66) |
| Lithuania | 2731.91 (2251.29-3161.91) | 45.74 (38.36-53.06) | 1.35 (1.13-1.56) |  | 349.79 (326.81-375.08) | 6.00 (5.61-6.45) | 2.17 (2.11-2.24) |  | 311.30 (269.37-347.84) | 4.65 (4.04-5.19) | 3.31 (2.91-3.71) |  | 5025.02 (4400.13-5607.20) | 84.70 (73.86-94.69) | 2.87 (2.20-3.54) |
| Cambodia | 571.25 (463.26-676.88) | 4.49 (3.65-5.25) | 1.33 (1.27-1.38) |  | 66.00 (55.84-79.61) | 0.51 (0.44-0.59) | 1.89 (1.81-1.96) |  | 19.75 (11.10-47.82) | 0.23 (0.13-0.55) | -1.26  (-1.41--1.11) |  | 466.71 (270.99-1012.21) | 4.20 (2.41-9.73) | -1.85 (-2.03--1.67) |
| Serbia | 2891.88 (2533.31-3292.80) | 20.62 (17.98-23.69) | 0.55 (0.42-0.69) |  | 623.75 (581.87-666.01) | 4.11 (3.77-4.46) | 1.13 (1.02-1.24) |  | 513.18 (410.96-633.37) | 2.92 (2.35-3.62) | -0.87  (-1.01--0.73) |  | 8849.73 (7110.05-10830.08) | 52.08 (41.92-63.52) | -1.37 (-1.53--1.21) |
| Marshall Islands | 2.45  (2.04-2.96) | 6.01 (4.99-7.09) | 0.47 (0.44-0.50) |  | 0.29  (0.25-0.35) | 0.67 (0.59-0.78) | 0.67 (0.62-0.71) |  | 0.07 (0.04-0.10) | 0.17 (0.11-0.25) | 0.59 (0.45-0.72) |  | 2.98  (1.85-4.39) | 6.02 (3.85-8.60) | 0.79 (0.56-1.03) |
| Japan | 110167.22 (95204.72-126274.59) | 42.91 (36.37-50.78) | -0.01 (-0.11-0.08) |  | 11257.63 (10099.81-12639.09) | 4.52 (3.91-5.32) | -0.20 (-0.29--0.12) |  | 4732.82 (3698.93-5351.67) | 0.88 (0.73-0.97) | 0.30 (0.10-0.51) |  | 63380.17 (52855.42-69876.29) | 15.71 (13.79-16.99) | 0.29 (0.03-0.54) |
| Iceland | 139.10 (113.31-166.64) | 26.55 (21.75-31.92) | 0.12 (-0.18-0.43) |  | 18.81 (17.09-20.76) | 3.58 (3.21-4.02) | -0.35 (-0.64--0.05) |  | 7.84 (6.28-9.09) | 1.19 (0.96-1.38) | -0.98  (-1.23--0.74) |  | 124.67 (106.36-142.24) | 20.88 (18.12-23.74) | -1.75 (-2.01--1.48) |
| Russian Federation | 105185.23 (89855.75-121158.56) | 46.77 (40.42-53.75) | 0.99 (0.86-1.12) |  | 11654.46 (10963.61-12345.50) | 5.24 (4.90-5.61) | 0.77 (0.65-0.90) |  | 13639.15 (12560.96-14766.24) | 5.64 (5.19-6.11) | 2.33 (2.18-2.47) |  | 240966.60 (222533.12-260610.91) | 101.48 (93.83-109.66) | 1.74 (1.50-1.97) |
| Belgium | 7721.77 (6225.93-9548.11) | 35.50 (28.95-44.09) | 0.53 (0.40-0.66) |  | 1443.35 (1347.32-1543.60) | 6.38 (5.92-6.85) | 0.85 (0.65-1.04) |  | 726.39 (601.54-808.45) | 2.60 (2.20-2.88) | -1.03  (-1.25--0.81) |  | 11415.01 (9906.05-12584.75) | 46.57 (41.37-50.98) | -1.34 (-1.53--1.15) |
| Slovenia | 969.33 (831.31-1127.89) | 25.93 (22.10-30.31) | 0.18 (-0.09-0.44) |  | 214.74 (197.38-231.27) | 5.17 (4.71-5.65) | 0.69 (0.64-0.74) |  | 127.25 (106.28-144.20) | 2.36 (1.98-2.67) | -0.70  (-1.08--0.32) |  | 1823.08 (1551.49-2065.25) | 37.20 (32.03-42.13) | -2.37 (-2.75--2.00) |
| Hungary | 3706.96 (3190.77-4281.87) | 23.05 (19.77-26.74) | 0.31 (0.23-0.38) |  | 831.33 (774.51-892.55) | 4.76 (4.38-5.17) | 0.60 (0.43-0.76) |  | 589.83 (513.29-663.68) | 2.78 (2.43-3.13) | -1.01  (-1.32--0.69) |  | 10572.95 (9187.91-11953.57) | 53.68 (46.44-60.92) | -2.00 (-2.21--1.79) |
| Singapore | 3859.73 (3244.90-4581.95) | 48.96 (41.17-57.40) | 1.29 (1.07-1.50) |  | 397.75 (345.06-468.37) | 5.15 (4.45-6.01) | 1.32 (1.09-1.55) |  | 56.66 (48.47-62.64) | 0.70 (0.59-0.77) | -0.19  (-0.36--0.02) |  | 1078.28 (965.67-1179.38) | 13.21 (11.83-14.47) | -0.60 (-0.87--0.34) |
| Montenegro | 262.01 (229.27-297.02) | 30.27 (26.56-34.27) | 0.97 (0.84-1.09) |  | 65.68 (60.68-70.23) | 7.09 (6.54-7.63) | 1.33 (1.19-1.48) |  | 41.93 (33.16-51.44) | 4.67 (3.72-5.74) | 0.25 (0.09-0.41) |  | 786.03 (628.53-969.56) | 83.26 (66.94-102.40) | 0.15 (0.04-0.26) |
| Denmark | 1645.21 (1317.82-2036.98) | 14.79 (11.85-17.82) | -0.37 (-0.49--0.26) |  | 361.14 (329.12-395.45) | 3.09 (2.78-3.42) | -0.46 (-0.76--0.16) |  | 152.07 (131.17-168.46) | 1.12 (0.98-1.24) | -1.33  (-1.82--0.84) |  | 2411.22 (2147.36-2627.87) | 19.31 (17.40-20.97) | -2.85 (-3.35--2.35) |
| Australia | 11665.71 (9307.50-14283.28) | 26.68 (21.46-32.52) | 0.10 (0.02-0.18) |  | 1615.57 (1471.27-1764.37) | 3.67 (3.29-4.04) | 0.30 (0.17-0.42) |  | 573.15 (480.42-643.72) | 1.10 (0.93-1.23) | -1.60  (-1.75--1.44) |  | 8702.45 (7608.51-9661.45) | 18.27 (16.12-20.17) | -2.08 (-2.23--1.93) |
| Belarus | 5990.95 (4869.21-7270.57) | 39.00 (32.20-47.00) | 2.13 (1.98-2.29) |  | 750.91 (694.67-807.96) | 4.94 (4.54-5.34) | 2.63 (2.45-2.80) |  | 556.79 (454.59-667.33) | 3.39 (2.76-4.07) | 1.05 (0.84-1.26) |  | 9795.35 (8006.12-11969.58) | 61.03 (49.81-74.84) | 0.14 (-0.09-0.37) |
| Israel | 2378.08 (1914.36-3005.95) | 19.70 (15.87-24.66) | 0.41 (0.32-0.50) |  | 447.52 (412.66-486.35) | 3.64 (3.34-4.00) | 0.86 (0.70-1.02) |  | 234.60 (194.03-260.17) | 1.68 (1.40-1.86) | -1.07  (-1.21--0.92) |  | 3479.22 (2983.13-3825.08) | 26.60 (22.98-29.20) | -1.59 (-1.75--1.42) |
| Latvia | 1999.09 (1612.33-2386.46) | 48.53 (39.26-57.50) | 0.29 (-0.03-0.61) |  | 235.96 (219.82-250.82) | 5.92 (5.48-6.36) | 0.44 (0.35-0.54) |  | 216.18 (186.74-243.01) | 4.81 (4.16-5.45) | 1.61 (1.16-2.07) |  | 3691.88 (3207.42-4190.55) | 93.02 (80.86-105.49) | 0.74 (0.16-1.33) |
| Brunei Darussalam | 167.06 (137.35-198.89) | 40.97 (35.14-47.10) | 0.39 (0.23-0.56) |  | 17.07 (14.46-20.41) | 4.15 (3.64-4.78) | 0.43 (0.25-0.61) |  | 2.40 (1.81-3.00) | 0.96 (0.70-1.21) | -0.26  (-0.49--0.04) |  | 62.28  (48.45-76.14) | 18.88 (14.53-23.27) | -0.27 (-0.55--0.00) |
| France | 28956.04 (23294.15-36370.12) | 22.37 (18.15-27.77) | 0.29 (0.23-0.35) |  | 5975.34 (5562.19-6367.40) | 4.45 (4.07-4.82) | 0.78 (0.68-0.89) |  | 3381.83 (2805.05-3807.95) | 1.87 (1.60-2.10) | -1.66  (-1.76--1.56) |  | 49660.36 (43387.51-54898.17) | 32.97 (29.45-36.30) | -1.56 (-1.62--1.49) |
| Luxembourg | 301.31 (251.30-356.62) | 30.15 (25.16-35.49) | -0.38 (-0.62--0.14) |  | 55.85 (51.40-60.38) | 5.45 (4.98-5.92) | 0.25 (-0.02-0.53) |  | 27.20 (23.37-30.77) | 2.25 (1.97-2.55) | -1.38  (-1.55--1.21) |  | 409.58 (362.01-462.30) | 36.61 (32.55-41.33) | -2.00 (-2.19--1.81) |
| Slovakia | 1009.65 (840.37-1178.65) | 13.10 (10.84-15.35) | 1.04 (0.62-1.47) |  | 206.67 (187.77-226.88) | 2.48 (2.21-2.80) | 2.23 (2.08-2.38) |  | 140.63 (108.30-174.32) | 1.46 (1.13-1.81) | -0.39  (-0.48--0.31) |  | 2447.37 (1908.62-3016.64) | 25.72 (20.15-31.65) | -0.65 (-0.80--0.51) |
| Republic of Moldova | 1868.12 (1502.44-2278.44) | 32.98 (26.93-39.71) | 2.35 (2.15-2.55) |  | 262.45 (244.78-281.71) | 4.65 (4.31-5.00) | 2.98 (2.77-3.19) |  | 227.70 (199.85-259.82) | 3.75 (3.30-4.28) | 2.78 (2.54-3.03) |  | 4417.63 (3925.09-5011.60) | 73.99 (65.67-84.10) | 3.12 (2.77-3.48) |
| Romania | 7994.10 (6707.61-9529.20) | 27.80 (23.44-32.59) | 1.57 (1.48-1.67) |  | 1445.29 (1343.08-1550.82) | 4.65 (4.21-5.16) | 2.37 (2.30-2.44) |  | 1193.36 (1013.57-1391.19) | 2.98 (2.53-3.45) | 0.95 (0.81-1.08) |  | 21843.33 (18782.80-25334.40) | 59.03 (50.87-68.31) | 0.68 (0.51-0.85) |
| Czechia | 2746.04 (2283.93-3375.51) | 16.68 (13.76-20.04) | -0.02 (-0.25-0.20) |  | 521.32 (473.54-569.96) | 2.89 (2.56-3.29) | 0.18 (0.00-0.35) |  | 300.97 (261.90-341.77) | 1.28 (1.11-1.45) | -1.36  (-1.70--1.02) |  | 5054.83 (4410.38-5714.03) | 22.87 (20.08-25.94) | -2.27 (-2.61--1.94) |
| Greece | 3570.55 (2963.52-4249.14) | 17.49 (14.62-21.03) | 0.34 (-0.11-0.79) |  | 583.53 (536.45-637.52) | 2.75 (2.46-3.09) | 0.54 (0.35-0.73) |  | 403.51 (335.25-457.96) | 1.25 (1.06-1.41) | -0.48  (-0.75--0.21) |  | 5591.22 (4797.03-6231.69) | 20.47 (17.93-22.55) | 0.11 (-0.20-0.42) |
| Republic of Korea | 35168.12 (29030.08-42245.44) | 43.87 (36.60-52.72) | 1.57 (1.32-1.82) |  | 3661.83 (3156.40-4322.31) | 4.66 (3.98-5.51) | 1.65 (1.38-1.91) |  | 609.47 (471.62-743.28) | 0.67 (0.52-0.81) | -1.04  (-1.30--0.78) |  | 10302.70 (8585.65-12125.62) | 11.54 (9.71-13.55) | -0.66 (-0.86--0.46) |
| North Macedonia | 387.95 (323.81-464.18) | 14.33 (12.04-16.94) | 0.36 (0.29-0.44) |  | 68.75 (61.74-75.98) | 2.39 (2.11-2.70) | 0.55 (0.36-0.74) |  | 48.75 (36.36-60.12) | 1.86 (1.38-2.25) | 0.74 (0.43-1.06) |  | 924.04 (692.87-1139.20) | 30.81 (23.71-37.54) | -0.66 (-0.94--0.38) |
| Cyprus | 192.96 (162.16-228.51) | 9.52 (8.08-11.23) | 0.62 (0.55-0.68) |  | 49.27 (44.17-54.44) | 2.36 (2.12-2.61) | 1.47 (1.33-1.61) |  | 24.63 (18.63-31.12) | 1.43 (1.08-1.84) | -2.78  (-3.00--2.55) |  | 384.65 (303.69-474.23) | 20.40 (15.91-25.42) | -3.33 (-3.49--3.17) |
| Netherlands | 7629.16 (5960.16-9250.25) | 23.28 (18.57-28.16) | 0.40 (0.29-0.51) |  | 1548.50 (1426.94-1681.12) | 4.55 (4.15-4.97) | 0.73 (0.54-0.92) |  | 597.36 (506.27-659.85) | 1.51 (1.29-1.67) | -0.98  (-1.22--0.74) |  | 9495.54 (8355.90-10427.27) | 25.80 (22.96-28.27) | -1.62 (-1.87--1.37) |
| Ireland | 1734.73 (1398.76-2163.37) | 23.01 (18.75-28.19) | 1.32 (1.24-1.41) |  | 354.39 (327.86-383.45) | 4.56 (4.20-4.98) | 1.85 (1.69-2.01) |  | 116.50 (97.71-130.38) | 1.37 (1.15-1.53) | -0.87  (-1.03--0.71) |  | 1920.14 (1675.86-2105.79) | 23.66 (20.78-25.89) | -1.33 (-1.54--1.12) |
| Ukraine | 24620.10 (21364.64-28678.07) | 33.82 (29.75-39.58) | -0.58 (-0.69--0.47) |  | 2614.79 (2451.19-2795.65) | 3.68 (3.42-4.01) | -0.70 (-0.87--0.54) |  | 2640.23 (2036.75-3307.77) | 3.28 (2.53-4.12) | 0.22 (0.04-0.41) |  | 49989.98 (38008.33-63140.66) | 64.27 (48.41-81.69) | -0.47 (-0.67--0.28) |
| Austria | 3955.29 (3322.15-4524.23) | 22.83 (19.43-26.03) | 1.28 (0.79-1.78) |  | 518.47 (475.91-562.90) | 3.02 (2.74-3.32) | -0.25 (-0.60-0.10) |  | 222.73 (186.08-246.53) | 1.04 (0.88-1.15) | -2.31  (-2.65--1.98) |  | 3500.11 (3063.36-3823.43) | 18.42 (16.47-20.12) | -2.87 (-3.18--2.55) |
| New Zealand | 1954.88 (1637.16-2212.15) | 23.85 (20.34-26.97) | -0.65 (-1.12--0.18) |  | 242.43 (223.69-262.09) | 2.96 (2.71-3.23) | -1.38 (-1.64--1.12) |  | 121.70 (102.04-134.66) | 1.31 (1.10-1.44) | -1.45  (-1.82--1.08) |  | 1882.40 (1645.30-2060.63) | 21.42 (18.88-23.33) | -1.76 (-2.41--1.11) |
| Spain | 33736.28 (29853.36-38293.39) | 36.31 (31.87-41.23) | 1.06 (0.89-1.24) |  | 6127.70 (5694.34-6524.87) | 6.17 (5.71-6.61) | 0.90 (0.55-1.25) |  | 3269.11 (2622.66-3646.44) | 2.53 (2.08-2.80) | -0.83  (-1.15--0.52) |  | 45254.22 (37897.06-49892.20) | 40.86 (35.60-44.67) | -1.83 (-2.06--1.59) |
| Finland | 2299.29 (1870.20-2892.01) | 19.09 (15.51-23.52) | 0.13 (-0.03-0.28) |  | 497.06 (464.05-535.15) | 3.89 (3.57-4.25) | 0.64 (0.53-0.75) |  | 221.59 (184.70-244.98) | 1.42 (1.21-1.56) | -1.52  (-1.77--1.27) |  | 3325.52 (2915.43-3646.43) | 24.40 (21.83-26.44) | -2.11 (-2.26--1.96) |
| Italy | 24470.44 (20795.83-28400.34) | 17.26 (14.83-19.96) | -1.82 (-2.24--1.39) |  | 4191.58 (3906.83-4492.56) | 2.87 (2.64-3.12) | -1.96 (-2.13--1.80) |  | 2334.20 (1898.84-2595.19) | 1.22 (1.02-1.35) | -1.67  (-1.79--1.55) |  | 32699.04 (27948.83-35599.17) | 19.99 (17.65-21.53) | -2.04 (-2.25--1.83) |
| Estonia | 1416.38 (1107.39-1654.12) | 50.65 (40.29-59.47) | 1.04 (0.86-1.22) |  | 178.27 (165.97-190.76) | 6.53 (6.08-7.04) | 1.19 (0.98-1.41) |  | 122.92 (104.32-138.19) | 3.95 (3.38-4.43) | 1.56 (1.08-2.05) |  | 2024.83 (1756.78-2277.47) | 74.10 (64.30-83.58) | 0.61 (-0.07-1.30) |
| Uruguay | 1911.73 (1605.10-2234.12) | 34.83 (29.13-40.74) | 0.48 (0.40-0.56) |  | 333.40 (312.62-356.71) | 6.01 (5.59-6.42) | 1.24 (1.11-1.37) |  | 295.80 (261.97-324.13) | 4.63 (4.12-5.07) | -0.67  (-0.82--0.51) |  | 4868.56 (4396.02-5292.46) | 85.79 (78.25-92.93) | -0.97 (-1.18--0.76) |
| Jamaica | 247.37 (204.08-295.29) | 8.31 (6.86-9.94) | 0.25 (0.19-0.30) |  | 31.21 (26.69-37.47) | 1.06 (0.91-1.28) | 0.36 (0.28-0.43) |  | 13.49 (10.49-17.08) | 0.42 (0.33-0.53) | -1.01  (-1.21--0.80) |  | 280.07 (215.36-360.73) | 9.03 (6.94-11.63) | -0.77 (-1.07--0.47) |
| Malta | 102.13  (85.04-122.42) | 10.84 (9.00-12.95) | 0.20 (0.06-0.34) |  | 25.46 (23.37-27.71) | 2.58 (2.34-2.83) | 0.50 (0.27-0.74) |  | 13.47 (11.37-15.35) | 1.21 (1.03-1.38) | -1.27  (-1.53--1.00) |  | 215.87 (191.22-242.30) | 21.33 (19.00-23.84) | -1.84 (-2.14--1.54) |
| Belize | 26.22  (21.23-32.18) | 7.30 (6.08-8.74) | 0.58 (0.43-0.72) |  | 3.27  (2.69-4.11) | 0.88 (0.75-1.08) | 0.70 (0.54-0.87) |  | 1.24 (1.07-1.42) | 0.46 (0.40-0.52) | -0.11  (-0.42-0.21) |  | 29.97  (26.03-34.22) | 9.69 (8.39-11.06) | -0.52 (-0.97--0.07) |
| Andorra | 34.78  (27.78-42.58) | 23.99 (19.28-29.14) | 0.68 (0.61-0.75) |  | 6.95  (6.25-7.64) | 4.72 (4.22-5.18) | 1.22 (1.08-1.36) |  | 2.55 (1.71-3.45) | 1.47 (0.98-1.99) | -0.65  (-0.76--0.53) |  | 38.82  (27.09-52.06) | 24.19 (16.81-32.50) | -0.76 (-0.95--0.57) |
| Switzerland | 3135.69 (2548.13-3791.82) | 18.90 (15.45-22.97) | 1.02 (0.63-1.41) |  | 565.46 (522.93-620.41) | 3.26 (2.94-3.62) | 1.01 (0.76-1.27) |  | 220.79 (177.61-251.24) | 0.98 (0.81-1.11) | -0.72  (-0.98--0.46) |  | 3236.57 (2755.12-3645.15) | 16.25 (14.08-18.22) | -1.18 (-1.52--0.83) |
| Germany | 53694.56 (43182.67-64682.52) | 30.93 (25.19-37.18) | 0.56 (0.16-0.96) |  | 9418.34 (8745.49-10064.14) | 5.17 (4.74-5.62) | 0.21 (-0.07-0.49) |  | 3849.78 (3240.44-4250.53) | 1.69 (1.46-1.85) | -2.01  (-2.28--1.73) |  | 62311.16 (55229.79-67820.33) | 31.38 (28.44-33.92) | -2.28 (-2.55--2.01) |
| Canada | 39313.39 (33446.13-45339.01) | 58.35 (50.05-67.37) | 0.37 (0.28-0.46) |  | 4091.48 (3787.48-4416.47) | 6.18 (5.62-6.80) | 0.44 (0.33-0.55) |  | 1267.10 (1087.81-1406.21) | 1.57 (1.37-1.74) | -1.28  (-1.38--1.19) |  | 21179.99 (18944.92-22979.77) | 29.19 (26.38-31.60) | -1.38 (-1.50--1.26) |
| Dominica | 9.11  (7.61-10.85) | 11.72 (9.91-13.92) | 0.26 (0.20-0.32) |  | 1.29  (1.16-1.43) | 1.65 (1.48-1.86) | 0.35 (0.26-0.44) |  | 1.32 (1.00-1.67) | 1.71 (1.30-2.17) | -0.25  (-0.34--0.15) |  | 26.16  (19.96-33.41) | 32.59 (24.86-41.16) | -0.52 (-0.56--0.48) |
| Saint Vincent and the Grenadines | 8.96  (7.26-10.78) | 6.91 (5.66-8.24) | 0.43 (0.31-0.55) |  | 1.06  (0.87-1.28) | 0.83 (0.69-1.01) | 0.47 (0.32-0.63) |  | 0.39 (0.34-0.45) | 0.30 (0.26-0.33) | -1.42  (-1.62--1.21) |  | 8.91  (7.80-10.15) | 6.46 (5.66-7.35) | -1.42 (-1.72--1.13) |
| Nicaragua | 614.31 (519.83-722.72) | 11.69 (9.98-13.76) | 1.17 (1.07-1.28) |  | 77.67 (66.35-93.23) | 1.44 (1.26-1.67) | 1.64 (1.50-1.77) |  | 28.38 (21.71-35.66) | 0.66 (0.51-0.83) | -0.61  (-0.83--0.40) |  | 620.22 (479.36-766.21) | 12.81 (9.94-15.95) | -0.39 (-0.64--0.15) |
| Portugal | 5387.19 (4675.48-6228.76) | 22.44 (19.55-26.07) | 1.63 (1.34-1.92) |  | 1290.49 (1204.20-1376.93) | 5.13 (4.74-5.55) | 2.84 (2.49-3.18) |  | 908.90 (739.07-1025.30) | 2.89 (2.39-3.23) | 0.86 (0.55-1.17) |  | 12686.96 (10770.40-14072.83) | 45.78 (39.95-50.36) | -0.31 (-0.52--0.09) |
| Antigua and Barbuda | 9.00  (7.33-10.90) | 9.05 (7.49-10.86) | 0.42 (0.32-0.52) |  | 1.10  (0.92-1.32) | 1.11 (0.94-1.33) | 0.51 (0.38-0.64) |  | 0.41 (0.37-0.45) | 0.44 (0.40-0.48) | -1.07  (-1.28--0.85) |  | 8.63  (7.74-9.56) | 8.43 (7.65-9.27) | -1.73 (-2.03--1.43) |
| Grenada | 9.82  (8.12-11.94) | 8.99 (7.45-10.76) | 0.98 (0.87-1.09) |  | 1.22  (1.03-1.47) | 1.12 (0.95-1.33) | 1.14 (0.99-1.29) |  | 0.86 (0.75-1.00) | 0.86 (0.75-0.98) | -0.78  (-0.91--0.66) |  | 19.44  (16.66-22.49) | 17.52 (15.14-20.18) | -1.17 (-1.32--1.03) |
| Norway | 2128.44 (1755.76-2561.80) | 23.27 (19.11-27.86) | -0.70 (-0.82--0.59) |  | 292.15 (268.15-317.73) | 3.13 (2.82-3.48) | -1.13 (-1.33--0.94) |  | 113.75 (96.08-124.57) | 0.97 (0.83-1.06) | -1.51  (-1.75--1.28) |  | 1713.21 (1509.38-1860.51) | 15.82 (14.04-17.13) | -2.22 (-2.48--1.96) |
| Chile | 5828.63 (5203.26-6535.42) | 23.43 (20.99-26.27) | 0.54 (0.06-1.02) |  | 970.52 (897.01-1043.98) | 3.87 (3.56-4.17) | 1.75 (1.71-1.78) |  | 487.69 (428.99-545.07) | 1.86 (1.64-2.08) | -1.61  (-1.74--1.47) |  | 8829.06 (7937.47-9776.95) | 34.48 (31.09-38.11) | -1.91 (-2.04--1.78) |
| Costa Rica | 1514.13 (1253.03-1797.15) | 28.23 (23.38-33.47) | 0.59 (0.56-0.63) |  | 251.50 (232.82-270.08) | 4.73 (4.37-5.10) | 1.46 (1.36-1.56) |  | 166.09 (141.04-188.01) | 3.02 (2.57-3.41) | -0.39  (-0.60--0.19) |  | 3064.77 (2646.99-3459.60) | 56.22 (48.54-63.48) | -0.94 (-1.14--0.74) |
| Trinidad and Tobago | 180.57 (149.03-213.25) | 10.55 (8.74-12.37) | 0.29 (0.22-0.36) |  | 21.60 (18.85-25.23) | 1.28 (1.11-1.53) | 0.32 (0.22-0.41) |  | 14.31 (10.63-18.33) | 0.76 (0.57-0.97) | -2.15  (-2.26--2.04) |  | 321.64 (236.14-417.97) | 17.12 (12.65-22.15) | -2.06 (-2.25--1.88) |
| Guatemala | 1081.17 (894.21-1287.02) | 8.46 (7.13-9.83) | 0.55 (0.41-0.69) |  | 130.30 (107.99-165.00) | 0.96 (0.82-1.17) | 0.83 (0.64-1.01) |  | 64.73 (55.72-74.78) | 0.65 (0.57-0.75) | -1.26  (-1.49--1.03) |  | 1464.87 (1243.62-1698.06) | 13.04 (11.16-15.09) | -1.98 (-2.17--1.80) |
| Haiti | 535.49 (437.49-650.30) | 5.80 (4.77-6.81) | -0.11 (-0.15--0.07) |  | 64.29 (52.88-81.27) | 0.65 (0.56-0.77) | 0.17 (0.10-0.23) |  | 71.02 (38.33-111.77) | 1.20 (0.63-1.90) | -0.84  (-0.90--0.78) |  | 1921.54 (1077.16-2944.16) | 25.53 (14.00-39.83) | -1.03 (-1.10--0.96) |
| Barbados | 41.34  (33.39-49.24) | 9.62 (7.92-11.45) | 0.27 (0.18-0.35) |  | 5.22  (4.58-6.00) | 1.24 (1.07-1.47) | 0.45 (0.36-0.55) |  | 3.34 (2.50-4.16) | 0.65 (0.49-0.80) | -0.95  (-1.05--0.85) |  | 63.43  (47.61-79.31) | 12.77 (9.65-15.92) | -1.02 (-1.13--0.91) |
| Sweden | 3514.98 (2766.73-4419.69) | 17.34 (13.89-21.66) | -1.64 (-1.98--1.30) |  | 571.01 (529.61-619.11) | 2.70 (2.46-3.00) | -2.22 (-2.37--2.08) |  | 238.86 (196.39-276.98) | 0.89 (0.73-1.02) | -2.49  (-2.73--2.26) |  | 3454.80 (2917.87-3964.45) | 14.18 (12.18-16.08) | -3.07 (-3.24--2.90) |
| United States of America | 271866.88 (244550.13-300815.82) | 49.67 (44.90-54.70) | -0.62 (-0.71--0.53) |  | 27547.63 (25782.73-29403.04) | 5.15 (4.76-5.57) | -0.81 (-0.86--0.76) |  | 10068.57 (8817.33-10710.39) | 1.64 (1.45-1.74) | -1.08  (-1.34--0.82) |  | 192482.18 (177377.15-201863.22) | 34.21 (31.82-35.78) | -1.52 (-1.71--1.33) |
| Venezuela (Bolivarian Republic of) | 5617.11 (4586.52-6686.33) | 19.53 (16.10-23.07) | 0.74 (0.62-0.87) |  | 830.24 (756.53-909.94) | 2.89 (2.63-3.16) | 1.63 (1.45-1.81) |  | 619.45 (454.17-799.09) | 2.25 (1.66-2.87) | -0.47  (-0.61--0.34) |  | 12724.82 (8965.32-16672.06) | 44.06 (31.39-57.48) | -0.27 (-0.40--0.15) |
| Bolivia (Plurinational State of) | 779.57 (648.00-927.13) | 8.37 (6.99-9.84) | 0.83 (0.79-0.88) |  | 105.99 (92.01-124.53) | 1.10 (0.97-1.26) | 1.46 (1.41-1.51) |  | 119.40 (75.66-171.13) | 1.59 (1.00-2.26) | -0.68  (-0.73--0.63) |  | 2514.25 (1635.27-3607.25) | 28.99 (18.87-41.16) | -1.28 (-1.35--1.21) |
| Mexico | 17659.44 (15208.98-20715.51) | 14.26 (12.27-16.73) | -1.79 (-1.96--1.62) |  | 2496.51 (2271.51-2738.32) | 2.00 (1.83-2.19) | -0.96 (-1.11--0.81) |  | 2596.46 (2278.26-2899.87) | 2.24 (1.98-2.50) | -1.18  (-1.27--1.10) |  | 52916.62 (46267.15-59316.00) | 42.80 (37.47-47.93) | -1.01 (-1.17--0.85) |
| Saint Lucia | 23.34  (19.47-28.10) | 10.62 (8.86-12.70) | 0.45 (0.40-0.49) |  | 3.09  (2.72-3.52) | 1.42 (1.24-1.65) | 0.70 (0.63-0.77) |  | 2.33 (1.82-2.78) | 1.02 (0.79-1.21) | -2.00  (-2.21--1.78) |  | 45.08  (36.05-54.43) | 19.40 (15.47-23.40) | -2.11 (-2.32--1.91) |
| United Kingdom | 29908.58 (25091.92-35676.67) | 25.03 (21.14-29.74) | 0.25 (-0.19-0.69) |  | 5752.10 (5378.86-6131.80) | 4.65 (4.30-5.05) | 0.59 (0.51-0.66) |  | 3617.79 (3173.33-3869.46) | 2.44 (2.17-2.60) | 0.81 (0.67-0.94) |  | 58195.65 (52884.50-61756.22) | 44.11 (40.77-46.74) | 0.62 (0.44-0.81) |
| Dominican Republic | 952.82 (789.86-1120.48) | 9.14 (7.62-10.70) | 1.12 (1.04-1.21) |  | 116.87 (98.98-140.78) | 1.11 (0.95-1.33) | 1.25 (1.12-1.39) |  | 59.99 (39.14-83.80) | 0.62 (0.40-0.86) | -1.01  (-1.20--0.81) |  | 1311.75 (869.40-1799.99) | 12.96 (8.59-17.78) | -0.50 (-0.63--0.38) |
| Cuba | 3017.75 (2520.78-3588.75) | 16.87 (14.26-19.95) | 0.74 (0.68-0.81) |  | 568.68 (524.52-615.37) | 3.12 (2.86-3.40) | 1.25 (1.22-1.29) |  | 390.60 (334.95-451.71) | 1.90 (1.63-2.20) | -0.66  (-0.77--0.55) |  | 7347.87 (6292.17-8547.88) | 38.01 (32.57-44.27) | -0.79 (-0.91--0.67) |
| Argentina | 11863.14 (9802.40-14138.56) | 21.57 (17.91-25.71) | 0.27 (0.19-0.36) |  | 1493.99 (1359.40-1628.54) | 2.73 (2.48-3.00) | 0.64 (0.53-0.74) |  | 1007.18 (905.87-1102.51) | 1.74 (1.57-1.90) | -1.39  (-1.48--1.29) |  | 18978.53 (17472.33-20608.00) | 33.81 (31.21-36.67) | -1.37 (-1.50--1.24) |
| Peru | 2621.84 (2153.39-3078.55) | 7.65 (6.28-8.97) | 0.69 (0.62-0.76) |  | 350.64 (298.24-414.69) | 1.02 (0.88-1.20) | 1.07 (0.98-1.16) |  | 224.90 (163.34-293.98) | 0.68 (0.49-0.89) | -1.64  (-1.85--1.43) |  | 4306.72 (3186.17-5656.86) | 12.79 (9.44-16.85) | -2.21 (-2.60--1.82) |
| Colombia | 11129.16 (9255.32-13068.07) | 20.74 (17.29-24.33) | 0.96 (0.88-1.04) |  | 1630.90 (1497.27-1779.03) | 3.06 (2.81-3.36) | 1.83 (1.70-1.96) |  | 944.21 (782.05-1109.91) | 1.71 (1.41-2.01) | -0.76  (-1.02--0.51) |  | 18145.74 (15063.25-21362.32) | 33.20 (27.56-39.11) | -1.40 (-1.69--1.11) |
| Guyana | 61.34  (50.79-73.30) | 8.88 (7.44-10.50) | 0.70 (0.65-0.74) |  | 7.85  (6.74-9.30) | 1.11 (0.97-1.30) | 0.89 (0.84-0.95) |  | 5.52 (4.30-6.89) | 0.96 (0.76-1.19) | -0.65  (-0.84--0.47) |  | 144.80 (111.07-182.17) | 21.97 (17.04-27.39) | -0.20 (-0.43-0.04) |
| Panama | 753.34 (619.24-887.15) | 17.14 (14.04-20.20) | 0.73 (0.67-0.78) |  | 95.63 (85.54-108.50) | 2.19 (1.95-2.49) | 1.10 (1.02-1.17) |  | 51.16 (39.79-60.88) | 1.13 (0.88-1.34) | -0.94  (-1.07--0.81) |  | 970.63 (766.00-1152.00) | 21.85 (17.26-25.96) | -1.03 (-1.22--0.83) |
| El Salvador | 770.63 (645.80-896.53) | 12.21 (10.15-14.27) | 1.51 (1.36-1.66) |  | 93.93 (82.05-108.75) | 1.50 (1.31-1.73) | 1.94 (1.73-2.15) |  | 80.21 (60.27-104.20) | 1.21 (0.91-1.57) | -1.01  (-1.21--0.81) |  | 1519.68 (1150.63-1933.77) | 23.97 (18.13-30.49) | -0.79 (-1.00--0.59) |
| Suriname | 45.44  (37.38-54.87) | 7.48 (6.15-8.93) | 0.55 (0.44-0.66) |  | 5.25  (4.42-6.37) | 0.87 (0.73-1.06) | 0.65 (0.51-0.79) |  | 3.06 (2.20-4.02) | 0.51 (0.37-0.67) | -0.65  (-0.79--0.51) |  | 70.41  (51.64-90.08) | 11.26 (8.22-14.32) | -0.86 (-1.03--0.69) |
| Ecuador | 1391.04 (1240.05-1548.85) | 8.47 (7.56-9.42) | 0.56 (0.31-0.80) |  | 251.97 (229.05-277.01) | 1.52 (1.39-1.67) | 2.32 (2.16-2.48) |  | 219.94 (177.95-270.45) | 1.46 (1.18-1.78) | -0.85  (-1.13--0.57) |  | 4187.11 (3384.86-5174.43) | 26.09 (21.18-32.10) | -0.67 (-1.02--0.31) |
| Bahamas | 55.89  (46.76-67.10) | 13.84 (11.68-16.28) | 0.26 (0.22-0.30) |  | 7.89  (7.01-8.84) | 1.94 (1.73-2.17) | 0.47 (0.42-0.52) |  | 4.75 (3.92-5.77) | 1.31 (1.09-1.58) | -1.14  (-1.21--1.07) |  | 113.13  (92.29-138.91) | 28.07 (23.05-34.19) | -1.34 (-1.47--1.21) |
| Libya | 568.77 (460.66-696.29) | 10.06 (8.38-11.90) | 0.68 (0.51-0.85) |  | 62.24 (51.65-75.61) | 1.08 (0.93-1.26) | 0.80 (0.62-0.98) |  | 23.54 (14.74-34.76) | 0.54 (0.33-0.80) | -1.49  (-1.71--1.27) |  | 569.29 (379.35-815.28) | 10.87 (7.06-15.75) | -1.16 (-1.45--0.87) |
| Honduras | 1362.52 (1151.77-1597.22) | 21.10 (17.71-24.51) | 1.06 (1.01-1.10) |  | 200.41 (180.55-225.68) | 2.99 (2.74-3.27) | 1.93 (1.89-1.98) |  | 152.81 (97.63-222.45) | 2.86 (1.84-4.13) | 0.31 (0.20-0.41) |  | 3474.38 (2222.26-5076.67) | 55.39 (35.26-80.96) | -0.30 (-0.42--0.17) |
| Bahrain | 187.33 (152.36-226.88) | 21.16 (17.48-24.59) | -0.30 (-0.51--0.10) |  | 22.03 (18.69-26.35) | 2.71 (2.43-2.99) | -0.12 (-0.41-0.18) |  | 2.62 (2.02-3.41) | 0.56 (0.42-0.75) | -2.96  (-3.30--2.61) |  | 69.55  (54.77-88.36) | 9.85 (7.73-12.84) | -4.07 (-4.40--3.75) |
| Palestine | 315.59 (258.92-375.72) | 11.16 (9.29-13.11) | 1.39 (1.32-1.47) |  | 36.39 (30.84-44.02) | 1.24 (1.09-1.42) | 1.59 (1.52-1.66) |  | 10.48 (8.03-12.91) | 0.54 (0.39-0.67) | -1.86  (-2.04--1.68) |  | 252.87 (197.03-308.57) | 10.31 (7.99-12.66) | -2.46 (-2.53--2.38) |
| Brazil | 25275.78 (21782.96-29094.05) | 10.34 (8.95-11.93) | 0.39 (0.04-0.75) |  | 4056.08 (3674.58-4427.98) | 1.65 (1.50-1.81) | -0.65 (-0.82--0.47) |  | 4368.05 (3959.04-4652.73) | 1.79 (1.62-1.91) | -1.68  (-1.78--1.59) |  | 97444.14 (90862.85-103302.15) | 39.02 (36.29-41.37) | -1.88 (-1.96--1.80) |
| India | 103933.81 (86902.71-124736.26) | 8.13 (6.93-9.53) | 0.33 (0.22-0.45) |  | 10284.57 (8599.65-12558.81) | 0.78 (0.66-0.93) | 0.29 (0.15-0.43) |  | 6703.23 (4826.69-9412.49) | 0.66 (0.48-0.92) | -0.82  (-0.94--0.70) |  | 148144.62 (107938.46-207457.74) | 12.87 (9.38-17.95) | -1.51 (-1.62--1.41) |
| Iran (Islamic Republic of) | 6992.05 (5750.60-8429.65) | 8.68 (7.23-10.20) | -0.31 (-0.39--0.24) |  | 759.29 (638.17-914.47) | 0.94 (0.80-1.10) | -0.39 (-0.47--0.32) |  | 266.85 (223.26-327.63) | 0.40 (0.33-0.49) | -1.36  (-1.44--1.28) |  | 5628.70 (4763.93-6805.86) | 7.55 (6.39-9.10) | -1.60 (-1.71--1.48) |
| Qatar | 624.53 (476.11-793.69) | 34.01 (29.31-39.46) | 2.02 (1.83-2.20) |  | 65.48 (50.34-83.54) | 3.51 (3.05-3.99) | 2.17 (2.01-2.33) |  | 1.67 (1.21-2.23) | 0.32 (0.22-0.43) | -2.15  (-2.57--1.73) |  | 70.78  (54.71-92.20) | 6.60 (4.87-8.40) | -2.55 (-3.04--2.06) |
| United Arab Emirates | 1043.39 (793.79-1354.23) | 14.26 (11.97-16.70) | 0.62 (0.44-0.80) |  | 112.39 (86.30-146.21) | 1.50 (1.30-1.74) | 0.74 (0.55-0.92) |  | 16.37 (11.80-21.71) | 1.08 (0.72-1.47) | 0.16  (-0.32-0.65) |  | 519.91 (392.89-681.62) | 19.48 (13.54-26.88) | 0.35 (-0.22-0.93) |
| Jordan | 952.05 (783.85-1140.00) | 11.80 (9.66-13.83) | 1.33 (1.26-1.40) |  | 112.23 (95.95-131.89) | 1.38 (1.23-1.55) | 1.19 (1.12-1.26) |  | 42.96 (32.31-55.97) | 0.74 (0.55-0.96) | -0.83  (-1.01--0.66) |  | 1071.47 (823.70-1363.13) | 14.51 (11.06-18.65) | -1.16 (-1.43--0.89) |
| Equatorial Guinea | 79.41  (65.77-95.36) | 11.59 (9.75-13.57) | 2.89 (2.63-3.15) |  | 10.60  (8.89-13.22) | 1.43 (1.28-1.61) | 3.75 (3.46-4.04) |  | 3.88 (2.16-6.05) | 0.94 (0.48-1.51) | -0.75  (-0.85--0.65) |  | 112.16  (69.45-170.06) | 19.13 (10.80-29.63) | -1.25 (-1.47--1.04) |
| Pakistan | 10905.42 (8955.66-13302.80) | 7.17 (6.19-8.30) | -0.42 (-0.53--0.31) |  | 1105.01 (910.62-1376.06) | 0.66 (0.57-0.77) | -0.63 (-0.75--0.50) |  | 1048.68 (590.48-1481.64) | 1.15 (0.68-1.60) | 0.28 (0.10-0.45) |  | 24745.07 (13941.30-35124.68) | 21.10 (11.90-29.65) | -0.47 (-0.61--0.32) |
| Algeria | 4046.90 (3329.39-4843.70) | 10.90 (9.09-12.92) | 1.26 (1.23-1.29) |  | 444.77 (376.18-528.59) | 1.18 (1.02-1.37) | 1.44 (1.40-1.49) |  | 107.72 (78.11-152.23) | 0.39 (0.27-0.54) | -2.42  (-2.63--2.22) |  | 2377.93 (1725.39-3249.92) | 7.11 (5.19-9.77) | -2.17 (-2.32--2.01) |
| Syrian Arab Republic | 1113.14 (886.76-1377.48) | 8.33 (6.82-10.06) | 1.38 (1.23-1.53) |  | 120.80 (97.71-146.46) | 0.90 (0.75-1.07) | 1.54 (1.36-1.73) |  | 49.93 (35.46-68.92) | 0.49 (0.36-0.66) | -1.15  (-1.44--0.86) |  | 1130.23 (810.85-1543.43) | 9.24 (6.62-12.76) | -2.14 (-2.44--1.84) |
| Lebanon | 800.29 (662.39-938.84) | 13.24 (10.91-15.58) | 1.84 (1.78-1.90) |  | 91.32 (80.63-104.84) | 1.51 (1.33-1.74) | 2.10 (2.04-2.17) |  | 67.78 (50.83-86.96) | 1.03 (0.77-1.31) | -1.57  (-1.64--1.49) |  | 1158.32 (891.89-1469.47) | 18.48 (14.39-23.39) | -1.96 (-2.05--1.86) |
| Afghanistan | 838.62 (663.53-1034.60) | 5.76 (4.90-6.70) | 0.68 (0.54-0.81) |  | 92.06 (72.87-120.73) | 0.55 (0.47-0.65) | 0.88 (0.72-1.04) |  | 58.85 (29.92-97.76) | 0.71 (0.37-1.17) | -2.03  (-2.12--1.93) |  | 1689.30 (792.06-2832.82) | 14.99 (7.49-24.53) | -2.37 (-2.52--2.22) |
| Egypt | 6900.61 (5683.11-8354.97) | 9.71 (8.12-11.55) | 1.65 (1.59-1.71) |  | 760.81 (629.18-930.99) | 1.03 (0.88-1.21) | 1.79 (1.70-1.87) |  | 315.27 (213.98-427.68) | 0.71 (0.48-0.97) | -1.07  (-1.36--0.78) |  | 7748.71 (5435.50-10406.53) | 13.53 (9.35-18.47) | -1.66 (-2.10--1.22) |
| Bhutan | 74.98  (63.03-89.40) | 11.59 (9.77-13.63) | 2.08 (2.01-2.15) |  | 8.63  (7.44-9.93) | 1.33 (1.16-1.51) | 2.64 (2.57-2.71) |  | 5.74 (3.72-8.69) | 1.05 (0.68-1.58) | -0.53  (-0.59--0.47) |  | 114.82  (73.00-177.41) | 19.28 (12.33-29.49) | -1.04 (-1.15--0.94) |
| Türkiye | 10444.49 (8406.22-12556.10) | 11.29 (9.19-13.50) | 0.96 (0.64-1.29) |  | 1352.25 (1204.24-1530.91) | 1.47 (1.32-1.66) | 2.01 (1.94-2.08) |  | 12420.18 (9647.46-15848.74) | 0.72 (0.56-0.94) | -1.46  (-1.57--1.34) |  | 616.15 (474.87-795.79) | 13.74 (10.73-17.50) | -2.20 (-2.34--2.06) |
| Morocco | 3108.65 (2558.00-3650.11) | 9.01 (7.53-10.51) | 1.37 (1.32-1.41) |  | 326.68 (277.35-386.89) | 0.94 (0.81-1.09) | 1.50 (1.46-1.54) |  | 133.56 (92.91-185.09) | 0.45 (0.31-0.63) | -1.57  (-1.66--1.49) |  | 2965.43 (2040.44-4088.41) | 8.96 (6.23-12.33) | -1.64 (-1.73--1.54) |
| Iraq | 2477.98 (1993.03-3025.56) | 8.33 (6.91-9.83) | 1.51 (1.40-1.62) |  | 278.15 (223.74-349.79) | 0.89 (0.74-1.07) | 1.62 (1.51-1.74) |  | 44.82 (32.72-59.04) | 0.22 (0.16-0.29) | -0.56  (-0.68--0.44) |  | 1269.85 (951.39-1668.49) | 4.96 (3.70-6.49) | -1.09 (-1.18--1.00) |
| Central African Republic | 156.88 (132.54-185.85) | 5.91 (5.04-6.94) | 0.04 (-0.02-0.09) |  | 18.92 (16.08-23.16) | 0.62 (0.55-0.69) | 0.06 (0.02-0.11) |  | 17.64 (11.03-25.11) | 1.02 (0.66-1.42) | 0.41 (0.35-0.47) |  | 574.31 (357.50-856.54) | 22.61 (14.30-31.98) | 0.23 (0.14-0.32) |
| Paraguay | 569.18 (480.21-671.22) | 9.24 (7.81-10.94) | 0.99 (0.95-1.03) |  | 103.12 (90.92-117.08) | 1.67 (1.49-1.86) | 1.54 (1.48-1.60) |  | 114.34 (85.65-153.28) | 2.10 (1.57-2.80) | 0.37 (0.26-0.48) |  | 2460.29 (1869.08-3317.19) | 42.21 (31.95-56.67) | 0.34 (0.22-0.46) |
| Oman | 399.61 (315.52-494.91) | 15.14 (12.48-17.90) | 1.86 (1.80-1.92) |  | 45.56 (36.11-56.89) | 1.68 (1.47-1.92) | 2.04 (1.98-2.11) |  | 3.99 (2.84-5.28) | 0.26 (0.18-0.34) | 0.32  (-0.11-0.76) |  | 123.02  (91.45-167.55) | 5.52 (3.98-7.07) | 0.99 (0.55-1.43) |
| Comoros | 23.36  (19.57-27.80) | 4.87 (4.10-5.73) | 0.30 (0.26-0.35) |  | 3.43  (3.04-3.88) | 0.67 (0.60-0.75) | 0.93 (0.82-1.05) |  | 2.30 (1.26-3.89) | 0.56 (0.31-0.93) | -0.55  (-0.60--0.49) |  | 57.15  (30.83-92.31) | 11.52 (6.32-18.72) | -0.85 (-0.97--0.74) |
| Yemen | 1216.08 (988.80-1487.82) | 6.66 (5.58-7.80) | 0.88 (0.72-1.04) |  | 133.48 (108.37-168.90) | 0.67 (0.57-0.78) | 1.02 (0.83-1.21) |  | 50.38 (31.95-77.21) | 0.45 (0.28-0.68) | -1.88  (-2.01--1.75) |  | 1295.81 (840.31-1928.53) | 8.88 (5.65-13.37) | -2.13 (-2.30--1.95) |
| Ethiopia | 2346.85 (1963.26-2755.88) | 5.02 (4.26-5.86) | 0.63 (0.49-0.78) |  | 259.79 (223.86-308.99) | 0.49 (0.44-0.55) | 1.05 (0.89-1.22) |  | 170.59 (89.93-253.27) | 0.46 (0.25-0.70) | -1.47  (-1.64--1.30) |  | 4366.12 (2242.79-6555.27) | 9.27 (4.92-13.75) | -2.22 (-2.41--2.03) |
| Nepal | 2099.67 (1739.99-2481.69) | 8.88 (7.48-10.34) | 1.23 (1.20-1.26) |  | 254.82 (223.05-292.57) | 1.06 (0.94-1.19) | 1.90 (1.87-1.93) |  | 184.41 (117.82-268.44) | 0.98 (0.62-1.42) | -0.72  (-0.83--0.62) |  | 4034.55 (2643.99-5793.39) | 18.25 (11.79-26.01) | -1.04 (-1.24--0.84) |
| Democratic Republic of the Congo | 3173.58 (2631.99-3789.87) | 7.27 (6.14-8.67) | -0.26 (-0.51--0.00) |  | 441.39 (378.88-530.96) | 0.92 (0.82-1.02) | 0.09 (-0.24-0.42) |  | 250.33 (158.18-363.61) | 0.87 (0.55-1.27) | -0.40  (-0.46--0.34) |  | 7386.96 (4829.61-11338.34) | 18.22 (11.67-26.23) | -0.30 (-0.37--0.22) |
| Saudi Arabia | 3559.12 (2811.84-4419.81) | 14.79 (12.22-17.69) | 1.56 (1.52-1.60) |  | 399.96 (324.30-501.19) | 1.62 (1.42-1.88) | 1.80 (1.76-1.83) |  | 78.79 (52.45-116.98) | 0.55 (0.39-0.80) | -1.88  (-2.01--1.75) |  | 2519.08 (1671.55-3773.68) | 11.40 (8.04-16.36) | -2.09 (-2.20--1.99) |
| Kuwait | 647.93 (530.94-789.12) | 20.18 (16.94-24.06) | 1.52 (1.44-1.61) |  | 75.88 (63.69-90.17) | 2.40 (2.13-2.70) | 1.67 (1.57-1.78) |  | 23.56 (18.99-28.50) | 1.03 (0.82-1.24) | -1.92  (-2.69--1.15) |  | 575.26 (467.22-696.76) | 19.71 (15.96-24.11) | -1.04 (-2.26-0.19) |
| Madagascar | 435.55 (360.16-525.92) | 3.74 (3.17-4.43) | 0.70 (0.67-0.73) |  | 63.68 (53.22-75.40) | 0.48 (0.43-0.54) | 1.25 (1.20-1.29) |  | 63.03 (38.50-113.80) | 0.73 (0.45-1.29) | -0.17  (-0.23--0.11) |  | 1920.85 (1133.74-3447.47) | 15.28 (9.43-27.39) | -0.34 (-0.38--0.31) |
| Gabon | 118.24  (97.67-140.09) | 10.29 (8.48-12.07) | 0.75 (0.71-0.79) |  | 15.23 (13.29-17.94) | 1.26 (1.13-1.40) | 1.16 (1.12-1.19) |  | 7.83 (4.52-12.13) | 0.97 (0.55-1.56) | 0.77 (0.67-0.86) |  | 195.92 (123.02-291.12) | 19.07 (11.36-29.18) | 0.48 (0.37-0.58) |
| Angola | 1171.13 (963.34-1423.09) | 7.53 (6.34-8.70) | 1.27 (1.13-1.40) |  | 148.62 (122.74-188.12) | 0.84 (0.74-0.95) | 1.66 (1.49-1.84) |  | 65.70 (35.62-93.78) | 0.72 (0.41-1.04) | -0.08  (-0.13--0.02) |  | 1983.99 (1063.88-2907.69) | 14.98 (8.33-21.25) | -0.22 (-0.31--0.13) |
| Bangladesh | 14046.83 (11607.27-16382.79) | 9.69 (8.12-11.18) | 1.34 (1.30-1.39) |  | 1736.35 (1512.30-1991.96) | 1.19 (1.05-1.34) | 2.09 (2.02-2.15) |  | 865.21 (578.23-1192.41) | 0.73 (0.49-1.01) | -1.17  (-1.45--0.90) |  | 18605.60 (12512.81-25527.08) | 13.90 (9.40-18.90) | -2.13 (-2.29--1.98) |
| Djibouti | 29.71  (24.69-35.44) | 4.65 (3.96-5.45) | 0.50 (0.42-0.58) |  | 4.59  (3.95-5.31) | 0.65 (0.59-0.73) | 1.09 (0.95-1.23) |  | 3.49 (2.23-5.11) | 0.74 (0.49-1.06) | 0.09  (-0.06-0.24) |  | 98.87  (61.07-148.27) | 15.10 (9.82-21.64) | -0.50 (-0.64--0.36) |
| Tunisia | 1444.65 (1189.75-1720.58) | 11.13 (9.20-13.13) | 1.66 (1.59-1.73) |  | 157.87 (136.03-185.05) | 1.22 (1.06-1.43) | 1.80 (1.72-1.88) |  | 41.33 (26.01-59.53) | 0.35 (0.22-0.51) | -2.06  (-2.19--1.92) |  | 850.67 (556.89-1206.97) | 6.73 (4.40-9.58) | -1.91 (-2.05--1.78) |
| Burundi | 197.16 (168.30-234.05) | 3.88 (3.34-4.60) | 0.49 (0.42-0.57) |  | 29.39 (25.39-33.96) | 0.52 (0.46-0.57) | 1.19 (1.08-1.30) |  | 16.18 (5.10-28.56) | 0.42 (0.14-0.76) | 3.78 (3.21-4.36) |  | 460.06 (149.71-808.09) | 8.63 (2.86-15.14) | 2.16 (1.50-2.83) |
| Mauritius | 102.42  (83.72-121.60) | 6.10 (5.05-7.17) | 0.73 (0.62-0.84) |  | 11.74 (10.04-13.74) | 0.71 (0.62-0.82) | 0.92 (0.79-1.05) |  | 4.77 (4.24-5.23) | 0.29 (0.26-0.32) | -1.17  (-1.77--0.57) |  | 95.82  (86.53-104.26) | 5.73 (5.21-6.23) | 0.38 (-0.46-1.22) |
| United Republic of Tanzania | 1295.37 (1074.53-1543.72) | 4.78 (3.99-5.69) | 1.06 (1.03-1.09) |  | 199.17 (172.71-230.93) | 0.68 (0.61-0.75) | 1.66 (1.63-1.70) |  | 135.94 (60.69-236.07) | 0.61 (0.29-1.05) | 0.43 (0.32-0.54) |  | 3748.02 (1585.47-6775.74) | 12.94 (5.82-22.34) | 0.32 (0.15-0.49) |
| South Africa | 5519.71 (4517.53-6753.10) | 10.47 (8.72-12.51) | 0.20 (0.08-0.32) |  | 596.17 (497.60-737.07) | 1.11 (0.94-1.34) | 0.13 (-0.02-0.29) |  | 328.14 (244.90-372.10) | 0.80 (0.57-0.91) | 0.45 (0.25-0.65) |  | 8887.74 (7246.11-9995.74) | 18.22 (14.34-20.48) | 0.51 (0.17-0.85) |
| Congo | 258.48 (214.33-311.01) | 8.43 (7.05-9.92) | 0.67 (0.64-0.69) |  | 32.30 (27.49-39.16) | 0.98 (0.87-1.09) | 1.02 (0.95-1.09) |  | 20.74 (12.10-29.27) | 1.01 (0.59-1.46) | 0.15 (0.10-0.20) |  | 580.25 (356.62-808.47) | 20.74 (12.37-29.18) | -0.17 (-0.23--0.11) |
| Rwanda | 277.14 (233.26-333.82) | 4.42 (3.72-5.18) | 0.48 (0.33-0.63) |  | 42.57 (37.00-48.85) | 0.62 (0.55-0.69) | 1.50 (1.28-1.71) |  | 14.47 (3.09-41.04) | 0.29 (0.06-0.86) | -0.73  (-1.28--0.18) |  | 396.39  (91.17-1048.35) | 6.02 (1.42-16.67) | -2.73 (-3.23--2.23) |
| Côte d'Ivoire | 901.09 (727.27-1099.53) | 5.26 (4.33-6.14) | 0.57 (0.51-0.64) |  | 132.22 (107.17-168.47) | 0.65 (0.56-0.77) | 0.73 (0.65-0.81) |  | 120.06 (77.85-179.34) | 0.97 (0.63-1.48) | 0.32 (0.09-0.56) |  | 4844.89 (3073.06-6907.35) | 26.88 (17.53-40.07) | -0.29 (-0.49--0.09) |
| Zambia | 371.30 (314.84-442.84) | 4.97 (4.25-5.96) | 0.54 (0.49-0.60) |  | 58.63 (50.30-68.91) | 0.70 (0.63-0.77) | 1.25 (1.12-1.39) |  | 46.52 (30.60-65.52) | 0.79 (0.51-1.13) | 0.24  (-0.00-0.49) |  | 1397.89 (921.04-2000.70) | 17.06 (11.32-23.90) | -0.62 (-0.85--0.39) |
| Mauritania | 160.71 (131.96-195.45) | 5.56 (4.65-6.61) | 0.65 (0.59-0.72) |  | 22.75 (18.38-29.44) | 0.70 (0.60-0.84) | 0.95 (0.87-1.03) |  | 16.94 (10.99-24.38) | 0.75 (0.50-1.10) | -0.31  (-0.41--0.22) |  | 567.82 (367.10-801.09) | 19.47 (12.73-27.78) | -0.70 (-0.77--0.63) |
| Zimbabwe | 814.96 (635.20-1064.01) | 6.96 (5.70-8.48) | -0.47 (-0.58--0.36) |  | 91.51 (70.04-122.61) | 0.70 (0.57-0.88) | -0.66 (-0.79--0.54) |  | 46.17 (31.84-64.95) | 0.75 (0.52-1.03) | 1.20 (0.91-1.49) |  | 1522.24 (1042.52-2175.14) | 17.87 (12.40-25.12) | 1.42 (0.88-1.98) |
| Kenya | 1696.17 (1430.02-2029.38) | 7.45 (6.30-8.89) | 0.79 (0.68-0.90) |  | 211.11 (183.73-242.55) | 0.85 (0.76-0.94) | 0.48 (0.45-0.51) |  | 133.05 (66.15-223.77) | 0.77 (0.38-1.31) | 0.65 (0.55-0.75) |  | 3382.46 (1690.65-5596.30) | 14.85 (7.47-24.91) | 0.78 (0.58-0.97) |
| Eritrea | 119.26 (100.44-143.60) | 4.38 (3.75-5.09) | 1.00 (0.85-1.15) |  | 17.17 (14.93-19.95) | 0.55 (0.49-0.60) | 1.51 (1.36-1.66) |  | 17.10 (9.79-24.20) | 0.81 (0.48-1.14) | -0.12  (-0.23--0.00) |  | 521.51 (296.81-773.59) | 17.02 (9.86-24.02) | -0.73 (-0.82--0.65) |
| Nigeria | 10324.12 (8357.13-12777.29) | 7.34 (6.19-8.64) | 0.48 (0.38-0.58) |  | 1382.82 (1131.26-1753.24) | 0.85 (0.73-1.00) | 0.18 (0.09-0.28) |  | 752.00 (491.91-1044.28) | 0.74 (0.52-0.98) | 0.38 (0.24-0.52) |  | 30901.86 (18598.03-44403.39) | 19.84 (13.00-27.55) | -0.03 (-0.16-0.10) |
| Ghana | 1369.20 (1115.66-1684.68) | 5.92 (4.86-6.98) | 0.63 (0.52-0.74) |  | 208.00 (171.40-263.29) | 0.77 (0.67-0.92) | 0.61 (0.45-0.78) |  | 152.98 (104.19-221.14) | 0.92 (0.62-1.31) | 0.65 (0.60-0.69) |  | 5517.86 (3829.37-7851.87) | 23.65 (16.20-34.28) | 0.34 (0.24-0.44) |
| Botswana | 254.68 (207.64-303.66) | 13.12 (11.02-15.21) | 0.95 (0.78-1.12) |  | 28.79 (24.10-35.35) | 1.43 (1.24-1.68) | 0.98 (0.86-1.10) |  | 6.61 (4.44-9.07) | 0.53 (0.35-0.73) | -0.38  (-0.55--0.21) |  | 198.83 (132.86-273.90) | 12.03 (8.20-16.46) | -0.97 (-1.21--0.73) |
| Benin | 402.19 (328.09-492.47) | 4.92 (4.13-5.80) | 0.89 (0.86-0.93) |  | 60.93 (49.16-78.11) | 0.61 (0.53-0.73) | 0.96 (0.92-1.00) |  | 50.43 (35.94-70.98) | 0.83 (0.59-1.15) | -0.22  (-0.28--0.16) |  | 2047.29 (1397.13-2945.53) | 23.10 (16.37-32.71) | -0.37 (-0.47--0.27) |
| Namibia | 216.81 (177.58-262.31) | 11.42 (9.72-13.16) | 1.00 (0.92-1.08) |  | 24.24 (19.92-30.32) | 1.21 (1.04-1.43) | 1.10 (1.02-1.18) |  | 9.22 (5.91-13.51) | 0.78 (0.49-1.14) | 0.22  (-0.01-0.44) |  | 269.11 (169.06-405.73) | 17.51 (11.19-25.59) | -0.41 (-0.74--0.08) |
| Cameroon | 1010.28 (825.06-1245.54) | 5.37 (4.46-6.36) | 0.90 (0.85-0.94) |  | 150.68 (122.33-195.07) | 0.68 (0.59-0.81) | 1.15 (1.06-1.24) |  | 117.57 (75.64-165.73) | 0.83 (0.56-1.18) | 0.31 (0.15-0.46) |  | 4726.14 (2928.33-6628.41) | 23.01 (14.88-32.40) | -0.09 (-0.25-0.06) |
| Somalia | 162.87 (134.20-196.26) | 2.50 (2.16-2.91) | -0.09 (-0.11--0.08) |  | 22.42 (18.42-27.41) | 0.28 (0.25-0.32) | 0.08 (0.06-0.10) |  | 38.03 (22.58-55.49) | 0.77 (0.47-1.08) | -0.35  (-0.40--0.29) |  | 1247.69 (740.52-1833.93) | 16.71 (10.17-24.44) | -0.61 (-0.68--0.53) |
| Guinea-Bissau | 58.40  (47.65-73.14) | 5.11 (4.31-5.99) | 0.60 (0.55-0.66) |  | 8.59  (7.02-11.07) | 0.62 (0.54-0.72) | 0.66 (0.62-0.70) |  | 11.53 (6.88-18.49) | 1.31 (0.80-2.05) | -0.19  (-0.26--0.12) |  | 501.32 (291.66-803.48) | 38.18 (22.89-61.12) | -0.20 (-0.36--0.05) |
| Mozambique | 621.03 (535.40-735.15) | 5.34 (4.62-6.22) | 1.58 (1.54-1.63) |  | 99.39 (87.65-113.15) | 0.76 (0.69-0.83) | 2.31 (2.24-2.38) |  | 96.17 (57.69-134.76) | 0.97 (0.58-1.35) | 0.62 (0.53-0.70) |  | 3008.45 (1706.23-4485.76) | 22.12 (13.43-30.70) | 0.80 (0.64-0.95) |
| Uganda | 775.87 (661.89-916.28) | 4.95 (4.21-5.75) | 0.93 (0.89-0.97) |  | 125.59 (109.45-146.01) | 0.73 (0.65-0.81) | 1.61 (1.50-1.73) |  | 49.36 (22.24-75.91) | 0.41 (0.19-0.64) | -0.11  (-0.26-0.04) |  | 1404.62 (607.70-2147.33) | 8.30 (3.88-12.73) | -0.57 (-0.77--0.37) |
| Malawi | 333.32 (282.50-395.45) | 4.31 (3.68-5.07) | 0.82 (0.78-0.86) |  | 51.80 (44.82-60.29) | 0.61 (0.54-0.67) | 1.56 (1.47-1.65) |  | 43.33 (23.35-63.60) | 0.68 (0.38-1.01) | 0.04  (-0.14-0.22) |  | 1261.11 (653.65-1894.75) | 14.81 (8.01-21.65) | -0.59 (-0.82--0.36) |
| Eswatini | 84.63  (68.45-106.90) | 9.44 (8.07-11.19) | 0.82 (0.80-0.85) |  | 9.50  (7.58-12.36) | 0.98 (0.82-1.19) | 0.77 (0.73-0.81) |  | 4.68 (3.20-6.65) | 0.92 (0.63-1.29) | 0.74 (0.46-1.02) |  | 155.98 (105.45-219.91) | 22.47 (15.66-31.91) | 0.51 (-0.01-1.03) |
| Chad | 474.56 (385.36-580.43) | 4.89 (4.13-5.70) | 0.79 (0.77-0.81) |  | 71.70 (57.64-91.01) | 0.58 (0.51-0.68) | 0.76 (0.71-0.80) |  | 65.76 (44.99-98.80) | 0.94 (0.64-1.43) | 1.04 (0.92-1.16) |  | 2771.16 (1888.87-4103.17) | 26.21 (17.97-40.24) | 0.88 (0.71-1.05) |
| Mali | 696.62 (555.06-860.08) | 4.86 (4.02-5.70) | 1.17 (1.12-1.21) |  | 110.16 (88.17-140.30) | 0.61 (0.52-0.72) | 1.19 (1.17-1.22) |  | 101.95 (73.08-153.63) | 1.00 (0.71-1.50) | -0.25  (-0.37--0.13) |  | 4138.38 (2897.01-6029.41) | 27.75 (19.72-41.85) | -0.04 (-0.15-0.07) |
| Senegal | 576.00 (470.36-697.26) | 5.55 (4.62-6.42) | 0.70 (0.66-0.75) |  | 81.00 (66.71-103.64) | 0.69 (0.59-0.81) | 0.73 (0.67-0.79) |  | 82.29 (50.79-120.14) | 0.99 (0.60-1.44) | -0.57  (-0.67--0.48) |  | 2942.75 (1857.06-4214.68) | 26.83 (16.72-38.70) | -0.85 (-0.96--0.75) |
| Burkina Faso | 680.77 (560.33-830.77) | 5.03 (4.25-5.91) | 1.14 (1.08-1.19) |  | 104.56 (86.16-131.43) | 0.63 (0.55-0.74) | 1.24 (1.17-1.31) |  | 70.89 (43.22-110.24) | 0.65 (0.41-0.96) | -0.04  (-0.13-0.06) |  | 2880.95 (1630.96-4989.82) | 18.35 (11.19-28.48) | 0.12 (-0.05-0.29) |
| Gambia | 73.25  (59.84-89.88) | 5.02 (4.19-5.90) | 0.20 (0.12-0.27) |  | 10.47  (8.42-13.64) | 0.62 (0.53-0.73) | 0.28 (0.22-0.35) |  | 14.11 (8.23-24.89) | 1.24 (0.70-2.20) | 0.53 (0.38-0.67) |  | 547.49 (328.75-939.56) | 34.71 (20.27-60.74) | 0.06 (-0.14-0.26) |
| Lesotho | 113.59  (91.43-141.32) | 7.45 (6.26-8.73) | 0.67 (0.65-0.69) |  | 12.38  (9.86-15.99) | 0.75 (0.63-0.92) | 0.53 (0.50-0.56) |  | 6.93 (4.74-9.49) | 0.71 (0.48-0.97) | 1.35 (1.11-1.60) |  | 217.57 (149.34-305.73) | 17.55 (12.17-24.09) | 1.63 (1.17-2.09) |
| Niger | 617.24 (501.66-758.07) | 4.51 (3.80-5.29) | 0.76 (0.74-0.79) |  | 98.01 (79.35-125.99) | 0.55 (0.48-0.65) | 0.81 (0.76-0.86) |  | 49.96 (17.02-109.17) | 0.50 (0.17-1.08) | -1.15  (-1.30--1.01) |  | 2089.33 (723.87-4638.88) | 13.81 (4.76-29.49) | -1.42 (-1.69--1.16) |
| Guinea | 387.99 (316.46-483.80) | 4.50 (3.79-5.27) | 0.46 (0.40-0.51) |  | 56.76 (45.83-72.47) | 0.54 (0.46-0.65) | 0.37 (0.30-0.44) |  | 56.22 (39.11-79.66) | 0.88 (0.61-1.25) | 0.48 (0.40-0.57) |  | 2163.80 (1484.50-3027.54) | 24.34 (16.99-34.43) | 0.34 (0.21-0.46) |
| Sao Tome and Principe | 7.72  (6.24-9.42) | 5.24 (4.38-6.21) | 0.81 (0.75-0.87) |  | 1.10  (0.89-1.42) | 0.68 (0.57-0.81) | 0.90 (0.85-0.95) |  | 0.95 (0.51-1.45) | 0.88 (0.48-1.38) | 0.53 (0.43-0.63) |  | 31.41  (17.56-48.58) | 22.06 (12.21-34.12) | -0.04 (-0.19-0.11) |
| Cabo Verde | 31.01  (25.63-36.88) | 6.44 (5.37-7.53) | 1.60 (1.52-1.68) |  | 4.31  (3.62-5.20) | 0.88 (0.75-1.03) | 1.89 (1.81-1.97) |  | 2.65 (1.79-3.59) | 0.64 (0.42-0.88) | 0.57 (0.35-0.79) |  | 77.25  (53.58-103.24) | 16.30 (11.12-21.75) | 0.00 (-0.26-0.27) |
| Liberia | 149.20 (119.21-183.84) | 4.66 (3.88-5.43) | 0.97 (0.89-1.05) |  | 22.08 (17.80-28.63) | 0.58 (0.50-0.69) | 1.10 (0.96-1.23) |  | 21.46 (13.84-32.00) | 0.91 (0.59-1.33) | 0.08 (-0.02-0.17) |  | 867.48 (555.68-1270.89) | 24.91 (16.12-37.10) | -0.21 (-0.34--0.07) |
| Seychelles | 8.07  (6.53-9.61) | 7.24 (5.91-8.60) | 0.89 (0.74-1.04) |  | 0.95  (0.83-1.11) | 0.86 (0.76-1.00) | 1.24 (1.07-1.42) |  | 0.14 (0.10-0.34) | 0.16 (0.11-0.38) | 0.31 (-0.05-0.66) |  | 2.78  (2.08-5.63) | 2.72 (2.02-5.79) | -0.95 (-1.17--0.73) |
| Sierra Leone | 253.55 (208.81-309.96) | 4.49 (3.80-5.23) | 0.85 (0.74-0.95) |  | 37.59 (30.50-47.86) | 0.56 (0.48-0.66) | 0.80 (0.63-0.97) |  | 37.24 (26.30-51.40) | 0.83 (0.59-1.17) | -0.07 (-0.13--0.01) |  | 1474.23 (1026.47-2048.15) | 23.75 (16.88-32.82) | 0.20 (0.12-0.27) |
| Togo | 276.24 (225.79-337.27) | 5.30 (4.44-6.25) | 0.92 (0.83-1.01) |  | 40.72 (33.23-50.91) | 0.68 (0.59-0.80) | 1.11 (0.95-1.26) |  | 41.67 (28.88-58.49) | 1.00 (0.71-1.38) | 0.12 (-0.02-0.26) |  | 1629.65 (1128.72-2322.96) | 28.38 (19.75-39.89) | -0.26 (-0.38--0.13) |
| Bermuda | 15.86  (12.83-19.32) | 14.19 (11.71-17.17) | 0.24 (0.16-0.31) |  | 2.27  (2.03-2.55) | 2.01 (1.76-2.31) | 0.30 (0.15-0.45) |  | 1.01 (0.82-1.26) | 0.66 (0.54-0.82) | -3.38 (-3.66--3.10) |  | 16.37  (13.57-20.33) | 11.93 (9.99-14.77) | -3.76 (-4.10--3.43) |
| Greenland | 38.45  (33.17-43.55) | 57.57 (50.06-64.69) | 0.79 (0.77-0.80) |  | 3.91  (3.58-4.25) | 5.83 (5.35-6.32) | 0.95 (0.94-0.97) |  | 2.13 (1.23-2.69) | 3.63 (1.98-4.64) | -1.07 (-1.18--0.97) |  | 51.25  (32.34-63.73) | 74.88 (46.05-93.69) | -0.94 (-1.06--0.81) |
| Nauru | 0.62 (0.51-0.76) | 9.00 (7.44-10.64) | 0.55 (0.34-0.77) |  | 0.08 (0.07-0.10) | 1.12 (1.00-1.27) | 0.83 (0.57-1.10) |  | 0.02 (0.01-0.02) | 0.22 (0.11-0.34) | 0.92 (0.74-1.09) |  | 0.67 (0.36-1.00) | 7.74 (4.22-11.33) | 0.92 (0.63-1.21) |
| American Samoa | 3.30 (2.69-3.94) | 6.79 (5.65-8.06) | 0.23 (0.17-0.30) |  | 0.38 (0.33-0.45) | 0.78 (0.68-0.91) | 0.32 (0.25-0.39) |  | 0.09 (0.06-0.12) | 0.21 (0.14-0.27) | 4.39 (3.57-5.21) |  | 3.46 (2.50-4.53) | 7.16 (5.20-9.46) | 6.40 (5.52-7.28) |
| Tokelau | 0.10 (0.08-0.12) | 6.90 (5.68-8.24) | 1.40 (1.37-1.43) |  | 0.01  (0.01-0.01) | 0.81 (0.70-0.95) | 1.70 (1.67-1.72) |  | 0.00 (0.00-0.00) | 0.15 (0.11-0.21) | -0.11 (-0.21--0.01) |  | 0.08 (0.06-0.13) | 5.94 (4.28-8.83) | 0.42 (0.12-0.72) |
| Northern Mariana Islands | 6.17 (5.12-7.42) | 11.74 (9.96-13.78) | -0.16 (-0.28--0.04) |  | 0.83  (0.73-0.96) | 1.60 (1.43-1.81) | 0.02 (-0.12-0.16) |  | 0.12 (0.07-0.15) | 0.29 (0.16-0.37) | 2.07 (1.32-2.82) |  | 3.79 (2.60-4.63) | 7.64 (5.12-9.34) | 3.68 (2.79-4.58) |
| United States Virgin Islands | 19.46 (15.61-23.49) | 13.03 (10.69-15.49) | 0.63 (0.51-0.74) |  | 2.56  (2.32-2.83) | 1.72 (1.51-1.97) | 0.76 (0.65-0.87) |  | 0.98 (0.65-1.31) | 0.57 (0.40-0.76) | -2.32 (-2.44--2.21) |  | 18.65 (12.87-25.30) | 11.67 (8.31-15.48) | -2.38 (-2.53--2.22) |
| Monaco | 20.33 (16.39-24.84) | 23.80 (19.53-28.50) | 0.48 (0.41-0.55) |  | 3.59  (3.29-3.93) | 4.00 (3.63-4.39) | 0.72 (0.60-0.84) |  | 2.53 (1.85-3.20) | 2.11 (1.57-2.67) | -0.37 (-0.45--0.29) |  | 37.00 (27.65-46.39) | 35.67 (27.16-44.66) | -0.36 (-0.47--0.25) |
| Puerto Rico | 701.44 (568.57-855.34) | 12.39 (10.15-14.90) | 0.09 (-0.01-0.18) |  | 91.06 (81.70-101.86) | 1.65 (1.42-1.94) | 0.15 (0.02-0.27) |  | 35.11 (28.65-41.93) | 0.42 (0.35-0.50) | -3.51 (-3.75--3.26) |  | 607.81 (506.26-716.43) | 8.92 (7.44-10.45) | -3.93 (-4.20--3.65) |
| Cook Islands | 3.04 (2.46-3.62) | 12.46 (10.26-14.73) | 1.07 (1.03-1.12) |  | 0.43  (0.39-0.48) | 1.78 (1.60-2.00) | 1.57 (1.52-1.62) |  | 0.04 (0.03-0.05) | 0.17 (0.12-0.24) | -0.03 (-0.14-0.08) |  | 1.36 (0.98-1.90) | 6.65 (4.66-9.51) | 0.61 (0.46-0.76) |
| San Marino | 8.55 (6.71-10.37) | 13.65 (10.96-16.31) | 0.27 (0.07-0.47) |  | 1.21 (1.05-1.39) | 1.92 (1.63-2.25) | 0.41 (0.16-0.67) |  | 0.37 (0.24-0.51) | 0.33 (0.21-0.47) | -1.52 (-1.74--1.30) |  | 4.76 (3.22-6.56) | 5.26 (3.62-7.21) | -1.43 (-1.76--1.11) |
| Niue | 0.20 (0.16-0.24) | 9.51 (7.76-11.32) | 1.21 (1.17-1.25) |  | 0.03 (0.02-0.03) | 1.26 (1.11-1.43) | 1.56 (1.53-1.58) |  | 0.00 (0.00-0.00) | 0.17 (0.12-0.23) | 0.70 (0.60-0.79) |  | 0.11 (0.09-0.16) | 6.57 (4.80-9.21) | 1.03 (0.80-1.27) |
| Sudan | 1785.28 (1436.85-2175.92) | 7.17 (5.93-8.48) | 1.52 (1.47-1.58) |  | 198.03 (162.36-250.20) | 0.74 (0.62-0.87) | 1.67 (1.60-1.74) |  | 84.70 (52.39-149.44) | 0.51 (0.31-0.88) | -1.24 (-1.33--1.14) |  | 2225.18 (1346.43-3879.77) | 10.45 (6.48-18.34) | -1.40 (-1.55--1.26) |
| Guam | 16.49 (13.39-19.63) | 8.48 (6.89-10.04) | 0.42 (0.38-0.47) |  | 1.89 (1.63-2.21) | 0.99 (0.85-1.18) | 0.49 (0.44-0.54) |  | 0.15 (0.13-0.19) | 0.09 (0.07-0.11) | 0.45 (-0.51-1.42) |  | 5.94 (4.89-7.26) | 3.51 (2.87-4.31) | 4.37 (3.21-5.53) |
| Tuvalu | 0.71 (0.59-0.85) | 6.53 (5.44-7.75) | 1.03 (0.97-1.09) |  | 0.09 (0.07-0.10) | 0.77 (0.67-0.90) | 1.35 (1.28-1.42) |  | 0.02 (0.01-0.02) | 0.17 (0.13-0.23) | 0.09 (0.03-0.15) |  | 0.66 (0.47-0.88) | 5.89 (4.25-7.95) | 0.50 (0.40-0.60) |
| Saint Kitts and Nevis | 10.40 (8.39-12.64) | 15.82 (12.99-18.64) | 0.59 (0.53-0.66) |  | 1.66 (1.48-1.84) | 2.50 (2.25-2.77) | 1.11 (0.99-1.23) |  | 0.60 (0.49-0.69) | 1.09 (0.92-1.25) | -1.38 (-1.53--1.23) |  | 13.52 (10.91-15.88) | 20.96 (17.25-24.13) | -1.49 (-1.66--1.33) |
| South Sudan | 160.04 (136.10-190.60) | 4.05 (3.50-4.82) | 0.11 (0.04-0.19) |  | 22.78 (19.50-26.91) | 0.51 (0.45-0.57) | 0.51 (0.46-0.56) |  | 25.11 (12.66-39.36) | 0.77 (0.40-1.23) | -0.01 (-0.11-0.08) |  | 763.40 (390.02-1227.79) | 16.79 (8.56-26.27) | -0.28 (-0.44--0.13) |
| Palau | 2.00 (1.62-2.45) | 9.07 (7.56-10.79) | 0.98 (0.92-1.03) |  | 0.25 (0.22-0.29) | 1.14 (1.01-1.31) | 1.23 (1.17-1.30) |  | 0.03 (0.02-0.04) | 0.17 (0.11-0.23) | 0.54 (0.44-0.64) |  | 1.21 (0.82-1.69) | 5.68 (3.92-7.95) | 1.13 (0.96-1.30) |
|  |  |  |  | | | | | | | | | | | | |

ASR, Age-standardized rates; ASIR,Age-standardized incidence rate; ASPR, Age-standardized prevalence rate; ASMR, Age-standardized mortality rate; ASDR, Age-standardized disability-adjusted life years rate;

EAPC, Estimated annual percentage changes; DALYs, Disability-adjusted life years.
