# Supplementary material for: The global burden of vascular intestinal diseases: results from the 2021 Global Burden of Disease Study and projections using Bayesian age-period-cohort analysis
Source: Environ Health Prev Med. 2024 Dec 11;29:71. doi: 10.1265/ehpm.24-00206 (PMC11653002; doi:10.1265/ehpm.24-00206)
Supplement: Supplementary file 15 — Additional file 15: Table S3 Correlation between SDI and disease burden across regions and countries, and EAPC. [file ehpm-29-071-s015.docx]

| **Table S3 Correlation between SDI and disease burden across regions and countries, and EAPC** | | | | | | | | | | | | | | |  |
| --- | --- | --- | --- | --- | --- | --- | --- | --- | --- | --- | --- | --- | --- | --- | --- |
|  | **Incidence** | | |  | **Prevalence** | | |  | **Deaths** | |  | | **DALYs** | |  |
|  |  |  |  |  |  |  |  |  |  |  |  |  |  |  |  |
|  |  |  |  |  |  |  |  |  |  |  |  |  |  |  |  |
|  |  |  |  |  |  |  |  |  |  |  |  |  |  |  |  |
|  | **R** | **p** |  |  | **R** | **p** |  |  | **R** | **p** |  |  | **R** | **p** |  |
| 21 regions-SDI | 0.821 | <0.001 |  |  | 0.843 | <0.001 |  |  | 0.467 | <0.001 |  |  | 0.393 | <0.001 |  |
| 204 countries-SDI | 0.836 | <0.001 |  |  | 8470 | <0.001 |  |  | 0.365 | <0.001 |  |  | 0.230 | <0.001 |  |
|  |  |  |  | | | | | | | | | | | |  |

EAPC, Estimated annual percentage changes; DALYs, Disability-adjusted life years;SDI, Socio-demographic Index.
