# Supplementary material for: The global burden of vascular intestinal diseases: results from the 2021 Global Burden of Disease Study and projections using Bayesian age-period-cohort analysis
Source: Environ Health Prev Med. 2024 Dec 11;29:71. doi: 10.1265/ehpm.24-00206 (PMC11653002; doi:10.1265/ehpm.24-00206)
Supplement: Supplementary file 16 — Additional file 16: Table S4 APC of VID from 1990 to 2021 for the World, SDI Regions, and the Top 3 Regions with the highest prevalence. [file ehpm-29-071-s016.docx]

| **Table S4 APC of VID from 1990 to 2021 for the World, SDI Regions, and the Top 3 Regions with the highest prevalence** | | | | | | | | | | | | | | |  |
| --- | --- | --- | --- | --- | --- | --- | --- | --- | --- | --- | --- | --- | --- | --- | --- |
|  | **Incidence (95% CI)** | |  | | **Prevalence (95% CI)** | | |  | **Deaths (95% CI)** | | |  | **DALYs (95% CI)** | |  |
|  |  |  |  |  |  |  |  |  |  |  |  |  |  |  |  |
|  |  |  |  |  |  |  |  |  |  |  |  |  |  |  |  |
|  |  |  |  |  |  |  |  |  |  |  |  |  |  |  |  |
| **Characteristics** | **APCs** | **p** |  |  | **APCs** | **p** |  |  | **APCs** | **p** |  |  | **APCs** | **p** |  |
| Global |  |  |  |  |  |  |  |  |  |  |  |  |  |  |  |
| slope1 | -0.456(-0.534, -0.378) | * |  |  | 0.114(0.080, 0.147) | * |  |  | -0.446(-0.896, 0.006) |  |  |  | -0.142(-0.606, 0.325) |  |  |
| slope2 | -0.020(-0.066, 0.027) |  |  |  | -0.378(-0.412, -0.345) | * |  |  | -0.806(-0.902, -0.710) | * |  |  | -0.902(-1.001, -0.804) | * |  |
| slope3 | -0.402(-0.440, -0.364) | * |  |  | -1.275(-1.347, -1.203) | * |  |  | -1.991(-2.057, -1.925) | * |  |  | -2.029(-2.187, -1.871) | * |  |
| slope4 | -0.930(-0.951, -0.910) | * |  |  | -0.696(-0.768, -0.623) | * |  |  | -2.241(-2.939, -1.538) | * |  |  | -1.818(-1.950, -1.686) | * |  |
| Low SDI |  |  |  |  |  |  |  |  |  |  |  |  |  |  |  |
| slope1 | 0.164(0.140, 0.187) | * |  |  | 0.187(0.152, 0.222) | * |  |  | 1.040(0.624, 1.459) | * |  |  | 0.684(0.447, 0.921) | * |  |
| slope2 | 0.922(0.796, 1.047) | * |  |  | 1.202(1.101, 1.302) | * |  |  | -1.140(-1.211, -1.070) | * |  |  | -1.314(-1.378, -1.249) | * |  |
| slope3 | -0.063(-0.138, 0.011) |  |  |  | -0.021(-0.188, 0.145) |  |  |  | 1.574(0.639, 2.518) | * |  |  | 0.087(-0.224, 0.399) |  |  |
| slope4 | 0.511(0.386, 0.636) | * |  |  | 0.945(0.818, 1.072) | * |  |  | -2.078(-2.276, -1.881) | * |  |  | -1.677(-1.860, -1.494) | * |  |
| Low middle SDI |  |  |  |  |  |  |  |  |  |  |  |  |  |  |  |
| slope1 | 0.451(0.425, 0.477) | * |  |  | 0.568(0.537, 0.599) | * |  |  | 0.272(-0.343, 0.891) |  |  |  | -0.029(-0.239, 0.182) |  |  |
| slope2 | 1.664(1.525, 1.803) | * |  |  | 1.989(1.820, 2.157) | * |  |  | -0.892(-0.987, -0.797) | * |  |  | -1.336(-1.505, -1.165) | * |  |
| slope3 | -0.142(-0.209, -0.076) | * |  |  | -0.256(-0.380, -0.131) | * |  |  | 0.997(-0.383, 2.396) |  |  |  | -0.791(-0.913, -0.669) | * |  |
| slope4 | 0.418(0.224, 0.612) | * |  |  | 0.533(0.408, 0.659) | * |  |  | -1.640(-2.001, -1.278) | * |  |  | -1.383(-1.591, -1.175) | * |  |
| Middle SDI |  |  |  |  |  |  |  |  |  |  |  |  |  |  |  |
| slope1 | 0.059(-0.006, 0.123) |  |  |  | 0.081(0.043, 0.119) | * |  |  | -0.303(-0.594, -0.012) | * |  |  | -1.112(-1.269, -0.954) | * |  |
| slope2 | 0.745(0.530, 0.961) | * |  |  | 0.744(0.688, 0.800) | * |  |  | -2.186(-2.718, -1.650) | * |  |  | 0.093(-1.083, 1.283) |  |  |
| slope3 | -0.901(-1.374, -0.425) | * |  |  | 0.073(0.045, 0.101) | * |  |  | 0.399(0.013, 0.788) | * |  |  | -1.647(-1.756, -1.538) | * |  |
| slope4 | 0.202(0.152, 0.253) | * |  |  | 0.209(0.116, 0.302) | * |  |  | -1.873(-1.933, -1.814) | * |  |  | -0.832(-1.570, -0.088) | * |  |
| High middle SDI |  |  |  |  |  |  |  |  |  |  |  |  |  |  |  |
| slope1 | -0.456(-0.503, -0.409) | * |  |  | 0.354(0.300, 0.407) | * |  |  | 0.768(-1.065, -0.470) | * |  |  | 1.301(0.822, 1.782) | * |  |
| slope2 | -0.013(-0.132, 0.105) |  |  |  | 0.102(0.002, 0.201) | * |  |  | 1.532(0.483, 2.592) | * |  |  | -2.996(-4.017, -1.963) | * |  |
| slope3 | -0.427(-0.648, -0.206) | * |  |  | -0.273(-0.458, -0.088) | * |  |  | -1.127(-1.252, -1.003) | * |  |  | 1.307(0.828, 1.787) | * |  |
| slope4 | -0.694(-0.741, -0.647) | * |  |  | -0.982(-1.012, -0.951) | * |  |  | -3.076(-4.655, -1.471) | * |  |  | -1.638(-1.700, -1.576) | * |  |
| High SDI |  |  |  |  |  |  |  |  |  |  |  |  |  |  |  |
| slope1 | -0.018(-0.068, 0.033) |  |  |  | 0.561(0.495, 0.627) | * |  |  | -0.694(-0.750, -0.637) | * |  |  | -0.678(-0.729, -0.627) | * |  |
| slope2 | 0.382(0.315, 0.450) | * |  |  | -0.158(-0.184, -0.132) | * |  |  | -1.711(-1.872, -1.550) | * |  |  | -1.498(-1.642, -1.353) | * |  |
| slope3 | -0.146(-0.173, -0.119) | * |  |  | -1.112(-1.220, -1.003) | * |  |  | -2.947(-3.105, -2.788) | * |  |  | -2.674(-2.863, -2.486) | * |  |
| slope4 | -0.691(-0.708, -0.673) | * |  |  | -0.303(-0.369, -0.238) | * |  |  | -2.066(-2.169, -1.962) | * |  |  | -1.750(-1.828, -1.673) | * |  |
| High-income North America |  |  |  |  |  |  |  |  |  |  |  |  |  |  |  |
| slope1 | NA |  |  |  | -0.025(-0.155, 0.106) |  |  |  | 0.377(0.241, 0.514) | * |  |  | NA |  |  |
| slope2 | NA |  |  |  | -0.494(-0.546, -0.441) | * |  |  | -1.759(-1.988, -1.530) | * |  |  | NA |  |  |
| slope3 | NA |  |  |  | -0.997(-1.042, -0.952) | * |  |  | -2.986(-3.100, -2.872) | * |  |  | NA |  |  |
| slope4 | NA |  |  |  | -0.434(-0.497, -0.370) | * |  |  | -0.797(-1.343, -0.248) | * |  |  | NA |  |  |
| Eastern Europe |  |  |  |  |  |  |  |  |  |  |  |  |  |  |  |
| slope1 | NA |  |  |  | 0.323(0.152, 0.495) | * |  |  | 0.808(0.123, 1.498) | * |  |  | NA |  |  |
| slope2 | NA |  |  |  | 0.901(0.849, 0.953) | * |  |  | 6.840(2.924, 10.906) | * |  |  | NA |  |  |
| slope3 | NA |  |  |  | 1.355(1.182, 1.528) | * |  |  | 1.586(1.266, 1.907) | * |  |  | NA |  |  |
| slope4 | NA |  |  |  | -0.528(-0.579, -0.477) | * |  |  | -0.169(-1.822, 1.513) |  |  |  | NA |  |  |
| High-income Asia Pacific |  |  |  |  |  |  |  |  |  |  |  |  |  |  |  |
| slope1 | NA |  |  |  | 0.205(-0.457, 0.872) |  |  |  | -1.933(-2.575, -1.287) | * |  |  | NA |  |  |
| slope2 | NA |  |  |  | 0.116(-0.026, 0.258) |  |  |  | -0.612(-1.262, 0.043) |  |  |  | NA |  |  |
| slope3 | NA |  |  |  | 1.505(1.029, 1.982) | * |  |  | 1.825(1.614, 2.037) | * |  |  | NA |  |  |
| slope4 | NA |  |  |  | -0.846(-0.969, -0.723) | * |  |  | -1.798(-4.180, 0.645) |  |  |  | NA |  |  |
|  |  |  |  | | | | | | | | | | | |  |

APC, Average percentage change;VID, Vascular intestinal disease; DALYs, Disability-adjusted life years;SDI, Socio-demographic Index.
