# Supplementary material for: The global burden of vascular intestinal diseases: results from the 2021 Global Burden of Disease Study and projections using Bayesian age-period-cohort analysis
Source: Environ Health Prev Med. 2024 Dec 11;29:71. doi: 10.1265/ehpm.24-00206 (PMC11653002; doi:10.1265/ehpm.24-00206)
Supplement: Supplementary file 17 — Additional file 17: Table S5 Age-standardized rates (95% UI) of vascular intestinal diseases from 1990 to 2021 and predicted age-standardized rates (95% UI) from 2022 to 2035. [file ehpm-29-071-s017.docx]

**Table S5 Age-standardized rates (95% UI) of vascular intestinal diseases from 1990 to 2021 and predicted age-standardized rates (95% UI) from 2022 to 2035.**

| \| **Time** \| **Incidence** \| \| \| \|  \| **Prevalence** \| \| \| \|  \| **Deaths** \| \| \| \|  \| **DALYs** \| \| \| \| \| --- \| --- \| --- \| --- \| --- \| --- \| --- \| --- \| --- \| --- \| --- \| --- \| --- \| --- \| --- \| --- \| --- \| --- \| --- \| --- \| \|  \| **ASR** \| **SD** \| **low_95%UI** \| **up_95%UI** \|  \| **ASR** \| **SD** \| **low_95%UI** \| **up_95%UI** \|  \| **ASR** \| **SD** \| **low_95%UI** \| **up_95%UI** \|  \| **ASR** \| **SD** \| **low_95%UI** \| **up_95%UI** \| \| 1990 \| 18.92 \| 0.02 \| 18.88 \| 18.97 \|  \| 2.34 \| 0.01 \| 2.33 \| 2.35 \|  \| 1.78 \| 0.01 \| 1.76 \| 1.79 \|  \| 31.48 \| 0.03 \| 31.42 \| 31.54 \| \| 1991 \| 18.82 \| 0.02 \| 18.77 \| 18.86 \|  \| 2.34 \| 0.01 \| 2.33 \| 2.36 \|  \| 1.77 \| 0.01 \| 1.76 \| 1.78 \|  \| 31.41 \| 0.03 \| 31.35 \| 31.47 \| \| 1992 \| 18.73 \| 0.02 \| 18.69 \| 18.77 \|  \| 2.35 \| 0.01 \| 2.34 \| 2.36 \|  \| 1.76 \| 0.01 \| 1.75 \| 1.77 \|  \| 31.37 \| 0.03 \| 31.31 \| 31.42 \| \| 1993 \| 18.65 \| 0.02 \| 18.61 \| 18.69 \|  \| 2.35 \| 0.01 \| 2.34 \| 2.37 \|  \| 1.76 \| 0.01 \| 1.74 \| 1.77 \|  \| 31.40 \| 0.03 \| 31.35 \| 31.46 \| \| 1994 \| 18.58 \| 0.02 \| 18.54 \| 18.62 \|  \| 2.36 \| 0.01 \| 2.34 \| 2.37 \|  \| 1.75 \| 0.01 \| 1.74 \| 1.76 \|  \| 31.29 \| 0.03 \| 31.23 \| 31.34 \| \| 1995 \| 18.53 \| 0.02 \| 18.49 \| 18.57 \|  \| 2.36 \| 0.01 \| 2.35 \| 2.37 \|  \| 1.73 \| 0.01 \| 1.72 \| 1.75 \|  \| 30.97 \| 0.03 \| 30.91 \| 31.02 \| \| 1996 \| 18.51 \| 0.02 \| 18.47 \| 18.55 \|  \| 2.36 \| 0.01 \| 2.35 \| 2.37 \|  \| 1.71 \| 0.01 \| 1.70 \| 1.72 \|  \| 30.49 \| 0.03 \| 30.44 \| 30.55 \| \| 1997 \| 18.52 \| 0.02 \| 18.48 \| 18.56 \|  \| 2.36 \| 0.01 \| 2.35 \| 2.37 \|  \| 1.69 \| 0.01 \| 1.67 \| 1.70 \|  \| 29.94 \| 0.03 \| 29.89 \| 29.99 \| \| 1998 \| 18.54 \| 0.02 \| 18.50 \| 18.58 \|  \| 2.36 \| 0.01 \| 2.35 \| 2.37 \|  \| 1.67 \| 0.01 \| 1.66 \| 1.68 \|  \| 29.58 \| 0.03 \| 29.53 \| 29.63 \| \| 1999 \| 18.54 \| 0.02 \| 18.50 \| 18.58 \|  \| 2.36 \| 0.01 \| 2.35 \| 2.37 \|  \| 1.65 \| 0.01 \| 1.64 \| 1.67 \|  \| 29.39 \| 0.03 \| 29.34 \| 29.44 \| \| 2000 \| 18.52 \| 0.02 \| 18.48 \| 18.56 \|  \| 2.35 \| 0.01 \| 2.34 \| 2.37 \|  \| 1.66 \| 0.01 \| 1.64 \| 1.67 \|  \| 29.45 \| 0.03 \| 29.40 \| 29.50 \| \| 2001 \| 18.47 \| 0.02 \| 18.44 \| 18.51 \|  \| 2.35 \| 0.01 \| 2.34 \| 2.36 \|  \| 1.65 \| 0.01 \| 1.63 \| 1.66 \|  \| 29.22 \| 0.03 \| 29.17 \| 29.27 \| \| 2002 \| 18.43 \| 0.02 \| 18.39 \| 18.47 \|  \| 2.34 \| 0.01 \| 2.33 \| 2.35 \|  \| 1.64 \| 0.01 \| 1.63 \| 1.65 \|  \| 29.07 \| 0.02 \| 29.02 \| 29.12 \| \| 2003 \| 18.38 \| 0.02 \| 18.34 \| 18.42 \|  \| 2.34 \| 0.01 \| 2.32 \| 2.35 \|  \| 1.63 \| 0.01 \| 1.62 \| 1.64 \|  \| 28.83 \| 0.02 \| 28.78 \| 28.87 \| \| 2004 \| 18.32 \| 0.02 \| 18.29 \| 18.36 \|  \| 2.33 \| 0.01 \| 2.32 \| 2.34 \|  \| 1.60 \| 0.01 \| 1.59 \| 1.61 \|  \| 28.31 \| 0.02 \| 28.27 \| 28.36 \| \| 2005 \| 18.26 \| 0.02 \| 18.23 \| 18.30 \|  \| 2.32 \| 0.01 \| 2.31 \| 2.33 \|  \| 1.58 \| 0.01 \| 1.57 \| 1.59 \|  \| 27.97 \| 0.02 \| 27.93 \| 28.02 \| \| 2006 \| 18.19 \| 0.02 \| 18.15 \| 18.22 \|  \| 2.31 \| 0.01 \| 2.30 \| 2.32 \|  \| 1.55 \| 0.01 \| 1.54 \| 1.56 \|  \| 27.35 \| 0.02 \| 27.30 \| 27.39 \| \| 2007 \| 18.11 \| 0.02 \| 18.07 \| 18.14 \|  \| 2.30 \| 0.01 \| 2.29 \| 2.31 \|  \| 1.52 \| 0.01 \| 1.51 \| 1.53 \|  \| 26.88 \| 0.02 \| 26.84 \| 26.93 \| \| 2008 \| 18.02 \| 0.02 \| 17.98 \| 18.05 \|  \| 2.29 \| 0.01 \| 2.28 \| 2.30 \|  \| 1.50 \| 0.00 \| 1.49 \| 1.51 \|  \| 26.48 \| 0.02 \| 26.44 \| 26.52 \| \| 2009 \| 17.91 \| 0.02 \| 17.88 \| 17.95 \|  \| 2.28 \| 0.01 \| 2.26 \| 2.29 \|  \| 1.47 \| 0.00 \| 1.46 \| 1.48 \|  \| 25.90 \| 0.02 \| 25.86 \| 25.95 \| \| 2010 \| 17.81 \| 0.02 \| 17.78 \| 17.84 \|  \| 2.26 \| 0.01 \| 2.25 \| 2.27 \|  \| 1.43 \| 0.00 \| 1.42 \| 1.44 \|  \| 25.36 \| 0.02 \| 25.32 \| 25.40 \| \| 2011 \| 17.68 \| 0.02 \| 17.64 \| 17.71 \|  \| 2.23 \| 0.01 \| 2.22 \| 2.25 \|  \| 1.40 \| 0.00 \| 1.39 \| 1.41 \|  \| 24.70 \| 0.02 \| 24.66 \| 24.74 \| \| 2012 \| 17.52 \| 0.02 \| 17.49 \| 17.55 \|  \| 2.21 \| 0.01 \| 2.20 \| 2.22 \|  \| 1.37 \| 0.00 \| 1.36 \| 1.38 \|  \| 24.19 \| 0.02 \| 24.16 \| 24.23 \| \| 2013 \| 17.35 \| 0.02 \| 17.32 \| 17.38 \|  \| 2.18 \| 0.01 \| 2.17 \| 2.19 \|  \| 1.35 \| 0.00 \| 1.34 \| 1.35 \|  \| 23.80 \| 0.02 \| 23.76 \| 23.84 \| \| 2014 \| 17.17 \| 0.02 \| 17.14 \| 17.20 \|  \| 2.15 \| 0.01 \| 2.14 \| 2.16 \|  \| 1.32 \| 0.00 \| 1.31 \| 1.33 \|  \| 23.36 \| 0.02 \| 23.33 \| 23.40 \| \| 2015 \| 17.03 \| 0.02 \| 17.00 \| 17.06 \|  \| 2.12 \| 0.00 \| 2.11 \| 2.13 \|  \| 1.30 \| 0.00 \| 1.29 \| 1.31 \|  \| 22.97 \| 0.02 \| 22.93 \| 23.00 \| \| 2016 \| 16.86 \| 0.01 \| 16.83 \| 16.89 \|  \| 2.10 \| 0.00 \| 2.09 \| 2.11 \|  \| 1.27 \| 0.00 \| 1.27 \| 1.28 \|  \| 22.65 \| 0.02 \| 22.61 \| 22.68 \| \| 2017 \| 16.69 \| 0.01 \| 16.66 \| 16.72 \|  \| 2.09 \| 0.00 \| 2.08 \| 2.10 \|  \| 1.24 \| 0.00 \| 1.23 \| 1.25 \|  \| 22.10 \| 0.02 \| 22.07 \| 22.13 \| \| 2018 \| 16.52 \| 0.01 \| 16.49 \| 16.55 \|  \| 2.07 \| 0.00 \| 2.06 \| 2.08 \|  \| 1.22 \| 0.00 \| 1.21 \| 1.23 \|  \| 21.78 \| 0.02 \| 21.74 \| 21.81 \| \| 2019 \| 16.38 \| 0.01 \| 16.35 \| 16.41 \|  \| 2.06 \| 0.00 \| 2.05 \| 2.07 \|  \| 1.19 \| 0.00 \| 1.19 \| 1.20 \|  \| 21.39 \| 0.02 \| 21.35 \| 21.42 \| \| 2020 \| 16.29 \| 0.01 \| 16.27 \| 16.32 \|  \| 2.05 \| 0.00 \| 2.04 \| 2.06 \|  \| 1.16 \| 0.00 \| 1.16 \| 1.17 \|  \| 20.87 \| 0.02 \| 20.84 \| 20.90 \| \| 2021 \| 16.08 \| 0.01 \| 16.06 \| 16.11 \|  \| 2.03 \| 0.00 \| 2.02 \| 2.04 \|  \| 1.14 \| 0.00 \| 1.14 \| 1.15 \|  \| 20.57 \| 0.02 \| 20.54 \| 20.60 \| \| 2022 \| 16.28 \| 0.13 \| 16.03 \| 16.54 \|  \| 2.03 \| 0.01 \| 2.00 \| 2.06 \|  \| 1.13 \| 0.01 \| 1.11 \| 1.16 \|  \| 20.69 \| 0.45 \| 19.81 \| 21.58 \| \| 2023 \| 16.20 \| 0.15 \| 15.91 \| 16.49 \|  \| 2.02 \| 0.02 \| 1.98 \| 2.06 \|  \| 1.12 \| 0.02 \| 1.08 \| 1.15 \|  \| 20.39 \| 0.47 \| 19.46 \| 21.32 \| \| 2024 \| 16.11 \| 0.16 \| 15.80 \| 16.43 \|  \| 2.01 \| 0.02 \| 1.97 \| 2.05 \|  \| 1.10 \| 0.02 \| 1.06 \| 1.13 \|  \| 20.09 \| 0.50 \| 19.11 \| 21.06 \| \| 2025 \| 16.03 \| 0.17 \| 15.69 \| 16.37 \|  \| 2.00 \| 0.02 \| 1.95 \| 2.05 \|  \| 1.08 \| 0.02 \| 1.04 \| 1.12 \|  \| 19.78 \| 0.52 \| 18.77 \| 20.79 \| \| 2026 \| 15.95 \| 0.19 \| 15.58 \| 16.32 \|  \| 1.99 \| 0.03 \| 1.94 \| 2.04 \|  \| 1.06 \| 0.02 \| 1.02 \| 1.11 \|  \| 19.49 \| 0.54 \| 18.44 \| 20.54 \| \| 2027 \| 15.87 \| 0.20 \| 15.48 \| 16.27 \|  \| 1.98 \| 0.03 \| 1.92 \| 2.04 \|  \| 1.05 \| 0.02 \| 1.00 \| 1.10 \|  \| 19.21 \| 0.55 \| 18.13 \| 20.30 \| \| 2028 \| 15.79 \| 0.21 \| 15.38 \| 16.21 \|  \| 1.97 \| 0.03 \| 1.91 \| 2.03 \|  \| 1.03 \| 0.03 \| 0.98 \| 1.08 \|  \| 18.94 \| 0.57 \| 17.82 \| 20.07 \| \| 2029 \| 15.71 \| 0.22 \| 15.27 \| 16.15 \|  \| 1.96 \| 0.03 \| 1.89 \| 2.02 \|  \| 1.02 \| 0.03 \| 0.96 \| 1.07 \|  \| 18.67 \| 0.59 \| 17.52 \| 19.83 \| \| 2030 \| 15.63 \| 0.24 \| 15.17 \| 16.09 \|  \| 1.95 \| 0.04 \| 1.88 \| 2.02 \|  \| 1.00 \| 0.03 \| 0.94 \| 1.06 \|  \| 18.41 \| 0.61 \| 17.22 \| 19.60 \| \| 2031 \| 15.55 \| 0.25 \| 15.07 \| 16.04 \|  \| 1.94 \| 0.04 \| 1.86 \| 2.01 \|  \| 0.99 \| 0.03 \| 0.93 \| 1.05 \|  \| 18.15 \| 0.62 \| 16.93 \| 19.37 \| \| 2032 \| 15.47 \| 0.26 \| 14.96 \| 15.98 \|  \| 1.93 \| 0.04 \| 1.85 \| 2.01 \|  \| 0.97 \| 0.03 \| 0.91 \| 1.04 \|  \| 17.90 \| 0.64 \| 16.65 \| 19.16 \| \| 2033 \| 15.39 \| 0.27 \| 14.86 \| 15.93 \|  \| 1.92 \| 0.04 \| 1.84 \| 2.00 \|  \| 0.96 \| 0.03 \| 0.89 \| 1.03 \|  \| 17.66 \| 0.66 \| 16.37 \| 18.95 \| \| 2034 \| 15.31 \| 0.28 \| 14.75 \| 15.87 \|  \| 1.91 \| 0.04 \| 1.82 \| 2.00 \|  \| 0.95 \| 0.04 \| 0.88 \| 1.02 \|  \| 17.42 \| 0.67 \| 16.10 \| 18.74 \| \| 2035 \| 15.23 \| 0.30 \| 14.65 \| 15.81 \|  \| 1.90 \| 0.05 \| 1.81 \| 1.99 \|  \| 0.93 \| 0.04 \| 0.86 \| 1.01 \|  \| 17.18 \| 0.69 \| 15.84 \| 18.52 \| |  |
| --- | --- | --- | --- | --- | --- | --- | --- | --- | --- | --- | --- | --- | --- | --- | --- | --- | --- | --- | --- | --- | --- | --- | --- | --- | --- | --- | --- | --- | --- | --- | --- | --- | --- | --- | --- | --- | --- | --- | --- | --- | --- | --- | --- | --- | --- | --- | --- | --- | --- | --- | --- | --- | --- | --- | --- | --- | --- | --- | --- | --- | --- | --- | --- | --- | --- | --- | --- | --- | --- | --- | --- | --- | --- | --- | --- | --- | --- | --- | --- | --- | --- | --- | --- | --- | --- | --- | --- | --- | --- | --- | --- | --- | --- | --- | --- | --- | --- | --- | --- | --- | --- | --- | --- | --- | --- | --- | --- | --- | --- | --- | --- | --- | --- | --- | --- | --- | --- | --- | --- | --- | --- | --- | --- | --- | --- | --- | --- | --- | --- | --- | --- | --- | --- | --- | --- | --- | --- | --- | --- | --- | --- | --- | --- | --- | --- | --- | --- | --- | --- | --- | --- | --- | --- | --- | --- | --- | --- | --- | --- | --- | --- | --- | --- | --- | --- | --- | --- | --- | --- | --- | --- | --- | --- | --- | --- | --- | --- | --- | --- | --- | --- | --- | --- | --- | --- | --- | --- | --- | --- | --- | --- | --- | --- | --- | --- | --- | --- | --- | --- | --- | --- | --- | --- | --- | --- | --- | --- | --- | --- | --- | --- | --- | --- | --- | --- | --- | --- | --- | --- | --- | --- | --- | --- | --- | --- | --- | --- | --- | --- | --- | --- | --- | --- | --- | --- | --- | --- | --- | --- | --- | --- | --- | --- | --- | --- | --- | --- | --- | --- | --- | --- | --- | --- | --- | --- | --- | --- | --- | --- | --- | --- | --- | --- | --- | --- | --- | --- | --- | --- | --- | --- | --- | --- | --- | --- | --- | --- | --- | --- | --- | --- | --- | --- | --- | --- | --- | --- | --- | --- | --- | --- | --- | --- | --- | --- | --- | --- | --- | --- | --- | --- | --- | --- | --- | --- | --- | --- | --- | --- | --- | --- | --- | --- | --- | --- | --- | --- | --- | --- | --- | --- | --- | --- | --- | --- | --- | --- | --- | --- | --- | --- | --- | --- | --- | --- | --- | --- | --- | --- | --- | --- | --- | --- | --- | --- | --- | --- | --- | --- | --- | --- | --- | --- | --- | --- | --- | --- | --- | --- | --- | --- | --- | --- | --- | --- | --- | --- | --- | --- | --- | --- | --- | --- | --- | --- | --- | --- | --- | --- | --- | --- | --- | --- | --- | --- | --- | --- | --- | --- | --- | --- | --- | --- | --- | --- | --- | --- | --- | --- | --- | --- | --- | --- | --- | --- | --- | --- | --- | --- | --- | --- | --- | --- | --- | --- | --- | --- | --- | --- | --- | --- | --- | --- | --- | --- | --- | --- | --- | --- | --- | --- | --- | --- | --- | --- | --- | --- | --- | --- | --- | --- | --- | --- | --- | --- | --- | --- | --- | --- | --- | --- | --- | --- | --- | --- | --- | --- | --- | --- | --- | --- | --- | --- | --- | --- | --- | --- | --- | --- | --- | --- | --- | --- | --- | --- | --- | --- | --- | --- | --- | --- | --- | --- | --- | --- | --- | --- | --- | --- | --- | --- | --- | --- | --- | --- | --- | --- | --- | --- | --- | --- | --- | --- | --- | --- | --- | --- | --- | --- | --- | --- | --- | --- | --- | --- | --- | --- | --- | --- | --- | --- | --- | --- | --- | --- | --- | --- | --- | --- | --- | --- | --- | --- | --- | --- | --- | --- | --- | --- | --- | --- | --- | --- | --- | --- | --- | --- | --- | --- | --- | --- | --- | --- | --- | --- | --- | --- | --- | --- | --- | --- | --- | --- | --- | --- | --- | --- | --- | --- | --- | --- | --- | --- | --- | --- | --- | --- | --- | --- | --- | --- | --- | --- | --- | --- | --- | --- | --- | --- | --- | --- | --- | --- | --- | --- | --- | --- | --- | --- | --- | --- | --- | --- | --- | --- | --- | --- | --- | --- | --- | --- | --- | --- | --- | --- | --- | --- | --- | --- | --- | --- | --- | --- | --- | --- | --- | --- | --- | --- | --- | --- | --- | --- | --- | --- | --- | --- | --- | --- | --- | --- | --- | --- | --- | --- | --- | --- | --- | --- | --- | --- | --- | --- | --- | --- | --- | --- | --- | --- | --- | --- | --- | --- | --- | --- | --- | --- | --- | --- | --- | --- | --- | --- | --- | --- | --- | --- | --- | --- | --- | --- | --- | --- | --- | --- | --- | --- | --- | --- | --- | --- | --- | --- | --- | --- | --- | --- | --- | --- | --- | --- | --- | --- | --- | --- | --- | --- | --- | --- | --- | --- | --- | --- | --- | --- | --- | --- | --- | --- | --- | --- | --- | --- | --- | --- | --- | --- | --- | --- | --- | --- | --- | --- | --- | --- | --- | --- | --- | --- | --- | --- | --- | --- | --- | --- | --- | --- | --- | --- | --- | --- | --- | --- | --- | --- | --- | --- | --- | --- | --- | --- | --- | --- | --- | --- | --- | --- | --- | --- | --- | --- | --- | --- | --- | --- | --- | --- | --- | --- | --- | --- | --- | --- | --- | --- | --- | --- | --- | --- | --- | --- | --- | --- | --- | --- | --- | --- | --- | --- | --- | --- | --- | --- | --- | --- | --- | --- | --- | --- | --- | --- | --- | --- | --- | --- | --- | --- | --- | --- | --- | --- | --- | --- | --- | --- | --- | --- | --- | --- | --- | --- | --- | --- | --- | --- | --- | --- | --- | --- | --- | --- | --- | --- | --- | --- | --- | --- | --- | --- | --- | --- | --- | --- | --- | --- | --- | --- | --- | --- | --- | --- | --- | --- | --- | --- | --- | --- | --- | --- | --- | --- | --- | --- | --- | --- | --- | --- | --- | --- | --- | --- | --- | --- | --- | --- | --- | --- | --- | --- | --- | --- | --- | --- | --- | --- | --- | --- | --- | --- | --- | --- | --- | --- | --- | --- | --- | --- | --- | --- | --- | --- | --- | --- | --- | --- | --- | --- | --- | --- | --- | --- | --- | --- | --- | --- | --- | --- | --- | --- | --- | --- | --- | --- | --- | --- | --- | --- | --- | --- | --- | --- | --- | --- | --- | --- | --- | --- | --- | --- | --- | --- | --- | --- | --- | --- | --- | --- | --- | --- | --- | --- |

ASR, Age-standardized rates; DALYs, Disability-adjusted life years;UI, Uncertainty Interval.
