# Supplementary material for: The global burden of vascular intestinal diseases: results from the 2021 Global Burden of Disease Study and projections using Bayesian age-period-cohort analysis
Source: Environ Health Prev Med. 2024 Dec 11;29:71. doi: 10.1265/ehpm.24-00206 (PMC11653002; doi:10.1265/ehpm.24-00206)
Supplement: Supplementary file 18 — Additional file 18: Table S6 Age-standardized rates (95% UI) of vascular intestinal diseases or different age groups from 1990 to 2021 and predicted age-standardized rates (95% UI) from 2022 to 2035. [file ehpm-29-071-s018.docx]

**Table S6 Age-standardized rates (95% UI) of vascular intestinal diseases or different age groups from 1990 to 2021 and predicted age-standardized rates (95% UI) from 2022 to 2035**

|  |  | **Incidence** | | | |  | **Prevalence** | | | |  | **Deaths** | | | |  | **DALYs** | | | |
| --- | --- | --- | --- | --- | --- | --- | --- | --- | --- | --- | --- | --- | --- | --- | --- | --- | --- | --- | --- | --- |
| **Time** | **group** | **ASR** | **SD** | **low_95%UI** | **up_95%UI** |  | **ASR** | **SD** | **low_95%UI** | **up_95%UI** |  | **ASR** | **SD** | **low_95%UI** | **up_95%UI** |  | **ASR** | **SD** | **low_95%UI** | **up_95%UI** |
| 1990 | <5 years | 1.62 | 16.36 | -30.45 | 33.69 |  | 0.27 | 27.57 | -53.77 | 54.31 |  | 0.02 | 24.34 | -47.68 | 47.72 |  | 2.83 | 15.01 | -26.59 | 32.25 |
| 1991 | <5 years | 1.62 | 16.36 | -30.44 | 33.68 |  | 0.27 | 27.63 | -53.88 | 54.42 |  | 0.02 | 24.65 | -48.29 | 48.34 |  | 2.78 | 15.03 | -26.69 | 32.25 |
| 1992 | <5 years | 1.61 | 16.33 | -30.39 | 33.62 |  | 0.27 | 27.15 | -52.94 | 53.49 |  | 0.02 | 24.93 | -48.84 | 48.88 |  | 2.73 | 15.06 | -26.79 | 32.25 |
| 1993 | <5 years | 1.60 | 16.28 | -30.31 | 33.52 |  | 0.27 | 26.49 | -51.64 | 52.19 |  | 0.02 | 25.02 | -49.02 | 49.07 |  | 2.66 | 15.10 | -26.92 | 32.25 |
| 1994 | <5 years | 1.59 | 16.23 | -30.21 | 33.40 |  | 0.27 | 25.63 | -49.96 | 50.51 |  | 0.02 | 25.01 | -49.00 | 49.04 |  | 2.58 | 15.14 | -27.09 | 32.25 |
| 1995 | <5 years | 1.58 | 16.16 | -30.10 | 33.26 |  | 0.27 | 24.72 | -48.18 | 48.72 |  | 0.02 | 24.95 | -48.88 | 48.92 |  | 2.51 | 15.18 | -27.25 | 32.26 |
| 1996 | <5 years | 1.56 | 16.10 | -29.99 | 33.12 |  | 0.27 | 23.80 | -46.37 | 46.92 |  | 0.02 | 24.89 | -48.76 | 48.81 |  | 2.41 | 15.23 | -27.44 | 32.27 |
| 1997 | <5 years | 1.55 | 16.04 | -29.89 | 32.98 |  | 0.27 | 22.78 | -44.38 | 44.93 |  | 0.02 | 24.79 | -48.57 | 48.62 |  | 2.32 | 15.28 | -27.63 | 32.28 |
| 1998 | <5 years | 1.53 | 15.97 | -29.77 | 32.83 |  | 0.27 | 21.81 | -42.48 | 43.02 |  | 0.02 | 24.64 | -48.27 | 48.31 |  | 2.22 | 15.34 | -27.85 | 32.30 |
| 1999 | <5 years | 1.51 | 15.91 | -29.66 | 32.69 |  | 0.27 | 20.99 | -40.87 | 41.42 |  | 0.02 | 24.57 | -48.13 | 48.17 |  | 2.14 | 15.39 | -28.03 | 32.32 |
| 2000 | <5 years | 1.50 | 15.87 | -29.60 | 32.60 |  | 0.27 | 20.41 | -39.73 | 40.27 |  | 0.02 | 24.29 | -47.58 | 47.63 |  | 2.07 | 15.44 | -28.19 | 32.33 |
| 2001 | <5 years | 1.49 | 15.86 | -29.59 | 32.57 |  | 0.27 | 20.02 | -38.97 | 39.51 |  | 0.02 | 23.77 | -46.56 | 46.61 |  | 1.99 | 15.49 | -28.37 | 32.36 |
| 2002 | <5 years | 1.48 | 15.85 | -29.59 | 32.55 |  | 0.27 | 19.70 | -38.34 | 38.88 |  | 0.02 | 22.72 | -44.52 | 44.56 |  | 1.92 | 15.54 | -28.54 | 32.38 |
| 2003 | <5 years | 1.47 | 15.84 | -29.58 | 32.52 |  | 0.27 | 19.37 | -37.69 | 38.23 |  | 0.02 | 21.70 | -42.50 | 42.54 |  | 1.86 | 15.59 | -28.70 | 32.41 |
| 2004 | <5 years | 1.47 | 15.83 | -29.56 | 32.49 |  | 0.27 | 19.07 | -37.12 | 37.65 |  | 0.02 | 21.32 | -41.76 | 41.80 |  | 1.79 | 15.64 | -28.85 | 32.44 |
| 2005 | <5 years | 1.46 | 15.82 | -29.55 | 32.48 |  | 0.27 | 18.87 | -36.71 | 37.25 |  | 0.02 | 21.23 | -41.59 | 41.63 |  | 1.73 | 15.68 | -29.00 | 32.47 |
| 2006 | <5 years | 1.46 | 15.83 | -29.57 | 32.49 |  | 0.27 | 18.84 | -36.65 | 37.19 |  | 0.02 | 21.38 | -41.89 | 41.93 |  | 1.69 | 15.72 | -29.12 | 32.50 |
| 2007 | <5 years | 1.46 | 15.84 | -29.59 | 32.51 |  | 0.27 | 18.92 | -36.82 | 37.35 |  | 0.02 | 21.72 | -42.56 | 42.60 |  | 1.64 | 15.76 | -29.26 | 32.53 |
| 2008 | <5 years | 1.46 | 15.85 | -29.61 | 32.53 |  | 0.27 | 19.07 | -37.10 | 37.63 |  | 0.02 | 22.47 | -44.02 | 44.06 |  | 1.58 | 15.81 | -29.40 | 32.56 |
| 2009 | <5 years | 1.46 | 15.86 | -29.62 | 32.55 |  | 0.27 | 19.29 | -37.55 | 38.08 |  | 0.02 | 23.51 | -46.07 | 46.11 |  | 1.54 | 15.84 | -29.51 | 32.59 |
| 2010 | <5 years | 1.47 | 15.88 | -29.64 | 32.59 |  | 0.27 | 19.73 | -38.40 | 38.94 |  | 0.02 | 24.69 | -48.38 | 48.42 |  | 1.48 | 15.90 | -29.68 | 32.64 |
| 2011 | <5 years | 1.48 | 15.91 | -29.69 | 32.66 |  | 0.27 | 20.35 | -39.63 | 40.16 |  | 0.02 | 25.92 | -50.78 | 50.82 |  | 1.43 | 15.94 | -29.81 | 32.67 |
| 2012 | <5 years | 1.49 | 15.95 | -29.77 | 32.75 |  | 0.26 | 20.91 | -40.73 | 41.25 |  | 0.02 | 27.09 | -53.07 | 53.11 |  | 1.40 | 15.97 | -29.91 | 32.70 |
| 2013 | <5 years | 1.49 | 15.97 | -29.81 | 32.79 |  | 0.26 | 21.23 | -41.35 | 41.88 |  | 0.02 | 27.94 | -54.75 | 54.79 |  | 1.37 | 16.00 | -29.98 | 32.72 |
| 2014 | <5 years | 1.49 | 15.98 | -29.83 | 32.81 |  | 0.26 | 21.45 | -41.78 | 42.31 |  | 0.02 | 28.43 | -55.71 | 55.75 |  | 1.33 | 16.04 | -30.10 | 32.76 |
| 2015 | <5 years | 1.49 | 15.98 | -29.83 | 32.81 |  | 0.26 | 21.41 | -41.70 | 42.23 |  | 0.02 | 28.09 | -55.03 | 55.06 |  | 1.29 | 16.08 | -30.22 | 32.81 |
| 2016 | <5 years | 1.49 | 15.97 | -29.81 | 32.80 |  | 0.26 | 21.16 | -41.20 | 41.73 |  | 0.02 | 25.93 | -50.81 | 50.84 |  | 1.25 | 16.13 | -30.38 | 32.87 |
| 2017 | <5 years | 1.50 | 15.97 | -29.81 | 32.81 |  | 0.26 | 20.90 | -40.69 | 41.22 |  | 0.01 | 22.26 | -43.62 | 43.65 |  | 1.19 | 16.21 | -30.58 | 32.95 |
| 2018 | <5 years | 1.51 | 15.98 | -29.82 | 32.84 |  | 0.26 | 20.71 | -40.32 | 40.85 |  | 0.01 | 20.63 | -40.41 | 40.44 |  | 1.13 | 16.28 | -30.77 | 33.03 |
| 2019 | <5 years | 1.52 | 15.98 | -29.79 | 32.83 |  | 0.27 | 20.20 | -39.32 | 39.85 |  | 0.01 | 19.49 | -38.19 | 38.22 |  | 1.09 | 16.33 | -30.92 | 33.10 |
| 2020 | <5 years | 1.52 | 15.93 | -29.71 | 32.75 |  | 0.27 | 19.47 | -37.89 | 38.43 |  | 0.01 | 18.20 | -35.67 | 35.69 |  | 1.01 | 16.44 | -31.22 | 33.23 |
| 2021 | <5 years | 1.53 | 15.92 | -29.68 | 32.73 |  | 0.27 | 19.56 | -38.07 | 38.61 |  | 0.01 | 16.95 | -33.22 | 33.24 |  | 0.95 | 16.51 | -31.42 | 33.31 |
| 2022 | <5 years | 1.54 | 19.78 | -37.22 | 40.30 |  | 0.27 | 24.47 | -47.69 | 48.23 |  | 0.01 | 16.23 | -31.80 | 31.82 |  | 0.89 | 15.98 | -30.43 | 32.21 |
| 2023 | <5 years | 1.55 | 23.93 | -45.36 | 48.46 |  | 0.27 | 30.33 | -59.18 | 59.73 |  | 0.01 | 16.05 | -31.44 | 31.46 |  | 0.82 | 14.81 | -28.21 | 29.85 |
| 2024 | <5 years | 1.56 | 30.34 | -57.91 | 61.02 |  | 0.27 | 37.03 | -72.31 | 72.86 |  | 0.01 | 16.29 | -31.92 | 31.94 |  | 0.76 | 15.07 | -28.78 | 30.30 |
| 2025 | <5 years | 1.57 | 37.29 | -71.52 | 74.65 |  | 0.28 | 43.19 | -84.37 | 84.93 |  | 0.01 | 16.81 | -32.94 | 32.96 |  | 0.70 | 17.74 | -34.06 | 35.47 |
| 2026 | <5 years | 1.57 | 43.68 | -84.03 | 87.18 |  | 0.28 | 48.38 | -94.54 | 95.10 |  | 0.01 | 17.49 | -34.27 | 34.28 |  | 0.65 | 21.85 | -42.18 | 43.49 |
| 2027 | <5 years | 1.58 | 49.11 | -94.68 | 97.85 |  | 0.28 | 52.60 | -102.81 | 103.37 |  | 0.01 | 18.52 | -36.29 | 36.30 |  | 0.60 | 26.68 | -51.69 | 52.90 |
| 2028 | <5 years | 1.60 | 53.58 | -103.43 | 106.62 |  | 0.28 | 55.99 | -109.45 | 110.02 |  | 0.01 | 20.43 | -40.05 | 40.06 |  | 0.56 | 31.66 | -61.50 | 62.62 |
| 2029 | <5 years | 1.61 | 57.19 | -110.50 | 113.71 |  | 0.28 | 58.68 | -114.74 | 115.31 |  | 0.01 | 22.33 | -43.76 | 43.77 |  | 0.52 | 36.47 | -70.97 | 72.01 |
| 2030 | <5 years | 1.62 | 60.08 | -116.15 | 119.38 |  | 0.29 | 60.82 | -118.92 | 119.49 |  | 0.00 | 24.16 | -47.35 | 47.36 |  | 0.48 | 40.94 | -79.76 | 80.73 |
| 2031 | <5 years | 1.63 | 62.38 | -120.64 | 123.90 |  | 0.29 | 62.50 | -122.21 | 122.79 |  | 0.00 | 25.90 | -50.77 | 50.78 |  | 0.45 | 45.00 | -87.74 | 88.64 |
| 2032 | <5 years | 1.64 | 64.19 | -124.18 | 127.46 |  | 0.29 | 63.80 | -124.76 | 125.34 |  | 0.00 | 27.54 | -53.98 | 53.98 |  | 0.42 | 48.62 | -94.88 | 95.71 |
| 2033 | <5 years | 1.66 | 65.60 | -126.93 | 130.24 |  | 0.30 | 64.79 | -126.70 | 127.29 |  | 0.00 | 29.06 | -56.96 | 56.97 |  | 0.39 | 51.83 | -101.19 | 101.97 |
| 2034 | <5 years | 1.67 | 66.68 | -129.03 | 132.37 |  | 0.30 | 65.52 | -128.13 | 128.73 |  | 0.00 | 30.47 | -59.72 | 59.72 |  | 0.36 | 54.64 | -106.73 | 107.46 |
| 2035 | <5 years | 1.68 | 67.49 | -130.59 | 133.96 |  | 0.30 | 66.03 | -129.12 | 129.73 |  | 0.00 | 31.76 | -62.24 | 62.25 |  | 0.34 | 57.09 | -111.56 | 112.23 |
| 1990 | 5-9 years | 2.48 | 15.48 | -27.86 | 32.82 |  | 0.35 | 27.45 | -53.46 | 54.15 |  | 0.04 | 22.13 | -43.33 | 43.40 |  | 2.96 | 14.93 | -26.29 | 32.22 |
| 1991 | 5-9 years | 2.48 | 15.50 | -27.91 | 32.87 |  | 0.34 | 27.86 | -54.26 | 54.95 |  | 0.04 | 21.82 | -42.73 | 42.81 |  | 2.93 | 14.94 | -26.35 | 32.22 |
| 1992 | 5-9 years | 2.47 | 15.52 | -27.96 | 32.89 |  | 0.34 | 27.79 | -54.13 | 54.81 |  | 0.04 | 21.48 | -42.07 | 42.15 |  | 2.92 | 14.95 | -26.38 | 32.22 |
| 1993 | 5-9 years | 2.45 | 15.53 | -27.99 | 32.89 |  | 0.34 | 27.61 | -53.76 | 54.45 |  | 0.04 | 21.32 | -41.74 | 41.82 |  | 2.91 | 14.95 | -26.40 | 32.22 |
| 1994 | 5-9 years | 2.44 | 15.53 | -28.00 | 32.88 |  | 0.34 | 27.28 | -53.13 | 53.81 |  | 0.04 | 21.57 | -42.24 | 42.31 |  | 2.89 | 14.96 | -26.43 | 32.22 |
| 1995 | 5-9 years | 2.43 | 15.51 | -27.98 | 32.83 |  | 0.34 | 26.68 | -51.96 | 52.64 |  | 0.04 | 22.23 | -43.54 | 43.61 |  | 2.90 | 14.96 | -26.42 | 32.22 |
| 1996 | 5-9 years | 2.41 | 15.48 | -27.93 | 32.75 |  | 0.34 | 25.61 | -49.86 | 50.54 |  | 0.04 | 22.72 | -44.50 | 44.57 |  | 2.88 | 14.97 | -26.45 | 32.21 |
| 1997 | 5-9 years | 2.38 | 15.43 | -27.86 | 32.62 |  | 0.34 | 24.08 | -46.86 | 47.55 |  | 0.04 | 23.08 | -45.19 | 45.27 |  | 2.88 | 14.97 | -26.46 | 32.21 |
| 1998 | 5-9 years | 2.35 | 15.37 | -27.78 | 32.47 |  | 0.34 | 22.45 | -43.66 | 44.35 |  | 0.04 | 23.12 | -45.28 | 45.35 |  | 2.88 | 14.97 | -26.46 | 32.21 |
| 1999 | 5-9 years | 2.32 | 15.32 | -27.70 | 32.34 |  | 0.34 | 21.08 | -40.97 | 41.65 |  | 0.04 | 23.04 | -45.12 | 45.20 |  | 2.87 | 14.97 | -26.47 | 32.21 |
| 2000 | 5-9 years | 2.31 | 15.28 | -27.65 | 32.26 |  | 0.34 | 20.18 | -39.21 | 39.89 |  | 0.04 | 22.84 | -44.72 | 44.80 |  | 2.90 | 14.96 | -26.41 | 32.21 |
| 2001 | 5-9 years | 2.30 | 15.28 | -27.64 | 32.24 |  | 0.34 | 19.66 | -38.19 | 38.87 |  | 0.04 | 22.78 | -44.62 | 44.69 |  | 2.94 | 14.94 | -26.33 | 32.22 |
| 2002 | 5-9 years | 2.28 | 15.27 | -27.65 | 32.22 |  | 0.34 | 19.20 | -37.30 | 37.98 |  | 0.04 | 22.53 | -44.11 | 44.19 |  | 2.94 | 14.94 | -26.34 | 32.22 |
| 2003 | 5-9 years | 2.27 | 15.27 | -27.65 | 32.19 |  | 0.34 | 18.81 | -36.54 | 37.21 |  | 0.04 | 22.34 | -43.75 | 43.83 |  | 2.92 | 14.95 | -26.38 | 32.22 |
| 2004 | 5-9 years | 2.26 | 15.26 | -27.65 | 32.17 |  | 0.34 | 18.41 | -35.74 | 36.42 |  | 0.04 | 22.46 | -43.99 | 44.07 |  | 2.95 | 14.94 | -26.33 | 32.22 |
| 2005 | 5-9 years | 2.25 | 15.26 | -27.65 | 32.15 |  | 0.34 | 18.16 | -35.26 | 35.94 |  | 0.04 | 22.48 | -44.02 | 44.09 |  | 2.99 | 14.92 | -26.24 | 32.23 |
| 2006 | 5-9 years | 2.24 | 15.26 | -27.66 | 32.15 |  | 0.34 | 18.10 | -35.14 | 35.81 |  | 0.04 | 22.18 | -43.44 | 43.52 |  | 3.06 | 14.89 | -26.12 | 32.24 |
| 2007 | 5-9 years | 2.24 | 15.27 | -27.68 | 32.16 |  | 0.34 | 18.17 | -35.28 | 35.95 |  | 0.04 | 21.10 | -41.32 | 41.39 |  | 3.07 | 14.88 | -26.10 | 32.24 |
| 2008 | 5-9 years | 2.23 | 15.27 | -27.70 | 32.16 |  | 0.33 | 18.22 | -35.38 | 36.05 |  | 0.03 | 20.32 | -39.80 | 39.86 |  | 3.08 | 14.88 | -26.10 | 32.25 |
| 2009 | 5-9 years | 2.22 | 15.27 | -27.71 | 32.16 |  | 0.33 | 18.30 | -35.54 | 36.21 |  | 0.03 | 20.03 | -39.22 | 39.29 |  | 3.10 | 14.87 | -26.05 | 32.25 |
| 2010 | 5-9 years | 2.22 | 15.28 | -27.73 | 32.16 |  | 0.33 | 18.46 | -35.85 | 36.51 |  | 0.03 | 20.07 | -39.30 | 39.36 |  | 3.11 | 14.87 | -26.04 | 32.26 |
| 2011 | 5-9 years | 2.21 | 15.29 | -27.76 | 32.18 |  | 0.33 | 18.77 | -36.47 | 37.13 |  | 0.03 | 20.36 | -39.87 | 39.94 |  | 3.12 | 14.87 | -26.01 | 32.26 |
| 2012 | 5-9 years | 2.21 | 15.31 | -27.80 | 32.21 |  | 0.33 | 19.16 | -37.23 | 37.88 |  | 0.03 | 20.97 | -41.07 | 41.14 |  | 3.20 | 14.83 | -25.87 | 32.27 |
| 2013 | 5-9 years | 2.20 | 15.32 | -27.83 | 32.23 |  | 0.32 | 19.50 | -37.90 | 38.55 |  | 0.03 | 22.17 | -43.42 | 43.48 |  | 3.26 | 14.81 | -25.77 | 32.28 |
| 2014 | 5-9 years | 2.19 | 15.33 | -27.85 | 32.24 |  | 0.32 | 19.75 | -38.40 | 39.04 |  | 0.03 | 23.63 | -46.29 | 46.36 |  | 3.27 | 14.80 | -25.74 | 32.28 |
| 2015 | 5-9 years | 2.20 | 15.33 | -27.86 | 32.25 |  | 0.32 | 19.99 | -38.86 | 39.50 |  | 0.03 | 25.19 | -49.33 | 49.40 |  | 3.28 | 14.80 | -25.72 | 32.29 |
| 2016 | 5-9 years | 2.20 | 15.35 | -27.87 | 32.28 |  | 0.32 | 20.31 | -39.49 | 40.13 |  | 0.03 | 26.54 | -51.99 | 52.06 |  | 3.33 | 14.78 | -25.64 | 32.30 |
| 2017 | 5-9 years | 2.22 | 15.38 | -27.92 | 32.36 |  | 0.32 | 20.61 | -40.08 | 40.72 |  | 0.03 | 27.39 | -53.65 | 53.72 |  | 3.28 | 14.80 | -25.72 | 32.29 |
| 2018 | 5-9 years | 2.23 | 15.40 | -27.96 | 32.42 |  | 0.32 | 20.68 | -40.22 | 40.86 |  | 0.03 | 27.52 | -53.90 | 53.96 |  | 3.18 | 14.84 | -25.91 | 32.27 |
| 2019 | 5-9 years | 2.24 | 15.40 | -27.96 | 32.43 |  | 0.32 | 20.44 | -39.75 | 40.39 |  | 0.03 | 27.33 | -53.54 | 53.60 |  | 3.11 | 14.88 | -26.05 | 32.27 |
| 2020 | 5-9 years | 2.23 | 15.38 | -27.92 | 32.37 |  | 0.32 | 19.92 | -38.72 | 39.36 |  | 0.03 | 26.56 | -52.02 | 52.08 |  | 2.99 | 14.93 | -26.27 | 32.26 |
| 2021 | 5-9 years | 2.23 | 15.36 | -27.88 | 32.33 |  | 0.32 | 19.45 | -37.80 | 38.44 |  | 0.03 | 24.50 | -47.99 | 48.05 |  | 2.88 | 14.98 | -26.48 | 32.25 |
| 2022 | 5-9 years | 2.25 | 18.11 | -33.25 | 37.74 |  | 0.32 | 23.51 | -45.76 | 46.40 |  | 0.02 | 21.50 | -42.12 | 42.17 |  | 2.07 | 17.48 | -32.18 | 36.33 |
| 2023 | 5-9 years | 2.26 | 18.13 | -33.27 | 37.79 |  | 0.33 | 24.19 | -47.08 | 47.74 |  | 0.02 | 20.41 | -39.98 | 40.03 |  | 1.92 | 17.88 | -33.11 | 36.96 |
| 2024 | 5-9 years | 2.27 | 18.33 | -33.66 | 38.20 |  | 0.33 | 25.34 | -49.33 | 49.99 |  | 0.02 | 19.54 | -38.28 | 38.32 |  | 1.78 | 18.38 | -34.24 | 37.80 |
| 2025 | 5-9 years | 2.28 | 18.58 | -34.13 | 38.70 |  | 0.33 | 25.80 | -50.23 | 50.89 |  | 0.02 | 18.41 | -36.06 | 36.09 |  | 1.65 | 18.63 | -34.88 | 38.17 |
| 2026 | 5-9 years | 2.30 | 18.63 | -34.22 | 38.81 |  | 0.33 | 24.87 | -48.41 | 49.08 |  | 0.02 | 17.17 | -33.64 | 33.68 |  | 1.52 | 17.81 | -33.39 | 36.43 |
| 2027 | 5-9 years | 2.31 | 19.31 | -35.53 | 40.16 |  | 0.34 | 24.98 | -48.63 | 49.30 |  | 0.02 | 16.25 | -31.83 | 31.87 |  | 1.41 | 15.87 | -29.70 | 32.51 |
| 2028 | 5-9 years | 2.32 | 22.28 | -41.34 | 45.99 |  | 0.34 | 28.26 | -55.05 | 55.72 |  | 0.01 | 15.84 | -31.04 | 31.07 |  | 1.30 | 14.43 | -26.98 | 29.58 |
| 2029 | 5-9 years | 2.34 | 27.52 | -51.60 | 56.28 |  | 0.34 | 33.58 | -65.47 | 66.15 |  | 0.01 | 15.88 | -31.12 | 31.15 |  | 1.20 | 14.43 | -27.08 | 29.49 |
| 2030 | 5-9 years | 2.35 | 33.73 | -63.77 | 68.47 |  | 0.34 | 39.30 | -76.68 | 77.37 |  | 0.01 | 16.24 | -31.82 | 31.84 |  | 1.11 | 16.44 | -31.12 | 33.35 |
| 2031 | 5-9 years | 2.37 | 39.78 | -75.61 | 80.34 |  | 0.35 | 44.53 | -86.93 | 87.62 |  | 0.01 | 16.79 | -32.89 | 32.91 |  | 1.03 | 19.92 | -38.00 | 40.07 |
| 2032 | 5-9 years | 2.38 | 45.12 | -86.05 | 90.82 |  | 0.35 | 48.97 | -95.64 | 96.34 |  | 0.01 | 17.44 | -34.17 | 34.19 |  | 0.96 | 24.21 | -46.49 | 48.41 |
| 2033 | 5-9 years | 2.40 | 49.60 | -94.82 | 99.62 |  | 0.35 | 52.63 | -102.81 | 103.51 |  | 0.01 | 19.13 | -37.49 | 37.51 |  | 0.89 | 28.79 | -55.54 | 57.32 |
| 2034 | 5-9 years | 2.41 | 53.27 | -102.00 | 106.83 |  | 0.35 | 55.59 | -108.61 | 109.31 |  | 0.01 | 20.91 | -40.98 | 40.99 |  | 0.82 | 33.31 | -64.46 | 66.11 |
| 2035 | 5-9 years | 2.43 | 56.24 | -107.79 | 112.66 |  | 0.36 | 57.96 | -113.24 | 113.96 |  | 0.01 | 22.65 | -44.39 | 44.40 |  | 0.76 | 37.57 | -72.87 | 74.40 |
| 1990 | 10-14 years | 2.55 | 15.41 | -27.65 | 32.76 |  | 0.33 | 27.04 | -52.67 | 53.34 |  | 0.01 | 23.81 | -46.67 | 46.68 |  | 1.08 | 16.38 | -31.03 | 33.18 |
| 1991 | 10-14 years | 2.56 | 15.41 | -27.64 | 32.75 |  | 0.33 | 27.11 | -52.80 | 53.47 |  | 0.01 | 23.91 | -46.86 | 46.88 |  | 1.07 | 16.38 | -31.04 | 33.18 |
| 1992 | 10-14 years | 2.56 | 15.40 | -27.63 | 32.74 |  | 0.33 | 26.72 | -52.04 | 52.71 |  | 0.01 | 24.06 | -47.15 | 47.17 |  | 1.07 | 16.39 | -31.05 | 33.19 |
| 1993 | 10-14 years | 2.55 | 15.40 | -27.63 | 32.73 |  | 0.34 | 26.37 | -51.35 | 52.02 |  | 0.01 | 24.15 | -47.33 | 47.35 |  | 1.07 | 16.39 | -31.07 | 33.20 |
| 1994 | 10-14 years | 2.54 | 15.40 | -27.64 | 32.72 |  | 0.33 | 26.05 | -50.73 | 51.40 |  | 0.01 | 24.11 | -47.24 | 47.26 |  | 1.05 | 16.41 | -31.12 | 33.22 |
| 1995 | 10-14 years | 2.52 | 15.39 | -27.65 | 32.69 |  | 0.33 | 25.59 | -49.81 | 50.48 |  | 0.01 | 23.93 | -46.89 | 46.91 |  | 1.03 | 16.44 | -31.19 | 33.25 |
| 1996 | 10-14 years | 2.49 | 15.38 | -27.65 | 32.64 |  | 0.33 | 24.77 | -48.23 | 48.89 |  | 0.01 | 23.70 | -46.44 | 46.46 |  | 1.00 | 16.48 | -31.30 | 33.30 |
| 1997 | 10-14 years | 2.45 | 15.35 | -27.63 | 32.53 |  | 0.33 | 23.42 | -45.58 | 46.24 |  | 0.01 | 23.43 | -45.92 | 45.94 |  | 0.97 | 16.53 | -31.42 | 33.36 |
| 1998 | 10-14 years | 2.40 | 15.30 | -27.59 | 32.40 |  | 0.33 | 21.97 | -42.74 | 43.39 |  | 0.01 | 23.23 | -45.52 | 45.54 |  | 0.93 | 16.58 | -31.56 | 33.42 |
| 1999 | 10-14 years | 2.37 | 15.25 | -27.53 | 32.26 |  | 0.32 | 20.61 | -40.08 | 40.73 |  | 0.01 | 23.36 | -45.78 | 45.80 |  | 0.93 | 16.57 | -31.56 | 33.42 |
| 2000 | 10-14 years | 2.35 | 15.23 | -27.51 | 32.20 |  | 0.32 | 19.61 | -38.12 | 38.76 |  | 0.01 | 23.58 | -46.20 | 46.22 |  | 0.93 | 16.57 | -31.55 | 33.41 |
| 2001 | 10-14 years | 2.34 | 15.22 | -27.49 | 32.17 |  | 0.32 | 19.03 | -36.98 | 37.63 |  | 0.01 | 23.74 | -46.52 | 46.54 |  | 0.92 | 16.58 | -31.59 | 33.42 |
| 2002 | 10-14 years | 2.33 | 15.21 | -27.48 | 32.14 |  | 0.32 | 18.53 | -35.99 | 36.64 |  | 0.01 | 23.77 | -46.57 | 46.59 |  | 0.89 | 16.61 | -31.66 | 33.44 |
| 2003 | 10-14 years | 2.33 | 15.20 | -27.46 | 32.12 |  | 0.33 | 18.13 | -35.21 | 35.86 |  | 0.01 | 23.74 | -46.51 | 46.53 |  | 0.87 | 16.64 | -31.75 | 33.48 |
| 2004 | 10-14 years | 2.33 | 15.19 | -27.45 | 32.10 |  | 0.33 | 17.86 | -34.67 | 35.32 |  | 0.01 | 23.70 | -46.45 | 46.47 |  | 0.86 | 16.66 | -31.79 | 33.50 |
| 2005 | 10-14 years | 2.33 | 15.19 | -27.44 | 32.10 |  | 0.33 | 17.69 | -34.34 | 34.99 |  | 0.01 | 23.63 | -46.31 | 46.33 |  | 0.85 | 16.66 | -31.80 | 33.51 |
| 2006 | 10-14 years | 2.33 | 15.19 | -27.44 | 32.10 |  | 0.33 | 17.58 | -34.13 | 34.79 |  | 0.01 | 23.63 | -46.30 | 46.32 |  | 0.83 | 16.69 | -31.88 | 33.55 |
| 2007 | 10-14 years | 2.32 | 15.19 | -27.46 | 32.10 |  | 0.33 | 17.58 | -34.12 | 34.77 |  | 0.01 | 23.59 | -46.22 | 46.24 |  | 0.82 | 16.72 | -31.95 | 33.58 |
| 2008 | 10-14 years | 2.32 | 15.20 | -27.47 | 32.10 |  | 0.33 | 17.62 | -34.20 | 34.85 |  | 0.01 | 23.53 | -46.11 | 46.13 |  | 0.81 | 16.74 | -32.00 | 33.61 |
| 2009 | 10-14 years | 2.31 | 15.20 | -27.48 | 32.10 |  | 0.32 | 17.73 | -34.44 | 35.09 |  | 0.01 | 23.60 | -46.25 | 46.27 |  | 0.81 | 16.74 | -32.01 | 33.62 |
| 2010 | 10-14 years | 2.31 | 15.21 | -27.49 | 32.12 |  | 0.32 | 18.03 | -35.02 | 35.67 |  | 0.01 | 23.63 | -46.31 | 46.33 |  | 0.80 | 16.76 | -32.06 | 33.65 |
| 2011 | 10-14 years | 2.31 | 15.22 | -27.53 | 32.15 |  | 0.32 | 18.66 | -36.26 | 36.90 |  | 0.01 | 23.46 | -45.96 | 45.98 |  | 0.77 | 16.81 | -32.17 | 33.71 |
| 2012 | 10-14 years | 2.30 | 15.25 | -27.59 | 32.20 |  | 0.32 | 19.52 | -37.93 | 38.57 |  | 0.01 | 22.91 | -44.90 | 44.92 |  | 0.74 | 16.86 | -32.30 | 33.78 |
| 2013 | 10-14 years | 2.30 | 15.28 | -27.64 | 32.24 |  | 0.32 | 20.40 | -39.67 | 40.30 |  | 0.01 | 22.57 | -44.24 | 44.26 |  | 0.73 | 16.88 | -32.36 | 33.82 |
| 2014 | 10-14 years | 2.29 | 15.30 | -27.70 | 32.29 |  | 0.31 | 21.18 | -41.19 | 41.82 |  | 0.01 | 22.41 | -43.91 | 43.93 |  | 0.72 | 16.89 | -32.39 | 33.84 |
| 2015 | 10-14 years | 2.29 | 15.34 | -27.77 | 32.35 |  | 0.31 | 21.76 | -42.33 | 42.95 |  | 0.01 | 22.43 | -43.96 | 43.98 |  | 0.71 | 16.92 | -32.44 | 33.87 |
| 2016 | 10-14 years | 2.30 | 15.39 | -27.86 | 32.45 |  | 0.31 | 22.49 | -43.76 | 44.38 |  | 0.01 | 22.59 | -44.27 | 44.29 |  | 0.70 | 16.94 | -32.49 | 33.90 |
| 2017 | 10-14 years | 2.31 | 15.45 | -27.97 | 32.59 |  | 0.31 | 23.38 | -45.51 | 46.13 |  | 0.01 | 22.87 | -44.81 | 44.83 |  | 0.69 | 16.96 | -32.55 | 33.93 |
| 2018 | 10-14 years | 2.32 | 15.49 | -28.04 | 32.69 |  | 0.31 | 24.04 | -46.80 | 47.42 |  | 0.01 | 23.31 | -45.67 | 45.69 |  | 0.67 | 16.99 | -32.62 | 33.97 |
| 2019 | 10-14 years | 2.33 | 15.51 | -28.06 | 32.72 |  | 0.31 | 24.25 | -47.22 | 47.83 |  | 0.01 | 23.93 | -46.90 | 46.91 |  | 0.66 | 17.02 | -32.69 | 34.01 |
| 2020 | 10-14 years | 2.33 | 15.50 | -28.05 | 32.71 |  | 0.31 | 23.99 | -46.72 | 47.33 |  | 0.01 | 24.61 | -48.22 | 48.24 |  | 0.63 | 17.07 | -32.82 | 34.07 |
| 2021 | 10-14 years | 2.34 | 15.50 | -28.04 | 32.72 |  | 0.31 | 24.00 | -46.73 | 47.35 |  | 0.01 | 25.30 | -49.58 | 49.60 |  | 0.62 | 17.08 | -32.87 | 34.10 |
| 2022 | 10-14 years | 2.26 | 18.38 | -33.75 | 38.28 |  | 0.30 | 25.01 | -48.72 | 49.33 |  | 0.01 | 26.17 | -51.28 | 51.30 |  | 0.68 | 19.04 | -36.64 | 38.01 |
| 2023 | 10-14 years | 2.27 | 18.44 | -33.88 | 38.42 |  | 0.30 | 26.11 | -50.86 | 51.47 |  | 0.01 | 27.17 | -53.24 | 53.26 |  | 0.67 | 19.05 | -36.67 | 38.01 |
| 2024 | 10-14 years | 2.27 | 18.77 | -34.51 | 39.06 |  | 0.31 | 28.67 | -55.88 | 56.49 |  | 0.01 | 28.33 | -55.52 | 55.54 |  | 0.65 | 19.29 | -37.16 | 38.45 |
| 2025 | 10-14 years | 2.28 | 19.33 | -35.60 | 40.16 |  | 0.31 | 32.10 | -62.61 | 63.23 |  | 0.01 | 29.49 | -57.80 | 57.82 |  | 0.62 | 19.82 | -38.23 | 39.46 |
| 2026 | 10-14 years | 2.29 | 20.04 | -36.99 | 41.57 |  | 0.31 | 35.74 | -69.74 | 70.35 |  | 0.01 | 30.15 | -59.09 | 59.11 |  | 0.58 | 20.65 | -39.90 | 41.06 |
| 2027 | 10-14 years | 2.30 | 20.87 | -38.60 | 43.20 |  | 0.31 | 39.09 | -76.30 | 76.92 |  | 0.01 | 29.65 | -58.11 | 58.12 |  | 0.54 | 21.66 | -41.92 | 43.00 |
| 2028 | 10-14 years | 2.31 | 21.87 | -40.55 | 45.18 |  | 0.31 | 42.28 | -82.56 | 83.18 |  | 0.01 | 28.03 | -54.93 | 54.94 |  | 0.50 | 22.69 | -43.96 | 44.97 |
| 2029 | 10-14 years | 2.33 | 22.94 | -42.63 | 47.28 |  | 0.31 | 44.53 | -86.97 | 87.59 |  | 0.01 | 25.48 | -49.93 | 49.94 |  | 0.46 | 23.70 | -45.98 | 46.91 |
| 2030 | 10-14 years | 2.34 | 23.58 | -43.88 | 48.56 |  | 0.32 | 43.60 | -85.15 | 85.78 |  | 0.00 | 22.41 | -43.92 | 43.93 |  | 0.43 | 24.21 | -47.02 | 47.88 |
| 2031 | 10-14 years | 2.35 | 23.06 | -42.85 | 47.55 |  | 0.32 | 38.08 | -74.33 | 74.96 |  | 0.00 | 20.54 | -40.25 | 40.26 |  | 0.40 | 23.22 | -45.11 | 45.90 |
| 2032 | 10-14 years | 2.37 | 22.14 | -41.02 | 45.75 |  | 0.32 | 32.05 | -62.51 | 63.15 |  | 0.00 | 19.01 | -37.26 | 37.27 |  | 0.37 | 20.62 | -40.05 | 40.79 |
| 2033 | 10-14 years | 2.38 | 23.28 | -43.25 | 48.02 |  | 0.32 | 30.64 | -59.74 | 60.38 |  | 0.00 | 18.15 | -35.58 | 35.58 |  | 0.34 | 18.23 | -35.38 | 36.06 |
| 2034 | 10-14 years | 2.39 | 27.18 | -50.87 | 55.66 |  | 0.33 | 33.41 | -65.16 | 65.81 |  | 0.00 | 17.90 | -35.08 | 35.09 |  | 0.31 | 17.67 | -34.31 | 34.94 |
| 2035 | 10-14 years | 2.41 | 32.66 | -61.61 | 66.43 |  | 0.33 | 38.05 | -74.25 | 74.91 |  | 0.00 | 18.08 | -35.44 | 35.45 |  | 0.29 | 19.38 | -37.69 | 38.27 |
| 1990 | 15-19 years | 2.91 | 15.11 | -26.71 | 32.53 |  | 0.37 | 26.00 | -50.59 | 51.33 |  | 0.02 | 23.91 | -46.85 | 46.89 |  | 1.64 | 15.77 | -29.27 | 32.56 |
| 1991 | 15-19 years | 2.92 | 15.13 | -26.74 | 32.58 |  | 0.37 | 26.43 | -51.43 | 52.18 |  | 0.02 | 23.94 | -46.91 | 46.94 |  | 1.65 | 15.77 | -29.25 | 32.55 |
| 1992 | 15-19 years | 2.93 | 15.14 | -26.75 | 32.61 |  | 0.38 | 26.31 | -51.19 | 51.94 |  | 0.02 | 24.03 | -47.08 | 47.11 |  | 1.66 | 15.76 | -29.23 | 32.54 |
| 1993 | 15-19 years | 2.94 | 15.15 | -26.76 | 32.63 |  | 0.38 | 26.13 | -50.83 | 51.59 |  | 0.02 | 24.18 | -47.37 | 47.41 |  | 1.68 | 15.74 | -29.16 | 32.53 |
| 1994 | 15-19 years | 2.94 | 15.15 | -26.75 | 32.63 |  | 0.38 | 25.83 | -50.25 | 51.00 |  | 0.02 | 24.34 | -47.69 | 47.73 |  | 1.68 | 15.74 | -29.17 | 32.53 |
| 1995 | 15-19 years | 2.94 | 15.13 | -26.72 | 32.60 |  | 0.38 | 25.29 | -49.18 | 49.94 |  | 0.02 | 24.52 | -48.03 | 48.07 |  | 1.67 | 15.74 | -29.18 | 32.53 |
| 1996 | 15-19 years | 2.92 | 15.10 | -26.67 | 32.51 |  | 0.38 | 24.13 | -46.91 | 47.67 |  | 0.02 | 24.60 | -48.20 | 48.24 |  | 1.65 | 15.76 | -29.24 | 32.54 |
| 1997 | 15-19 years | 2.88 | 15.04 | -26.60 | 32.36 |  | 0.38 | 22.27 | -43.27 | 44.03 |  | 0.02 | 24.78 | -48.55 | 48.58 |  | 1.63 | 15.78 | -29.29 | 32.55 |
| 1998 | 15-19 years | 2.83 | 14.98 | -26.54 | 32.20 |  | 0.38 | 20.39 | -39.58 | 40.34 |  | 0.02 | 24.71 | -48.41 | 48.45 |  | 1.59 | 15.81 | -29.40 | 32.58 |
| 1999 | 15-19 years | 2.78 | 14.97 | -26.56 | 32.11 |  | 0.38 | 18.85 | -36.56 | 37.31 |  | 0.02 | 24.54 | -48.08 | 48.12 |  | 1.58 | 15.82 | -29.43 | 32.59 |
| 2000 | 15-19 years | 2.75 | 14.96 | -26.58 | 32.07 |  | 0.37 | 17.91 | -34.72 | 35.47 |  | 0.02 | 24.03 | -47.07 | 47.11 |  | 1.55 | 15.85 | -29.51 | 32.61 |
| 2001 | 15-19 years | 2.72 | 14.97 | -26.62 | 32.07 |  | 0.37 | 17.57 | -34.06 | 34.80 |  | 0.02 | 23.49 | -46.02 | 46.05 |  | 1.50 | 15.89 | -29.65 | 32.65 |
| 2002 | 15-19 years | 2.70 | 14.98 | -26.66 | 32.07 |  | 0.37 | 17.37 | -33.67 | 34.41 |  | 0.02 | 22.87 | -44.80 | 44.84 |  | 1.45 | 15.94 | -29.78 | 32.69 |
| 2003 | 15-19 years | 2.69 | 14.99 | -26.69 | 32.07 |  | 0.37 | 17.26 | -33.45 | 34.19 |  | 0.02 | 22.50 | -44.09 | 44.13 |  | 1.42 | 15.96 | -29.87 | 32.71 |
| 2004 | 15-19 years | 2.68 | 15.00 | -26.71 | 32.07 |  | 0.37 | 17.19 | -33.33 | 34.07 |  | 0.02 | 22.66 | -44.39 | 44.42 |  | 1.40 | 15.98 | -29.92 | 32.73 |
| 2005 | 15-19 years | 2.68 | 14.99 | -26.71 | 32.07 |  | 0.37 | 17.10 | -33.15 | 33.89 |  | 0.02 | 23.04 | -45.15 | 45.18 |  | 1.38 | 16.00 | -29.97 | 32.74 |
| 2006 | 15-19 years | 2.68 | 14.99 | -26.70 | 32.06 |  | 0.37 | 17.04 | -33.02 | 33.76 |  | 0.02 | 23.39 | -45.82 | 45.86 |  | 1.36 | 16.02 | -30.05 | 32.76 |
| 2007 | 15-19 years | 2.69 | 14.99 | -26.68 | 32.06 |  | 0.37 | 17.01 | -32.97 | 33.71 |  | 0.02 | 23.57 | -46.18 | 46.21 |  | 1.33 | 16.04 | -30.11 | 32.78 |
| 2008 | 15-19 years | 2.69 | 14.98 | -26.68 | 32.06 |  | 0.37 | 17.03 | -33.00 | 33.74 |  | 0.02 | 23.58 | -46.19 | 46.23 |  | 1.33 | 16.05 | -30.13 | 32.79 |
| 2009 | 15-19 years | 2.70 | 14.99 | -26.67 | 32.08 |  | 0.37 | 17.17 | -33.28 | 34.02 |  | 0.02 | 23.60 | -46.24 | 46.27 |  | 1.32 | 16.06 | -30.14 | 32.79 |
| 2010 | 15-19 years | 2.71 | 15.00 | -26.68 | 32.11 |  | 0.37 | 17.59 | -34.11 | 34.85 |  | 0.02 | 23.63 | -46.29 | 46.33 |  | 1.31 | 16.07 | -30.18 | 32.81 |
| 2011 | 15-19 years | 2.72 | 15.01 | -26.70 | 32.15 |  | 0.37 | 18.46 | -35.81 | 36.55 |  | 0.02 | 23.74 | -46.50 | 46.54 |  | 1.30 | 16.08 | -30.21 | 32.82 |
| 2012 | 15-19 years | 2.73 | 15.03 | -26.74 | 32.20 |  | 0.37 | 19.62 | -38.09 | 38.83 |  | 0.02 | 23.72 | -46.48 | 46.51 |  | 1.27 | 16.11 | -30.31 | 32.85 |
| 2013 | 15-19 years | 2.73 | 15.07 | -26.80 | 32.27 |  | 0.36 | 20.80 | -40.40 | 41.13 |  | 0.02 | 23.66 | -46.35 | 46.38 |  | 1.26 | 16.13 | -30.36 | 32.87 |
| 2014 | 15-19 years | 2.74 | 15.11 | -26.88 | 32.35 |  | 0.36 | 21.86 | -42.49 | 43.21 |  | 0.02 | 23.70 | -46.43 | 46.46 |  | 1.24 | 16.15 | -30.41 | 32.89 |
| 2015 | 15-19 years | 2.74 | 15.15 | -26.95 | 32.42 |  | 0.36 | 22.77 | -44.27 | 44.99 |  | 0.02 | 23.67 | -46.37 | 46.40 |  | 1.21 | 16.18 | -30.49 | 32.92 |
| 2016 | 15-19 years | 2.75 | 15.19 | -27.03 | 32.53 |  | 0.36 | 23.82 | -46.33 | 47.04 |  | 0.02 | 23.39 | -45.83 | 45.86 |  | 1.20 | 16.19 | -30.53 | 32.94 |
| 2017 | 15-19 years | 2.76 | 15.25 | -27.12 | 32.65 |  | 0.36 | 24.95 | -48.54 | 49.26 |  | 0.02 | 22.74 | -44.56 | 44.59 |  | 1.19 | 16.21 | -30.58 | 32.96 |
| 2018 | 15-19 years | 2.78 | 15.29 | -27.19 | 32.75 |  | 0.36 | 25.84 | -50.28 | 50.99 |  | 0.02 | 22.01 | -43.12 | 43.15 |  | 1.15 | 16.25 | -30.69 | 33.00 |
| 2019 | 15-19 years | 2.78 | 15.31 | -27.24 | 32.80 |  | 0.36 | 26.24 | -51.08 | 51.80 |  | 0.01 | 21.80 | -42.71 | 42.74 |  | 1.13 | 16.28 | -30.78 | 33.04 |
| 2020 | 15-19 years | 2.78 | 15.32 | -27.26 | 32.81 |  | 0.35 | 26.19 | -50.98 | 51.69 |  | 0.01 | 21.82 | -42.76 | 42.79 |  | 1.11 | 16.31 | -30.86 | 33.07 |
| 2021 | 15-19 years | 2.77 | 15.33 | -27.28 | 32.83 |  | 0.35 | 26.29 | -51.17 | 51.87 |  | 0.01 | 21.99 | -43.09 | 43.12 |  | 1.10 | 16.32 | -30.89 | 33.08 |
| 2022 | 15-19 years | 2.61 | 18.14 | -32.95 | 38.16 |  | 0.34 | 25.33 | -49.30 | 49.99 |  | 0.01 | 23.12 | -45.30 | 45.33 |  | 1.14 | 18.75 | -35.61 | 37.88 |
| 2023 | 15-19 years | 2.60 | 18.17 | -33.01 | 38.21 |  | 0.34 | 26.46 | -51.51 | 52.20 |  | 0.01 | 25.18 | -49.34 | 49.36 |  | 1.13 | 18.82 | -35.76 | 38.02 |
| 2024 | 15-19 years | 2.60 | 18.40 | -33.47 | 38.67 |  | 0.34 | 29.29 | -57.06 | 57.75 |  | 0.01 | 27.97 | -54.81 | 54.83 |  | 1.12 | 19.05 | -36.21 | 38.45 |
| 2025 | 15-19 years | 2.60 | 18.87 | -34.38 | 39.58 |  | 0.34 | 33.25 | -64.83 | 65.52 |  | 0.01 | 31.20 | -61.13 | 61.16 |  | 1.11 | 19.39 | -36.90 | 39.12 |
| 2026 | 15-19 years | 2.61 | 19.59 | -35.79 | 41.00 |  | 0.34 | 37.82 | -73.78 | 74.47 |  | 0.01 | 34.56 | -67.72 | 67.75 |  | 1.10 | 19.83 | -37.77 | 39.97 |
| 2027 | 15-19 years | 2.61 | 20.59 | -37.75 | 42.97 |  | 0.34 | 42.56 | -83.07 | 83.76 |  | 0.01 | 37.78 | -74.03 | 74.05 |  | 1.08 | 20.37 | -38.85 | 41.01 |
| 2028 | 15-19 years | 2.62 | 21.86 | -40.24 | 45.47 |  | 0.34 | 47.56 | -92.88 | 93.56 |  | 0.01 | 40.82 | -79.99 | 80.02 |  | 1.06 | 21.09 | -40.28 | 42.39 |
| 2029 | 15-19 years | 2.62 | 23.39 | -43.23 | 48.47 |  | 0.34 | 52.72 | -102.98 | 103.67 |  | 0.01 | 43.82 | -85.88 | 85.91 |  | 1.02 | 22.07 | -42.23 | 44.27 |
| 2030 | 15-19 years | 2.63 | 25.09 | -46.56 | 51.81 |  | 0.34 | 57.56 | -112.48 | 113.17 |  | 0.01 | 46.54 | -91.20 | 91.23 |  | 0.97 | 23.43 | -44.94 | 46.89 |
| 2031 | 15-19 years | 2.64 | 26.82 | -49.93 | 55.21 |  | 0.35 | 61.50 | -120.20 | 120.89 |  | 0.01 | 47.76 | -93.60 | 93.62 |  | 0.92 | 25.19 | -48.46 | 50.29 |
| 2032 | 15-19 years | 2.65 | 28.49 | -53.18 | 58.48 |  | 0.35 | 64.32 | -125.71 | 126.41 |  | 0.01 | 45.92 | -90.00 | 90.02 |  | 0.86 | 27.12 | -52.30 | 54.01 |
| 2033 | 15-19 years | 2.67 | 30.22 | -56.57 | 61.90 |  | 0.35 | 66.74 | -130.45 | 131.15 |  | 0.01 | 41.19 | -80.71 | 80.73 |  | 0.79 | 28.85 | -55.76 | 57.35 |
| 2034 | 15-19 years | 2.68 | 31.81 | -59.67 | 65.03 |  | 0.35 | 67.85 | -132.63 | 133.34 |  | 0.01 | 34.79 | -68.19 | 68.21 |  | 0.73 | 30.20 | -58.45 | 59.92 |
| 2035 | 15-19 years | 2.70 | 32.35 | -60.70 | 66.09 |  | 0.35 | 64.70 | -126.46 | 127.17 |  | 0.01 | 28.02 | -54.92 | 54.93 |  | 0.68 | 30.50 | -59.09 | 60.45 |
| 1990 | 20-24 years | 3.40 | 14.77 | -25.55 | 32.36 |  | 0.44 | 23.58 | -45.78 | 46.65 |  | 0.04 | 22.77 | -44.58 | 44.66 |  | 3.01 | 14.90 | -26.20 | 32.22 |
| 1991 | 20-24 years | 3.43 | 14.80 | -25.57 | 32.44 |  | 0.44 | 24.23 | -47.05 | 47.93 |  | 0.04 | 23.00 | -45.04 | 45.13 |  | 3.03 | 14.89 | -26.15 | 32.22 |
| 1992 | 20-24 years | 3.45 | 14.81 | -25.57 | 32.48 |  | 0.44 | 24.29 | -47.16 | 48.05 |  | 0.04 | 23.27 | -45.56 | 45.65 |  | 3.08 | 14.87 | -26.07 | 32.23 |
| 1993 | 20-24 years | 3.46 | 14.82 | -25.58 | 32.51 |  | 0.44 | 24.36 | -47.31 | 48.19 |  | 0.04 | 23.47 | -45.95 | 46.04 |  | 3.11 | 14.86 | -26.01 | 32.23 |
| 1994 | 20-24 years | 3.47 | 14.83 | -25.60 | 32.54 |  | 0.44 | 24.46 | -47.50 | 48.39 |  | 0.04 | 23.72 | -46.45 | 46.54 |  | 3.16 | 14.83 | -25.91 | 32.24 |
| 1995 | 20-24 years | 3.47 | 14.84 | -25.61 | 32.55 |  | 0.44 | 24.34 | -47.26 | 48.15 |  | 0.05 | 23.85 | -46.69 | 46.78 |  | 3.19 | 14.82 | -25.86 | 32.24 |
| 1996 | 20-24 years | 3.45 | 14.82 | -25.61 | 32.51 |  | 0.44 | 23.52 | -45.66 | 46.54 |  | 0.05 | 23.80 | -46.60 | 46.69 |  | 3.20 | 14.82 | -25.84 | 32.24 |
| 1997 | 20-24 years | 3.41 | 14.79 | -25.57 | 32.40 |  | 0.44 | 21.91 | -42.50 | 43.39 |  | 0.05 | 23.77 | -46.54 | 46.63 |  | 3.23 | 14.81 | -25.79 | 32.25 |
| 1998 | 20-24 years | 3.37 | 14.74 | -25.53 | 32.26 |  | 0.44 | 20.17 | -39.10 | 39.98 |  | 0.05 | 23.76 | -46.52 | 46.61 |  | 3.25 | 14.80 | -25.75 | 32.25 |
| 1999 | 20-24 years | 3.33 | 14.72 | -25.53 | 32.18 |  | 0.44 | 18.60 | -36.01 | 36.90 |  | 0.05 | 24.01 | -47.02 | 47.11 |  | 3.30 | 14.78 | -25.66 | 32.26 |
| 2000 | 20-24 years | 3.31 | 14.71 | -25.52 | 32.14 |  | 0.44 | 17.62 | -34.10 | 34.99 |  | 0.05 | 23.94 | -46.89 | 46.98 |  | 3.31 | 14.77 | -25.63 | 32.26 |
| 2001 | 20-24 years | 3.31 | 14.70 | -25.51 | 32.12 |  | 0.44 | 17.13 | -33.13 | 34.01 |  | 0.05 | 23.90 | -46.80 | 46.89 |  | 3.31 | 14.77 | -25.64 | 32.26 |
| 2002 | 20-24 years | 3.30 | 14.70 | -25.52 | 32.12 |  | 0.44 | 16.91 | -32.71 | 33.59 |  | 0.05 | 23.76 | -46.52 | 46.61 |  | 3.27 | 14.79 | -25.71 | 32.26 |
| 2003 | 20-24 years | 3.30 | 14.71 | -25.53 | 32.13 |  | 0.44 | 16.88 | -32.65 | 33.54 |  | 0.05 | 23.54 | -46.09 | 46.18 |  | 3.23 | 14.81 | -25.79 | 32.25 |
| 2004 | 20-24 years | 3.30 | 14.72 | -25.55 | 32.15 |  | 0.44 | 16.92 | -32.71 | 33.60 |  | 0.05 | 23.30 | -45.62 | 45.71 |  | 3.20 | 14.82 | -25.84 | 32.25 |
| 2005 | 20-24 years | 3.29 | 14.73 | -25.58 | 32.16 |  | 0.44 | 16.94 | -32.77 | 33.65 |  | 0.05 | 22.63 | -44.32 | 44.41 |  | 3.16 | 14.84 | -25.93 | 32.25 |
| 2006 | 20-24 years | 3.28 | 14.74 | -25.61 | 32.17 |  | 0.44 | 17.05 | -32.98 | 33.86 |  | 0.04 | 21.98 | -43.03 | 43.12 |  | 3.09 | 14.87 | -26.06 | 32.24 |
| 2007 | 20-24 years | 3.27 | 14.75 | -25.65 | 32.18 |  | 0.44 | 17.16 | -33.20 | 34.08 |  | 0.04 | 21.23 | -41.57 | 41.65 |  | 3.04 | 14.89 | -26.15 | 32.24 |
| 2008 | 20-24 years | 3.25 | 14.76 | -25.68 | 32.18 |  | 0.44 | 17.24 | -33.36 | 34.23 |  | 0.04 | 20.68 | -40.49 | 40.57 |  | 2.98 | 14.92 | -26.27 | 32.23 |
| 2009 | 20-24 years | 3.24 | 14.77 | -25.71 | 32.19 |  | 0.43 | 17.31 | -33.49 | 34.36 |  | 0.04 | 21.07 | -41.25 | 41.34 |  | 2.96 | 14.94 | -26.32 | 32.23 |
| 2010 | 20-24 years | 3.24 | 14.77 | -25.71 | 32.19 |  | 0.43 | 17.58 | -34.02 | 34.89 |  | 0.04 | 21.89 | -42.86 | 42.94 |  | 2.94 | 14.95 | -26.35 | 32.23 |
| 2011 | 20-24 years | 3.25 | 14.78 | -25.71 | 32.21 |  | 0.43 | 18.32 | -35.48 | 36.34 |  | 0.04 | 22.67 | -44.39 | 44.48 |  | 2.94 | 14.94 | -26.35 | 32.23 |
| 2012 | 20-24 years | 3.27 | 14.78 | -25.71 | 32.25 |  | 0.43 | 19.38 | -37.56 | 38.42 |  | 0.04 | 23.21 | -45.44 | 45.53 |  | 2.95 | 14.94 | -26.33 | 32.23 |
| 2013 | 20-24 years | 3.28 | 14.80 | -25.73 | 32.30 |  | 0.43 | 20.50 | -39.76 | 40.62 |  | 0.04 | 23.38 | -45.79 | 45.88 |  | 2.98 | 14.92 | -26.27 | 32.23 |
| 2014 | 20-24 years | 3.30 | 14.83 | -25.77 | 32.37 |  | 0.43 | 21.64 | -41.99 | 42.85 |  | 0.04 | 23.47 | -45.96 | 46.04 |  | 3.02 | 14.91 | -26.20 | 32.24 |
| 2015 | 20-24 years | 3.31 | 14.86 | -25.81 | 32.44 |  | 0.43 | 22.55 | -43.76 | 44.62 |  | 0.04 | 23.54 | -46.10 | 46.18 |  | 3.04 | 14.90 | -26.16 | 32.24 |
| 2016 | 20-24 years | 3.33 | 14.89 | -25.86 | 32.52 |  | 0.43 | 23.51 | -45.66 | 46.52 |  | 0.04 | 23.83 | -46.67 | 46.76 |  | 3.13 | 14.86 | -25.99 | 32.26 |
| 2017 | 20-24 years | 3.35 | 14.93 | -25.92 | 32.61 |  | 0.43 | 24.52 | -47.64 | 48.49 |  | 0.04 | 23.88 | -46.76 | 46.84 |  | 3.13 | 14.86 | -26.00 | 32.26 |
| 2018 | 20-24 years | 3.36 | 14.96 | -25.96 | 32.68 |  | 0.43 | 25.32 | -49.21 | 50.06 |  | 0.04 | 23.69 | -46.40 | 46.48 |  | 3.12 | 14.86 | -26.01 | 32.26 |
| 2019 | 20-24 years | 3.37 | 14.98 | -25.99 | 32.73 |  | 0.43 | 25.78 | -50.09 | 50.94 |  | 0.04 | 23.72 | -46.46 | 46.54 |  | 3.14 | 14.86 | -25.98 | 32.26 |
| 2020 | 20-24 years | 3.37 | 15.00 | -26.02 | 32.76 |  | 0.42 | 26.14 | -50.80 | 51.65 |  | 0.04 | 23.70 | -46.40 | 46.48 |  | 3.14 | 14.86 | -25.98 | 32.26 |
| 2021 | 20-24 years | 3.38 | 15.01 | -26.04 | 32.80 |  | 0.42 | 26.45 | -51.41 | 52.26 |  | 0.04 | 23.36 | -45.74 | 45.82 |  | 3.19 | 14.84 | -25.89 | 32.27 |
| 2022 | 20-24 years | 3.15 | 17.79 | -31.72 | 38.02 |  | 0.41 | 25.38 | -49.33 | 50.16 |  | 0.04 | 22.67 | -44.39 | 44.47 |  | 2.63 | 17.27 | -31.21 | 36.48 |
| 2023 | 20-24 years | 3.14 | 17.84 | -31.82 | 38.10 |  | 0.41 | 26.65 | -51.82 | 52.64 |  | 0.04 | 23.90 | -46.81 | 46.88 |  | 2.58 | 17.45 | -31.62 | 36.78 |
| 2024 | 20-24 years | 3.13 | 18.08 | -32.32 | 38.57 |  | 0.41 | 29.63 | -57.67 | 58.49 |  | 0.04 | 26.42 | -51.75 | 51.82 |  | 2.54 | 17.72 | -32.20 | 37.28 |
| 2025 | 20-24 years | 3.11 | 18.57 | -33.29 | 39.51 |  | 0.41 | 33.77 | -65.78 | 66.59 |  | 0.04 | 30.00 | -58.76 | 58.83 |  | 2.50 | 18.11 | -32.99 | 37.99 |
| 2026 | 20-24 years | 3.10 | 19.33 | -34.78 | 40.98 |  | 0.41 | 38.57 | -75.18 | 75.99 |  | 0.04 | 34.38 | -67.36 | 67.43 |  | 2.48 | 18.61 | -34.01 | 38.96 |
| 2027 | 20-24 years | 3.09 | 20.33 | -36.76 | 42.94 |  | 0.40 | 43.56 | -84.97 | 85.78 |  | 0.04 | 39.28 | -76.95 | 77.02 |  | 2.45 | 19.27 | -35.32 | 40.23 |
| 2028 | 20-24 years | 3.08 | 21.54 | -39.14 | 45.31 |  | 0.40 | 48.86 | -95.36 | 96.16 |  | 0.04 | 44.46 | -87.11 | 87.18 |  | 2.44 | 20.14 | -37.05 | 41.92 |
| 2029 | 20-24 years | 3.08 | 22.97 | -41.95 | 48.12 |  | 0.40 | 54.41 | -106.24 | 107.04 |  | 0.03 | 49.71 | -97.40 | 97.47 |  | 2.42 | 21.23 | -39.19 | 44.02 |
| 2030 | 20-24 years | 3.09 | 24.61 | -45.16 | 51.33 |  | 0.40 | 59.87 | -116.95 | 117.75 |  | 0.03 | 54.68 | -107.14 | 107.20 |  | 2.40 | 22.50 | -41.70 | 46.49 |
| 2031 | 20-24 years | 3.09 | 26.43 | -48.72 | 54.90 |  | 0.40 | 64.90 | -126.80 | 127.60 |  | 0.03 | 58.95 | -115.51 | 115.57 |  | 2.37 | 23.88 | -44.43 | 49.17 |
| 2032 | 20-24 years | 3.10 | 28.42 | -52.61 | 58.80 |  | 0.40 | 69.33 | -135.48 | 136.28 |  | 0.03 | 62.36 | -122.19 | 122.25 |  | 2.33 | 25.31 | -47.27 | 51.94 |
| 2033 | 20-24 years | 3.10 | 30.63 | -56.93 | 63.13 |  | 0.40 | 73.72 | -144.08 | 144.88 |  | 0.03 | 65.26 | -127.87 | 127.93 |  | 2.28 | 26.87 | -50.39 | 54.95 |
| 2034 | 20-24 years | 3.11 | 33.04 | -61.64 | 67.86 |  | 0.40 | 78.07 | -152.61 | 153.41 |  | 0.03 | 67.93 | -133.11 | 133.17 |  | 2.20 | 28.71 | -54.08 | 58.48 |
| 2035 | 20-24 years | 3.12 | 35.49 | -66.45 | 72.69 |  | 0.40 | 81.91 | -160.15 | 160.95 |  | 0.03 | 69.98 | -137.14 | 137.19 |  | 2.10 | 31.02 | -58.69 | 62.89 |
| 1990 | 25-29 years | 4.30 | 14.38 | -23.88 | 32.48 |  | 0.55 | 20.59 | -39.82 | 40.91 |  | 0.06 | 21.98 | -43.02 | 43.15 |  | 3.78 | 14.58 | -24.79 | 32.35 |
| 1991 | 25-29 years | 4.24 | 14.42 | -24.02 | 32.51 |  | 0.54 | 21.59 | -41.78 | 42.86 |  | 0.06 | 22.44 | -43.92 | 44.04 |  | 3.74 | 14.59 | -24.86 | 32.34 |
| 1992 | 25-29 years | 4.21 | 14.47 | -24.14 | 32.56 |  | 0.53 | 22.22 | -43.02 | 44.08 |  | 0.06 | 22.90 | -44.83 | 44.95 |  | 3.72 | 14.60 | -24.91 | 32.34 |
| 1993 | 25-29 years | 4.19 | 14.50 | -24.23 | 32.60 |  | 0.53 | 22.63 | -43.84 | 44.89 |  | 0.06 | 23.29 | -45.59 | 45.71 |  | 3.68 | 14.62 | -24.96 | 32.33 |
| 1994 | 25-29 years | 4.18 | 14.52 | -24.28 | 32.63 |  | 0.52 | 22.88 | -44.31 | 45.36 |  | 0.06 | 23.63 | -46.25 | 46.37 |  | 3.67 | 14.62 | -25.00 | 32.33 |
| 1995 | 25-29 years | 4.18 | 14.52 | -24.29 | 32.65 |  | 0.52 | 22.77 | -44.11 | 45.16 |  | 0.06 | 23.90 | -46.79 | 46.90 |  | 3.66 | 14.63 | -25.00 | 32.33 |
| 1996 | 25-29 years | 4.18 | 14.52 | -24.28 | 32.64 |  | 0.52 | 22.39 | -43.36 | 44.41 |  | 0.06 | 23.93 | -46.84 | 46.96 |  | 3.64 | 14.64 | -25.05 | 32.33 |
| 1997 | 25-29 years | 4.15 | 14.50 | -24.27 | 32.56 |  | 0.52 | 21.07 | -40.78 | 41.83 |  | 0.06 | 23.93 | -46.85 | 46.96 |  | 3.62 | 14.64 | -25.09 | 32.32 |
| 1998 | 25-29 years | 4.10 | 14.47 | -24.27 | 32.46 |  | 0.52 | 19.77 | -38.23 | 39.28 |  | 0.06 | 23.90 | -46.79 | 46.90 |  | 3.64 | 14.64 | -25.05 | 32.33 |
| 1999 | 25-29 years | 4.06 | 14.47 | -24.30 | 32.41 |  | 0.52 | 18.79 | -36.30 | 37.34 |  | 0.06 | 24.03 | -47.05 | 47.16 |  | 3.70 | 14.61 | -24.95 | 32.34 |
| 2000 | 25-29 years | 4.04 | 14.47 | -24.31 | 32.39 |  | 0.52 | 18.28 | -35.31 | 36.35 |  | 0.06 | 23.86 | -46.72 | 46.83 |  | 3.77 | 14.59 | -24.83 | 32.36 |
| 2001 | 25-29 years | 4.04 | 14.47 | -24.32 | 32.39 |  | 0.52 | 18.08 | -34.92 | 35.96 |  | 0.06 | 23.65 | -46.29 | 46.41 |  | 3.81 | 14.57 | -24.75 | 32.38 |
| 2002 | 25-29 years | 4.04 | 14.46 | -24.31 | 32.38 |  | 0.52 | 17.81 | -34.38 | 35.42 |  | 0.06 | 23.12 | -45.26 | 45.38 |  | 3.81 | 14.57 | -24.75 | 32.37 |
| 2003 | 25-29 years | 4.04 | 14.46 | -24.31 | 32.38 |  | 0.52 | 17.66 | -34.10 | 35.14 |  | 0.06 | 22.90 | -44.83 | 44.95 |  | 3.81 | 14.57 | -24.76 | 32.37 |
| 2004 | 25-29 years | 4.05 | 14.46 | -24.29 | 32.39 |  | 0.52 | 17.64 | -34.05 | 35.10 |  | 0.06 | 22.98 | -44.98 | 45.10 |  | 3.79 | 14.58 | -24.79 | 32.37 |
| 2005 | 25-29 years | 4.07 | 14.46 | -24.27 | 32.40 |  | 0.53 | 17.70 | -34.17 | 35.22 |  | 0.06 | 23.05 | -45.12 | 45.24 |  | 3.80 | 14.58 | -24.78 | 32.37 |
| 2006 | 25-29 years | 4.09 | 14.46 | -24.24 | 32.43 |  | 0.53 | 17.96 | -34.67 | 35.73 |  | 0.06 | 23.24 | -45.50 | 45.62 |  | 3.77 | 14.59 | -24.82 | 32.36 |
| 2007 | 25-29 years | 4.12 | 14.46 | -24.22 | 32.46 |  | 0.53 | 18.37 | -35.47 | 36.53 |  | 0.06 | 23.41 | -45.82 | 45.94 |  | 3.74 | 14.60 | -24.88 | 32.36 |
| 2008 | 25-29 years | 4.14 | 14.48 | -24.23 | 32.51 |  | 0.53 | 18.95 | -36.61 | 37.67 |  | 0.06 | 23.24 | -45.50 | 45.61 |  | 3.69 | 14.62 | -24.97 | 32.34 |
| 2009 | 25-29 years | 4.15 | 14.49 | -24.25 | 32.55 |  | 0.53 | 19.39 | -37.48 | 38.53 |  | 0.06 | 23.34 | -45.70 | 45.81 |  | 3.72 | 14.61 | -24.92 | 32.36 |
| 2010 | 25-29 years | 4.13 | 14.50 | -24.29 | 32.54 |  | 0.53 | 19.60 | -37.90 | 38.95 |  | 0.05 | 22.92 | -44.88 | 44.98 |  | 3.67 | 14.63 | -25.01 | 32.35 |
| 2011 | 25-29 years | 4.09 | 14.51 | -24.35 | 32.52 |  | 0.52 | 19.80 | -38.28 | 39.32 |  | 0.05 | 22.33 | -43.72 | 43.82 |  | 3.57 | 14.67 | -25.18 | 32.33 |
| 2012 | 25-29 years | 4.04 | 14.52 | -24.42 | 32.50 |  | 0.51 | 20.02 | -38.72 | 39.75 |  | 0.05 | 21.63 | -42.34 | 42.45 |  | 3.50 | 14.70 | -25.30 | 32.31 |
| 2013 | 25-29 years | 3.99 | 14.53 | -24.49 | 32.47 |  | 0.51 | 20.23 | -39.14 | 40.16 |  | 0.05 | 21.23 | -41.55 | 41.66 |  | 3.46 | 14.71 | -25.38 | 32.30 |
| 2014 | 25-29 years | 3.96 | 14.54 | -24.54 | 32.45 |  | 0.50 | 20.50 | -39.68 | 40.68 |  | 0.05 | 21.63 | -42.34 | 42.44 |  | 3.45 | 14.72 | -25.40 | 32.30 |
| 2015 | 25-29 years | 3.94 | 14.54 | -24.56 | 32.45 |  | 0.50 | 20.78 | -40.24 | 41.23 |  | 0.05 | 22.49 | -44.04 | 44.14 |  | 3.43 | 14.73 | -25.45 | 32.30 |
| 2016 | 25-29 years | 3.96 | 14.56 | -24.57 | 32.49 |  | 0.50 | 21.42 | -41.49 | 42.48 |  | 0.05 | 23.38 | -45.77 | 45.88 |  | 3.49 | 14.71 | -25.33 | 32.31 |
| 2017 | 25-29 years | 3.99 | 14.58 | -24.58 | 32.56 |  | 0.50 | 22.24 | -43.08 | 44.08 |  | 0.05 | 24.04 | -47.06 | 47.17 |  | 3.52 | 14.69 | -25.28 | 32.32 |
| 2018 | 25-29 years | 4.02 | 14.59 | -24.58 | 32.63 |  | 0.50 | 23.01 | -44.60 | 45.60 |  | 0.05 | 24.02 | -47.03 | 47.13 |  | 3.53 | 14.69 | -25.26 | 32.32 |
| 2019 | 25-29 years | 4.04 | 14.61 | -24.59 | 32.68 |  | 0.50 | 23.57 | -45.71 | 46.71 |  | 0.05 | 23.96 | -46.92 | 47.02 |  | 3.56 | 14.68 | -25.20 | 32.33 |
| 2020 | 25-29 years | 4.06 | 14.62 | -24.60 | 32.72 |  | 0.50 | 24.08 | -46.69 | 47.69 |  | 0.05 | 23.95 | -46.90 | 47.00 |  | 3.58 | 14.67 | -25.17 | 32.33 |
| 2021 | 25-29 years | 4.07 | 14.64 | -24.62 | 32.77 |  | 0.50 | 24.50 | -47.51 | 48.51 |  | 0.05 | 24.06 | -47.10 | 47.20 |  | 3.65 | 14.64 | -25.05 | 32.35 |
| 2022 | 25-29 years | 3.89 | 17.47 | -30.36 | 38.13 |  | 0.49 | 25.25 | -49.00 | 49.99 |  | 0.05 | 24.51 | -48.00 | 48.10 |  | 3.26 | 16.81 | -29.69 | 36.21 |
| 2023 | 25-29 years | 3.87 | 17.52 | -30.46 | 38.20 |  | 0.49 | 26.63 | -51.71 | 52.69 |  | 0.05 | 27.05 | -52.97 | 53.07 |  | 3.21 | 16.91 | -29.93 | 36.35 |
| 2024 | 25-29 years | 3.86 | 17.76 | -30.96 | 38.68 |  | 0.49 | 29.83 | -57.97 | 58.95 |  | 0.05 | 30.77 | -60.26 | 60.36 |  | 3.16 | 17.10 | -30.36 | 36.68 |
| 2025 | 25-29 years | 3.84 | 18.25 | -31.92 | 39.61 |  | 0.49 | 34.20 | -66.55 | 67.52 |  | 0.05 | 35.01 | -68.57 | 68.67 |  | 3.11 | 17.44 | -31.07 | 37.29 |
| 2026 | 25-29 years | 3.83 | 19.01 | -33.43 | 41.08 |  | 0.49 | 39.25 | -76.45 | 77.42 |  | 0.05 | 39.19 | -76.77 | 76.86 |  | 3.05 | 17.97 | -32.18 | 38.27 |
| 2027 | 25-29 years | 3.80 | 20.05 | -35.48 | 43.09 |  | 0.48 | 44.51 | -86.76 | 87.72 |  | 0.04 | 42.85 | -83.94 | 84.03 |  | 2.98 | 18.72 | -33.71 | 39.67 |
| 2028 | 25-29 years | 3.79 | 21.28 | -37.92 | 45.49 |  | 0.48 | 49.98 | -97.47 | 98.43 |  | 0.04 | 46.43 | -90.96 | 91.05 |  | 2.92 | 19.64 | -35.57 | 41.41 |
| 2029 | 25-29 years | 3.77 | 22.71 | -40.74 | 48.28 |  | 0.48 | 55.58 | -108.45 | 109.41 |  | 0.04 | 50.40 | -98.74 | 98.83 |  | 2.87 | 20.73 | -37.76 | 43.50 |
| 2030 | 25-29 years | 3.76 | 24.34 | -43.95 | 51.46 |  | 0.48 | 61.06 | -119.21 | 120.16 |  | 0.04 | 54.87 | -107.49 | 107.58 |  | 2.83 | 22.01 | -40.31 | 45.97 |
| 2031 | 25-29 years | 3.74 | 26.16 | -47.53 | 55.02 |  | 0.47 | 66.19 | -129.26 | 130.21 |  | 0.04 | 59.64 | -116.85 | 116.94 |  | 2.80 | 23.49 | -43.25 | 48.85 |
| 2032 | 25-29 years | 3.73 | 28.14 | -51.42 | 58.88 |  | 0.47 | 70.78 | -138.26 | 139.21 |  | 0.04 | 64.46 | -126.29 | 126.38 |  | 2.78 | 25.20 | -46.61 | 52.17 |
| 2033 | 25-29 years | 3.72 | 30.27 | -55.61 | 63.06 |  | 0.47 | 75.35 | -147.21 | 148.15 |  | 0.04 | 69.25 | -135.68 | 135.77 |  | 2.76 | 27.17 | -50.50 | 56.01 |
| 2034 | 25-29 years | 3.72 | 32.57 | -60.12 | 67.57 |  | 0.47 | 79.95 | -156.24 | 157.18 |  | 0.04 | 73.86 | -144.73 | 144.81 |  | 2.74 | 29.41 | -54.91 | 60.38 |
| 2035 | 25-29 years | 3.72 | 34.98 | -64.84 | 72.29 |  | 0.47 | 84.31 | -164.77 | 165.71 |  | 0.04 | 77.97 | -152.77 | 152.86 |  | 2.71 | 31.84 | -59.71 | 65.13 |
| 1990 | 30-34 years | 5.62 | 13.96 | -21.75 | 32.99 |  | 0.71 | 17.68 | -33.95 | 35.37 |  | 0.09 | 22.49 | -43.99 | 44.17 |  | 5.13 | 14.14 | -22.60 | 32.85 |
| 1991 | 30-34 years | 5.69 | 13.98 | -21.70 | 33.09 |  | 0.71 | 18.85 | -36.24 | 37.66 |  | 0.09 | 22.85 | -44.70 | 44.88 |  | 5.20 | 14.13 | -22.49 | 32.89 |
| 1992 | 30-34 years | 5.71 | 13.99 | -21.72 | 33.14 |  | 0.71 | 19.79 | -38.08 | 39.49 |  | 0.09 | 23.40 | -45.78 | 45.95 |  | 5.21 | 14.12 | -22.47 | 32.90 |
| 1993 | 30-34 years | 5.67 | 14.03 | -21.83 | 33.17 |  | 0.70 | 20.46 | -39.40 | 40.79 |  | 0.09 | 23.79 | -46.54 | 46.71 |  | 5.15 | 14.14 | -22.56 | 32.87 |
| 1994 | 30-34 years | 5.59 | 14.07 | -22.00 | 33.17 |  | 0.69 | 21.07 | -40.62 | 41.99 |  | 0.08 | 24.12 | -47.19 | 47.36 |  | 5.07 | 14.17 | -22.69 | 32.84 |
| 1995 | 30-34 years | 5.49 | 14.12 | -22.18 | 33.16 |  | 0.67 | 21.51 | -41.48 | 42.83 |  | 0.08 | 24.45 | -47.83 | 48.00 |  | 4.94 | 14.20 | -22.90 | 32.78 |
| 1996 | 30-34 years | 5.35 | 14.14 | -22.37 | 33.07 |  | 0.66 | 21.09 | -40.68 | 41.99 |  | 0.08 | 24.50 | -47.95 | 48.10 |  | 4.76 | 14.26 | -23.19 | 32.70 |
| 1997 | 30-34 years | 5.22 | 14.16 | -22.52 | 32.97 |  | 0.65 | 20.27 | -39.09 | 40.38 |  | 0.07 | 24.52 | -47.98 | 48.13 |  | 4.61 | 14.30 | -23.42 | 32.64 |
| 1998 | 30-34 years | 5.12 | 14.16 | -22.64 | 32.88 |  | 0.64 | 19.35 | -37.28 | 38.56 |  | 0.07 | 24.62 | -48.17 | 48.32 |  | 4.52 | 14.33 | -23.56 | 32.61 |
| 1999 | 30-34 years | 5.05 | 14.17 | -22.71 | 32.82 |  | 0.63 | 18.62 | -35.86 | 37.12 |  | 0.07 | 25.12 | -49.16 | 49.31 |  | 4.55 | 14.32 | -23.52 | 32.63 |
| 2000 | 30-34 years | 5.03 | 14.17 | -22.73 | 32.80 |  | 0.63 | 18.27 | -35.19 | 36.45 |  | 0.07 | 24.87 | -48.67 | 48.82 |  | 4.54 | 14.33 | -23.53 | 32.62 |
| 2001 | 30-34 years | 5.07 | 14.17 | -22.70 | 32.84 |  | 0.63 | 18.60 | -35.83 | 37.09 |  | 0.07 | 24.66 | -48.26 | 48.41 |  | 4.55 | 14.33 | -23.53 | 32.63 |
| 2002 | 30-34 years | 5.08 | 14.17 | -22.69 | 32.84 |  | 0.63 | 18.54 | -35.71 | 36.98 |  | 0.07 | 23.94 | -46.84 | 46.98 |  | 4.48 | 14.35 | -23.64 | 32.60 |
| 2003 | 30-34 years | 5.08 | 14.16 | -22.68 | 32.84 |  | 0.63 | 18.52 | -35.67 | 36.94 |  | 0.07 | 23.55 | -46.09 | 46.23 |  | 4.45 | 14.36 | -23.69 | 32.59 |
| 2004 | 30-34 years | 5.08 | 14.17 | -22.68 | 32.84 |  | 0.63 | 18.60 | -35.82 | 37.09 |  | 0.07 | 23.41 | -45.82 | 45.96 |  | 4.42 | 14.37 | -23.73 | 32.58 |
| 2005 | 30-34 years | 5.08 | 14.17 | -22.68 | 32.85 |  | 0.63 | 18.70 | -36.02 | 37.28 |  | 0.07 | 22.87 | -44.75 | 44.89 |  | 4.35 | 14.39 | -23.85 | 32.55 |
| 2006 | 30-34 years | 5.10 | 14.17 | -22.67 | 32.87 |  | 0.63 | 18.85 | -36.30 | 37.57 |  | 0.07 | 22.75 | -44.52 | 44.66 |  | 4.33 | 14.39 | -23.88 | 32.54 |
| 2007 | 30-34 years | 5.13 | 14.17 | -22.65 | 32.90 |  | 0.64 | 19.13 | -36.85 | 38.12 |  | 0.07 | 22.55 | -44.13 | 44.27 |  | 4.33 | 14.39 | -23.88 | 32.54 |
| 2008 | 30-34 years | 5.16 | 14.18 | -22.63 | 32.96 |  | 0.64 | 19.68 | -37.94 | 39.22 |  | 0.07 | 22.47 | -43.98 | 44.12 |  | 4.33 | 14.39 | -23.88 | 32.54 |
| 2009 | 30-34 years | 5.20 | 14.18 | -22.60 | 33.01 |  | 0.64 | 20.30 | -39.14 | 40.42 |  | 0.07 | 22.90 | -44.81 | 44.95 |  | 4.35 | 14.39 | -23.84 | 32.55 |
| 2010 | 30-34 years | 5.24 | 14.19 | -22.57 | 33.04 |  | 0.64 | 20.85 | -40.22 | 41.51 |  | 0.07 | 23.13 | -45.27 | 45.41 |  | 4.30 | 14.40 | -23.92 | 32.53 |
| 2011 | 30-34 years | 5.24 | 14.19 | -22.56 | 33.05 |  | 0.64 | 21.21 | -40.93 | 42.21 |  | 0.07 | 23.44 | -45.87 | 46.01 |  | 4.25 | 14.42 | -24.01 | 32.51 |
| 2012 | 30-34 years | 5.22 | 14.19 | -22.59 | 33.03 |  | 0.64 | 21.32 | -41.15 | 42.42 |  | 0.07 | 23.66 | -46.30 | 46.43 |  | 4.17 | 14.45 | -24.15 | 32.49 |
| 2013 | 30-34 years | 5.17 | 14.19 | -22.65 | 32.99 |  | 0.63 | 21.37 | -41.26 | 42.52 |  | 0.07 | 23.69 | -46.37 | 46.51 |  | 4.14 | 14.46 | -24.20 | 32.48 |
| 2014 | 30-34 years | 5.11 | 14.20 | -22.72 | 32.94 |  | 0.62 | 21.23 | -40.99 | 42.23 |  | 0.07 | 23.54 | -46.08 | 46.21 |  | 4.10 | 14.47 | -24.27 | 32.46 |
| 2015 | 30-34 years | 5.04 | 14.21 | -22.80 | 32.89 |  | 0.62 | 20.90 | -40.34 | 41.57 |  | 0.07 | 22.95 | -44.91 | 45.04 |  | 4.01 | 14.50 | -24.42 | 32.44 |
| 2016 | 30-34 years | 4.99 | 14.22 | -22.88 | 32.85 |  | 0.61 | 20.73 | -40.03 | 41.25 |  | 0.06 | 22.43 | -43.89 | 44.02 |  | 3.96 | 14.52 | -24.50 | 32.43 |
| 2017 | 30-34 years | 4.94 | 14.23 | -22.95 | 32.84 |  | 0.60 | 20.69 | -39.94 | 41.15 |  | 0.06 | 21.75 | -42.57 | 42.70 |  | 3.86 | 14.56 | -24.68 | 32.40 |
| 2018 | 30-34 years | 4.91 | 14.25 | -23.02 | 32.83 |  | 0.60 | 20.68 | -39.92 | 41.12 |  | 0.06 | 21.25 | -41.59 | 41.71 |  | 3.79 | 14.59 | -24.80 | 32.38 |
| 2019 | 30-34 years | 4.88 | 14.26 | -23.06 | 32.82 |  | 0.60 | 20.58 | -39.74 | 40.93 |  | 0.06 | 21.66 | -42.39 | 42.52 |  | 3.77 | 14.59 | -24.84 | 32.37 |
| 2020 | 30-34 years | 4.86 | 14.26 | -23.08 | 32.81 |  | 0.60 | 20.54 | -39.65 | 40.85 |  | 0.06 | 22.33 | -43.70 | 43.82 |  | 3.72 | 14.61 | -24.92 | 32.36 |
| 2021 | 30-34 years | 4.88 | 14.26 | -23.08 | 32.83 |  | 0.60 | 20.77 | -40.12 | 41.31 |  | 0.06 | 22.96 | -44.95 | 45.07 |  | 3.78 | 14.59 | -24.82 | 32.37 |
| 2022 | 30-34 years | 4.85 | 17.18 | -28.82 | 38.51 |  | 0.59 | 25.49 | -49.37 | 50.56 |  | 0.06 | 25.29 | -49.50 | 49.62 |  | 3.86 | 16.47 | -28.43 | 36.14 |
| 2023 | 30-34 years | 4.85 | 17.18 | -28.83 | 38.52 |  | 0.59 | 26.77 | -51.87 | 53.06 |  | 0.06 | 28.68 | -56.14 | 56.27 |  | 3.85 | 16.55 | -28.59 | 36.29 |
| 2024 | 30-34 years | 4.84 | 17.43 | -29.32 | 39.01 |  | 0.59 | 29.94 | -58.09 | 59.28 |  | 0.06 | 33.35 | -65.30 | 65.42 |  | 3.83 | 16.75 | -28.99 | 36.66 |
| 2025 | 30-34 years | 4.84 | 17.93 | -30.31 | 39.98 |  | 0.59 | 34.38 | -66.78 | 67.97 |  | 0.06 | 38.74 | -75.88 | 76.00 |  | 3.81 | 17.07 | -29.65 | 37.27 |
| 2026 | 30-34 years | 4.82 | 18.70 | -31.83 | 41.47 |  | 0.59 | 39.54 | -76.91 | 78.09 |  | 0.06 | 44.28 | -86.72 | 86.84 |  | 3.77 | 17.56 | -30.64 | 38.19 |
| 2027 | 30-34 years | 4.80 | 19.73 | -33.87 | 43.48 |  | 0.59 | 44.94 | -87.49 | 88.67 |  | 0.06 | 49.46 | -96.87 | 96.99 |  | 3.72 | 18.23 | -32.02 | 39.46 |
| 2028 | 30-34 years | 4.78 | 20.98 | -36.33 | 45.90 |  | 0.59 | 50.58 | -98.54 | 99.72 |  | 0.06 | 54.45 | -106.66 | 106.77 |  | 3.67 | 19.07 | -33.70 | 41.04 |
| 2029 | 30-34 years | 4.77 | 22.43 | -39.19 | 48.73 |  | 0.59 | 56.33 | -109.83 | 111.00 |  | 0.06 | 59.30 | -116.17 | 116.29 |  | 3.61 | 20.06 | -35.70 | 42.92 |
| 2030 | 30-34 years | 4.75 | 24.07 | -42.43 | 51.93 |  | 0.59 | 61.93 | -120.80 | 121.97 |  | 0.06 | 63.75 | -124.89 | 125.00 |  | 3.55 | 21.25 | -38.10 | 45.19 |
| 2031 | 30-34 years | 4.73 | 25.90 | -46.04 | 55.49 |  | 0.58 | 67.15 | -131.03 | 132.20 |  | 0.06 | 67.41 | -132.07 | 132.18 |  | 3.48 | 22.69 | -40.99 | 47.95 |
| 2032 | 30-34 years | 4.70 | 27.90 | -49.99 | 59.39 |  | 0.58 | 71.83 | -140.20 | 141.36 |  | 0.05 | 70.08 | -137.30 | 137.41 |  | 3.41 | 24.41 | -44.43 | 51.24 |
| 2033 | 30-34 years | 4.68 | 30.04 | -54.19 | 63.55 |  | 0.58 | 76.39 | -149.14 | 150.30 |  | 0.05 | 72.57 | -142.18 | 142.28 |  | 3.34 | 26.34 | -48.29 | 54.96 |
| 2034 | 30-34 years | 4.66 | 32.30 | -58.65 | 67.97 |  | 0.57 | 80.90 | -157.99 | 159.14 |  | 0.05 | 75.46 | -147.85 | 147.95 |  | 3.28 | 28.50 | -52.57 | 59.13 |
| 2035 | 30-34 years | 4.64 | 34.66 | -63.29 | 72.58 |  | 0.57 | 85.17 | -166.35 | 167.49 |  | 0.05 | 78.86 | -154.51 | 154.61 |  | 3.24 | 30.89 | -57.32 | 63.79 |
| 1990 | 35-39 years | 7.39 | 13.60 | -19.26 | 34.04 |  | 0.88 | 18.47 | -35.32 | 37.07 |  | 0.13 | 24.11 | -47.12 | 47.38 |  | 7.09 | 13.69 | -19.74 | 33.92 |
| 1991 | 35-39 years | 7.41 | 13.61 | -19.26 | 34.09 |  | 0.87 | 19.33 | -37.01 | 38.76 |  | 0.13 | 25.07 | -49.01 | 49.27 |  | 7.19 | 13.67 | -19.60 | 33.98 |
| 1992 | 35-39 years | 7.44 | 13.61 | -19.24 | 34.13 |  | 0.88 | 19.37 | -37.09 | 38.84 |  | 0.13 | 25.05 | -48.98 | 49.24 |  | 7.22 | 13.66 | -19.56 | 34.00 |
| 1993 | 35-39 years | 7.51 | 13.61 | -19.17 | 34.19 |  | 0.88 | 19.58 | -37.50 | 39.26 |  | 0.13 | 25.42 | -49.69 | 49.96 |  | 7.38 | 13.63 | -19.35 | 34.10 |
| 1994 | 35-39 years | 7.58 | 13.61 | -19.10 | 34.27 |  | 0.89 | 19.74 | -37.81 | 39.59 |  | 0.13 | 25.34 | -49.52 | 49.79 |  | 7.46 | 13.62 | -19.23 | 34.15 |
| 1995 | 35-39 years | 7.63 | 13.62 | -19.06 | 34.32 |  | 0.89 | 19.73 | -37.79 | 39.57 |  | 0.13 | 25.36 | -49.57 | 49.84 |  | 7.50 | 13.61 | -19.18 | 34.18 |
| 1996 | 35-39 years | 7.61 | 13.62 | -19.09 | 34.30 |  | 0.89 | 19.38 | -37.09 | 38.87 |  | 0.13 | 24.82 | -48.51 | 48.78 |  | 7.34 | 13.64 | -19.40 | 34.08 |
| 1997 | 35-39 years | 7.49 | 13.63 | -19.22 | 34.20 |  | 0.88 | 18.55 | -35.47 | 37.24 |  | 0.13 | 24.48 | -47.85 | 48.10 |  | 7.11 | 13.69 | -19.72 | 33.94 |
| 1998 | 35-39 years | 7.30 | 13.65 | -19.45 | 34.04 |  | 0.86 | 17.59 | -33.61 | 35.34 |  | 0.12 | 24.25 | -47.40 | 47.65 |  | 6.90 | 13.73 | -20.01 | 33.81 |
| 1999 | 35-39 years | 7.09 | 13.68 | -19.72 | 33.90 |  | 0.84 | 16.93 | -32.34 | 34.03 |  | 0.12 | 24.39 | -47.69 | 47.93 |  | 6.75 | 13.76 | -20.22 | 33.73 |
| 2000 | 35-39 years | 6.94 | 13.71 | -19.94 | 33.81 |  | 0.83 | 16.96 | -32.42 | 34.07 |  | 0.12 | 24.07 | -47.05 | 47.29 |  | 6.59 | 13.80 | -20.45 | 33.63 |
| 2001 | 35-39 years | 6.80 | 13.74 | -20.14 | 33.74 |  | 0.81 | 17.14 | -32.79 | 34.41 |  | 0.11 | 24.02 | -46.97 | 47.20 |  | 6.42 | 13.84 | -20.70 | 33.54 |
| 2002 | 35-39 years | 6.72 | 13.77 | -20.27 | 33.70 |  | 0.80 | 17.61 | -33.71 | 35.31 |  | 0.11 | 23.57 | -46.09 | 46.32 |  | 6.25 | 13.87 | -20.94 | 33.44 |
| 2003 | 35-39 years | 6.67 | 13.78 | -20.35 | 33.68 |  | 0.79 | 18.02 | -34.52 | 36.10 |  | 0.11 | 23.14 | -45.24 | 45.45 |  | 6.05 | 13.92 | -21.23 | 33.33 |
| 2004 | 35-39 years | 6.65 | 13.79 | -20.38 | 33.68 |  | 0.79 | 18.39 | -35.26 | 36.83 |  | 0.11 | 22.83 | -44.64 | 44.85 |  | 5.87 | 13.96 | -21.50 | 33.24 |
| 2005 | 35-39 years | 6.66 | 13.79 | -20.37 | 33.70 |  | 0.79 | 18.62 | -35.71 | 37.28 |  | 0.10 | 22.43 | -43.86 | 44.07 |  | 5.74 | 13.99 | -21.69 | 33.17 |
| 2006 | 35-39 years | 6.72 | 13.79 | -20.31 | 33.76 |  | 0.79 | 19.26 | -36.96 | 38.53 |  | 0.10 | 22.42 | -43.84 | 44.05 |  | 5.67 | 14.01 | -21.79 | 33.14 |
| 2007 | 35-39 years | 6.77 | 13.79 | -20.26 | 33.80 |  | 0.79 | 19.61 | -37.65 | 39.23 |  | 0.10 | 22.08 | -43.18 | 43.38 |  | 5.58 | 14.03 | -21.92 | 33.09 |
| 2008 | 35-39 years | 6.81 | 13.79 | -20.23 | 33.84 |  | 0.79 | 20.15 | -38.69 | 40.28 |  | 0.10 | 22.30 | -43.62 | 43.82 |  | 5.63 | 14.02 | -21.85 | 33.12 |
| 2009 | 35-39 years | 6.83 | 13.80 | -20.21 | 33.87 |  | 0.79 | 20.72 | -39.82 | 41.40 |  | 0.10 | 22.28 | -43.57 | 43.77 |  | 5.58 | 14.04 | -21.93 | 33.09 |
| 2010 | 35-39 years | 6.84 | 13.80 | -20.20 | 33.89 |  | 0.79 | 21.16 | -40.68 | 42.26 |  | 0.10 | 22.12 | -43.25 | 43.45 |  | 5.49 | 14.06 | -22.06 | 33.05 |
| 2011 | 35-39 years | 6.82 | 13.80 | -20.23 | 33.87 |  | 0.79 | 21.29 | -40.94 | 42.51 |  | 0.10 | 21.82 | -42.66 | 42.86 |  | 5.39 | 14.08 | -22.22 | 32.99 |
| 2012 | 35-39 years | 6.77 | 13.80 | -20.28 | 33.83 |  | 0.78 | 21.08 | -40.53 | 42.10 |  | 0.10 | 21.59 | -42.22 | 42.42 |  | 5.31 | 14.10 | -22.33 | 32.96 |
| 2013 | 35-39 years | 6.72 | 13.81 | -20.34 | 33.78 |  | 0.78 | 20.81 | -40.01 | 41.56 |  | 0.10 | 22.26 | -43.53 | 43.73 |  | 5.45 | 14.07 | -22.12 | 33.02 |
| 2014 | 35-39 years | 6.66 | 13.81 | -20.40 | 33.72 |  | 0.77 | 20.51 | -39.43 | 40.97 |  | 0.10 | 22.29 | -43.59 | 43.78 |  | 5.38 | 14.09 | -22.23 | 32.99 |
| 2015 | 35-39 years | 6.63 | 13.81 | -20.43 | 33.69 |  | 0.77 | 20.28 | -38.98 | 40.51 |  | 0.10 | 22.84 | -44.67 | 44.87 |  | 5.41 | 14.08 | -22.19 | 33.00 |
| 2016 | 35-39 years | 6.62 | 13.81 | -20.45 | 33.68 |  | 0.76 | 20.23 | -38.89 | 40.42 |  | 0.10 | 23.25 | -45.47 | 45.66 |  | 5.41 | 14.08 | -22.18 | 33.01 |
| 2017 | 35-39 years | 6.61 | 13.81 | -20.47 | 33.68 |  | 0.76 | 20.19 | -38.81 | 40.34 |  | 0.10 | 23.74 | -46.44 | 46.63 |  | 5.35 | 14.09 | -22.27 | 32.98 |
| 2018 | 35-39 years | 6.59 | 13.82 | -20.50 | 33.67 |  | 0.76 | 20.15 | -38.73 | 40.25 |  | 0.10 | 23.34 | -45.65 | 45.84 |  | 5.22 | 14.13 | -22.48 | 32.91 |
| 2019 | 35-39 years | 6.54 | 13.83 | -20.57 | 33.64 |  | 0.76 | 19.87 | -38.18 | 39.70 |  | 0.09 | 23.32 | -45.62 | 45.81 |  | 5.21 | 14.13 | -22.49 | 32.91 |
| 2020 | 35-39 years | 6.47 | 13.84 | -20.66 | 33.59 |  | 0.75 | 19.36 | -37.19 | 38.70 |  | 0.09 | 22.64 | -44.29 | 44.47 |  | 5.07 | 14.17 | -22.71 | 32.85 |
| 2021 | 35-39 years | 6.39 | 13.85 | -20.76 | 33.54 |  | 0.74 | 19.04 | -36.57 | 38.06 |  | 0.09 | 22.14 | -43.30 | 43.48 |  | 5.04 | 14.18 | -22.76 | 32.83 |
| 2022 | 35-39 years | 6.38 | 16.78 | -26.50 | 39.26 |  | 0.74 | 25.55 | -49.34 | 50.81 |  | 0.09 | 23.83 | -46.62 | 46.80 |  | 5.04 | 16.44 | -27.19 | 37.27 |
| 2023 | 35-39 years | 6.34 | 16.89 | -26.77 | 39.44 |  | 0.73 | 26.91 | -52.00 | 53.47 |  | 0.09 | 27.72 | -54.24 | 54.42 |  | 4.99 | 16.53 | -27.41 | 37.38 |
| 2024 | 35-39 years | 6.31 | 17.19 | -27.38 | 39.99 |  | 0.73 | 30.20 | -58.46 | 59.92 |  | 0.09 | 33.93 | -66.42 | 66.60 |  | 4.97 | 16.64 | -27.64 | 37.59 |
| 2025 | 35-39 years | 6.30 | 17.64 | -28.28 | 40.88 |  | 0.73 | 34.79 | -67.46 | 68.92 |  | 0.09 | 41.33 | -80.91 | 81.09 |  | 4.98 | 16.81 | -27.97 | 37.93 |
| 2026 | 35-39 years | 6.29 | 18.32 | -29.61 | 42.20 |  | 0.73 | 39.99 | -77.65 | 79.11 |  | 0.09 | 48.38 | -94.74 | 94.92 |  | 5.00 | 17.15 | -28.62 | 38.61 |
| 2027 | 35-39 years | 6.29 | 19.24 | -31.42 | 44.01 |  | 0.73 | 45.31 | -88.07 | 89.53 |  | 0.09 | 54.59 | -106.91 | 107.09 |  | 5.00 | 17.70 | -29.70 | 39.70 |
| 2028 | 35-39 years | 6.29 | 20.42 | -33.74 | 46.32 |  | 0.73 | 50.87 | -98.97 | 100.43 |  | 0.09 | 60.40 | -118.31 | 118.48 |  | 4.99 | 18.46 | -31.19 | 41.17 |
| 2029 | 35-39 years | 6.29 | 21.85 | -36.53 | 49.10 |  | 0.73 | 56.55 | -110.11 | 111.57 |  | 0.09 | 65.99 | -129.25 | 129.42 |  | 4.97 | 19.40 | -33.05 | 43.00 |
| 2030 | 35-39 years | 6.28 | 23.48 | -39.74 | 52.29 |  | 0.73 | 62.11 | -121.00 | 122.46 |  | 0.09 | 71.15 | -139.36 | 139.54 |  | 4.94 | 20.55 | -35.33 | 45.21 |
| 2031 | 35-39 years | 6.26 | 25.31 | -43.34 | 55.86 |  | 0.73 | 67.34 | -131.26 | 132.72 |  | 0.09 | 75.54 | -147.97 | 148.15 |  | 4.89 | 21.93 | -38.09 | 47.87 |
| 2032 | 35-39 years | 6.23 | 27.31 | -47.30 | 59.77 |  | 0.72 | 72.07 | -140.52 | 141.97 |  | 0.08 | 79.07 | -154.88 | 155.05 |  | 4.83 | 23.56 | -41.35 | 51.01 |
| 2033 | 35-39 years | 6.21 | 29.47 | -51.55 | 63.98 |  | 0.72 | 76.71 | -149.63 | 151.08 |  | 0.08 | 82.32 | -161.27 | 161.43 |  | 4.76 | 25.39 | -45.01 | 54.53 |
| 2034 | 35-39 years | 6.19 | 31.76 | -56.06 | 68.44 |  | 0.72 | 81.30 | -158.62 | 160.06 |  | 0.08 | 85.48 | -167.46 | 167.62 |  | 4.68 | 27.41 | -49.04 | 58.41 |
| 2035 | 35-39 years | 6.16 | 34.13 | -60.72 | 73.05 |  | 0.72 | 85.59 | -167.03 | 168.47 |  | 0.08 | 88.27 | -172.94 | 173.10 |  | 4.60 | 29.63 | -53.48 | 62.68 |
| 1990 | 40-44 years | 10.73 | 13.09 | -14.93 | 36.39 |  | 1.26 | 18.75 | -35.49 | 38.01 |  | 0.22 | 24.36 | -47.53 | 47.96 |  | 10.75 | 13.09 | -14.92 | 36.41 |
| 1991 | 40-44 years | 10.86 | 13.09 | -14.80 | 36.52 |  | 1.27 | 19.65 | -37.24 | 39.77 |  | 0.22 | 25.10 | -48.98 | 49.42 |  | 11.01 | 13.06 | -14.59 | 36.61 |
| 1992 | 40-44 years | 10.76 | 13.11 | -14.93 | 36.45 |  | 1.26 | 19.52 | -37.00 | 39.52 |  | 0.22 | 26.50 | -51.72 | 52.16 |  | 11.26 | 13.03 | -14.28 | 36.80 |
| 1993 | 40-44 years | 10.59 | 13.13 | -15.14 | 36.33 |  | 1.24 | 19.40 | -36.79 | 39.27 |  | 0.22 | 28.05 | -54.76 | 55.19 |  | 11.49 | 13.00 | -14.00 | 36.98 |
| 1994 | 40-44 years | 10.46 | 13.16 | -15.32 | 36.25 |  | 1.23 | 19.71 | -37.41 | 39.86 |  | 0.22 | 28.66 | -55.96 | 56.40 |  | 11.48 | 13.01 | -14.02 | 36.97 |
| 1995 | 40-44 years | 10.31 | 13.18 | -15.52 | 36.14 |  | 1.21 | 19.39 | -36.80 | 39.22 |  | 0.21 | 28.54 | -55.73 | 56.16 |  | 11.22 | 13.04 | -14.34 | 36.78 |
| 1996 | 40-44 years | 10.22 | 13.19 | -15.64 | 36.08 |  | 1.20 | 19.20 | -36.42 | 38.83 |  | 0.21 | 27.89 | -54.45 | 54.88 |  | 10.94 | 13.08 | -14.69 | 36.56 |
| 1997 | 40-44 years | 10.11 | 13.20 | -15.75 | 35.97 |  | 1.20 | 18.13 | -34.34 | 36.74 |  | 0.21 | 26.24 | -51.23 | 51.64 |  | 10.59 | 13.12 | -15.12 | 36.31 |
| 1998 | 40-44 years | 10.06 | 13.19 | -15.80 | 35.92 |  | 1.20 | 17.30 | -32.70 | 35.10 |  | 0.21 | 25.23 | -49.25 | 49.67 |  | 10.50 | 13.13 | -15.24 | 36.24 |
| 1999 | 40-44 years | 10.04 | 13.19 | -15.81 | 35.90 |  | 1.20 | 16.67 | -31.48 | 33.88 |  | 0.21 | 25.01 | -48.80 | 49.22 |  | 10.62 | 13.12 | -15.10 | 36.33 |
| 2000 | 40-44 years | 10.05 | 13.19 | -15.80 | 35.90 |  | 1.20 | 16.35 | -30.85 | 33.25 |  | 0.21 | 24.10 | -47.02 | 47.44 |  | 10.68 | 13.11 | -15.02 | 36.37 |
| 2001 | 40-44 years | 10.06 | 13.19 | -15.80 | 35.91 |  | 1.20 | 16.45 | -31.04 | 33.45 |  | 0.21 | 23.05 | -44.97 | 45.39 |  | 10.51 | 13.13 | -15.23 | 36.25 |
| 2002 | 40-44 years | 10.00 | 13.20 | -15.88 | 35.87 |  | 1.19 | 16.70 | -31.53 | 33.92 |  | 0.21 | 22.69 | -44.27 | 44.69 |  | 10.38 | 13.15 | -15.39 | 36.16 |
| 2003 | 40-44 years | 9.86 | 13.22 | -16.06 | 35.78 |  | 1.18 | 16.90 | -31.96 | 34.31 |  | 0.20 | 22.97 | -44.82 | 45.22 |  | 10.24 | 13.17 | -15.57 | 36.06 |
| 2004 | 40-44 years | 9.69 | 13.25 | -16.28 | 35.67 |  | 1.15 | 17.27 | -32.69 | 34.99 |  | 0.20 | 22.74 | -44.37 | 44.76 |  | 9.85 | 13.23 | -16.07 | 35.78 |
| 2005 | 40-44 years | 9.53 | 13.28 | -16.51 | 35.57 |  | 1.13 | 17.77 | -33.71 | 35.97 |  | 0.19 | 21.66 | -42.26 | 42.63 |  | 9.33 | 13.30 | -16.74 | 35.41 |
| 2006 | 40-44 years | 9.36 | 13.31 | -16.73 | 35.46 |  | 1.11 | 18.11 | -34.38 | 36.59 |  | 0.18 | 20.89 | -40.77 | 41.13 |  | 8.80 | 13.39 | -17.44 | 35.04 |
| 2007 | 40-44 years | 9.27 | 13.34 | -16.87 | 35.41 |  | 1.09 | 18.73 | -35.62 | 37.81 |  | 0.17 | 20.49 | -40.00 | 40.34 |  | 8.41 | 13.45 | -17.96 | 34.77 |
| 2008 | 40-44 years | 9.23 | 13.35 | -16.94 | 35.40 |  | 1.08 | 19.38 | -36.91 | 39.07 |  | 0.17 | 20.66 | -40.34 | 40.67 |  | 8.20 | 13.49 | -18.24 | 34.64 |
| 2009 | 40-44 years | 9.22 | 13.36 | -16.96 | 35.40 |  | 1.08 | 19.98 | -38.08 | 40.23 |  | 0.16 | 20.54 | -40.10 | 40.42 |  | 7.92 | 13.54 | -18.62 | 34.45 |
| 2010 | 40-44 years | 9.23 | 13.36 | -16.96 | 35.41 |  | 1.07 | 20.35 | -38.81 | 40.95 |  | 0.16 | 20.77 | -40.54 | 40.86 |  | 7.77 | 13.57 | -18.82 | 34.36 |
| 2011 | 40-44 years | 9.25 | 13.36 | -16.93 | 35.43 |  | 1.07 | 20.76 | -39.63 | 41.77 |  | 0.16 | 20.76 | -40.53 | 40.84 |  | 7.60 | 13.60 | -19.05 | 34.25 |
| 2012 | 40-44 years | 9.18 | 13.36 | -17.01 | 35.36 |  | 1.06 | 20.36 | -38.85 | 40.97 |  | 0.15 | 20.92 | -40.86 | 41.16 |  | 7.53 | 13.61 | -19.15 | 34.20 |
| 2013 | 40-44 years | 9.06 | 13.37 | -17.14 | 35.27 |  | 1.05 | 19.82 | -37.80 | 39.90 |  | 0.15 | 21.19 | -41.38 | 41.68 |  | 7.52 | 13.61 | -19.15 | 34.20 |
| 2014 | 40-44 years | 8.94 | 13.38 | -17.28 | 35.16 |  | 1.04 | 19.26 | -36.71 | 38.79 |  | 0.15 | 20.67 | -40.37 | 40.67 |  | 7.37 | 13.64 | -19.36 | 34.11 |
| 2015 | 40-44 years | 8.85 | 13.38 | -17.39 | 35.08 |  | 1.03 | 18.78 | -35.78 | 37.83 |  | 0.15 | 20.97 | -40.94 | 41.25 |  | 7.40 | 13.64 | -19.33 | 34.12 |
| 2016 | 40-44 years | 8.78 | 13.39 | -17.47 | 35.03 |  | 1.02 | 18.43 | -35.10 | 37.15 |  | 0.15 | 21.42 | -41.82 | 42.13 |  | 7.48 | 13.62 | -19.22 | 34.17 |
| 2017 | 40-44 years | 8.74 | 13.40 | -17.52 | 35.00 |  | 1.02 | 18.11 | -34.49 | 36.52 |  | 0.15 | 21.52 | -42.03 | 42.34 |  | 7.44 | 13.63 | -19.27 | 34.15 |
| 2018 | 40-44 years | 8.71 | 13.40 | -17.55 | 34.97 |  | 1.02 | 17.89 | -34.04 | 36.07 |  | 0.15 | 21.64 | -42.27 | 42.57 |  | 7.45 | 13.63 | -19.26 | 34.15 |
| 2019 | 40-44 years | 8.69 | 13.40 | -17.58 | 34.96 |  | 1.01 | 17.63 | -33.54 | 35.57 |  | 0.15 | 22.51 | -43.96 | 44.27 |  | 7.58 | 13.60 | -19.08 | 34.24 |
| 2020 | 40-44 years | 8.69 | 13.40 | -17.58 | 34.96 |  | 1.02 | 17.37 | -33.03 | 35.06 |  | 0.15 | 22.70 | -44.33 | 44.63 |  | 7.51 | 13.61 | -19.18 | 34.19 |
| 2021 | 40-44 years | 8.68 | 13.40 | -17.60 | 34.95 |  | 1.01 | 17.28 | -32.86 | 34.88 |  | 0.15 | 22.99 | -44.92 | 45.22 |  | 7.55 | 13.61 | -19.12 | 34.22 |
| 2022 | 40-44 years | 8.80 | 16.07 | -22.71 | 40.31 |  | 1.02 | 25.06 | -48.10 | 50.14 |  | 0.15 | 26.11 | -51.03 | 51.33 |  | 7.79 | 15.25 | -22.11 | 37.69 |
| 2023 | 40-44 years | 8.77 | 16.12 | -22.82 | 40.37 |  | 1.02 | 26.35 | -50.63 | 52.67 |  | 0.15 | 30.68 | -59.99 | 60.28 |  | 7.69 | 15.37 | -22.44 | 37.82 |
| 2024 | 40-44 years | 8.73 | 16.41 | -23.44 | 40.90 |  | 1.01 | 29.66 | -57.13 | 59.15 |  | 0.14 | 37.11 | -72.60 | 72.89 |  | 7.56 | 15.66 | -23.14 | 38.26 |
| 2025 | 40-44 years | 8.68 | 16.93 | -24.51 | 41.87 |  | 1.01 | 34.29 | -66.20 | 68.22 |  | 0.14 | 44.43 | -86.94 | 87.22 |  | 7.40 | 16.14 | -24.23 | 39.04 |
| 2026 | 40-44 years | 8.62 | 17.71 | -26.09 | 43.32 |  | 1.00 | 39.54 | -76.50 | 78.50 |  | 0.14 | 51.33 | -100.47 | 100.75 |  | 7.26 | 16.73 | -25.54 | 40.05 |
| 2027 | 40-44 years | 8.56 | 18.71 | -28.11 | 45.22 |  | 0.99 | 44.86 | -86.94 | 88.93 |  | 0.14 | 57.14 | -111.86 | 112.14 |  | 7.14 | 17.40 | -26.96 | 41.24 |
| 2028 | 40-44 years | 8.50 | 19.94 | -30.58 | 47.58 |  | 0.99 | 50.44 | -97.87 | 99.84 |  | 0.14 | 63.01 | -123.35 | 123.63 |  | 7.07 | 18.16 | -28.52 | 42.65 |
| 2029 | 40-44 years | 8.46 | 21.37 | -33.43 | 50.35 |  | 0.98 | 56.23 | -109.24 | 111.20 |  | 0.14 | 69.54 | -136.16 | 136.43 |  | 7.05 | 19.03 | -30.26 | 44.35 |
| 2030 | 40-44 years | 8.45 | 22.97 | -36.57 | 53.46 |  | 0.98 | 62.00 | -120.54 | 122.50 |  | 0.14 | 76.00 | -148.82 | 149.09 |  | 7.06 | 20.05 | -32.23 | 46.35 |
| 2031 | 40-44 years | 8.44 | 24.70 | -39.97 | 56.85 |  | 0.98 | 67.24 | -130.80 | 132.76 |  | 0.14 | 81.19 | -159.00 | 159.27 |  | 7.08 | 21.26 | -34.59 | 48.75 |
| 2032 | 40-44 years | 8.44 | 26.57 | -43.63 | 60.52 |  | 0.98 | 71.81 | -139.76 | 141.72 |  | 0.14 | 85.11 | -166.68 | 166.95 |  | 7.09 | 22.71 | -37.42 | 51.59 |
| 2033 | 40-44 years | 8.44 | 28.63 | -47.68 | 64.56 |  | 0.98 | 76.31 | -148.58 | 150.54 |  | 0.14 | 88.72 | -173.75 | 174.02 |  | 7.08 | 24.39 | -40.72 | 54.87 |
| 2034 | 40-44 years | 8.43 | 30.86 | -52.06 | 68.92 |  | 0.98 | 80.74 | -157.27 | 159.23 |  | 0.14 | 92.22 | -180.61 | 180.88 |  | 7.05 | 26.28 | -44.47 | 58.56 |
| 2035 | 40-44 years | 8.42 | 33.19 | -56.64 | 73.48 |  | 0.98 | 84.92 | -165.47 | 167.43 |  | 0.13 | 95.39 | -186.83 | 187.10 |  | 7.00 | 28.41 | -48.69 | 62.69 |
| 1990 | 45-49 years | 14.17 | 12.69 | -10.71 | 39.05 |  | 1.68 | 18.19 | -33.98 | 37.34 |  | 0.41 | 23.63 | -45.91 | 46.73 |  | 18.11 | 12.35 | -6.10 | 42.32 |
| 1991 | 45-49 years | 14.08 | 12.70 | -10.82 | 38.98 |  | 1.67 | 18.14 | -33.88 | 37.23 |  | 0.40 | 25.53 | -49.63 | 50.44 |  | 18.04 | 12.36 | -6.19 | 42.26 |
| 1992 | 45-49 years | 14.31 | 12.70 | -10.58 | 39.20 |  | 1.69 | 18.86 | -35.27 | 38.65 |  | 0.40 | 26.32 | -51.18 | 51.98 |  | 18.13 | 12.35 | -6.08 | 42.34 |
| 1993 | 45-49 years | 14.56 | 12.69 | -10.30 | 39.43 |  | 1.71 | 19.21 | -35.94 | 39.37 |  | 0.40 | 27.34 | -53.18 | 53.99 |  | 18.49 | 12.32 | -5.66 | 42.65 |
| 1994 | 45-49 years | 14.67 | 12.67 | -10.16 | 39.51 |  | 1.73 | 18.69 | -34.89 | 38.36 |  | 0.41 | 27.43 | -53.35 | 54.16 |  | 18.66 | 12.31 | -5.47 | 42.79 |
| 1995 | 45-49 years | 14.90 | 12.66 | -9.91 | 39.71 |  | 1.76 | 19.16 | -35.79 | 39.30 |  | 0.41 | 27.43 | -53.35 | 54.17 |  | 18.93 | 12.29 | -5.16 | 43.03 |
| 1996 | 45-49 years | 14.91 | 12.66 | -9.90 | 39.73 |  | 1.76 | 19.12 | -35.72 | 39.24 |  | 0.41 | 25.88 | -50.31 | 51.14 |  | 18.66 | 12.31 | -5.47 | 42.80 |
| 1997 | 45-49 years | 14.61 | 12.68 | -10.23 | 39.46 |  | 1.74 | 18.27 | -34.07 | 37.55 |  | 0.41 | 24.37 | -47.36 | 48.17 |  | 18.13 | 12.36 | -6.09 | 42.34 |
| 1998 | 45-49 years | 14.24 | 12.70 | -10.65 | 39.14 |  | 1.71 | 17.62 | -32.82 | 36.24 |  | 0.40 | 23.03 | -44.73 | 45.53 |  | 17.64 | 12.39 | -6.65 | 41.94 |
| 1999 | 45-49 years | 13.97 | 12.73 | -10.98 | 38.92 |  | 1.68 | 17.58 | -32.79 | 36.15 |  | 0.40 | 23.99 | -46.63 | 47.42 |  | 17.70 | 12.39 | -6.58 | 41.99 |
| 2000 | 45-49 years | 13.72 | 12.75 | -11.28 | 38.71 |  | 1.66 | 17.16 | -31.98 | 35.29 |  | 0.40 | 23.80 | -46.25 | 47.04 |  | 17.62 | 12.40 | -6.68 | 41.92 |
| 2001 | 45-49 years | 13.64 | 12.77 | -11.38 | 38.66 |  | 1.65 | 17.29 | -32.24 | 35.53 |  | 0.39 | 23.05 | -44.79 | 45.57 |  | 17.42 | 12.41 | -6.91 | 41.75 |
| 2002 | 45-49 years | 13.60 | 12.77 | -11.43 | 38.62 |  | 1.64 | 16.96 | -31.61 | 34.89 |  | 0.39 | 21.99 | -42.71 | 43.50 |  | 17.36 | 12.42 | -6.98 | 41.70 |
| 2003 | 45-49 years | 13.66 | 12.76 | -11.36 | 38.68 |  | 1.65 | 16.93 | -31.54 | 34.83 |  | 0.39 | 21.38 | -41.51 | 42.30 |  | 17.41 | 12.42 | -6.93 | 41.74 |
| 2004 | 45-49 years | 13.75 | 12.76 | -11.25 | 38.76 |  | 1.66 | 16.97 | -31.60 | 34.91 |  | 0.39 | 20.78 | -40.34 | 41.13 |  | 17.37 | 12.42 | -6.97 | 41.71 |
| 2005 | 45-49 years | 13.83 | 12.75 | -11.16 | 38.82 |  | 1.66 | 17.01 | -31.68 | 35.00 |  | 0.39 | 20.81 | -40.40 | 41.19 |  | 17.46 | 12.41 | -6.87 | 41.78 |
| 2006 | 45-49 years | 13.88 | 12.75 | -11.12 | 38.87 |  | 1.66 | 17.32 | -32.29 | 35.62 |  | 0.39 | 19.24 | -37.32 | 38.09 |  | 16.86 | 12.46 | -7.57 | 41.28 |
| 2007 | 45-49 years | 13.85 | 12.76 | -11.16 | 38.86 |  | 1.65 | 17.84 | -33.31 | 36.62 |  | 0.38 | 18.88 | -36.62 | 37.37 |  | 16.37 | 12.50 | -8.14 | 40.87 |
| 2008 | 45-49 years | 13.71 | 12.78 | -11.34 | 38.75 |  | 1.63 | 18.28 | -34.20 | 37.45 |  | 0.36 | 18.24 | -35.39 | 36.12 |  | 15.72 | 12.56 | -8.90 | 40.34 |
| 2009 | 45-49 years | 13.47 | 12.81 | -11.63 | 38.57 |  | 1.60 | 18.66 | -34.97 | 38.16 |  | 0.35 | 17.74 | -34.41 | 35.11 |  | 14.95 | 12.63 | -9.81 | 39.71 |
| 2010 | 45-49 years | 13.19 | 12.84 | -11.97 | 38.35 |  | 1.56 | 19.00 | -35.68 | 38.79 |  | 0.33 | 18.90 | -36.72 | 37.39 |  | 14.52 | 12.68 | -10.33 | 39.36 |
| 2011 | 45-49 years | 12.82 | 12.87 | -12.41 | 38.06 |  | 1.51 | 18.76 | -35.25 | 38.28 |  | 0.32 | 19.13 | -37.17 | 37.80 |  | 13.85 | 12.74 | -11.13 | 38.83 |
| 2012 | 45-49 years | 12.50 | 12.90 | -12.79 | 37.80 |  | 1.47 | 18.49 | -34.76 | 37.70 |  | 0.30 | 19.69 | -38.29 | 38.90 |  | 13.37 | 12.79 | -11.71 | 38.45 |
| 2013 | 45-49 years | 12.23 | 12.93 | -13.11 | 37.57 |  | 1.44 | 18.09 | -34.03 | 36.90 |  | 0.29 | 20.59 | -40.06 | 40.65 |  | 13.11 | 12.82 | -12.02 | 38.24 |
| 2014 | 45-49 years | 12.02 | 12.95 | -13.36 | 37.40 |  | 1.41 | 17.62 | -33.12 | 35.95 |  | 0.29 | 20.38 | -39.65 | 40.23 |  | 12.71 | 12.87 | -12.51 | 37.93 |
| 2015 | 45-49 years | 11.88 | 12.96 | -13.52 | 37.28 |  | 1.40 | 17.14 | -32.20 | 34.99 |  | 0.28 | 20.75 | -40.39 | 40.96 |  | 12.54 | 12.89 | -12.71 | 37.80 |
| 2016 | 45-49 years | 11.86 | 12.96 | -13.55 | 37.26 |  | 1.39 | 17.15 | -32.22 | 35.00 |  | 0.28 | 21.66 | -42.17 | 42.73 |  | 12.56 | 12.88 | -12.70 | 37.81 |
| 2017 | 45-49 years | 11.79 | 12.97 | -13.63 | 37.21 |  | 1.38 | 16.73 | -31.40 | 34.17 |  | 0.28 | 21.76 | -42.37 | 42.93 |  | 12.38 | 12.91 | -12.92 | 37.67 |
| 2018 | 45-49 years | 11.71 | 12.98 | -13.73 | 37.14 |  | 1.38 | 16.39 | -30.75 | 33.51 |  | 0.27 | 22.25 | -43.33 | 43.88 |  | 12.42 | 12.90 | -12.86 | 37.71 |
| 2019 | 45-49 years | 11.63 | 12.98 | -13.82 | 37.08 |  | 1.37 | 16.12 | -30.23 | 32.97 |  | 0.27 | 22.76 | -44.34 | 44.89 |  | 12.46 | 12.90 | -12.81 | 37.74 |
| 2020 | 45-49 years | 11.58 | 12.99 | -13.88 | 37.04 |  | 1.37 | 15.87 | -29.74 | 32.47 |  | 0.27 | 22.59 | -44.01 | 44.56 |  | 12.36 | 12.91 | -12.94 | 37.66 |
| 2021 | 45-49 years | 11.50 | 13.00 | -13.98 | 36.98 |  | 1.36 | 15.67 | -29.36 | 32.08 |  | 0.27 | 22.58 | -43.98 | 44.52 |  | 12.43 | 12.90 | -12.85 | 37.72 |
| 2022 | 45-49 years | 11.84 | 15.73 | -18.99 | 42.67 |  | 1.38 | 25.21 | -48.04 | 50.80 |  | 0.27 | 25.65 | -50.01 | 50.55 |  | 12.61 | 14.62 | -16.05 | 41.27 |
| 2023 | 45-49 years | 11.84 | 15.77 | -19.06 | 42.74 |  | 1.38 | 26.34 | -50.24 | 53.00 |  | 0.27 | 31.28 | -61.04 | 61.58 |  | 12.69 | 14.66 | -16.05 | 41.43 |
| 2024 | 45-49 years | 11.84 | 16.01 | -19.53 | 43.22 |  | 1.38 | 29.50 | -56.43 | 59.20 |  | 0.27 | 38.95 | -76.08 | 76.62 |  | 12.75 | 14.79 | -16.23 | 41.74 |
| 2025 | 45-49 years | 11.85 | 16.43 | -20.36 | 44.06 |  | 1.38 | 33.96 | -65.17 | 67.94 |  | 0.27 | 47.22 | -92.28 | 92.83 |  | 12.79 | 15.01 | -16.64 | 42.21 |
| 2026 | 45-49 years | 11.85 | 17.07 | -21.61 | 45.32 |  | 1.39 | 39.01 | -75.08 | 77.85 |  | 0.27 | 54.86 | -107.26 | 107.80 |  | 12.76 | 15.38 | -17.38 | 42.91 |
| 2027 | 45-49 years | 11.84 | 17.94 | -23.33 | 47.01 |  | 1.38 | 44.16 | -85.17 | 87.94 |  | 0.27 | 61.25 | -119.78 | 120.32 |  | 12.67 | 15.92 | -18.53 | 43.87 |
| 2028 | 45-49 years | 11.80 | 19.09 | -25.62 | 49.21 |  | 1.38 | 49.65 | -95.93 | 98.69 |  | 0.27 | 67.35 | -131.75 | 132.28 |  | 12.51 | 16.66 | -20.15 | 45.17 |
| 2029 | 45-49 years | 11.74 | 20.48 | -28.40 | 51.89 |  | 1.37 | 55.38 | -107.17 | 109.92 |  | 0.26 | 73.56 | -143.91 | 144.44 |  | 12.29 | 17.64 | -22.29 | 46.87 |
| 2030 | 45-49 years | 11.67 | 22.07 | -31.59 | 54.94 |  | 1.37 | 61.02 | -118.23 | 120.97 |  | 0.26 | 79.45 | -155.47 | 155.98 |  | 12.04 | 18.87 | -24.96 | 49.03 |
| 2031 | 45-49 years | 11.59 | 23.83 | -35.12 | 58.30 |  | 1.36 | 66.17 | -128.34 | 131.05 |  | 0.25 | 83.97 | -164.33 | 164.83 |  | 11.80 | 20.25 | -27.89 | 51.49 |
| 2032 | 45-49 years | 11.51 | 25.72 | -38.91 | 61.93 |  | 1.35 | 70.66 | -137.14 | 139.83 |  | 0.25 | 87.08 | -170.44 | 170.93 |  | 11.61 | 21.74 | -31.00 | 54.21 |
| 2033 | 45-49 years | 11.43 | 27.80 | -43.05 | 65.91 |  | 1.34 | 75.12 | -145.90 | 148.58 |  | 0.25 | 90.36 | -176.87 | 177.36 |  | 11.49 | 23.37 | -34.32 | 57.30 |
| 2034 | 45-49 years | 11.38 | 30.02 | -47.46 | 70.23 |  | 1.33 | 79.67 | -154.81 | 157.48 |  | 0.25 | 94.38 | -184.74 | 185.23 |  | 11.46 | 25.19 | -37.92 | 60.83 |
| 2035 | 45-49 years | 11.36 | 32.32 | -51.99 | 74.72 |  | 1.33 | 84.05 | -163.41 | 166.07 |  | 0.25 | 98.40 | -192.62 | 193.11 |  | 11.48 | 27.17 | -41.78 | 64.74 |
| 1990 | 50-54 years | 20.39 | 12.16 | -3.44 | 44.22 |  | 2.50 | 14.26 | -25.45 | 30.44 |  | 0.78 | 17.58 | -33.67 | 35.23 |  | 29.64 | 11.65 | 6.81 | 52.48 |
| 1991 | 50-54 years | 20.59 | 12.15 | -3.23 | 44.41 |  | 2.51 | 14.72 | -26.34 | 31.36 |  | 0.78 | 18.97 | -36.40 | 37.96 |  | 29.90 | 11.64 | 7.09 | 52.72 |
| 1992 | 50-54 years | 20.74 | 12.15 | -3.07 | 44.55 |  | 2.51 | 15.48 | -27.84 | 32.86 |  | 0.78 | 21.44 | -41.25 | 42.80 |  | 30.34 | 11.62 | 7.56 | 53.11 |
| 1993 | 50-54 years | 20.79 | 12.15 | -3.03 | 44.60 |  | 2.50 | 16.21 | -29.26 | 34.27 |  | 0.77 | 24.45 | -47.16 | 48.69 |  | 30.50 | 11.61 | 7.74 | 53.26 |
| 1994 | 50-54 years | 20.79 | 12.16 | -3.05 | 44.63 |  | 2.49 | 17.02 | -30.87 | 35.85 |  | 0.74 | 25.93 | -50.07 | 51.56 |  | 29.84 | 11.64 | 7.02 | 52.66 |
| 1995 | 50-54 years | 20.61 | 12.18 | -3.26 | 44.49 |  | 2.47 | 17.20 | -31.24 | 36.18 |  | 0.72 | 26.47 | -51.17 | 52.61 |  | 28.86 | 11.69 | 5.95 | 51.78 |
| 1996 | 50-54 years | 20.32 | 12.19 | -3.58 | 44.21 |  | 2.44 | 16.46 | -29.82 | 34.70 |  | 0.70 | 26.73 | -51.69 | 53.09 |  | 28.04 | 11.73 | 5.05 | 51.04 |
| 1997 | 50-54 years | 20.47 | 12.19 | -3.41 | 44.36 |  | 2.46 | 16.79 | -30.44 | 35.36 |  | 0.68 | 25.35 | -49.00 | 50.37 |  | 27.27 | 11.77 | 4.20 | 50.34 |
| 1998 | 50-54 years | 20.66 | 12.17 | -3.20 | 44.52 |  | 2.49 | 16.90 | -30.64 | 35.62 |  | 0.68 | 24.66 | -47.65 | 49.01 |  | 27.09 | 11.78 | 4.00 | 50.18 |
| 1999 | 50-54 years | 20.71 | 12.16 | -3.13 | 44.55 |  | 2.51 | 16.38 | -29.60 | 34.62 |  | 0.68 | 23.87 | -46.11 | 47.48 |  | 27.14 | 11.78 | 4.05 | 50.22 |
| 2000 | 50-54 years | 21.00 | 12.15 | -2.82 | 44.82 |  | 2.54 | 16.99 | -30.75 | 35.84 |  | 0.70 | 23.78 | -45.91 | 47.31 |  | 27.85 | 11.74 | 4.83 | 50.86 |
| 2001 | 50-54 years | 21.08 | 12.15 | -2.73 | 44.89 |  | 2.55 | 17.16 | -31.08 | 36.18 |  | 0.70 | 21.96 | -42.35 | 43.75 |  | 27.75 | 11.75 | 4.73 | 50.78 |
| 2002 | 50-54 years | 20.81 | 12.17 | -3.04 | 44.65 |  | 2.52 | 16.82 | -30.45 | 35.49 |  | 0.70 | 21.03 | -40.53 | 41.92 |  | 27.57 | 11.76 | 4.53 | 50.62 |
| 2003 | 50-54 years | 20.46 | 12.19 | -3.43 | 44.35 |  | 2.48 | 16.70 | -30.25 | 35.22 |  | 0.69 | 20.98 | -40.43 | 41.81 |  | 27.36 | 11.77 | 4.29 | 50.43 |
| 2004 | 50-54 years | 20.24 | 12.21 | -3.69 | 44.17 |  | 2.45 | 17.06 | -30.99 | 35.89 |  | 0.68 | 19.86 | -38.25 | 39.60 |  | 26.61 | 11.81 | 3.46 | 49.76 |
| 2005 | 50-54 years | 19.96 | 12.23 | -4.01 | 43.93 |  | 2.41 | 16.87 | -30.66 | 35.48 |  | 0.67 | 20.11 | -38.74 | 40.08 |  | 26.25 | 11.83 | 3.06 | 49.44 |
| 2006 | 50-54 years | 19.91 | 12.24 | -4.07 | 43.89 |  | 2.40 | 17.18 | -31.27 | 36.07 |  | 0.65 | 19.29 | -37.15 | 38.46 |  | 25.58 | 11.87 | 2.32 | 48.84 |
| 2007 | 50-54 years | 19.92 | 12.24 | -4.06 | 43.90 |  | 2.40 | 17.15 | -31.21 | 36.01 |  | 0.65 | 18.60 | -35.81 | 37.10 |  | 25.19 | 11.89 | 1.89 | 48.49 |
| 2008 | 50-54 years | 20.06 | 12.23 | -3.91 | 44.02 |  | 2.41 | 17.33 | -31.55 | 36.37 |  | 0.64 | 18.18 | -35.00 | 36.28 |  | 25.08 | 11.90 | 1.76 | 48.39 |
| 2009 | 50-54 years | 20.22 | 12.22 | -3.73 | 44.17 |  | 2.42 | 17.57 | -32.01 | 36.85 |  | 0.64 | 17.98 | -34.59 | 35.87 |  | 24.94 | 11.90 | 1.61 | 48.27 |
| 2010 | 50-54 years | 20.32 | 12.21 | -3.62 | 44.26 |  | 2.42 | 17.76 | -32.38 | 37.23 |  | 0.63 | 18.06 | -34.77 | 36.04 |  | 24.76 | 11.91 | 1.41 | 48.11 |
| 2011 | 50-54 years | 20.23 | 12.22 | -3.72 | 44.18 |  | 2.41 | 17.77 | -32.43 | 37.25 |  | 0.62 | 18.04 | -34.74 | 35.98 |  | 24.26 | 11.94 | 0.85 | 47.67 |
| 2012 | 50-54 years | 19.90 | 12.24 | -4.09 | 43.89 |  | 2.36 | 17.50 | -31.94 | 36.66 |  | 0.60 | 18.54 | -35.74 | 36.95 |  | 23.67 | 11.98 | 0.19 | 47.15 |
| 2013 | 50-54 years | 19.34 | 12.28 | -4.72 | 43.40 |  | 2.29 | 16.99 | -31.02 | 35.60 |  | 0.59 | 18.89 | -36.43 | 37.60 |  | 22.99 | 12.02 | -0.57 | 46.55 |
| 2014 | 50-54 years | 18.73 | 12.32 | -5.41 | 42.88 |  | 2.22 | 16.57 | -30.26 | 34.70 |  | 0.56 | 19.43 | -37.51 | 38.64 |  | 22.25 | 12.07 | -1.40 | 45.91 |
| 2015 | 50-54 years | 18.18 | 12.36 | -6.04 | 42.40 |  | 2.15 | 16.39 | -29.97 | 34.27 |  | 0.54 | 19.29 | -37.26 | 38.35 |  | 21.29 | 12.13 | -2.49 | 45.07 |
| 2016 | 50-54 years | 17.64 | 12.40 | -6.67 | 41.94 |  | 2.09 | 16.14 | -29.54 | 33.72 |  | 0.52 | 19.52 | -37.74 | 38.78 |  | 20.45 | 12.19 | -3.44 | 44.34 |
| 2017 | 50-54 years | 17.22 | 12.43 | -7.15 | 41.59 |  | 2.04 | 16.02 | -29.35 | 33.43 |  | 0.50 | 19.32 | -37.38 | 38.37 |  | 19.48 | 12.26 | -4.54 | 43.51 |
| 2018 | 50-54 years | 16.91 | 12.46 | -7.50 | 41.33 |  | 2.01 | 15.94 | -29.22 | 33.25 |  | 0.48 | 20.53 | -39.76 | 40.72 |  | 19.15 | 12.28 | -4.93 | 43.22 |
| 2019 | 50-54 years | 16.72 | 12.47 | -7.73 | 41.16 |  | 1.99 | 15.86 | -29.10 | 33.08 |  | 0.47 | 21.17 | -41.02 | 41.96 |  | 18.76 | 12.31 | -5.38 | 42.89 |
| 2020 | 50-54 years | 16.63 | 12.48 | -7.83 | 41.09 |  | 1.98 | 15.80 | -28.98 | 32.95 |  | 0.46 | 21.42 | -41.52 | 42.44 |  | 18.39 | 12.34 | -5.79 | 42.58 |
| 2021 | 50-54 years | 16.59 | 12.48 | -7.88 | 41.05 |  | 1.97 | 15.45 | -28.31 | 32.25 |  | 0.46 | 21.94 | -42.55 | 43.46 |  | 18.39 | 12.34 | -5.80 | 42.58 |
| 2022 | 50-54 years | 16.96 | 14.88 | -12.20 | 46.12 |  | 1.99 | 24.44 | -45.91 | 49.89 |  | 0.45 | 26.09 | -50.69 | 51.59 |  | 18.27 | 14.10 | -9.37 | 45.90 |
| 2023 | 50-54 years | 16.95 | 14.95 | -12.35 | 46.24 |  | 1.99 | 25.75 | -48.47 | 52.45 |  | 0.45 | 32.58 | -63.40 | 64.30 |  | 18.26 | 14.13 | -9.44 | 45.96 |
| 2024 | 50-54 years | 16.92 | 15.27 | -13.01 | 46.86 |  | 1.99 | 29.05 | -54.95 | 58.92 |  | 0.45 | 41.11 | -80.13 | 81.03 |  | 18.31 | 14.27 | -9.66 | 46.28 |
| 2025 | 50-54 years | 16.90 | 15.80 | -14.06 | 47.86 |  | 1.98 | 33.55 | -63.77 | 67.74 |  | 0.45 | 50.16 | -97.86 | 98.75 |  | 18.41 | 14.53 | -10.06 | 46.88 |
| 2026 | 50-54 years | 16.88 | 16.49 | -15.45 | 49.21 |  | 1.98 | 38.53 | -73.54 | 77.51 |  | 0.45 | 58.26 | -113.75 | 114.64 |  | 18.52 | 14.92 | -10.72 | 47.75 |
| 2027 | 50-54 years | 16.87 | 17.35 | -17.14 | 50.88 |  | 1.98 | 43.49 | -83.26 | 87.23 |  | 0.45 | 64.57 | -126.11 | 127.01 |  | 18.63 | 15.43 | -11.62 | 48.88 |
| 2028 | 50-54 years | 16.87 | 18.44 | -19.28 | 53.01 |  | 1.98 | 48.78 | -93.62 | 97.59 |  | 0.45 | 70.50 | -137.73 | 138.63 |  | 18.75 | 16.08 | -12.77 | 50.26 |
| 2029 | 50-54 years | 16.87 | 19.75 | -21.83 | 55.58 |  | 1.99 | 54.31 | -104.46 | 108.43 |  | 0.45 | 76.52 | -149.52 | 150.42 |  | 18.84 | 16.87 | -14.23 | 51.91 |
| 2030 | 50-54 years | 16.89 | 21.22 | -24.70 | 58.48 |  | 1.99 | 59.77 | -115.16 | 119.14 |  | 0.45 | 82.14 | -160.54 | 161.44 |  | 18.89 | 17.83 | -16.07 | 53.84 |
| 2031 | 50-54 years | 16.89 | 22.83 | -27.86 | 61.63 |  | 1.99 | 64.74 | -124.89 | 128.87 |  | 0.45 | 86.63 | -169.35 | 170.25 |  | 18.85 | 18.97 | -18.33 | 56.03 |
| 2032 | 50-54 years | 16.87 | 24.57 | -31.29 | 65.02 |  | 1.99 | 69.04 | -133.33 | 137.31 |  | 0.45 | 89.82 | -175.61 | 176.50 |  | 18.71 | 20.30 | -21.08 | 58.50 |
| 2033 | 50-54 years | 16.81 | 26.52 | -35.18 | 68.80 |  | 1.98 | 73.40 | -141.89 | 145.85 |  | 0.44 | 93.06 | -181.95 | 182.83 |  | 18.47 | 21.87 | -24.39 | 61.34 |
| 2034 | 50-54 years | 16.73 | 28.67 | -39.45 | 72.92 |  | 1.97 | 77.84 | -150.59 | 154.54 |  | 0.43 | 96.67 | -189.04 | 189.91 |  | 18.15 | 23.72 | -28.34 | 64.64 |
| 2035 | 50-54 years | 16.64 | 30.90 | -43.94 | 77.21 |  | 1.96 | 82.07 | -158.89 | 162.81 |  | 0.43 | 100.21 | -195.98 | 196.83 |  | 17.78 | 25.85 | -32.88 | 68.44 |
| 1990 | 55-59 years | 28.53 | 11.69 | 5.61 | 51.45 |  | 3.56 | 13.78 | -23.44 | 30.56 |  | 1.39 | 15.33 | -28.66 | 31.44 |  | 46.20 | 11.02 | 24.60 | 67.79 |
| 1991 | 55-59 years | 28.33 | 11.70 | 5.40 | 51.27 |  | 3.54 | 13.79 | -23.49 | 30.57 |  | 1.37 | 16.36 | -30.69 | 33.43 |  | 45.73 | 11.03 | 24.10 | 67.35 |
| 1992 | 55-59 years | 28.34 | 11.70 | 5.40 | 51.28 |  | 3.54 | 13.83 | -23.57 | 30.66 |  | 1.37 | 17.61 | -33.15 | 35.89 |  | 46.17 | 11.02 | 24.57 | 67.77 |
| 1993 | 55-59 years | 28.49 | 11.70 | 5.56 | 51.41 |  | 3.57 | 13.92 | -23.72 | 30.86 |  | 1.39 | 19.53 | -36.89 | 39.66 |  | 47.37 | 10.98 | 25.85 | 68.90 |
| 1994 | 55-59 years | 28.72 | 11.69 | 5.81 | 51.62 |  | 3.60 | 14.11 | -24.06 | 31.26 |  | 1.40 | 21.58 | -40.89 | 43.70 |  | 48.67 | 10.95 | 27.22 | 70.13 |
| 1995 | 55-59 years | 28.91 | 11.68 | 6.01 | 51.80 |  | 3.62 | 14.34 | -24.49 | 31.72 |  | 1.40 | 21.41 | -40.56 | 43.37 |  | 48.53 | 10.95 | 27.07 | 70.00 |
| 1996 | 55-59 years | 29.02 | 11.68 | 6.14 | 51.91 |  | 3.63 | 14.79 | -25.35 | 32.61 |  | 1.38 | 21.15 | -40.08 | 42.85 |  | 47.81 | 10.97 | 26.31 | 69.31 |
| 1997 | 55-59 years | 29.03 | 11.68 | 6.14 | 51.92 |  | 3.63 | 15.29 | -26.34 | 33.59 |  | 1.35 | 20.34 | -38.52 | 41.22 |  | 46.40 | 11.01 | 24.82 | 67.99 |
| 1998 | 55-59 years | 28.91 | 11.69 | 6.00 | 51.82 |  | 3.60 | 15.70 | -27.16 | 34.36 |  | 1.31 | 20.31 | -38.51 | 41.13 |  | 44.95 | 11.06 | 23.27 | 66.62 |
| 1999 | 55-59 years | 28.74 | 11.70 | 5.80 | 51.68 |  | 3.57 | 16.18 | -28.14 | 35.28 |  | 1.28 | 22.77 | -43.36 | 45.92 |  | 44.44 | 11.08 | 22.73 | 66.15 |
| 2000 | 55-59 years | 28.43 | 11.72 | 5.46 | 51.40 |  | 3.53 | 16.25 | -28.32 | 35.38 |  | 1.25 | 22.82 | -43.48 | 45.97 |  | 43.32 | 11.11 | 21.54 | 65.10 |
| 2001 | 55-59 years | 28.09 | 11.73 | 5.10 | 51.08 |  | 3.50 | 15.74 | -27.35 | 34.34 |  | 1.21 | 21.78 | -41.47 | 43.90 |  | 42.03 | 11.15 | 20.16 | 63.89 |
| 2002 | 55-59 years | 28.47 | 11.72 | 5.50 | 51.44 |  | 3.53 | 16.30 | -28.42 | 35.47 |  | 1.20 | 20.31 | -38.61 | 41.01 |  | 41.37 | 11.18 | 19.46 | 63.28 |
| 2003 | 55-59 years | 28.91 | 11.70 | 5.98 | 51.84 |  | 3.57 | 16.60 | -28.96 | 36.10 |  | 1.20 | 19.87 | -37.75 | 40.15 |  | 41.34 | 11.18 | 19.43 | 63.25 |
| 2004 | 55-59 years | 29.12 | 11.69 | 6.22 | 52.03 |  | 3.60 | 16.24 | -28.23 | 35.43 |  | 1.19 | 18.11 | -34.31 | 36.69 |  | 40.74 | 11.20 | 18.79 | 62.69 |
| 2005 | 55-59 years | 29.62 | 11.67 | 6.76 | 52.49 |  | 3.65 | 16.73 | -29.14 | 36.44 |  | 1.20 | 19.18 | -36.40 | 38.81 |  | 41.64 | 11.17 | 19.75 | 63.54 |
| 2006 | 55-59 years | 29.75 | 11.66 | 6.90 | 52.61 |  | 3.66 | 16.81 | -29.28 | 36.60 |  | 1.19 | 17.78 | -33.66 | 36.05 |  | 41.04 | 11.19 | 19.10 | 62.97 |
| 2007 | 55-59 years | 29.39 | 11.68 | 6.50 | 52.28 |  | 3.62 | 16.57 | -28.85 | 36.09 |  | 1.18 | 16.55 | -31.25 | 33.61 |  | 40.06 | 11.23 | 18.05 | 62.06 |
| 2008 | 55-59 years | 28.89 | 11.70 | 5.96 | 51.83 |  | 3.56 | 16.48 | -28.75 | 35.87 |  | 1.16 | 16.43 | -31.05 | 33.36 |  | 39.36 | 11.25 | 17.31 | 61.41 |
| 2009 | 55-59 years | 28.50 | 11.72 | 5.53 | 51.48 |  | 3.51 | 16.73 | -29.28 | 36.29 |  | 1.13 | 17.27 | -32.71 | 34.98 |  | 38.78 | 11.27 | 16.68 | 60.87 |
| 2010 | 55-59 years | 27.97 | 11.75 | 4.94 | 51.00 |  | 3.44 | 16.47 | -28.85 | 35.73 |  | 1.10 | 17.46 | -33.11 | 35.32 |  | 37.84 | 11.31 | 15.67 | 60.00 |
| 2011 | 55-59 years | 27.69 | 11.76 | 4.64 | 50.75 |  | 3.40 | 16.53 | -29.00 | 35.80 |  | 1.08 | 16.96 | -32.17 | 34.33 |  | 36.78 | 11.35 | 14.54 | 59.03 |
| 2012 | 55-59 years | 27.41 | 11.78 | 4.33 | 50.49 |  | 3.36 | 16.22 | -28.43 | 35.15 |  | 1.06 | 17.50 | -33.23 | 35.36 |  | 36.50 | 11.36 | 14.23 | 58.77 |
| 2013 | 55-59 years | 27.27 | 11.78 | 4.18 | 50.36 |  | 3.33 | 16.03 | -28.08 | 34.74 |  | 1.06 | 16.75 | -31.78 | 33.90 |  | 36.11 | 11.38 | 13.82 | 58.41 |
| 2014 | 55-59 years | 27.20 | 11.78 | 4.11 | 50.30 |  | 3.31 | 15.89 | -27.84 | 34.46 |  | 1.06 | 17.47 | -33.18 | 35.30 |  | 36.38 | 11.37 | 14.10 | 58.66 |
| 2015 | 55-59 years | 27.15 | 11.79 | 4.05 | 50.26 |  | 3.30 | 15.86 | -27.79 | 34.39 |  | 1.06 | 18.20 | -34.60 | 36.72 |  | 36.51 | 11.36 | 14.24 | 58.78 |
| 2016 | 55-59 years | 26.99 | 11.79 | 3.87 | 50.11 |  | 3.28 | 15.88 | -27.84 | 34.39 |  | 1.05 | 19.61 | -37.39 | 39.49 |  | 36.56 | 11.36 | 14.30 | 58.83 |
| 2017 | 55-59 years | 26.56 | 11.82 | 3.40 | 49.72 |  | 3.23 | 15.76 | -27.66 | 34.12 |  | 1.02 | 19.72 | -37.62 | 39.66 |  | 35.49 | 11.40 | 13.15 | 57.84 |
| 2018 | 55-59 years | 25.91 | 11.85 | 2.69 | 49.14 |  | 3.16 | 15.58 | -27.37 | 33.70 |  | 0.99 | 20.54 | -39.27 | 41.25 |  | 34.64 | 11.44 | 12.22 | 57.06 |
| 2019 | 55-59 years | 25.23 | 11.89 | 1.94 | 48.53 |  | 3.09 | 15.57 | -27.42 | 33.60 |  | 0.95 | 20.85 | -39.91 | 41.82 |  | 33.35 | 11.49 | 10.83 | 55.87 |
| 2020 | 55-59 years | 24.62 | 11.92 | 1.25 | 47.99 |  | 3.02 | 15.78 | -27.91 | 33.94 |  | 0.91 | 20.45 | -39.17 | 40.98 |  | 31.68 | 11.57 | 9.01 | 54.35 |
| 2021 | 55-59 years | 23.90 | 11.96 | 0.45 | 47.35 |  | 2.93 | 15.33 | -27.13 | 32.98 |  | 0.87 | 20.75 | -39.80 | 41.54 |  | 30.59 | 11.62 | 7.83 | 53.36 |
| 2022 | 55-59 years | 23.95 | 14.70 | -4.86 | 52.76 |  | 2.89 | 24.26 | -44.66 | 50.45 |  | 0.83 | 25.82 | -49.77 | 51.43 |  | 29.47 | 13.43 | 3.15 | 55.79 |
| 2023 | 55-59 years | 23.66 | 14.72 | -5.19 | 52.50 |  | 2.85 | 25.41 | -46.94 | 52.65 |  | 0.81 | 32.99 | -63.86 | 65.48 |  | 28.66 | 13.53 | 2.15 | 55.17 |
| 2024 | 55-59 years | 23.48 | 14.91 | -5.74 | 52.69 |  | 2.83 | 28.51 | -53.04 | 58.71 |  | 0.79 | 42.35 | -82.22 | 83.80 |  | 28.03 | 13.70 | 1.18 | 54.88 |
| 2025 | 55-59 years | 23.40 | 15.27 | -6.53 | 53.33 |  | 2.82 | 32.86 | -61.59 | 67.23 |  | 0.78 | 52.11 | -101.37 | 102.92 |  | 27.57 | 13.97 | 0.19 | 54.96 |
| 2026 | 55-59 years | 23.38 | 15.80 | -7.58 | 54.35 |  | 2.82 | 37.73 | -71.13 | 76.76 |  | 0.77 | 60.67 | -118.15 | 119.68 |  | 27.29 | 14.34 | -0.82 | 55.39 |
| 2027 | 55-59 years | 23.39 | 16.57 | -9.09 | 55.86 |  | 2.82 | 42.63 | -80.74 | 86.38 |  | 0.76 | 67.15 | -130.85 | 132.37 |  | 27.14 | 14.82 | -1.89 | 56.18 |
| 2028 | 55-59 years | 23.37 | 17.65 | -11.22 | 57.96 |  | 2.81 | 47.92 | -91.11 | 96.73 |  | 0.76 | 73.10 | -142.52 | 144.04 |  | 27.13 | 15.42 | -3.10 | 57.36 |
| 2029 | 55-59 years | 23.34 | 18.98 | -13.86 | 60.53 |  | 2.81 | 53.39 | -101.83 | 107.45 |  | 0.76 | 79.02 | -154.13 | 155.65 |  | 27.21 | 16.19 | -4.53 | 58.95 |
| 2030 | 55-59 years | 23.30 | 20.48 | -16.83 | 63.44 |  | 2.80 | 58.70 | -112.25 | 117.86 |  | 0.76 | 84.59 | -165.03 | 166.55 |  | 27.35 | 17.14 | -6.24 | 60.94 |
| 2031 | 55-59 years | 23.28 | 22.08 | -19.99 | 66.55 |  | 2.80 | 63.48 | -121.62 | 127.22 |  | 0.76 | 88.95 | -173.57 | 175.09 |  | 27.52 | 18.25 | -8.26 | 63.30 |
| 2032 | 55-59 years | 23.26 | 23.75 | -23.29 | 69.82 |  | 2.80 | 67.55 | -129.60 | 135.21 |  | 0.76 | 91.73 | -179.03 | 180.56 |  | 27.69 | 19.53 | -10.59 | 65.97 |
| 2033 | 55-59 years | 23.26 | 25.62 | -26.95 | 73.47 |  | 2.80 | 71.70 | -137.73 | 143.34 |  | 0.76 | 94.52 | -184.49 | 186.02 |  | 27.86 | 20.97 | -13.24 | 68.95 |
| 2034 | 55-59 years | 23.27 | 27.65 | -30.92 | 77.45 |  | 2.81 | 75.95 | -146.06 | 151.67 |  | 0.77 | 97.71 | -190.74 | 192.27 |  | 27.99 | 22.59 | -16.28 | 72.27 |
| 2035 | 55-59 years | 23.29 | 29.76 | -35.04 | 81.61 |  | 2.81 | 80.03 | -154.05 | 159.67 |  | 0.77 | 100.82 | -196.84 | 198.37 |  | 28.06 | 24.40 | -19.76 | 75.88 |
| 1990 | 60-64 years | 45.94 | 11.03 | 24.33 | 67.55 |  | 5.62 | 14.59 | -22.97 | 34.21 |  | 2.60 | 14.37 | -25.56 | 30.77 |  | 75.50 | 10.32 | 55.28 | 95.72 |
| 1991 | 60-64 years | 45.28 | 11.05 | 23.63 | 66.93 |  | 5.57 | 14.60 | -23.03 | 34.18 |  | 2.59 | 15.51 | -27.81 | 32.99 |  | 75.66 | 10.32 | 55.44 | 95.87 |
| 1992 | 60-64 years | 44.43 | 11.07 | 22.73 | 66.12 |  | 5.51 | 14.28 | -22.49 | 33.50 |  | 2.58 | 16.59 | -29.93 | 35.08 |  | 75.64 | 10.32 | 55.42 | 95.86 |
| 1993 | 60-64 years | 43.56 | 11.10 | 21.81 | 65.31 |  | 5.43 | 13.98 | -21.97 | 32.84 |  | 2.56 | 18.42 | -33.54 | 38.66 |  | 75.80 | 10.31 | 55.58 | 96.01 |
| 1994 | 60-64 years | 42.69 | 11.13 | 20.88 | 64.49 |  | 5.35 | 13.77 | -21.63 | 32.34 |  | 2.51 | 18.94 | -34.61 | 39.62 |  | 74.26 | 10.34 | 53.99 | 94.53 |
| 1995 | 60-64 years | 42.12 | 11.15 | 20.28 | 63.97 |  | 5.30 | 13.76 | -21.66 | 32.26 |  | 2.43 | 18.68 | -34.19 | 39.05 |  | 71.87 | 10.39 | 51.50 | 92.23 |
| 1996 | 60-64 years | 41.87 | 11.15 | 20.00 | 63.73 |  | 5.27 | 13.82 | -21.81 | 32.35 |  | 2.36 | 18.29 | -33.49 | 38.21 |  | 69.72 | 10.43 | 49.28 | 90.17 |
| 1997 | 60-64 years | 41.98 | 11.15 | 20.12 | 63.84 |  | 5.29 | 14.08 | -22.31 | 32.88 |  | 2.33 | 18.09 | -33.13 | 37.78 |  | 68.68 | 10.45 | 48.19 | 89.17 |
| 1998 | 60-64 years | 42.34 | 11.14 | 20.51 | 64.18 |  | 5.33 | 14.55 | -23.19 | 33.85 |  | 2.32 | 17.70 | -32.38 | 37.02 |  | 68.44 | 10.46 | 47.94 | 88.94 |
| 1999 | 60-64 years | 42.82 | 11.13 | 21.00 | 64.63 |  | 5.38 | 15.09 | -24.20 | 34.95 |  | 2.34 | 18.08 | -33.10 | 37.78 |  | 69.21 | 10.44 | 48.74 | 89.68 |
| 2000 | 60-64 years | 43.13 | 11.12 | 21.33 | 64.93 |  | 5.40 | 15.45 | -24.87 | 35.68 |  | 2.36 | 18.07 | -33.05 | 37.77 |  | 69.92 | 10.43 | 49.47 | 90.36 |
| 2001 | 60-64 years | 43.28 | 11.12 | 21.49 | 65.07 |  | 5.41 | 15.65 | -25.26 | 36.08 |  | 2.35 | 18.01 | -32.94 | 37.64 |  | 69.64 | 10.43 | 49.19 | 90.09 |
| 2002 | 60-64 years | 43.26 | 11.12 | 21.46 | 65.05 |  | 5.38 | 15.68 | -25.35 | 36.12 |  | 2.32 | 17.62 | -32.21 | 36.85 |  | 68.63 | 10.46 | 48.13 | 89.12 |
| 2003 | 60-64 years | 43.05 | 11.13 | 21.23 | 64.86 |  | 5.33 | 15.72 | -25.47 | 36.14 |  | 2.26 | 17.96 | -32.93 | 37.46 |  | 66.95 | 10.49 | 46.39 | 87.51 |
| 2004 | 60-64 years | 42.73 | 11.14 | 20.90 | 64.57 |  | 5.28 | 15.86 | -25.81 | 36.37 |  | 2.17 | 17.58 | -32.28 | 36.62 |  | 63.98 | 10.56 | 43.29 | 84.67 |
| 2005 | 60-64 years | 42.17 | 11.16 | 20.29 | 64.04 |  | 5.21 | 15.81 | -25.78 | 36.20 |  | 2.09 | 17.31 | -31.84 | 36.02 |  | 61.43 | 10.61 | 40.62 | 82.23 |
| 2006 | 60-64 years | 41.54 | 11.18 | 19.64 | 63.45 |  | 5.16 | 15.33 | -24.89 | 35.20 |  | 2.01 | 16.86 | -31.03 | 35.05 |  | 59.08 | 10.67 | 38.17 | 79.99 |
| 2007 | 60-64 years | 41.98 | 11.16 | 20.10 | 63.86 |  | 5.20 | 15.64 | -25.46 | 35.85 |  | 1.96 | 16.77 | -30.91 | 34.84 |  | 58.07 | 10.69 | 37.11 | 79.03 |
| 2008 | 60-64 years | 42.50 | 11.15 | 20.65 | 64.35 |  | 5.25 | 15.71 | -25.55 | 36.05 |  | 1.95 | 17.11 | -31.60 | 35.49 |  | 57.92 | 10.70 | 36.95 | 78.89 |
| 2009 | 60-64 years | 42.68 | 11.14 | 20.85 | 64.51 |  | 5.28 | 15.27 | -24.66 | 35.21 |  | 1.93 | 16.76 | -30.91 | 34.78 |  | 57.42 | 10.71 | 36.42 | 78.41 |
| 2010 | 60-64 years | 43.26 | 11.12 | 21.46 | 65.06 |  | 5.33 | 15.54 | -25.13 | 35.78 |  | 1.94 | 17.70 | -32.75 | 36.63 |  | 57.97 | 10.70 | 37.00 | 78.94 |
| 2011 | 60-64 years | 43.12 | 11.13 | 21.32 | 64.93 |  | 5.29 | 15.37 | -24.84 | 35.42 |  | 1.92 | 16.98 | -31.35 | 35.20 |  | 57.13 | 10.72 | 36.12 | 78.14 |
| 2012 | 60-64 years | 42.17 | 11.15 | 20.30 | 64.03 |  | 5.17 | 14.95 | -24.12 | 34.47 |  | 1.89 | 16.99 | -31.41 | 35.20 |  | 56.23 | 10.74 | 35.18 | 77.29 |
| 2013 | 60-64 years | 41.06 | 11.19 | 19.13 | 63.00 |  | 5.03 | 14.70 | -23.78 | 33.84 |  | 1.85 | 16.17 | -29.85 | 33.55 |  | 54.77 | 10.78 | 33.64 | 75.90 |
| 2014 | 60-64 years | 40.21 | 11.22 | 18.22 | 62.20 |  | 4.91 | 14.77 | -24.05 | 33.86 |  | 1.82 | 16.49 | -30.50 | 34.14 |  | 53.88 | 10.80 | 32.70 | 75.05 |
| 2015 | 60-64 years | 39.31 | 11.25 | 17.26 | 61.37 |  | 4.79 | 14.52 | -23.67 | 33.25 |  | 1.78 | 16.29 | -30.14 | 33.71 |  | 52.65 | 10.84 | 31.41 | 73.89 |
| 2016 | 60-64 years | 38.91 | 11.27 | 16.83 | 61.00 |  | 4.74 | 14.62 | -23.92 | 33.40 |  | 1.76 | 16.97 | -31.50 | 35.03 |  | 52.19 | 10.85 | 30.93 | 73.46 |
| 2017 | 60-64 years | 38.57 | 11.28 | 16.46 | 60.68 |  | 4.71 | 14.45 | -23.62 | 33.04 |  | 1.74 | 17.05 | -31.67 | 35.15 |  | 51.45 | 10.87 | 30.15 | 72.76 |
| 2018 | 60-64 years | 38.48 | 11.28 | 16.36 | 60.59 |  | 4.70 | 14.41 | -23.54 | 32.95 |  | 1.74 | 18.57 | -34.65 | 38.14 |  | 52.15 | 10.85 | 30.89 | 73.42 |
| 2019 | 60-64 years | 38.55 | 11.28 | 16.44 | 60.67 |  | 4.72 | 14.49 | -23.68 | 33.12 |  | 1.74 | 19.00 | -35.50 | 38.98 |  | 52.23 | 10.85 | 30.97 | 73.50 |
| 2020 | 60-64 years | 38.68 | 11.28 | 16.57 | 60.78 |  | 4.75 | 14.86 | -24.37 | 33.86 |  | 1.72 | 18.51 | -34.55 | 37.99 |  | 51.47 | 10.87 | 30.16 | 72.77 |
| 2021 | 60-64 years | 38.38 | 11.29 | 16.25 | 60.50 |  | 4.71 | 14.71 | -24.13 | 33.55 |  | 1.70 | 18.80 | -35.14 | 38.54 |  | 51.11 | 10.88 | 29.78 | 72.44 |
| 2022 | 60-64 years | 38.37 | 13.30 | 12.29 | 64.45 |  | 4.67 | 22.40 | -39.22 | 48.57 |  | 1.65 | 25.63 | -48.58 | 51.88 |  | 49.63 | 12.49 | 25.15 | 74.12 |
| 2023 | 60-64 years | 37.77 | 13.46 | 11.39 | 64.15 |  | 4.59 | 23.94 | -42.33 | 51.51 |  | 1.60 | 33.15 | -63.38 | 66.58 |  | 48.27 | 12.61 | 23.56 | 72.97 |
| 2024 | 60-64 years | 37.01 | 13.87 | 9.82 | 64.21 |  | 4.49 | 27.33 | -49.07 | 58.05 |  | 1.54 | 42.72 | -82.19 | 85.28 |  | 46.68 | 12.85 | 21.49 | 71.87 |
| 2025 | 60-64 years | 36.20 | 14.49 | 7.80 | 64.60 |  | 4.39 | 31.82 | -57.98 | 66.75 |  | 1.48 | 52.71 | -101.83 | 104.79 |  | 45.00 | 13.23 | 19.08 | 70.92 |
| 2026 | 60-64 years | 35.45 | 15.22 | 5.61 | 65.29 |  | 4.29 | 36.73 | -67.71 | 76.28 |  | 1.42 | 61.35 | -118.83 | 121.68 |  | 43.41 | 13.68 | 16.60 | 70.23 |
| 2027 | 60-64 years | 34.85 | 16.04 | 3.41 | 66.30 |  | 4.21 | 41.54 | -77.21 | 85.63 |  | 1.37 | 67.64 | -131.21 | 133.95 |  | 42.02 | 14.21 | 14.17 | 69.87 |
| 2028 | 60-64 years | 34.42 | 17.04 | 1.03 | 67.82 |  | 4.15 | 46.64 | -87.27 | 95.57 |  | 1.33 | 73.56 | -142.84 | 145.50 |  | 40.87 | 14.83 | 11.80 | 69.95 |
| 2029 | 60-64 years | 34.16 | 18.23 | -1.56 | 69.88 |  | 4.12 | 51.98 | -97.76 | 105.99 |  | 1.30 | 79.63 | -154.77 | 157.37 |  | 39.98 | 15.60 | 9.41 | 70.55 |
| 2030 | 60-64 years | 34.04 | 19.57 | -4.32 | 72.40 |  | 4.10 | 57.24 | -108.08 | 116.28 |  | 1.28 | 85.37 | -166.04 | 168.61 |  | 39.32 | 16.52 | 6.94 | 71.71 |
| 2031 | 60-64 years | 34.03 | 21.00 | -7.14 | 75.19 |  | 4.10 | 61.93 | -117.29 | 125.48 |  | 1.27 | 89.83 | -174.80 | 177.34 |  | 38.91 | 17.58 | 4.45 | 73.37 |
| 2032 | 60-64 years | 34.03 | 22.57 | -10.21 | 78.26 |  | 4.10 | 65.91 | -125.09 | 133.28 |  | 1.26 | 92.62 | -180.28 | 182.80 |  | 38.71 | 18.79 | 1.89 | 75.53 |
| 2033 | 60-64 years | 34.00 | 24.38 | -13.79 | 81.79 |  | 4.09 | 70.00 | -133.11 | 141.29 |  | 1.26 | 95.34 | -185.60 | 188.12 |  | 38.69 | 20.15 | -0.81 | 78.18 |
| 2034 | 60-64 years | 33.96 | 26.38 | -17.76 | 85.67 |  | 4.08 | 74.13 | -141.21 | 149.37 |  | 1.26 | 98.36 | -191.53 | 194.05 |  | 38.80 | 21.71 | -3.75 | 81.35 |
| 2035 | 60-64 years | 33.91 | 28.46 | -21.88 | 89.69 |  | 4.08 | 78.02 | -148.84 | 156.99 |  | 1.26 | 101.37 | -197.43 | 199.95 |  | 39.00 | 23.46 | -6.99 | 85.00 |
| 1990 | 65-69 years | 72.74 | 10.37 | 52.42 | 93.06 |  | 8.90 | 13.28 | -17.13 | 34.93 |  | 4.65 | 12.99 | -20.82 | 30.12 |  | 113.44 | 9.74 | 94.35 | 132.52 |
| 1991 | 65-69 years | 72.22 | 10.38 | 51.88 | 92.56 |  | 8.91 | 13.50 | -17.55 | 35.37 |  | 4.67 | 13.65 | -22.08 | 31.41 |  | 114.60 | 9.72 | 95.54 | 133.66 |
| 1992 | 65-69 years | 71.83 | 10.39 | 51.47 | 92.19 |  | 8.92 | 13.72 | -17.97 | 35.80 |  | 4.69 | 14.09 | -22.93 | 32.31 |  | 115.64 | 9.71 | 96.60 | 134.67 |
| 1993 | 65-69 years | 71.50 | 10.39 | 51.13 | 91.87 |  | 8.92 | 13.93 | -18.38 | 36.21 |  | 4.74 | 15.32 | -25.29 | 34.77 |  | 117.62 | 9.69 | 98.63 | 136.61 |
| 1994 | 65-69 years | 71.00 | 10.40 | 50.61 | 91.39 |  | 8.89 | 14.01 | -18.57 | 36.35 |  | 4.75 | 16.02 | -26.65 | 36.14 |  | 118.14 | 9.68 | 99.17 | 137.12 |
| 1995 | 65-69 years | 70.41 | 10.42 | 49.99 | 90.82 |  | 8.84 | 14.05 | -18.70 | 36.39 |  | 4.69 | 16.14 | -26.93 | 36.32 |  | 116.74 | 9.70 | 97.73 | 135.74 |
| 1996 | 65-69 years | 69.87 | 10.43 | 49.43 | 90.31 |  | 8.79 | 14.19 | -19.02 | 36.60 |  | 4.60 | 15.87 | -26.50 | 35.71 |  | 114.23 | 9.73 | 95.16 | 133.29 |
| 1997 | 65-69 years | 69.12 | 10.44 | 48.65 | 89.59 |  | 8.70 | 14.28 | -19.28 | 36.68 |  | 4.50 | 15.60 | -26.07 | 35.07 |  | 111.46 | 9.76 | 92.32 | 130.59 |
| 1998 | 65-69 years | 68.34 | 10.46 | 47.83 | 88.85 |  | 8.60 | 14.40 | -19.63 | 36.83 |  | 4.40 | 15.52 | -26.02 | 34.82 |  | 108.89 | 9.80 | 89.69 | 128.09 |
| 1999 | 65-69 years | 67.41 | 10.48 | 46.86 | 87.96 |  | 8.49 | 14.48 | -19.88 | 36.87 |  | 4.30 | 16.07 | -27.20 | 35.80 |  | 106.79 | 9.82 | 87.53 | 126.05 |
| 2000 | 65-69 years | 66.74 | 10.50 | 46.16 | 87.32 |  | 8.41 | 14.60 | -20.22 | 37.03 |  | 4.24 | 16.98 | -29.04 | 37.52 |  | 105.84 | 9.84 | 86.56 | 125.12 |
| 2001 | 65-69 years | 66.38 | 10.51 | 45.78 | 86.97 |  | 8.35 | 14.63 | -20.32 | 37.02 |  | 4.17 | 17.22 | -29.58 | 37.93 |  | 104.38 | 9.86 | 85.06 | 123.70 |
| 2002 | 65-69 years | 66.52 | 10.50 | 45.93 | 87.11 |  | 8.35 | 14.67 | -20.40 | 37.10 |  | 4.14 | 16.62 | -28.44 | 36.72 |  | 103.51 | 9.87 | 84.17 | 122.85 |
| 2003 | 65-69 years | 66.98 | 10.50 | 46.41 | 87.55 |  | 8.39 | 14.69 | -20.40 | 37.17 |  | 4.14 | 16.45 | -28.09 | 36.37 |  | 103.58 | 9.87 | 84.24 | 122.93 |
| 2004 | 65-69 years | 67.56 | 10.48 | 47.01 | 88.11 |  | 8.43 | 14.72 | -20.43 | 37.29 |  | 4.12 | 15.75 | -26.74 | 34.98 |  | 102.75 | 9.88 | 83.38 | 122.11 |
| 2005 | 65-69 years | 67.87 | 10.48 | 47.33 | 88.41 |  | 8.45 | 14.70 | -20.37 | 37.26 |  | 4.11 | 16.03 | -27.30 | 35.53 |  | 102.69 | 9.88 | 83.33 | 122.06 |
| 2006 | 65-69 years | 67.90 | 10.48 | 47.36 | 88.43 |  | 8.44 | 14.72 | -20.41 | 37.29 |  | 4.03 | 15.34 | -26.04 | 34.10 |  | 100.18 | 9.92 | 80.74 | 119.61 |
| 2007 | 65-69 years | 67.54 | 10.48 | 46.99 | 88.09 |  | 8.38 | 14.62 | -20.28 | 37.04 |  | 3.95 | 16.27 | -27.93 | 35.83 |  | 98.69 | 9.94 | 79.21 | 118.17 |
| 2008 | 65-69 years | 66.78 | 10.50 | 46.21 | 87.36 |  | 8.28 | 14.53 | -20.19 | 36.76 |  | 3.81 | 16.08 | -27.71 | 35.33 |  | 94.96 | 9.99 | 75.38 | 114.55 |
| 2009 | 65-69 years | 65.83 | 10.52 | 45.22 | 86.45 |  | 8.16 | 14.50 | -20.27 | 36.59 |  | 3.64 | 16.26 | -28.23 | 35.51 |  | 90.71 | 10.06 | 70.99 | 110.42 |
| 2010 | 65-69 years | 64.58 | 10.54 | 43.92 | 85.25 |  | 8.02 | 14.35 | -20.10 | 36.14 |  | 3.49 | 17.10 | -30.03 | 37.01 |  | 87.31 | 10.11 | 67.49 | 107.13 |
| 2011 | 65-69 years | 63.25 | 10.57 | 42.53 | 83.97 |  | 7.87 | 13.86 | -19.30 | 35.04 |  | 3.34 | 16.57 | -29.14 | 35.83 |  | 83.47 | 10.18 | 63.52 | 103.42 |
| 2012 | 65-69 years | 63.45 | 10.57 | 42.74 | 84.16 |  | 7.85 | 13.98 | -19.54 | 35.24 |  | 3.25 | 16.11 | -28.33 | 34.83 |  | 81.42 | 10.21 | 61.40 | 101.43 |
| 2013 | 65-69 years | 63.73 | 10.56 | 43.03 | 84.43 |  | 7.84 | 13.89 | -19.39 | 35.08 |  | 3.22 | 16.01 | -28.17 | 34.60 |  | 80.72 | 10.23 | 60.68 | 100.76 |
| 2014 | 65-69 years | 63.54 | 10.57 | 42.83 | 84.24 |  | 7.80 | 13.45 | -18.56 | 34.17 |  | 3.18 | 14.55 | -25.34 | 31.71 |  | 79.26 | 10.25 | 59.17 | 99.35 |
| 2015 | 65-69 years | 64.13 | 10.55 | 43.45 | 84.82 |  | 7.83 | 13.60 | -18.82 | 34.49 |  | 3.22 | 15.47 | -27.11 | 33.55 |  | 80.52 | 10.23 | 60.47 | 100.57 |
| 2016 | 65-69 years | 63.82 | 10.56 | 43.12 | 84.52 |  | 7.78 | 13.48 | -18.64 | 34.20 |  | 3.24 | 16.34 | -28.80 | 35.28 |  | 81.25 | 10.22 | 61.22 | 101.28 |
| 2017 | 65-69 years | 62.51 | 10.59 | 41.75 | 83.26 |  | 7.65 | 13.36 | -18.54 | 33.83 |  | 3.18 | 16.02 | -28.22 | 34.58 |  | 79.53 | 10.25 | 59.44 | 99.62 |
| 2018 | 65-69 years | 61.12 | 10.62 | 40.30 | 81.94 |  | 7.49 | 13.34 | -18.66 | 33.65 |  | 3.14 | 16.65 | -29.50 | 35.78 |  | 78.70 | 10.26 | 58.59 | 98.82 |
| 2019 | 65-69 years | 60.24 | 10.64 | 39.38 | 81.10 |  | 7.38 | 13.48 | -19.04 | 33.80 |  | 3.08 | 17.20 | -30.63 | 36.80 |  | 77.52 | 10.29 | 57.37 | 97.68 |
| 2020 | 65-69 years | 59.29 | 10.67 | 38.39 | 80.20 |  | 7.28 | 13.57 | -19.31 | 33.87 |  | 2.98 | 16.18 | -28.72 | 34.69 |  | 74.59 | 10.34 | 54.32 | 94.85 |
| 2021 | 65-69 years | 58.39 | 10.69 | 37.44 | 79.33 |  | 7.19 | 13.56 | -19.39 | 33.78 |  | 2.94 | 16.39 | -29.18 | 35.06 |  | 73.71 | 10.36 | 53.41 | 94.01 |
| 2022 | 65-69 years | 59.73 | 13.13 | 33.98 | 85.47 |  | 7.27 | 22.77 | -37.37 | 51.91 |  | 2.92 | 25.90 | -47.84 | 53.67 |  | 75.85 | 12.63 | 51.10 | 100.61 |
| 2023 | 65-69 years | 59.72 | 13.01 | 34.21 | 85.22 |  | 7.28 | 23.52 | -38.83 | 53.38 |  | 2.92 | 32.92 | -61.61 | 67.44 |  | 75.39 | 12.63 | 50.63 | 100.14 |
| 2024 | 65-69 years | 59.78 | 13.09 | 34.12 | 85.44 |  | 7.29 | 26.24 | -44.13 | 58.72 |  | 2.92 | 42.12 | -79.64 | 85.48 |  | 74.89 | 12.69 | 50.01 | 99.76 |
| 2025 | 65-69 years | 59.75 | 13.39 | 33.51 | 86.00 |  | 7.30 | 30.20 | -51.91 | 66.50 |  | 2.91 | 51.74 | -98.50 | 104.32 |  | 74.14 | 12.85 | 48.96 | 99.33 |
| 2026 | 65-69 years | 59.52 | 13.88 | 32.32 | 86.72 |  | 7.27 | 34.77 | -60.89 | 75.42 |  | 2.87 | 60.32 | -115.34 | 121.09 |  | 73.03 | 13.12 | 47.31 | 98.75 |
| 2027 | 65-69 years | 58.95 | 14.61 | 30.32 | 87.59 |  | 7.19 | 39.48 | -70.20 | 84.58 |  | 2.81 | 66.66 | -127.84 | 133.47 |  | 71.48 | 13.54 | 44.95 | 98.01 |
| 2028 | 65-69 years | 58.04 | 15.65 | 27.37 | 88.71 |  | 7.07 | 44.58 | -80.31 | 94.45 |  | 2.73 | 72.25 | -138.88 | 144.34 |  | 69.51 | 14.10 | 41.87 | 97.16 |
| 2029 | 65-69 years | 56.87 | 16.94 | 23.67 | 90.07 |  | 6.92 | 49.82 | -90.72 | 104.56 |  | 2.63 | 77.97 | -150.19 | 155.46 |  | 67.23 | 14.86 | 38.11 | 96.35 |
| 2030 | 65-69 years | 55.62 | 18.40 | 19.56 | 91.68 |  | 6.75 | 54.88 | -100.81 | 114.31 |  | 2.53 | 83.63 | -161.38 | 166.44 |  | 64.81 | 15.80 | 33.85 | 95.77 |
| 2031 | 65-69 years | 54.47 | 19.90 | 15.46 | 93.48 |  | 6.60 | 59.42 | -109.87 | 123.07 |  | 2.43 | 88.04 | -170.13 | 174.99 |  | 62.52 | 16.85 | 29.49 | 95.55 |
| 2032 | 65-69 years | 53.55 | 21.45 | 11.52 | 95.58 |  | 6.48 | 63.29 | -117.58 | 130.54 |  | 2.34 | 90.71 | -175.44 | 180.13 |  | 60.52 | 18.02 | 25.20 | 95.83 |
| 2033 | 65-69 years | 52.89 | 23.14 | 7.54 | 98.24 |  | 6.39 | 67.26 | -125.43 | 138.22 |  | 2.28 | 93.43 | -180.84 | 185.40 |  | 58.87 | 19.33 | 20.99 | 96.75 |
| 2034 | 65-69 years | 52.48 | 24.98 | 3.51 | 101.45 |  | 6.34 | 71.33 | -133.46 | 146.14 |  | 2.23 | 96.63 | -187.17 | 191.62 |  | 57.58 | 20.82 | 16.77 | 98.38 |
| 2035 | 65-69 years | 52.31 | 26.91 | -0.44 | 105.05 |  | 6.32 | 75.23 | -141.13 | 153.76 |  | 2.19 | 99.83 | -193.48 | 197.86 |  | 56.63 | 22.51 | 12.51 | 100.75 |
| 1990 | 70-74 years | 110.97 | 9.77 | 91.82 | 130.12 |  | 13.35 | 13.42 | -12.95 | 39.64 |  | 8.31 | 13.80 | -18.75 | 35.36 |  | 168.99 | 9.17 | 151.01 | 186.96 |
| 1991 | 70-74 years | 109.89 | 9.78 | 90.73 | 129.06 |  | 13.47 | 13.26 | -12.53 | 39.46 |  | 8.33 | 14.09 | -19.28 | 35.94 |  | 170.04 | 9.16 | 152.08 | 187.99 |
| 1992 | 70-74 years | 109.65 | 9.78 | 90.47 | 128.82 |  | 13.67 | 13.17 | -12.14 | 39.47 |  | 8.46 | 14.09 | -19.15 | 36.08 |  | 172.85 | 9.14 | 154.94 | 190.76 |
| 1993 | 70-74 years | 109.63 | 9.78 | 90.46 | 128.80 |  | 13.85 | 13.12 | -11.86 | 39.56 |  | 8.61 | 13.76 | -18.36 | 35.59 |  | 175.59 | 9.12 | 157.72 | 193.46 |
| 1994 | 70-74 years | 109.51 | 9.78 | 90.34 | 128.69 |  | 13.97 | 13.10 | -11.71 | 39.65 |  | 8.71 | 13.84 | -18.42 | 35.84 |  | 177.56 | 9.10 | 159.72 | 195.40 |
| 1995 | 70-74 years | 109.55 | 9.78 | 90.37 | 128.72 |  | 14.02 | 13.16 | -11.77 | 39.81 |  | 8.66 | 13.75 | -18.29 | 35.61 |  | 176.44 | 9.11 | 158.59 | 194.30 |
| 1996 | 70-74 years | 109.62 | 9.78 | 90.44 | 128.79 |  | 14.02 | 13.21 | -11.88 | 39.91 |  | 8.56 | 13.71 | -18.30 | 35.43 |  | 174.41 | 9.13 | 156.52 | 192.30 |
| 1997 | 70-74 years | 109.90 | 9.78 | 90.73 | 129.07 |  | 14.00 | 13.33 | -12.14 | 40.13 |  | 8.47 | 13.70 | -18.37 | 35.32 |  | 172.63 | 9.14 | 154.71 | 190.55 |
| 1998 | 70-74 years | 110.24 | 9.78 | 91.08 | 129.41 |  | 13.98 | 13.49 | -12.46 | 40.42 |  | 8.43 | 13.72 | -18.46 | 35.33 |  | 171.90 | 9.15 | 153.97 | 189.83 |
| 1999 | 70-74 years | 110.14 | 9.78 | 90.97 | 129.31 |  | 13.91 | 13.54 | -12.62 | 40.44 |  | 8.40 | 13.59 | -18.25 | 35.04 |  | 170.97 | 9.16 | 153.02 | 188.91 |
| 2000 | 70-74 years | 109.54 | 9.79 | 90.35 | 128.73 |  | 13.81 | 13.50 | -12.66 | 40.27 |  | 8.42 | 14.22 | -19.46 | 36.30 |  | 171.95 | 9.15 | 154.02 | 189.88 |
| 2001 | 70-74 years | 108.64 | 9.80 | 89.42 | 127.85 |  | 13.68 | 13.51 | -12.79 | 40.16 |  | 8.36 | 14.43 | -19.92 | 36.63 |  | 170.75 | 9.16 | 152.80 | 188.69 |
| 2002 | 70-74 years | 107.18 | 9.82 | 87.93 | 126.43 |  | 13.52 | 13.48 | -12.89 | 39.93 |  | 8.29 | 14.74 | -20.59 | 37.18 |  | 169.67 | 9.17 | 151.70 | 187.63 |
| 2003 | 70-74 years | 105.59 | 9.84 | 86.30 | 124.89 |  | 13.34 | 13.49 | -13.10 | 39.78 |  | 8.12 | 14.57 | -20.44 | 36.67 |  | 165.86 | 9.20 | 147.83 | 183.89 |
| 2004 | 70-74 years | 103.93 | 9.87 | 84.59 | 123.27 |  | 13.16 | 13.50 | -13.31 | 39.63 |  | 7.84 | 14.33 | -20.26 | 35.93 |  | 159.97 | 9.25 | 141.84 | 178.10 |
| 2005 | 70-74 years | 102.80 | 9.88 | 83.43 | 122.16 |  | 13.02 | 13.57 | -13.57 | 39.62 |  | 7.61 | 14.72 | -21.25 | 36.47 |  | 155.84 | 9.29 | 137.63 | 174.04 |
| 2006 | 70-74 years | 102.11 | 9.89 | 82.73 | 121.50 |  | 12.92 | 13.53 | -13.61 | 39.45 |  | 7.35 | 14.33 | -20.74 | 35.45 |  | 150.44 | 9.34 | 132.13 | 168.74 |
| 2007 | 70-74 years | 102.07 | 9.89 | 82.68 | 121.45 |  | 12.89 | 13.47 | -13.52 | 39.30 |  | 7.27 | 14.94 | -22.02 | 36.55 |  | 149.39 | 9.35 | 131.07 | 167.71 |
| 2008 | 70-74 years | 102.42 | 9.89 | 83.05 | 121.80 |  | 12.91 | 13.39 | -13.34 | 39.15 |  | 7.23 | 15.09 | -22.34 | 36.80 |  | 148.83 | 9.35 | 130.50 | 167.16 |
| 2009 | 70-74 years | 102.92 | 9.88 | 83.56 | 122.29 |  | 12.93 | 13.34 | -13.21 | 39.07 |  | 7.17 | 14.99 | -22.20 | 36.54 |  | 147.50 | 9.37 | 129.14 | 165.85 |
| 2010 | 70-74 years | 103.02 | 9.88 | 83.65 | 122.38 |  | 12.89 | 13.25 | -13.08 | 38.86 |  | 7.12 | 15.57 | -23.39 | 37.63 |  | 146.84 | 9.37 | 128.47 | 165.21 |
| 2011 | 70-74 years | 102.61 | 9.88 | 83.24 | 121.98 |  | 12.79 | 13.22 | -13.13 | 38.70 |  | 6.96 | 15.46 | -23.34 | 37.26 |  | 143.35 | 9.41 | 124.91 | 161.78 |
| 2012 | 70-74 years | 101.42 | 9.90 | 82.01 | 120.82 |  | 12.58 | 13.10 | -13.09 | 38.25 |  | 6.76 | 15.27 | -23.17 | 36.70 |  | 138.94 | 9.45 | 120.41 | 157.46 |
| 2013 | 70-74 years | 99.72 | 9.92 | 80.28 | 119.17 |  | 12.32 | 13.03 | -13.22 | 37.86 |  | 6.51 | 15.03 | -22.95 | 35.97 |  | 133.31 | 9.51 | 114.67 | 151.95 |
| 2014 | 70-74 years | 97.89 | 9.95 | 78.39 | 117.39 |  | 12.06 | 13.04 | -13.51 | 37.62 |  | 6.20 | 14.31 | -21.84 | 34.24 |  | 126.37 | 9.59 | 107.59 | 145.16 |
| 2015 | 70-74 years | 95.91 | 9.98 | 76.35 | 115.46 |  | 11.82 | 13.01 | -13.68 | 37.32 |  | 5.94 | 14.15 | -21.79 | 33.68 |  | 121.01 | 9.65 | 102.10 | 139.92 |
| 2016 | 70-74 years | 93.96 | 10.01 | 74.35 | 113.57 |  | 11.63 | 12.84 | -13.54 | 36.80 |  | 5.75 | 14.21 | -22.10 | 33.60 |  | 117.34 | 9.69 | 98.35 | 136.34 |
| 2017 | 70-74 years | 94.35 | 10.00 | 74.75 | 113.95 |  | 11.68 | 13.00 | -13.80 | 37.16 |  | 5.61 | 14.33 | -22.49 | 33.70 |  | 115.00 | 9.72 | 95.95 | 134.06 |
| 2018 | 70-74 years | 95.02 | 9.99 | 75.44 | 114.60 |  | 11.77 | 13.05 | -13.81 | 37.36 |  | 5.58 | 14.97 | -23.75 | 34.91 |  | 115.12 | 9.72 | 96.07 | 134.17 |
| 2019 | 70-74 years | 95.11 | 9.99 | 75.53 | 114.69 |  | 11.79 | 12.83 | -13.36 | 36.95 |  | 5.54 | 14.62 | -23.12 | 34.19 |  | 114.05 | 9.73 | 94.97 | 133.12 |
| 2020 | 70-74 years | 96.28 | 9.97 | 76.74 | 115.83 |  | 11.90 | 12.89 | -13.36 | 37.15 |  | 5.55 | 14.89 | -23.63 | 34.72 |  | 114.45 | 9.73 | 95.39 | 133.52 |
| 2021 | 70-74 years | 95.24 | 9.99 | 75.66 | 114.82 |  | 11.84 | 12.87 | -13.39 | 37.07 |  | 5.57 | 15.17 | -24.17 | 35.31 |  | 114.96 | 9.72 | 95.90 | 134.01 |
| 2022 | 70-74 years | 96.35 | 11.90 | 73.03 | 119.67 |  | 11.85 | 21.22 | -29.74 | 53.44 |  | 5.51 | 24.94 | -43.38 | 54.39 |  | 116.06 | 12.97 | 90.65 | 141.48 |
| 2023 | 70-74 years | 94.95 | 12.16 | 71.13 | 118.78 |  | 11.66 | 22.66 | -32.76 | 56.08 |  | 5.43 | 32.11 | -57.50 | 68.37 |  | 114.97 | 13.06 | 89.38 | 140.56 |
| 2024 | 70-74 years | 93.43 | 12.56 | 68.82 | 118.04 |  | 11.46 | 25.59 | -38.69 | 61.62 |  | 5.35 | 41.05 | -75.12 | 85.81 |  | 113.62 | 13.23 | 87.70 | 139.54 |
| 2025 | 70-74 years | 92.09 | 13.07 | 66.47 | 117.70 |  | 11.29 | 29.50 | -46.52 | 69.11 |  | 5.25 | 50.31 | -93.36 | 103.86 |  | 112.22 | 13.47 | 85.81 | 138.62 |
| 2026 | 70-74 years | 91.23 | 13.61 | 64.55 | 117.91 |  | 11.19 | 33.81 | -55.07 | 77.45 |  | 5.18 | 58.42 | -109.33 | 119.69 |  | 111.02 | 13.78 | 84.02 | 138.02 |
| 2027 | 70-74 years | 90.87 | 14.23 | 62.99 | 118.76 |  | 11.16 | 38.12 | -63.56 | 85.87 |  | 5.14 | 64.38 | -121.03 | 131.32 |  | 110.13 | 14.13 | 82.44 | 137.82 |
| 2028 | 70-74 years | 90.86 | 15.01 | 61.44 | 120.28 |  | 11.17 | 42.76 | -72.64 | 94.97 |  | 5.14 | 69.61 | -131.30 | 141.58 |  | 109.45 | 14.56 | 80.92 | 137.99 |
| 2029 | 70-74 years | 90.96 | 15.98 | 59.63 | 122.28 |  | 11.19 | 47.56 | -82.03 | 104.42 |  | 5.15 | 74.84 | -141.54 | 151.83 |  | 108.73 | 15.09 | 79.15 | 138.30 |
| 2030 | 70-74 years | 90.92 | 17.13 | 57.34 | 124.49 |  | 11.20 | 52.27 | -91.26 | 113.66 |  | 5.13 | 80.03 | -151.73 | 161.99 |  | 107.65 | 15.76 | 76.77 | 138.53 |
| 2031 | 70-74 years | 90.56 | 18.38 | 54.52 | 126.59 |  | 11.16 | 56.54 | -99.66 | 121.97 |  | 5.07 | 84.36 | -160.27 | 170.41 |  | 106.03 | 16.56 | 73.58 | 138.48 |
| 2032 | 70-74 years | 89.70 | 19.79 | 50.91 | 128.49 |  | 11.04 | 60.25 | -107.06 | 129.14 |  | 4.96 | 87.03 | -165.63 | 175.55 |  | 103.78 | 17.52 | 69.45 | 138.11 |
| 2033 | 70-74 years | 88.31 | 21.46 | 46.24 | 130.37 |  | 10.85 | 64.13 | -114.84 | 136.54 |  | 4.82 | 89.50 | -170.61 | 180.24 |  | 100.93 | 18.67 | 64.34 | 137.51 |
| 2034 | 70-74 years | 86.53 | 23.33 | 40.80 | 132.26 |  | 10.62 | 68.03 | -122.73 | 143.96 |  | 4.65 | 92.48 | -176.62 | 185.91 |  | 97.61 | 20.04 | 58.33 | 136.89 |
| 2035 | 70-74 years | 84.63 | 25.27 | 35.10 | 134.16 |  | 10.37 | 71.71 | -130.19 | 150.92 |  | 4.47 | 95.71 | -183.13 | 192.06 |  | 94.10 | 21.64 | 51.70 | 136.51 |
| 1990 | 75-79 years | 157.62 | 9.28 | 139.44 | 175.80 |  | 20.14 | 13.07 | -5.48 | 45.76 |  | 17.02 | 14.07 | -10.55 | 44.59 |  | 278.80 | 8.47 | 262.20 | 295.40 |
| 1991 | 75-79 years | 154.89 | 9.30 | 136.66 | 173.11 |  | 20.00 | 13.14 | -5.75 | 45.75 |  | 16.72 | 14.05 | -10.82 | 44.26 |  | 273.05 | 8.50 | 256.39 | 289.71 |
| 1992 | 75-79 years | 151.85 | 9.32 | 133.58 | 170.12 |  | 19.75 | 13.02 | -5.77 | 45.28 |  | 16.31 | 13.97 | -11.08 | 43.70 |  | 265.57 | 8.54 | 248.83 | 282.30 |
| 1993 | 75-79 years | 148.74 | 9.35 | 130.42 | 167.07 |  | 19.47 | 12.68 | -5.38 | 44.32 |  | 15.82 | 13.67 | -10.98 | 42.62 |  | 256.71 | 8.59 | 239.88 | 273.54 |
| 1994 | 75-79 years | 146.90 | 9.36 | 128.54 | 165.25 |  | 19.32 | 12.40 | -4.99 | 43.62 |  | 15.35 | 13.31 | -10.74 | 41.44 |  | 249.01 | 8.63 | 232.09 | 265.92 |
| 1995 | 75-79 years | 146.07 | 9.37 | 127.70 | 164.44 |  | 19.37 | 12.29 | -4.73 | 43.47 |  | 15.02 | 13.10 | -10.66 | 40.71 |  | 244.30 | 8.65 | 227.34 | 261.27 |
| 1996 | 75-79 years | 146.85 | 9.37 | 128.49 | 165.21 |  | 19.61 | 12.40 | -4.69 | 43.91 |  | 14.89 | 12.84 | -10.27 | 40.06 |  | 242.57 | 8.66 | 225.59 | 259.55 |
| 1997 | 75-79 years | 148.87 | 9.35 | 130.55 | 167.19 |  | 19.95 | 12.59 | -4.73 | 44.63 |  | 14.91 | 12.53 | -9.65 | 39.46 |  | 242.71 | 8.66 | 225.73 | 259.69 |
| 1998 | 75-79 years | 150.98 | 9.33 | 132.69 | 169.27 |  | 20.25 | 12.76 | -4.75 | 45.25 |  | 15.11 | 12.73 | -9.84 | 40.05 |  | 246.18 | 8.64 | 229.24 | 263.12 |
| 1999 | 75-79 years | 152.41 | 9.32 | 134.14 | 170.67 |  | 20.43 | 12.84 | -4.74 | 45.59 |  | 15.19 | 12.43 | -9.18 | 39.57 |  | 247.11 | 8.64 | 230.18 | 264.04 |
| 2000 | 75-79 years | 153.18 | 9.31 | 134.93 | 171.43 |  | 20.47 | 12.88 | -4.77 | 45.71 |  | 15.40 | 13.20 | -10.47 | 41.27 |  | 251.63 | 8.61 | 234.75 | 268.50 |
| 2001 | 75-79 years | 153.21 | 9.31 | 134.96 | 171.46 |  | 20.41 | 12.83 | -4.73 | 45.56 |  | 15.40 | 13.37 | -10.80 | 41.61 |  | 251.92 | 8.61 | 235.05 | 268.80 |
| 2002 | 75-79 years | 153.15 | 9.31 | 134.89 | 171.40 |  | 20.34 | 12.83 | -4.81 | 45.50 |  | 15.42 | 13.45 | -10.93 | 41.78 |  | 252.47 | 8.61 | 235.60 | 269.34 |
| 2003 | 75-79 years | 152.97 | 9.32 | 134.71 | 171.22 |  | 20.26 | 12.85 | -4.92 | 45.45 |  | 15.36 | 13.25 | -10.62 | 41.34 |  | 251.07 | 8.61 | 234.19 | 267.95 |
| 2004 | 75-79 years | 152.29 | 9.32 | 134.01 | 170.56 |  | 20.14 | 12.81 | -4.98 | 45.25 |  | 15.18 | 13.24 | -10.77 | 41.13 |  | 248.05 | 8.63 | 231.14 | 264.97 |
| 2005 | 75-79 years | 150.99 | 9.33 | 132.69 | 169.29 |  | 19.96 | 12.73 | -4.99 | 44.91 |  | 15.02 | 13.59 | -11.62 | 41.66 |  | 245.77 | 8.64 | 228.83 | 262.71 |
| 2006 | 75-79 years | 149.18 | 9.35 | 130.85 | 167.51 |  | 19.75 | 12.67 | -5.08 | 44.58 |  | 14.67 | 13.55 | -11.88 | 41.22 |  | 239.84 | 8.68 | 222.83 | 256.84 |
| 2007 | 75-79 years | 146.36 | 9.38 | 127.98 | 164.74 |  | 19.46 | 12.54 | -5.11 | 44.03 |  | 14.30 | 13.37 | -11.90 | 40.50 |  | 233.38 | 8.72 | 216.30 | 250.47 |
| 2008 | 75-79 years | 143.38 | 9.41 | 124.94 | 161.81 |  | 19.14 | 12.45 | -5.25 | 43.54 |  | 13.93 | 13.53 | -12.60 | 40.46 |  | 227.43 | 8.75 | 210.27 | 244.58 |
| 2009 | 75-79 years | 140.36 | 9.43 | 121.87 | 158.86 |  | 18.82 | 12.39 | -5.46 | 43.10 |  | 13.36 | 13.36 | -12.83 | 39.55 |  | 217.90 | 8.81 | 200.63 | 235.17 |
| 2010 | 75-79 years | 138.46 | 9.45 | 119.93 | 156.99 |  | 18.55 | 12.45 | -5.85 | 42.96 |  | 12.87 | 13.62 | -13.83 | 39.56 |  | 210.30 | 8.86 | 192.93 | 227.67 |
| 2011 | 75-79 years | 137.51 | 9.46 | 118.96 | 156.06 |  | 18.33 | 12.49 | -6.16 | 42.81 |  | 12.41 | 13.58 | -14.22 | 39.03 |  | 203.00 | 8.91 | 185.53 | 220.46 |
| 2012 | 75-79 years | 137.47 | 9.47 | 118.91 | 156.02 |  | 18.16 | 12.54 | -6.41 | 42.74 |  | 12.13 | 13.34 | -14.02 | 38.28 |  | 198.38 | 8.94 | 180.85 | 215.91 |
| 2013 | 75-79 years | 137.95 | 9.46 | 119.41 | 156.50 |  | 18.05 | 12.55 | -6.55 | 42.65 |  | 12.02 | 13.26 | -13.96 | 38.01 |  | 196.70 | 8.96 | 179.15 | 214.26 |
| 2014 | 75-79 years | 138.63 | 9.45 | 120.10 | 157.16 |  | 17.97 | 12.58 | -6.69 | 42.63 |  | 11.99 | 13.29 | -14.06 | 38.04 |  | 196.06 | 8.96 | 178.49 | 213.62 |
| 2015 | 75-79 years | 138.67 | 9.45 | 120.14 | 157.20 |  | 17.87 | 12.56 | -6.75 | 42.49 |  | 11.90 | 13.19 | -13.94 | 37.75 |  | 194.23 | 8.97 | 176.64 | 211.82 |
| 2016 | 75-79 years | 137.63 | 9.46 | 119.07 | 156.18 |  | 17.76 | 12.59 | -6.91 | 42.43 |  | 11.73 | 13.12 | -13.99 | 37.44 |  | 191.06 | 9.00 | 173.42 | 208.69 |
| 2017 | 75-79 years | 135.28 | 9.49 | 116.69 | 153.88 |  | 17.56 | 12.50 | -6.95 | 42.07 |  | 11.38 | 13.09 | -14.27 | 37.04 |  | 185.15 | 9.04 | 167.43 | 202.88 |
| 2018 | 75-79 years | 132.43 | 9.52 | 113.77 | 151.08 |  | 17.32 | 12.48 | -7.13 | 41.78 |  | 10.97 | 13.02 | -14.55 | 36.49 |  | 177.99 | 9.10 | 160.16 | 195.83 |
| 2019 | 75-79 years | 129.99 | 9.54 | 111.29 | 148.70 |  | 17.09 | 12.55 | -7.51 | 41.68 |  | 10.50 | 13.18 | -15.33 | 36.33 |  | 170.43 | 9.16 | 152.48 | 188.39 |
| 2020 | 75-79 years | 128.25 | 9.56 | 109.51 | 146.99 |  | 16.85 | 12.59 | -7.82 | 41.53 |  | 10.02 | 13.21 | -15.88 | 35.92 |  | 162.90 | 9.22 | 144.83 | 180.98 |
| 2021 | 75-79 years | 125.11 | 9.60 | 106.30 | 143.91 |  | 16.63 | 12.49 | -7.85 | 41.10 |  | 9.70 | 13.37 | -16.49 | 35.90 |  | 158.40 | 9.26 | 140.24 | 176.56 |
| 2022 | 75-79 years | 130.34 | 12.07 | 106.68 | 153.99 |  | 16.84 | 21.27 | -24.85 | 58.54 |  | 9.63 | 24.72 | -38.82 | 58.07 |  | 165.63 | 14.74 | 136.74 | 194.51 |
| 2023 | 75-79 years | 131.17 | 11.91 | 107.82 | 154.51 |  | 16.97 | 21.81 | -25.77 | 59.71 |  | 9.56 | 31.03 | -51.25 | 70.38 |  | 164.09 | 14.73 | 135.22 | 192.96 |
| 2024 | 75-79 years | 132.06 | 11.93 | 108.67 | 155.45 |  | 17.10 | 24.30 | -30.53 | 64.73 |  | 9.57 | 39.55 | -67.94 | 87.08 |  | 163.31 | 14.79 | 134.32 | 192.29 |
| 2025 | 75-79 years | 132.63 | 12.13 | 108.86 | 156.39 |  | 17.20 | 27.81 | -37.30 | 71.70 |  | 9.62 | 48.17 | -84.79 | 104.04 |  | 162.87 | 14.92 | 133.62 | 192.11 |
| 2026 | 75-79 years | 132.38 | 12.58 | 107.73 | 157.04 |  | 17.17 | 31.99 | -45.53 | 79.88 |  | 9.64 | 55.89 | -99.91 | 119.19 |  | 162.27 | 15.14 | 132.60 | 191.95 |
| 2027 | 75-79 years | 131.18 | 13.35 | 105.02 | 157.33 |  | 17.00 | 36.48 | -54.49 | 88.50 |  | 9.56 | 61.79 | -111.54 | 130.66 |  | 161.24 | 15.45 | 130.96 | 191.51 |
| 2028 | 75-79 years | 129.28 | 14.36 | 101.14 | 157.41 |  | 16.73 | 41.16 | -63.94 | 97.41 |  | 9.44 | 66.72 | -121.34 | 140.21 |  | 159.71 | 15.85 | 128.65 | 190.78 |
| 2029 | 75-79 years | 127.20 | 15.49 | 96.84 | 157.57 |  | 16.45 | 45.77 | -73.25 | 106.15 |  | 9.28 | 71.48 | -130.82 | 149.38 |  | 157.85 | 16.36 | 125.79 | 189.91 |
| 2030 | 75-79 years | 125.37 | 16.72 | 92.61 | 158.14 |  | 16.21 | 50.23 | -82.24 | 114.66 |  | 9.12 | 76.27 | -140.37 | 158.61 |  | 155.90 | 16.99 | 122.60 | 189.19 |
| 2031 | 75-79 years | 124.21 | 17.97 | 88.99 | 159.42 |  | 16.06 | 54.33 | -90.42 | 122.54 |  | 8.99 | 80.28 | -148.35 | 166.34 |  | 154.23 | 17.71 | 119.51 | 188.95 |
| 2032 | 75-79 years | 123.72 | 19.27 | 85.96 | 161.49 |  | 16.01 | 57.88 | -97.43 | 129.45 |  | 8.93 | 82.79 | -153.33 | 171.19 |  | 153.00 | 18.55 | 116.65 | 189.35 |
| 2033 | 75-79 years | 123.71 | 20.71 | 83.11 | 164.30 |  | 16.02 | 61.50 | -104.52 | 136.57 |  | 8.93 | 85.02 | -157.71 | 175.56 |  | 152.06 | 19.50 | 113.85 | 190.28 |
| 2034 | 75-79 years | 123.84 | 22.28 | 80.17 | 167.51 |  | 16.06 | 65.15 | -111.63 | 143.75 |  | 8.94 | 87.59 | -162.75 | 180.62 |  | 151.05 | 20.59 | 110.70 | 191.41 |
| 2035 | 75-79 years | 123.78 | 23.94 | 76.85 | 170.72 |  | 16.07 | 68.60 | -118.39 | 150.53 |  | 8.91 | 90.44 | -168.36 | 186.18 |  | 149.56 | 21.83 | 106.76 | 192.35 |
| 1990 | 80-84 years | 189.43 | 9.02 | 171.75 | 207.11 |  | 24.05 | 12.60 | -0.66 | 48.75 |  | 29.58 | 13.19 | 3.74 | 55.43 |  | 379.66 | 8.07 | 363.85 | 395.46 |
| 1991 | 80-84 years | 188.96 | 9.02 | 171.28 | 206.63 |  | 24.37 | 12.48 | -0.09 | 48.82 |  | 29.55 | 13.01 | 4.06 | 55.05 |  | 378.22 | 8.07 | 362.41 | 394.04 |
| 1992 | 80-84 years | 188.50 | 9.02 | 170.83 | 206.18 |  | 24.66 | 12.25 | 0.64 | 48.68 |  | 29.44 | 12.62 | 4.71 | 54.18 |  | 375.38 | 8.08 | 359.55 | 391.21 |
| 1993 | 80-84 years | 188.36 | 9.02 | 170.68 | 206.03 |  | 24.91 | 12.13 | 1.13 | 48.69 |  | 29.30 | 12.17 | 5.45 | 53.15 |  | 372.08 | 8.09 | 356.23 | 387.93 |
| 1994 | 80-84 years | 188.12 | 9.02 | 170.44 | 205.80 |  | 25.07 | 12.11 | 1.33 | 48.82 |  | 29.15 | 11.97 | 5.68 | 52.61 |  | 369.30 | 8.10 | 353.43 | 385.17 |
| 1995 | 80-84 years | 187.72 | 9.02 | 170.04 | 205.40 |  | 25.09 | 12.12 | 1.33 | 48.85 |  | 28.85 | 11.90 | 5.51 | 52.18 |  | 364.80 | 8.11 | 348.89 | 380.70 |
| 1996 | 80-84 years | 187.60 | 9.02 | 169.92 | 205.29 |  | 24.96 | 12.27 | 0.91 | 49.00 |  | 28.26 | 12.10 | 4.54 | 51.98 |  | 357.09 | 8.14 | 341.13 | 373.04 |
| 1997 | 80-84 years | 188.22 | 9.02 | 170.54 | 205.90 |  | 24.72 | 12.47 | 0.28 | 49.16 |  | 27.30 | 12.14 | 3.51 | 51.08 |  | 344.24 | 8.19 | 328.19 | 360.30 |
| 1998 | 80-84 years | 189.16 | 9.02 | 171.48 | 206.83 |  | 24.45 | 12.54 | -0.13 | 49.04 |  | 26.36 | 12.26 | 2.33 | 50.39 |  | 332.57 | 8.24 | 316.42 | 348.71 |
| 1999 | 80-84 years | 190.70 | 9.01 | 173.04 | 208.36 |  | 24.35 | 12.68 | -0.50 | 49.20 |  | 25.50 | 11.73 | 2.51 | 48.48 |  | 321.47 | 8.28 | 305.23 | 337.70 |
| 2000 | 80-84 years | 191.73 | 9.00 | 174.08 | 209.37 |  | 24.47 | 12.89 | -0.79 | 49.74 |  | 25.24 | 11.72 | 2.27 | 48.21 |  | 319.69 | 8.29 | 303.44 | 335.94 |
| 2001 | 80-84 years | 192.47 | 9.00 | 174.83 | 210.11 |  | 24.78 | 13.02 | -0.75 | 50.30 |  | 25.24 | 11.59 | 2.52 | 47.96 |  | 320.33 | 8.29 | 304.09 | 336.57 |
| 2002 | 80-84 years | 193.52 | 8.99 | 175.90 | 211.14 |  | 25.12 | 12.99 | -0.33 | 50.57 |  | 25.68 | 11.72 | 2.70 | 48.65 |  | 326.76 | 8.26 | 310.58 | 342.95 |
| 2003 | 80-84 years | 194.21 | 8.98 | 176.60 | 211.82 |  | 25.39 | 12.85 | 0.21 | 50.56 |  | 26.09 | 11.90 | 2.78 | 49.41 |  | 332.23 | 8.23 | 316.09 | 348.37 |
| 2004 | 80-84 years | 194.35 | 8.98 | 176.74 | 211.95 |  | 25.52 | 12.70 | 0.63 | 50.41 |  | 26.26 | 12.28 | 2.20 | 50.32 |  | 334.87 | 8.22 | 318.76 | 350.98 |
| 2005 | 80-84 years | 194.13 | 8.98 | 176.52 | 211.73 |  | 25.52 | 12.60 | 0.83 | 50.22 |  | 26.15 | 12.54 | 1.58 | 50.72 |  | 334.02 | 8.22 | 317.90 | 350.14 |
| 2006 | 80-84 years | 192.89 | 8.99 | 175.27 | 210.51 |  | 25.37 | 12.36 | 1.15 | 49.60 |  | 25.78 | 12.61 | 1.05 | 50.50 |  | 329.26 | 8.24 | 313.10 | 345.41 |
| 2007 | 80-84 years | 191.19 | 9.00 | 173.55 | 208.83 |  | 25.18 | 12.14 | 1.39 | 48.96 |  | 25.41 | 12.49 | 0.94 | 49.89 |  | 324.38 | 8.26 | 308.18 | 340.57 |
| 2008 | 80-84 years | 189.31 | 9.01 | 171.65 | 206.98 |  | 24.96 | 11.93 | 1.57 | 48.35 |  | 25.18 | 12.56 | 0.56 | 49.80 |  | 321.44 | 8.27 | 305.22 | 337.66 |
| 2009 | 80-84 years | 187.20 | 9.03 | 169.51 | 204.90 |  | 24.70 | 11.80 | 1.58 | 47.83 |  | 24.74 | 12.56 | 0.12 | 49.37 |  | 315.63 | 8.30 | 299.36 | 331.89 |
| 2010 | 80-84 years | 185.10 | 9.04 | 167.38 | 202.83 |  | 24.41 | 11.77 | 1.34 | 47.48 |  | 24.11 | 12.42 | -0.23 | 48.45 |  | 307.15 | 8.34 | 290.81 | 323.48 |
| 2011 | 80-84 years | 183.25 | 9.06 | 165.50 | 201.01 |  | 24.09 | 11.86 | 0.85 | 47.33 |  | 23.48 | 12.48 | -0.99 | 47.95 |  | 298.99 | 8.37 | 282.58 | 315.39 |
| 2012 | 80-84 years | 180.49 | 9.08 | 162.69 | 198.28 |  | 23.64 | 11.97 | 0.18 | 47.11 |  | 22.89 | 12.54 | -1.69 | 47.48 |  | 291.23 | 8.41 | 274.75 | 307.71 |
| 2013 | 80-84 years | 177.67 | 9.10 | 159.83 | 195.51 |  | 23.18 | 12.19 | -0.71 | 47.07 |  | 22.34 | 12.82 | -2.78 | 47.46 |  | 284.35 | 8.44 | 267.80 | 300.89 |
| 2014 | 80-84 years | 174.68 | 9.13 | 156.79 | 192.57 |  | 22.73 | 12.40 | -1.57 | 47.03 |  | 21.59 | 12.89 | -3.68 | 46.86 |  | 274.86 | 8.49 | 258.22 | 291.49 |
| 2015 | 80-84 years | 172.70 | 9.14 | 154.78 | 190.62 |  | 22.40 | 12.65 | -2.40 | 47.20 |  | 20.85 | 12.90 | -4.44 | 46.13 |  | 265.50 | 8.54 | 248.77 | 282.23 |
| 2016 | 80-84 years | 170.90 | 9.16 | 152.95 | 188.85 |  | 22.17 | 12.75 | -2.83 | 47.16 |  | 20.19 | 12.68 | -4.67 | 45.04 |  | 257.20 | 8.58 | 240.38 | 274.01 |
| 2017 | 80-84 years | 169.27 | 9.17 | 151.30 | 187.25 |  | 22.06 | 12.74 | -2.92 | 47.04 |  | 19.67 | 12.40 | -4.63 | 43.97 |  | 250.49 | 8.62 | 233.60 | 267.38 |
| 2018 | 80-84 years | 168.07 | 9.18 | 150.08 | 186.06 |  | 22.03 | 12.65 | -2.77 | 46.82 |  | 19.45 | 12.19 | -4.45 | 43.35 |  | 247.60 | 8.63 | 230.67 | 264.52 |
| 2019 | 80-84 years | 167.84 | 9.18 | 149.85 | 185.83 |  | 22.04 | 12.59 | -2.63 | 46.71 |  | 19.37 | 12.33 | -4.80 | 43.55 |  | 246.64 | 8.64 | 229.71 | 263.57 |
| 2020 | 80-84 years | 168.07 | 9.18 | 150.08 | 186.06 |  | 22.02 | 12.55 | -2.58 | 46.62 |  | 19.12 | 12.27 | -4.94 | 43.17 |  | 243.25 | 8.66 | 226.28 | 260.22 |
| 2021 | 80-84 years | 165.57 | 9.20 | 147.54 | 183.60 |  | 21.78 | 12.31 | -2.35 | 45.92 |  | 18.87 | 12.33 | -5.30 | 43.03 |  | 239.88 | 8.68 | 222.87 | 256.89 |
| 2022 | 80-84 years | 167.95 | 11.40 | 145.60 | 190.29 |  | 21.71 | 19.45 | -16.40 | 59.83 |  | 18.65 | 22.65 | -25.76 | 63.05 |  | 242.05 | 17.94 | 206.90 | 277.21 |
| 2023 | 80-84 years | 166.55 | 11.52 | 143.97 | 189.13 |  | 21.48 | 20.65 | -18.99 | 61.95 |  | 18.08 | 29.21 | -39.17 | 75.34 |  | 236.07 | 17.81 | 201.17 | 270.97 |
| 2024 | 80-84 years | 164.81 | 11.86 | 141.56 | 188.05 |  | 21.21 | 23.54 | -24.93 | 67.34 |  | 17.41 | 37.43 | -55.95 | 90.77 |  | 229.51 | 17.70 | 194.81 | 264.21 |
| 2025 | 80-84 years | 163.18 | 12.38 | 138.92 | 187.45 |  | 20.94 | 27.44 | -32.84 | 74.72 |  | 16.76 | 45.77 | -72.95 | 106.46 |  | 223.27 | 17.67 | 188.63 | 257.90 |
| 2026 | 80-84 years | 162.31 | 12.94 | 136.95 | 187.67 |  | 20.79 | 31.53 | -41.01 | 82.60 |  | 16.25 | 53.06 | -87.75 | 120.26 |  | 218.22 | 17.73 | 183.47 | 252.96 |
| 2027 | 80-84 years | 162.64 | 13.48 | 136.21 | 189.06 |  | 20.84 | 35.56 | -48.85 | 90.53 |  | 15.96 | 58.49 | -98.67 | 130.60 |  | 214.78 | 17.89 | 179.73 | 249.84 |
| 2028 | 80-84 years | 163.67 | 14.15 | 135.94 | 191.41 |  | 20.99 | 39.88 | -57.18 | 99.16 |  | 15.86 | 63.17 | -107.96 | 139.68 |  | 212.80 | 18.16 | 177.21 | 248.39 |
| 2029 | 80-84 years | 164.79 | 14.98 | 135.43 | 194.15 |  | 21.15 | 44.40 | -65.87 | 108.18 |  | 15.86 | 67.93 | -117.28 | 149.00 |  | 211.78 | 18.55 | 175.43 | 248.13 |
| 2030 | 80-84 years | 165.49 | 15.93 | 134.28 | 196.71 |  | 21.28 | 48.68 | -74.13 | 116.68 |  | 15.96 | 72.40 | -125.96 | 157.87 |  | 211.21 | 19.04 | 173.88 | 248.54 |
| 2031 | 80-84 years | 165.19 | 17.06 | 131.75 | 198.62 |  | 21.25 | 52.62 | -81.89 | 124.38 |  | 15.98 | 76.22 | -133.40 | 165.36 |  | 210.44 | 19.65 | 171.94 | 248.95 |
| 2032 | 80-84 years | 163.68 | 18.45 | 127.53 | 199.84 |  | 21.03 | 56.20 | -89.12 | 131.19 |  | 15.86 | 78.73 | -138.46 | 170.17 |  | 209.10 | 20.35 | 169.21 | 248.99 |
| 2033 | 80-84 years | 161.32 | 20.03 | 122.06 | 200.58 |  | 20.70 | 59.77 | -96.46 | 137.86 |  | 15.65 | 80.82 | -142.76 | 174.05 |  | 207.13 | 21.18 | 165.62 | 248.64 |
| 2034 | 80-84 years | 158.73 | 21.67 | 116.25 | 201.20 |  | 20.35 | 63.21 | -103.55 | 144.25 |  | 15.40 | 83.10 | -147.48 | 178.27 |  | 204.71 | 22.14 | 161.31 | 248.11 |
| 2035 | 80-84 years | 156.45 | 23.32 | 110.73 | 202.16 |  | 20.05 | 66.49 | -110.27 | 150.37 |  | 15.12 | 85.70 | -152.85 | 183.10 |  | 202.18 | 23.26 | 156.60 | 247.76 |
| 1990 | 85-89 years | 195.86 | 9.00 | 178.22 | 213.49 |  | 24.66 | 12.86 | -0.54 | 49.86 |  | 48.71 | 11.93 | 25.33 | 72.09 |  | 492.50 | 7.83 | 477.16 | 507.84 |
| 1991 | 85-89 years | 195.06 | 8.99 | 177.44 | 212.68 |  | 25.04 | 12.93 | -0.29 | 50.38 |  | 48.37 | 11.81 | 25.22 | 71.52 |  | 487.90 | 7.83 | 472.55 | 503.25 |
| 1992 | 85-89 years | 195.26 | 8.98 | 177.65 | 212.86 |  | 25.47 | 12.85 | 0.27 | 50.66 |  | 48.15 | 11.54 | 25.54 | 70.76 |  | 484.15 | 7.83 | 468.80 | 499.50 |
| 1993 | 85-89 years | 196.06 | 8.97 | 178.47 | 213.64 |  | 25.93 | 12.84 | 0.76 | 51.10 |  | 48.12 | 11.24 | 26.09 | 70.15 |  | 482.24 | 7.83 | 466.89 | 497.59 |
| 1994 | 85-89 years | 197.04 | 8.96 | 179.47 | 214.61 |  | 26.38 | 12.83 | 1.23 | 51.53 |  | 48.25 | 11.08 | 26.53 | 69.97 |  | 482.86 | 7.82 | 467.53 | 498.19 |
| 1995 | 85-89 years | 198.40 | 8.95 | 180.85 | 215.94 |  | 26.83 | 12.87 | 1.59 | 52.06 |  | 48.54 | 11.32 | 26.35 | 70.73 |  | 486.60 | 7.81 | 471.30 | 501.90 |
| 1996 | 85-89 years | 201.07 | 8.94 | 183.55 | 218.58 |  | 27.25 | 12.96 | 1.85 | 52.65 |  | 48.38 | 11.42 | 25.99 | 70.77 |  | 485.46 | 7.81 | 470.16 | 500.76 |
| 1997 | 85-89 years | 205.14 | 8.92 | 187.66 | 222.62 |  | 27.67 | 13.28 | 1.65 | 53.69 |  | 47.76 | 11.20 | 25.81 | 69.71 |  | 478.28 | 7.82 | 462.95 | 493.61 |
| 1998 | 85-89 years | 209.68 | 8.90 | 192.23 | 227.13 |  | 28.03 | 13.67 | 1.24 | 54.83 |  | 47.42 | 11.28 | 25.32 | 69.52 |  | 475.17 | 7.82 | 459.84 | 490.50 |
| 1999 | 85-89 years | 213.11 | 8.89 | 195.69 | 230.54 |  | 28.25 | 13.97 | 0.86 | 55.63 |  | 47.38 | 11.52 | 24.79 | 69.97 |  | 475.31 | 7.82 | 459.99 | 490.64 |
| 2000 | 85-89 years | 214.11 | 8.89 | 196.69 | 231.53 |  | 28.20 | 13.90 | 0.96 | 55.44 |  | 47.56 | 11.86 | 24.32 | 70.80 |  | 477.97 | 7.81 | 462.67 | 493.28 |
| 2001 | 85-89 years | 212.82 | 8.89 | 195.39 | 230.25 |  | 27.91 | 13.62 | 1.22 | 54.60 |  | 46.78 | 11.62 | 24.01 | 69.55 |  | 468.51 | 7.83 | 453.16 | 483.86 |
| 2002 | 85-89 years | 210.44 | 8.90 | 192.99 | 227.90 |  | 27.48 | 13.21 | 1.59 | 53.37 |  | 45.76 | 11.85 | 22.54 | 68.99 |  | 458.31 | 7.86 | 442.90 | 473.72 |
| 2003 | 85-89 years | 207.67 | 8.92 | 190.19 | 225.14 |  | 27.05 | 12.66 | 2.24 | 51.85 |  | 44.27 | 11.90 | 20.95 | 67.59 |  | 443.27 | 7.90 | 427.79 | 458.76 |
| 2004 | 85-89 years | 206.45 | 8.92 | 188.97 | 223.94 |  | 26.84 | 12.40 | 2.52 | 51.15 |  | 42.78 | 12.03 | 19.20 | 66.35 |  | 429.64 | 7.94 | 414.08 | 445.20 |
| 2005 | 85-89 years | 206.70 | 8.92 | 189.23 | 224.18 |  | 26.93 | 12.51 | 2.42 | 51.45 |  | 41.88 | 12.15 | 18.07 | 65.70 |  | 422.45 | 7.96 | 406.86 | 438.05 |
| 2006 | 85-89 years | 207.58 | 8.91 | 190.12 | 225.04 |  | 27.24 | 12.66 | 2.42 | 52.06 |  | 41.59 | 12.45 | 17.19 | 65.98 |  | 421.34 | 7.95 | 405.75 | 436.93 |
| 2007 | 85-89 years | 208.32 | 8.90 | 190.87 | 225.76 |  | 27.56 | 12.51 | 3.05 | 52.08 |  | 41.61 | 12.27 | 17.56 | 65.65 |  | 421.51 | 7.95 | 405.94 | 437.09 |
| 2008 | 85-89 years | 208.49 | 8.89 | 191.06 | 225.93 |  | 27.80 | 12.22 | 3.85 | 51.74 |  | 41.93 | 12.35 | 17.73 | 66.12 |  | 424.84 | 7.93 | 409.29 | 440.38 |
| 2009 | 85-89 years | 208.05 | 8.89 | 190.62 | 225.48 |  | 27.86 | 11.96 | 4.42 | 51.30 |  | 41.70 | 12.28 | 17.64 | 65.76 |  | 421.99 | 7.94 | 406.44 | 437.55 |
| 2010 | 85-89 years | 207.35 | 8.89 | 189.92 | 224.78 |  | 27.77 | 11.85 | 4.55 | 51.00 |  | 40.78 | 11.79 | 17.68 | 63.88 |  | 411.13 | 7.97 | 395.51 | 426.74 |
| 2011 | 85-89 years | 205.78 | 8.90 | 188.33 | 223.23 |  | 27.49 | 11.65 | 4.65 | 50.33 |  | 39.96 | 11.77 | 16.89 | 63.04 |  | 402.62 | 7.99 | 386.96 | 418.29 |
| 2012 | 85-89 years | 204.18 | 8.91 | 186.71 | 221.65 |  | 27.15 | 11.68 | 4.26 | 50.04 |  | 39.32 | 11.70 | 16.38 | 62.25 |  | 395.81 | 8.01 | 380.11 | 411.52 |
| 2013 | 85-89 years | 202.53 | 8.92 | 185.04 | 220.02 |  | 26.79 | 11.81 | 3.65 | 49.93 |  | 38.99 | 11.88 | 15.72 | 62.27 |  | 392.82 | 8.02 | 377.10 | 408.53 |
| 2014 | 85-89 years | 200.88 | 8.94 | 183.36 | 218.39 |  | 26.47 | 12.10 | 2.76 | 50.18 |  | 38.53 | 11.97 | 15.07 | 61.99 |  | 388.24 | 8.03 | 372.49 | 403.98 |
| 2015 | 85-89 years | 198.76 | 8.95 | 181.22 | 216.31 |  | 26.16 | 12.37 | 1.92 | 50.39 |  | 37.81 | 11.89 | 14.49 | 61.12 |  | 380.45 | 8.06 | 364.66 | 396.25 |
| 2016 | 85-89 years | 195.77 | 8.97 | 178.19 | 213.35 |  | 25.86 | 12.66 | 1.06 | 50.67 |  | 37.07 | 11.91 | 13.74 | 60.41 |  | 372.80 | 8.08 | 356.96 | 388.65 |
| 2017 | 85-89 years | 191.18 | 9.00 | 173.54 | 208.82 |  | 25.50 | 12.80 | 0.41 | 50.59 |  | 35.81 | 11.45 | 13.37 | 58.26 |  | 358.67 | 8.14 | 342.73 | 374.62 |
| 2018 | 85-89 years | 186.71 | 9.03 | 169.00 | 204.41 |  | 25.15 | 13.01 | -0.34 | 50.64 |  | 34.66 | 11.27 | 12.57 | 56.75 |  | 346.42 | 8.18 | 330.39 | 362.46 |
| 2019 | 85-89 years | 182.41 | 9.06 | 164.65 | 200.18 |  | 24.79 | 13.16 | -1.00 | 50.57 |  | 33.29 | 11.21 | 11.33 | 55.25 |  | 332.21 | 8.24 | 316.06 | 348.35 |
| 2020 | 85-89 years | 179.58 | 9.09 | 161.77 | 197.38 |  | 24.51 | 13.38 | -1.72 | 50.73 |  | 32.07 | 11.36 | 9.79 | 54.34 |  | 320.91 | 8.28 | 304.68 | 337.15 |
| 2021 | 85-89 years | 175.98 | 9.11 | 158.13 | 193.84 |  | 24.08 | 12.86 | -1.13 | 49.28 |  | 31.12 | 11.35 | 8.87 | 53.36 |  | 311.60 | 8.32 | 295.28 | 327.91 |
| 2022 | 85-89 years | 181.01 | 11.64 | 158.19 | 203.83 |  | 24.00 | 19.44 | -14.10 | 62.10 |  | 31.21 | 21.77 | -11.46 | 73.88 |  | 327.71 | 22.59 | 283.43 | 371.99 |
| 2023 | 85-89 years | 181.30 | 11.57 | 158.63 | 203.97 |  | 24.02 | 20.15 | -15.46 | 63.51 |  | 31.09 | 27.51 | -22.82 | 85.00 |  | 324.67 | 22.58 | 280.42 | 368.92 |
| 2024 | 85-89 years | 181.72 | 11.67 | 158.86 | 204.59 |  | 24.07 | 22.57 | -20.17 | 68.30 |  | 31.06 | 34.87 | -37.28 | 99.40 |  | 321.98 | 22.60 | 277.68 | 366.28 |
| 2025 | 85-89 years | 181.85 | 11.97 | 158.39 | 205.31 |  | 24.06 | 26.20 | -27.29 | 75.41 |  | 30.91 | 42.62 | -52.61 | 114.44 |  | 318.62 | 22.63 | 274.27 | 362.97 |
| 2026 | 85-89 years | 181.53 | 12.42 | 157.18 | 205.88 |  | 23.98 | 30.29 | -35.38 | 83.35 |  | 30.56 | 49.76 | -66.96 | 128.08 |  | 314.00 | 22.64 | 269.63 | 358.37 |
| 2027 | 85-89 years | 180.64 | 13.03 | 155.09 | 206.18 |  | 23.82 | 34.41 | -43.62 | 91.27 |  | 29.96 | 54.89 | -77.64 | 137.55 |  | 307.83 | 22.62 | 263.50 | 352.16 |
| 2028 | 85-89 years | 179.13 | 13.86 | 151.96 | 206.30 |  | 23.57 | 38.79 | -52.46 | 99.60 |  | 29.05 | 59.22 | -87.03 | 145.13 |  | 300.22 | 22.58 | 255.95 | 344.48 |
| 2029 | 85-89 years | 177.26 | 14.91 | 148.04 | 206.49 |  | 23.27 | 43.31 | -61.62 | 108.16 |  | 27.97 | 63.63 | -96.75 | 152.70 |  | 291.88 | 22.59 | 247.61 | 336.16 |
| 2030 | 85-89 years | 175.51 | 16.10 | 143.95 | 207.07 |  | 22.98 | 47.68 | -70.47 | 116.43 |  | 26.92 | 68.03 | -106.42 | 160.27 |  | 283.94 | 22.71 | 239.43 | 328.45 |
| 2031 | 85-89 years | 174.57 | 17.30 | 140.66 | 208.48 |  | 22.82 | 51.51 | -78.14 | 123.78 |  | 26.11 | 71.71 | -114.43 | 166.66 |  | 277.52 | 22.98 | 232.48 | 322.56 |
| 2032 | 85-89 years | 174.93 | 18.51 | 138.64 | 211.22 |  | 22.86 | 54.95 | -84.83 | 130.55 |  | 25.65 | 74.08 | -119.55 | 170.86 |  | 273.16 | 23.43 | 227.24 | 319.08 |
| 2033 | 85-89 years | 176.04 | 19.84 | 137.16 | 214.92 |  | 23.03 | 58.47 | -91.57 | 137.63 |  | 25.48 | 76.12 | -123.71 | 174.68 |  | 270.63 | 24.06 | 223.48 | 317.79 |
| 2034 | 85-89 years | 177.24 | 21.25 | 135.59 | 218.90 |  | 23.21 | 62.02 | -98.34 | 144.76 |  | 25.49 | 78.50 | -128.38 | 179.36 |  | 269.34 | 24.86 | 220.61 | 318.07 |
| 2035 | 85-89 years | 178.00 | 22.69 | 133.53 | 222.47 |  | 23.34 | 65.23 | -104.50 | 151.19 |  | 25.65 | 80.89 | -132.89 | 184.18 |  | 268.62 | 25.80 | 218.04 | 319.20 |
| 1990 | 90-94 years | 183.37 | 9.23 | 165.27 | 201.46 |  | 19.70 | 12.99 | -5.76 | 45.15 |  | 77.43 | 11.00 | 55.88 | 98.98 |  | 673.09 | 8.17 | 657.08 | 689.10 |
| 1991 | 90-94 years | 183.32 | 9.20 | 165.28 | 201.36 |  | 19.95 | 13.25 | -6.01 | 45.92 |  | 76.89 | 10.79 | 55.74 | 98.04 |  | 663.95 | 8.11 | 648.04 | 679.85 |
| 1992 | 90-94 years | 183.27 | 9.17 | 165.30 | 201.23 |  | 20.25 | 13.52 | -6.26 | 46.75 |  | 76.54 | 10.57 | 55.82 | 97.25 |  | 657.91 | 8.07 | 642.09 | 673.72 |
| 1993 | 90-94 years | 183.74 | 9.14 | 165.82 | 201.66 |  | 20.55 | 13.77 | -6.43 | 47.54 |  | 76.49 | 10.60 | 55.71 | 97.27 |  | 658.01 | 8.02 | 642.29 | 673.73 |
| 1994 | 90-94 years | 184.23 | 9.12 | 166.35 | 202.11 |  | 20.87 | 13.97 | -6.51 | 48.25 |  | 76.32 | 10.70 | 55.34 | 97.29 |  | 657.50 | 7.98 | 641.87 | 673.14 |
| 1995 | 90-94 years | 184.76 | 9.11 | 166.91 | 202.62 |  | 21.20 | 14.13 | -6.50 | 48.90 |  | 76.04 | 11.14 | 54.20 | 97.88 |  | 658.40 | 7.94 | 642.84 | 673.95 |
| 1996 | 90-94 years | 186.53 | 9.11 | 168.69 | 204.38 |  | 21.54 | 14.31 | -6.50 | 49.59 |  | 75.44 | 11.66 | 52.59 | 98.29 |  | 656.80 | 7.91 | 641.31 | 672.30 |
| 1997 | 90-94 years | 189.89 | 9.13 | 171.99 | 207.79 |  | 21.91 | 14.51 | -6.54 | 50.36 |  | 74.60 | 11.91 | 51.26 | 97.95 |  | 651.14 | 7.88 | 635.70 | 666.58 |
| 1998 | 90-94 years | 193.99 | 9.16 | 176.04 | 211.94 |  | 22.30 | 14.82 | -6.75 | 51.35 |  | 74.05 | 11.72 | 51.07 | 97.03 |  | 645.57 | 7.86 | 630.18 | 660.97 |
| 1999 | 90-94 years | 197.42 | 9.16 | 179.46 | 215.38 |  | 22.67 | 15.06 | -6.85 | 52.19 |  | 74.23 | 11.57 | 51.55 | 96.90 |  | 646.39 | 7.83 | 631.04 | 661.74 |
| 2000 | 90-94 years | 199.45 | 9.15 | 181.53 | 217.38 |  | 23.01 | 15.10 | -6.59 | 52.61 |  | 74.91 | 11.10 | 53.16 | 96.67 |  | 650.45 | 7.80 | 635.16 | 665.75 |
| 2001 | 90-94 years | 200.37 | 9.12 | 182.49 | 218.25 |  | 23.30 | 14.77 | -5.64 | 52.25 |  | 75.19 | 10.96 | 53.72 | 96.66 |  | 652.52 | 7.78 | 637.27 | 667.77 |
| 2002 | 90-94 years | 200.96 | 9.10 | 183.13 | 218.79 |  | 23.56 | 14.40 | -4.66 | 51.78 |  | 75.48 | 11.43 | 53.06 | 97.89 |  | 657.78 | 7.75 | 642.58 | 672.98 |
| 2003 | 90-94 years | 201.53 | 9.08 | 183.74 | 219.31 |  | 23.75 | 14.07 | -3.83 | 51.33 |  | 75.34 | 11.85 | 52.12 | 98.56 |  | 658.80 | 7.73 | 643.64 | 673.96 |
| 2004 | 90-94 years | 201.84 | 9.06 | 184.09 | 219.59 |  | 23.85 | 13.87 | -3.33 | 51.03 |  | 74.29 | 11.56 | 51.64 | 96.95 |  | 648.24 | 7.73 | 633.08 | 663.39 |
| 2005 | 90-94 years | 201.36 | 9.05 | 183.63 | 219.09 |  | 23.80 | 13.60 | -2.85 | 50.45 |  | 73.15 | 11.18 | 51.23 | 95.06 |  | 636.96 | 7.73 | 621.81 | 652.12 |
| 2006 | 90-94 years | 199.53 | 9.04 | 181.81 | 217.25 |  | 23.58 | 13.40 | -2.67 | 49.84 |  | 71.32 | 11.44 | 48.90 | 93.75 |  | 622.20 | 7.74 | 607.03 | 637.38 |
| 2007 | 90-94 years | 197.13 | 9.03 | 179.42 | 214.84 |  | 23.28 | 13.36 | -2.90 | 49.46 |  | 68.72 | 11.41 | 46.36 | 91.08 |  | 598.82 | 7.77 | 583.60 | 614.05 |
| 2008 | 90-94 years | 194.80 | 9.03 | 177.10 | 212.50 |  | 22.99 | 13.37 | -3.23 | 49.20 |  | 65.88 | 11.34 | 43.67 | 88.10 |  | 573.14 | 7.80 | 557.85 | 588.43 |
| 2009 | 90-94 years | 194.23 | 9.03 | 176.54 | 211.93 |  | 22.83 | 13.64 | -3.91 | 49.57 |  | 63.36 | 11.68 | 40.47 | 86.25 |  | 551.86 | 7.82 | 536.53 | 567.20 |
| 2010 | 90-94 years | 194.67 | 9.03 | 176.98 | 212.36 |  | 22.84 | 13.94 | -4.48 | 50.17 |  | 61.42 | 11.59 | 38.71 | 84.14 |  | 534.32 | 7.84 | 518.96 | 549.67 |
| 2011 | 90-94 years | 195.37 | 9.02 | 177.69 | 213.05 |  | 22.97 | 14.25 | -4.95 | 50.89 |  | 60.73 | 12.03 | 37.16 | 84.30 |  | 530.35 | 7.82 | 515.02 | 545.68 |
| 2012 | 90-94 years | 195.47 | 9.01 | 177.81 | 213.14 |  | 23.07 | 14.15 | -4.66 | 50.80 |  | 60.68 | 11.99 | 37.17 | 84.18 |  | 530.32 | 7.80 | 515.03 | 545.61 |
| 2013 | 90-94 years | 195.12 | 9.01 | 177.47 | 212.78 |  | 23.11 | 14.08 | -4.49 | 50.71 |  | 61.01 | 11.95 | 37.60 | 84.42 |  | 533.53 | 7.78 | 518.28 | 548.78 |
| 2014 | 90-94 years | 194.61 | 9.01 | 176.96 | 212.27 |  | 23.07 | 14.29 | -4.93 | 51.08 |  | 61.14 | 12.21 | 37.22 | 85.07 |  | 535.89 | 7.76 | 520.68 | 551.10 |
| 2015 | 90-94 years | 193.94 | 9.01 | 176.28 | 211.60 |  | 22.98 | 14.64 | -5.71 | 51.66 |  | 60.54 | 12.21 | 36.61 | 84.47 |  | 530.21 | 7.76 | 515.00 | 545.43 |
| 2016 | 90-94 years | 192.33 | 9.01 | 174.66 | 210.00 |  | 22.81 | 14.67 | -5.95 | 51.57 |  | 59.62 | 11.89 | 36.33 | 82.91 |  | 520.71 | 7.77 | 505.48 | 535.95 |
| 2017 | 90-94 years | 190.44 | 9.02 | 172.76 | 208.13 |  | 22.64 | 14.71 | -6.19 | 51.47 |  | 58.45 | 11.56 | 35.79 | 81.11 |  | 509.06 | 7.79 | 493.79 | 524.32 |
| 2018 | 90-94 years | 188.15 | 9.03 | 170.45 | 205.86 |  | 22.45 | 14.53 | -6.04 | 50.93 |  | 57.65 | 11.30 | 35.51 | 79.79 |  | 501.15 | 7.80 | 485.86 | 516.43 |
| 2019 | 90-94 years | 185.92 | 9.05 | 168.19 | 203.66 |  | 22.25 | 14.42 | -6.01 | 50.50 |  | 56.56 | 11.04 | 34.92 | 78.21 |  | 490.81 | 7.81 | 475.50 | 506.13 |
| 2020 | 90-94 years | 183.19 | 9.06 | 165.43 | 200.95 |  | 22.02 | 14.25 | -5.92 | 49.95 |  | 55.15 | 10.90 | 33.80 | 76.51 |  | 477.86 | 7.84 | 462.50 | 493.22 |
| 2021 | 90-94 years | 180.67 | 9.08 | 162.87 | 198.47 |  | 21.71 | 14.18 | -6.08 | 49.51 |  | 53.75 | 10.50 | 33.17 | 74.34 |  | 463.91 | 7.87 | 448.49 | 479.34 |
| 2022 | 90-94 years | 181.78 | 11.45 | 159.33 | 204.23 |  | 21.45 | 18.79 | -15.39 | 58.28 |  | 53.55 | 19.73 | 14.88 | 92.22 |  | 477.79 | 31.26 | 416.53 | 539.05 |
| 2023 | 90-94 years | 178.85 | 11.57 | 156.17 | 201.53 |  | 21.13 | 19.79 | -17.65 | 59.91 |  | 52.25 | 25.27 | 2.72 | 101.78 |  | 466.62 | 30.87 | 406.12 | 527.12 |
| 2024 | 90-94 years | 176.03 | 11.87 | 152.78 | 199.29 |  | 20.82 | 22.31 | -22.91 | 64.55 |  | 50.65 | 32.38 | -12.80 | 114.11 |  | 455.13 | 30.47 | 395.42 | 514.85 |
| 2025 | 90-94 years | 173.79 | 12.29 | 149.71 | 197.87 |  | 20.54 | 25.83 | -30.08 | 71.16 |  | 49.06 | 39.64 | -28.63 | 126.75 |  | 444.55 | 30.14 | 385.48 | 503.62 |
| 2026 | 90-94 years | 172.49 | 12.76 | 147.47 | 197.50 |  | 20.37 | 29.72 | -37.89 | 78.62 |  | 47.83 | 46.29 | -42.90 | 138.56 |  | 436.14 | 29.96 | 377.42 | 494.87 |
| 2027 | 90-94 years | 172.17 | 13.30 | 146.10 | 198.23 |  | 20.30 | 33.68 | -45.71 | 86.32 |  | 47.23 | 51.40 | -53.51 | 147.97 |  | 430.36 | 29.95 | 371.64 | 489.07 |
| 2028 | 90-94 years | 172.44 | 13.98 | 145.05 | 199.84 |  | 20.32 | 37.89 | -53.95 | 94.59 |  | 47.05 | 55.36 | -61.46 | 155.55 |  | 426.36 | 30.08 | 367.41 | 485.31 |
| 2029 | 90-94 years | 172.85 | 14.83 | 143.77 | 201.93 |  | 20.36 | 42.26 | -62.48 | 103.19 |  | 47.00 | 59.24 | -69.11 | 163.12 |  | 422.83 | 30.27 | 363.51 | 482.15 |
| 2030 | 90-94 years | 172.97 | 15.88 | 141.84 | 204.09 |  | 20.35 | 46.66 | -71.11 | 111.80 |  | 46.78 | 63.27 | -77.23 | 170.80 |  | 418.42 | 30.46 | 358.73 | 478.12 |
| 2031 | 90-94 years | 172.66 | 17.03 | 139.29 | 206.03 |  | 20.29 | 50.66 | -79.01 | 119.58 |  | 46.25 | 66.95 | -84.98 | 177.47 |  | 412.36 | 30.62 | 352.35 | 472.37 |
| 2032 | 90-94 years | 171.82 | 18.26 | 136.02 | 207.61 |  | 20.15 | 54.13 | -85.95 | 126.25 |  | 45.34 | 69.21 | -90.30 | 180.98 |  | 404.26 | 30.73 | 344.03 | 464.49 |
| 2033 | 90-94 years | 170.38 | 19.70 | 131.78 | 208.99 |  | 19.94 | 57.67 | -93.09 | 132.97 |  | 43.97 | 71.10 | -95.38 | 183.33 |  | 394.27 | 30.81 | 333.87 | 454.66 |
| 2034 | 90-94 years | 168.61 | 21.31 | 126.84 | 210.37 |  | 19.68 | 61.23 | -100.32 | 139.69 |  | 42.33 | 73.39 | -101.51 | 186.18 |  | 383.33 | 30.95 | 322.66 | 443.99 |
| 2035 | 90-94 years | 166.94 | 22.98 | 121.90 | 211.99 |  | 19.44 | 64.57 | -107.12 | 146.00 |  | 40.75 | 75.93 | -108.07 | 189.57 |  | 372.90 | 31.24 | 311.68 | 434.12 |
| 1990 | 95+ years | 180.28 | 9.93 | 160.82 | 199.75 |  | 15.08 | 16.14 | -16.55 | 46.71 |  | 101.73 | 10.25 | 81.63 | 121.82 |  | 827.82 | 11.23 | 805.80 | 849.84 |
| 1991 | 95+ years | 180.16 | 9.71 | 161.14 | 199.19 |  | 15.24 | 14.50 | -13.19 | 43.67 |  | 102.23 | 10.36 | 81.92 | 122.53 |  | 839.63 | 11.12 | 817.84 | 861.42 |
| 1992 | 95+ years | 180.08 | 9.70 | 161.07 | 199.09 |  | 15.41 | 13.69 | -11.42 | 42.25 |  | 102.59 | 10.69 | 81.65 | 123.54 |  | 834.28 | 10.95 | 812.83 | 855.74 |
| 1993 | 95+ years | 180.06 | 9.68 | 161.08 | 199.04 |  | 15.59 | 13.48 | -10.82 | 42.01 |  | 103.32 | 11.24 | 81.30 | 125.35 |  | 841.79 | 10.83 | 820.57 | 863.01 |
| 1994 | 95+ years | 180.18 | 9.66 | 161.25 | 199.11 |  | 15.78 | 13.56 | -10.79 | 42.35 |  | 103.79 | 11.75 | 80.76 | 126.81 |  | 850.93 | 10.71 | 829.94 | 871.92 |
| 1995 | 95+ years | 180.57 | 9.65 | 161.66 | 199.48 |  | 15.97 | 13.73 | -10.94 | 42.88 |  | 103.64 | 12.43 | 79.28 | 127.99 |  | 858.45 | 10.56 | 837.75 | 879.14 |
| 1996 | 95+ years | 181.78 | 9.70 | 162.76 | 200.80 |  | 16.17 | 13.93 | -11.13 | 43.47 |  | 102.53 | 12.73 | 77.58 | 127.48 |  | 849.02 | 10.34 | 828.76 | 869.28 |
| 1997 | 95+ years | 183.61 | 9.81 | 164.39 | 202.84 |  | 16.38 | 14.09 | -11.24 | 44.00 |  | 101.39 | 13.31 | 75.30 | 127.48 |  | 848.60 | 10.17 | 828.66 | 868.53 |
| 1998 | 95+ years | 185.87 | 9.92 | 166.43 | 205.31 |  | 16.59 | 14.19 | -11.22 | 44.40 |  | 100.37 | 13.21 | 74.47 | 126.27 |  | 838.65 | 9.97 | 819.11 | 858.20 |
| 1999 | 95+ years | 187.80 | 9.97 | 168.26 | 207.35 |  | 16.82 | 14.29 | -11.18 | 44.82 |  | 99.61 | 12.62 | 74.88 | 124.34 |  | 821.64 | 9.77 | 802.50 | 840.79 |
| 2000 | 95+ years | 188.72 | 9.92 | 169.28 | 208.16 |  | 17.06 | 14.35 | -11.06 | 45.18 |  | 99.32 | 11.56 | 76.66 | 121.98 |  | 804.92 | 9.57 | 786.16 | 823.68 |
| 2001 | 95+ years | 189.32 | 9.82 | 170.08 | 208.57 |  | 17.31 | 14.33 | -10.77 | 45.40 |  | 98.86 | 11.14 | 77.02 | 120.70 |  | 797.20 | 9.41 | 778.75 | 815.65 |
| 2002 | 95+ years | 189.86 | 9.70 | 170.85 | 208.88 |  | 17.58 | 14.22 | -10.30 | 45.46 |  | 98.29 | 10.52 | 77.67 | 118.91 |  | 787.57 | 9.26 | 769.42 | 805.72 |
| 2003 | 95+ years | 190.60 | 9.59 | 171.81 | 209.39 |  | 17.85 | 14.11 | -9.81 | 45.51 |  | 98.08 | 10.80 | 76.92 | 119.24 |  | 790.35 | 9.15 | 772.41 | 808.29 |
| 2004 | 95+ years | 191.43 | 9.48 | 172.84 | 210.01 |  | 18.12 | 14.01 | -9.34 | 45.59 |  | 97.64 | 10.78 | 76.51 | 118.78 |  | 786.68 | 9.04 | 768.96 | 804.41 |
| 2005 | 95+ years | 192.14 | 9.38 | 173.75 | 210.52 |  | 18.39 | 13.91 | -8.88 | 45.67 |  | 97.35 | 10.09 | 77.58 | 117.12 |  | 779.45 | 8.94 | 761.92 | 796.97 |
| 2006 | 95+ years | 192.49 | 9.27 | 174.32 | 210.67 |  | 18.63 | 13.72 | -8.26 | 45.52 |  | 96.58 | 10.25 | 76.49 | 116.67 |  | 774.81 | 8.85 | 757.47 | 792.14 |
| 2007 | 95+ years | 192.59 | 9.17 | 174.62 | 210.56 |  | 18.83 | 13.47 | -7.57 | 45.23 |  | 95.34 | 10.18 | 75.40 | 115.28 |  | 763.43 | 8.75 | 746.28 | 780.59 |
| 2008 | 95+ years | 192.67 | 9.11 | 174.81 | 210.54 |  | 18.97 | 13.28 | -7.06 | 45.01 |  | 94.31 | 10.66 | 73.41 | 115.21 |  | 759.85 | 8.67 | 742.86 | 776.84 |
| 2009 | 95+ years | 192.46 | 9.08 | 174.66 | 210.25 |  | 19.02 | 13.13 | -6.72 | 44.75 |  | 92.40 | 10.16 | 72.49 | 112.31 |  | 739.33 | 8.58 | 722.50 | 756.15 |
| 2010 | 95+ years | 191.97 | 9.06 | 174.21 | 209.74 |  | 18.93 | 13.07 | -6.69 | 44.55 |  | 90.27 | 9.65 | 71.36 | 109.18 |  | 718.02 | 8.51 | 701.33 | 734.70 |
| 2011 | 95+ years | 190.85 | 9.07 | 173.08 | 208.62 |  | 18.69 | 13.39 | -7.56 | 44.94 |  | 87.60 | 9.86 | 68.27 | 106.94 |  | 698.48 | 8.46 | 681.90 | 715.05 |
| 2012 | 95+ years | 189.49 | 9.09 | 171.68 | 207.30 |  | 18.35 | 14.01 | -9.12 | 45.82 |  | 84.40 | 10.30 | 64.21 | 104.59 |  | 673.96 | 8.41 | 657.47 | 690.45 |
| 2013 | 95+ years | 188.55 | 9.12 | 170.69 | 206.42 |  | 18.01 | 14.78 | -10.95 | 46.98 |  | 81.26 | 11.05 | 59.59 | 102.92 |  | 651.24 | 8.38 | 634.83 | 667.66 |
| 2014 | 95+ years | 189.09 | 9.18 | 171.10 | 207.09 |  | 17.80 | 15.56 | -12.70 | 48.30 |  | 79.32 | 13.72 | 52.44 | 106.21 |  | 650.68 | 8.33 | 634.35 | 667.01 |
| 2015 | 95+ years | 189.90 | 9.22 | 171.83 | 207.98 |  | 17.75 | 16.01 | -13.63 | 49.13 |  | 77.63 | 14.32 | 49.57 | 105.70 |  | 638.55 | 8.28 | 622.33 | 654.77 |
| 2016 | 95+ years | 190.77 | 9.24 | 172.67 | 208.87 |  | 17.84 | 16.16 | -13.82 | 49.51 |  | 77.39 | 15.04 | 47.91 | 106.86 |  | 641.01 | 8.22 | 624.91 | 657.11 |
| 2017 | 95+ years | 191.48 | 9.23 | 173.39 | 209.57 |  | 18.01 | 16.10 | -13.54 | 49.55 |  | 77.15 | 14.84 | 48.08 | 106.23 |  | 638.30 | 8.16 | 622.30 | 654.29 |
| 2018 | 95+ years | 191.94 | 9.22 | 173.88 | 210.01 |  | 18.15 | 15.95 | -13.12 | 49.42 |  | 77.32 | 14.23 | 49.44 | 105.21 |  | 637.35 | 8.11 | 621.45 | 653.25 |
| 2019 | 95+ years | 192.17 | 9.21 | 174.12 | 210.23 |  | 18.22 | 15.94 | -13.03 | 49.46 |  | 76.71 | 13.39 | 50.47 | 102.95 |  | 626.94 | 8.07 | 611.11 | 642.76 |
| 2020 | 95+ years | 191.54 | 9.20 | 173.51 | 209.57 |  | 18.19 | 15.90 | -12.97 | 49.35 |  | 75.40 | 13.14 | 49.63 | 101.16 |  | 615.75 | 8.04 | 599.99 | 631.52 |
| 2021 | 95+ years | 191.91 | 9.22 | 173.83 | 209.98 |  | 18.11 | 16.48 | -14.20 | 50.42 |  | 73.80 | 11.60 | 51.06 | 96.53 |  | 593.66 | 8.04 | 577.91 | 609.42 |
| 2022 | 95+ years | 188.44 | 11.55 | 165.81 | 211.08 |  | 17.93 | 17.01 | -15.42 | 51.28 |  | 73.60 | 18.34 | 37.65 | 109.55 |  | 620.52 | 39.97 | 542.18 | 698.86 |
| 2023 | 95+ years | 187.20 | 11.57 | 164.52 | 209.89 |  | 17.81 | 17.69 | -16.86 | 52.48 |  | 73.05 | 23.23 | 27.53 | 118.58 |  | 614.78 | 39.97 | 536.43 | 693.13 |
| 2024 | 95+ years | 185.66 | 11.72 | 162.69 | 208.64 |  | 17.67 | 19.45 | -20.44 | 55.79 |  | 72.26 | 29.69 | 14.06 | 130.46 |  | 607.27 | 39.86 | 529.14 | 685.39 |
| 2025 | 95+ years | 183.76 | 12.01 | 160.22 | 207.30 |  | 17.50 | 22.07 | -25.75 | 60.76 |  | 71.16 | 36.43 | -0.24 | 142.57 |  | 597.86 | 39.63 | 520.19 | 675.53 |
| 2026 | 95+ years | 181.46 | 12.45 | 157.07 | 205.86 |  | 17.31 | 25.19 | -32.05 | 66.67 |  | 69.95 | 42.72 | -13.78 | 153.68 |  | 586.90 | 39.33 | 509.81 | 663.98 |
| 2027 | 95+ years | 178.80 | 13.05 | 153.22 | 204.38 |  | 17.08 | 28.64 | -39.05 | 73.21 |  | 68.65 | 47.70 | -24.84 | 162.13 |  | 574.62 | 38.98 | 498.22 | 651.01 |
| 2028 | 95+ years | 175.91 | 13.86 | 148.75 | 203.08 |  | 16.83 | 32.41 | -46.69 | 80.35 |  | 66.99 | 51.69 | -34.33 | 168.31 |  | 561.18 | 38.56 | 485.59 | 636.77 |
| 2029 | 95+ years | 173.15 | 14.84 | 144.05 | 202.24 |  | 16.58 | 36.39 | -54.74 | 87.90 |  | 64.94 | 55.56 | -43.96 | 173.83 |  | 547.37 | 38.15 | 472.60 | 622.14 |
| 2030 | 95+ years | 170.94 | 15.95 | 139.68 | 202.20 |  | 16.36 | 40.39 | -62.80 | 95.52 |  | 62.89 | 59.43 | -53.59 | 179.38 |  | 534.64 | 37.84 | 460.47 | 608.81 |
| 2031 | 95+ years | 169.66 | 17.09 | 136.15 | 203.16 |  | 16.22 | 44.14 | -70.30 | 102.73 |  | 61.32 | 62.97 | -62.10 | 184.75 |  | 524.54 | 37.74 | 450.57 | 598.52 |
| 2032 | 95+ years | 169.35 | 18.28 | 133.52 | 205.17 |  | 16.17 | 47.60 | -77.14 | 109.47 |  | 60.55 | 65.45 | -67.73 | 188.83 |  | 517.58 | 37.88 | 443.33 | 591.84 |
| 2033 | 95+ years | 169.62 | 19.58 | 131.24 | 207.99 |  | 16.18 | 51.14 | -84.05 | 116.41 |  | 60.32 | 67.17 | -71.33 | 191.97 |  | 512.78 | 38.20 | 437.90 | 587.66 |
| 2034 | 95+ years | 170.02 | 21.01 | 128.83 | 211.20 |  | 16.21 | 54.68 | -90.97 | 123.39 |  | 60.27 | 69.11 | -75.18 | 195.71 |  | 508.54 | 38.62 | 432.86 | 584.23 |
| 2035 | 95+ years | 170.13 | 22.57 | 125.89 | 214.38 |  | 16.20 | 58.16 | -97.80 | 130.21 |  | 59.99 | 71.40 | -79.96 | 199.93 |  | 503.25 | 39.04 | 426.74 | 579.76 |
| ASR, Age-standardized rates; DALYs, Disability-adjusted life years;UI, Uncertainty Interval. | | | | | | | | | | | | | | | | | | | | |
